# C−H Oxygenation Reactions Enabled by Dual Catalysis with Electrogenerated Hypervalent Iodine Species and Ruthenium Complexes

**DOI:** 10.1002/anie.201914226

**Published:** 2020-01-09

**Authors:** Leonardo Massignan, Xuefeng Tan, Tjark H. Meyer, Rositha Kuniyil, Antonis M. Messinis, Lutz Ackermann

**Affiliations:** ^1^ Institut für Organische und Biomolekulare Chemie Georg-August-Universität Göttingen Tammannstraße 2 37077 Göttingen Germany

**Keywords:** C−H activation, electrocatalysis, hypervalent iodine species, oxygenation, ruthenium

## Abstract

The catalytic generation of hypervalent iodine(III) reagents by anodic electrooxidation was orchestrated towards an unprecedented electrocatalytic C−H oxygenation of weakly coordinating aromatic amides and ketones. Thus, catalytic quantities of iodoarenes in concert with catalytic amounts of ruthenium(II) complexes set the stage for versatile C−H activations with ample scope and high functional group tolerance. Detailed mechanistic studies by experiment and computation substantiate the role of the iodoarene as the electrochemically relevant species towards C−H oxygenations with electricity as a sustainable oxidant and molecular hydrogen as the sole by‐product. *para*‐Selective C−H oxygenations likewise proved viable in the absence of directing groups.

## Graphical abstract

**Dual catalysis**: The joint action of catalytic amounts of iodoarenes, ruthenium(II) carboxylates, and electricity enables the expedient C−H oxygenation of amides and ketones by weak coordination.

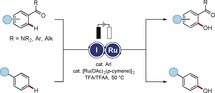

Organic electrochemistry has emerged as an increasingly viable tool for molecular synthesis.^1^ In addition to the unique potential of electrosynthesis, it is attractive also because of its storability and sustainable properties.^2^ Thus, the effective conversion of renewable electricity into value‐added chemical products holds major prospect for a sustainable energy economy.1h In this scenario, the merger of electrosynthesis and metal‐catalyzed C−H activation^3^ has recently been identified as a particularly powerful approach for the resource‐economic transformation of ubiquitous, but otherwise inert C−H bonds.^4^ Despite indisputable advances by the groups of Mei, Sanford, and Ackermann,^5^ electrochemical C−H oxygenations^6^ of challenging arenes by weak coordination^7^ have thus far proven elusive. The reported metal‐catalyzed C−H oxygenations largely require cost‐intensive palladium complexes and were inherently limited to strongly coordinating N‐directing groups, such as oximes and pyridines.^5^ In sharp contrast, C−H oxygenations by synthetically useful weak O‐coordination have not been realized in terms of sustainable electrocatalysis. Instead, highly reactive hypervalent iodine(III) reagents,^8^, ^9^ such as (diacetoxyiodo)benzene and [bis(trifluoroacetoxy)iodo]benzene, are required in overstoichiometric quantities, which calls for strong chemical oxidants for their synthesis and leads to equimolar amounts of undesired halogenated waste products during the C−H functionalization process. Contrarily, we herein present a mechanistically distinct strategy to address this molecular challenge, which orchestrates the catalytic electro‐regeneration^10^ of hypervalent iodine(III) reagents with ruthenium(II)‐catalyzed^11^, ^12^ C−H functionalizations (Figure 1). Salient features of our findings include a) the first electrocatalyzed C−H oxygenations by weak coordination, b) the user‐friendly electrochemical generation of hypervalent iodine reagents, c) ioda/ruthena‐electrocatalyzed C−H functionalizations that combine the advantages of ruthenium‐catalyzed C−H activation with electrocatalytic hypervalent iodine chemistry, and d) mechanistic studies by experiment, computation, cyclic voltammetry, and in operando NMR spectroscopy.

**Figure 1:**
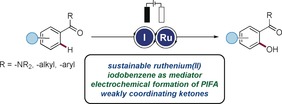
Orchestrating iodine(III)/ruthenium(II) electrocatalytic C−H activation.

We began our studies by exploring various reaction conditions for the envisioned electrochemical orchestrated C−H oxygenation of substrate **1 a** in a user‐friendly undivided cell (Table 1; see also Table S1 in the Supporting Information).^13^ Preliminary experimentation indicated that the reaction could indeed be accomplished in the presence of catalytic amounts of iodobenzene and ruthenium(II) carboxylate (entry 1). The ideal current density was found to be 2.67 mA cm^−2^ (entries 2 and 3), and the C−H activation proceeded equally well under constant potential conditions at a 2.0 V working potential (entry 4). Interestingly, a platinum plate as the anode was found to be beneficial in comparison to a reticulated vitreous carbon (RVC) anode (entries 5 and 6). Here, detailed IR‐spectroscopic analysis of the RVC anode indicated its electrochemical modification.^13^ Control experiments confirmed the essential role of electricity, the ruthenium catalyst, and the iodoarene (entries 7–9). Furthermore, iodobenzene was found to be the only co‐catalyst that enabled the desired C−H oxygenation, while benzoquinone (entry 10) as well as chlorine, bromine, or chalcogenide redox catalysis^14^ fell short in converting substrate **1 a** (entries 11 and 12).^12^ Notably, the replacement of electricity by the typical chemical oxidants *m*CPBA or Oxone resulted in considerably inferior efficacy (entries 13 and 14).

**Table 1:** Optimization of the iodine/ruthenium‐electrocatalyzed C−H oxygenation.^[a]^ 
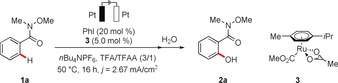

Entry	Deviation from the standard conditions	Yield [%]^[b]^
**1**	**none**	**80**
2	*j*=4.00 mA cm^−2^	51
3	*j*=1.33 mA cm^−2^	37
4	CPE at 2.0 V	86^[c]^
5	RVC anode instead of Pt	24
6	RVC anode instead of Pt, without PhI	28
7	no current	–
8	without [Ru]	–
9	without PhI	–
10	1,4‐benzoquinone instead of PhI	–
11	PhBr or PhCl instead of PhI	–
12	PhS‐SPh or PhSe‐SePh instead of PhI	–
13	*m*CPBA instead of electricity	15
14	Oxone instead of electricity	32

[a] Undivided cell, **1 a** (0.50 mmol), iodobenzene (20 mol %), **3** (5.0 mol %), electrolyte (1.0 equiv), solvent (3.0 mL), 50 °C, 16 h, Pt plate electrodes (10 mm×15 mm×0.125 mm), constant current electrolysis (CCE) at 4 mA. [b] Yield of isolated product. [c] CPE=constant potential electrolysis at 2.0 V vs. Ag/Ag^+^. TFA=trifluoroacetic acid. TFAA=trifluoroacetic anhydride.

With optimized reaction conditions in hand, we probed the versatility of the co‐catalytic^15^ electrochemical C−H oxygenation system with a representative set of weakly O‐coordinating amides **1** (Scheme 1). Differently decorated amides bearing *para* and *meta* substituents were efficiently transformed into products **2 a**–**k**. Useful electrophilic functional groups, such as chloro, bromo, or even iodo substituents, as well as sensitive benzyl chlorides were fully tolerated, an invaluable asset in terms of future late‐stage modifications (**2 l**–**p**). It is noteworthy that the reaction was not limited to Weinreb amides **1**. Indeed, differently substituted amides **1 q**–**w** were efficiently converted into the corresponding oxygenated arenes **2** with excellent efficiency (Scheme 2).

**Scheme 1:**
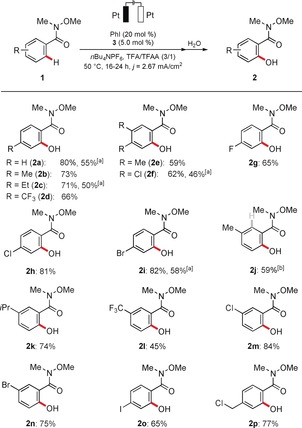
Electro‐catalyzed C−H activation of Weinreb amides **1**. [a] Without *n*Bu_4_NPF_6_. [b] Regioisomer **2 j′** was isolated in 2 % yield.

**Scheme 2:**
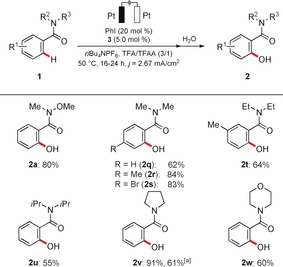
Electrooxidative C−H activation of various amides **1**. [a] Without *n*Bu_4_NPF_6_.

The outstanding robustness of the iodine(III)/ruthenium(II)‐catalyzed C−H oxygenation process was further highlighted by its ability to also transform weakly coordinating ketones **4** (Scheme 3).^7^ The versatility of the electrocatalysis was hence reflected by the successful use of differently decorated ketones **4**. Thereby, various substitution patterns were well tolerated to deliver products **5 e**–**j**. The inherent selectivity features were probed by intramolecular competition experiments with diaryl ketones **4 k** and **4 l**, which were both functionalized with excellent mono‐ and chemoselectivity. The regioselectivity of the C−H transformation of the unsymmetrically substituted substrate **4 l** further illustrates the inherent preference for electron‐rich arenes (see below).

**Scheme 3:**
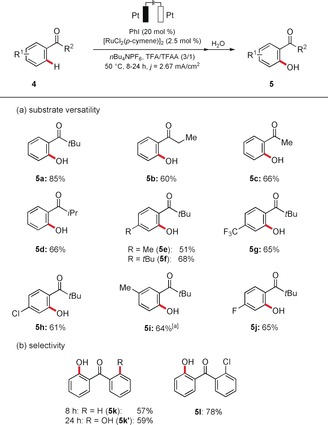
Ruthena‐electrocatalyzed C−H activation of ketones **4**. [a] 3 mA.

Moreover, the ruthena‐electrocatalyzed C−H oxygenation enabled the modification of synthetically useful pyrazole derivatives **6** (Scheme 4).

**Scheme 4:**
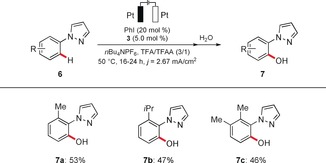
Ruthena‐electrocatalyzed C−H activation of pyrazolyl substrates **6**.

It is noteworthy that the ruthena‐electrocatalyzed C−H functionalization was not limited to chelation‐assisted *ortho* oxygenation. Indeed, directing‐group‐free6f functionalization in the challenging remote position was likewise sequentially accomplished with excellent levels of site selectivity, while the ruthenium catalyst was found to be essential (Scheme 5).

**Scheme 5:**
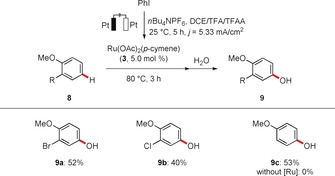
Directing‐group‐free remote C−H oxygenation. DCE=1,2‐dichloroethane.

The scalability of the orchestrated electrochemical C−H oxygenation was demonstrated by the gram‐scale synthesis of product **2 a** without loss of efficiency (Scheme 6).

**Scheme 6:**
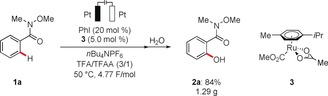
Gram‐scale iodine/ruthena‐electrocatalyzed C−H oxygenation.

Given the efficiency of the unprecedented electrochemical C−H oxygenation system, we became interested in delineating its mode of action. First, the use of a deuterated solvent in the catalytic reaction revealed the reversibility of the C−H activation step (Scheme 7 a). This finding contrasts with C−H oxygenations enabled by the chemical oxidant PIFA, for which H/D scrambling was not observed.6g Second, kinetic studies provided strong support for a fast and reversible C−H metalation with a minor kinetic isotope effect (KIE) of only *k*_H_/*k*_D_≈1.6.^13^ These observations overall suggest that not the C−H activation, but rather the oxidation of the cyclometalated species is the rate‐determining step. These experimental data are again in contrast with the use of chemical oxidants, for which the C−H activation was proposed to be the rate‐limiting step with a KIE of *k*_H_/*k*_D_≈3.0.6f Third, competition experiments, using either the Weinreb amides **1 b** and **1 d** or the difunctionalized ketone **4 m**, highlighted that electron‐rich substrates are preferentially functionalized (see above; Scheme 7 b), which can be rationalized in terms of a base‐assisted internal electrophilic‐type substitution (BIES) being operative for the C−H metalation.^16^ Forth, an intramolecular competition experiment with substrate **1 x** revealed the Weinreb amide as a more powerful coordinating group for the iodine/ruthenium‐co‐catalyzed C−H transformation (Scheme 7 c). Fifth, we probed the possibility of *p*‐cymene dissociation.^17^ Detailed GC analysis did not provide evidence for free *p*‐cymene in the reaction mixture at any point during the reaction.^12^

**Scheme 7:**
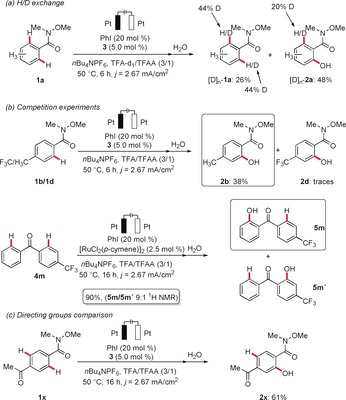
Summary of the mechanistic findings.

Next, we studied the reaction profile of the direct anodic generation of the hypervalent iodine reagents by in operando NMR spectroscopy (Figure 2 a).^12^ This combination of electrochemistry and in situ spectroscopy enabled us to study the generation of otherwise unstable electrochemically generated iodine(III) reagents. Initially, the anodic oxidation of iodobenzene in trifluoroethanol (TFE) was monitored and showed almost full conversion of the aryl halide after 2.5 h at 10 mA (Figure 2 a, i).10a Subsequently, the anodic generation of hypervalent iodine **11 b** from TFA and iodobenzene was completed with only slightly prolonged reaction times within 3 h (Figure 2 a, ii). Thereafter, we examined the electrochemical C−H oxygenation by means of cyclic voltammetry (Figure 2 b).^12^ To this end, the oxidation of different aryl halides was recorded.^13^ In trifluoroacetic acid, only iodobenzene underwent irreversible anodic oxidation with an onset potential of *E=*1.25 V vs. ferrocene. By means of computation we also confirmed that the oxidation potential of the iodobenzene is 200 mV lower than that of the ruthenium(II/IV) manifold,^12^ substantiating the iodine co‐catalysis. Notably, other organic halides are known to undergo oxidation at considerably higher potentials,^12^, ^18^ reflecting the unique catalytic competence of iodine reagents (see above, Table 1). The amide **1 a** and electron‐deficient iodoarenes showed significantly higher potentials for anodic oxidation than unsubstituted and electron‐rich iodoarenes. A mixture of iodobenzene and amide **1 a** did not lead to significant changes in the voltammogram, which is in agreement with the control experiments summarized in Table 1. Cyclic voltammetry of the independently prepared ruthenacycle **10** in DCE provided support for its facile oxidation.^13^

**Figure 2:**
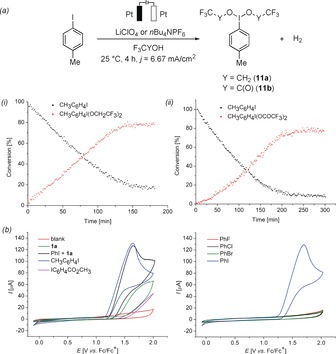
a) In operando NMR studies under constant current electrolysis at 10 mA in trifluoroethanol (TFE) or trifluoroacetic acid (TFA) respectively. Conversion determined by ^1^H NMR analysis using CH_2_Br_2_ as the internal standard. i) Reaction profile of the anodic formation of CH_3_C_6_H_4_I(OCH_2_CF_3_)_2_ (**11 a**). ii) Reaction profile of the anodic synthesis/formation of CH_3_C_6_H_4_I(OCOCF_3_)_2_ (**11 b**). b) Cyclic voltammetry (TFA, 0.1 m *n*Bu_4_NPF_6_, 100 mV s^−1^) using glassy carbon as the working electrode. Cyclic voltammograms of different reaction components and their mixtures as well as of different haloarenes.

Based on our detailed mechanistic studies, we propose a plausible catalytic cycle for the ioda/ruthena‐electrocatalyzed C−H oxygenation (Scheme 8). The catalytic cycle is initiated by C−H activation on amide **1** by a ruthenium(II) carboxylate. Meanwhile, iodobenzene undergoes a two‐electron‐transfer anodic oxidation to generate the hypervalent iodine(III) species. The iodine(III) reagent then mediates the oxidation of **12** by carboxylate transfer to the ruthena(II)cycle, delivering ruthenium(IV) intermediate **13**, which then undergoes rapid oxidatively induced reductive elimination to furnish product **2** after hydrolysis. Lastly, the regeneration of the active catalyst takes place. The formation of molecular hydrogen as the only stoichiometric by‐product was confirmed by GC headspace analysis,^12^ and bears great potential for paired electrochemical approaches.^19^

**Scheme 8:**
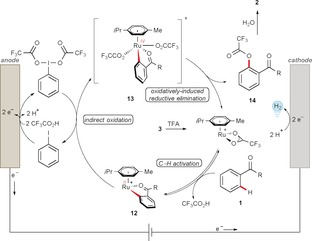
Plausible catalytic cycle.

In conclusion, we have devised a novel electrochemical co‐catalytic system for the C−H oxygenation of synthetically useful amides and ketones by challenging weak O‐coordination. The versatile iodine(III)/ruthenium(II)‐electrocatalyzed C−H functionalization was enabled by orchestrating the catalytic generation of hypervalent iodine(III) reagents with sustainable electricity as a cost‐effective terminal oxidant, with the formation of molecular hydrogen as the sole by‐product. Detailed mechanistic studies by experiment, computation, and flow‐NMR spectroscopy provided—in contrast to chemical oxidation—support for a fast and reversible C−H ruthenation. The ruthenium catalyst also allowed for electrochemical remote C−H oxygenations in the absence of directing groups.

## Conflict of interest

The authors declare no conflict of interest.

## Supporting information

As a service to our authors and readers, this journal provides supporting information supplied by the authors. Such materials are peer reviewed and may be re‐organized for online delivery, but are not copy‐edited or typeset. Technical support issues arising from supporting information (other than missing files) should be addressed to the authors.

Supplementary
