# Supplementary material for: C−H Oxygenation Reactions Enabled by Dual Catalysis with Electrogenerated Hypervalent Iodine Species and Ruthenium Complexes
Source: Angew Chem Int Ed Engl. 2020 Jan 9;59(8):3184–9. doi: 10.1002/anie.201914226 (PMC7027769; doi:10.1002/anie.201914226)
Supplement: Supplementary file 1 — Supplementary [file ANIE-59-3184-s001.pdf]

Angewandte Chemie
Eine Zeitschrift der Gesellschaft Deutscher Chemiker

Supporting Information

C–H Oxygenation Reactions Enabled by Dual Catalysis with Electrogenerated Hypervalent Iodine Species and Ruthenium Complexes

*Leonardo Massignan[+], Xuefeng Tan[+], Tjark H. Meyer, Rositha Kuniyil, Antonis M. Messinis, and Lutz Ackermann**

anie_201914226_sm_miscellaneous_information.pdf

Table of Contents

General Remarks S-3

Optimization of Ioda/Ruthenaelectro-Catalyzed C–H Activation S-4

General Procedure A for Ioda/Ruthenaelectro-Catalyzed C–H Oxygenation of Amides S-7

General Procedure B for Ioda/Ruthenaelectro-Catalyzed C–H Oxygenation of Ketones S-7

General Procedure C for Ioda/Ruthenaelectro-Catalyzed C–H Oxygenation in *para*-Position S-8

Characterization Data of Products S-9

Mechanistic Studies S-32

Cost of Goods Analysis S-53

Identification of the Trifluoroacetate Product after C–H Acyloxylation S-55

IR Spectroscopic Analysis of the Carbon Anode S-57

Synthesis of the Cyclometalated Complex **10** S-58

Computational Studies S-66

References S-78

NMR Spectra S-81

General Remarks

Iodobenzene was obtained from Acros Organics and used without further purification (99%, pure). Ethyl acetate, *n*hexane and dichloromethane were distilled before use. Platinum electrodes (10 mm × 15 mm × 0.125 mm, 99.95%; obtained from ESG-Edelmetall-Handel GmbH & Co. KG) and RVC electrodes (10 mm × 15 mm × 6 mm, SIGRACELL® GFA 6 EA, obtained from SGL Carbon, Wiesbaden, Germany) were connected using stainless steel adapters. Electrocatalysis was conducted using an AXIOMET AX-3003P potentiostat in constant current mode. CV and RDE studies were performed using a Metrohm Autolab PGSTAT204 workstation and Nova 2.1 software. The ^{1}H-NMR-flow was performed on a Magritek Spinsolve 60^{ULTRA} (from Magritek GmbH, Germany). Yields refer to isolated compounds, estimated to be >95% pure as determined by ^{1}H-NMR spectroscopy. Chromatography was carried out on Merck silica gel 60 (40–63 µm). NMR spectra were recorded on a Varian Mercury VX 300, Inova 500 or Bruker Avance III 300, Avance III 400 and Avance III HD 500 in the solvent indicated; chemical shifts (δ) are provided in ppm relative to the residual solvent peak. All IR spectra were recorded on a Bruker FT-IR Alpha-P device. EI-MS was recorded on Jeol AccuTOF at 70 eV, ESI-MS on Bruker MicrOTOF and maXis. GC-MS was recorded on Agilent 7890B and Agilent 5977B. M. p.: Stuart melting point apparatus SMP3, Barloworld Scientific, values are uncorrected.

Optimization of Ioda/Ruthenaelectro-Catalyzed C–H Activation

Table S1. Optimization of ioda/ruthenaelectro-catalyzed C–H oxygenation.[a]

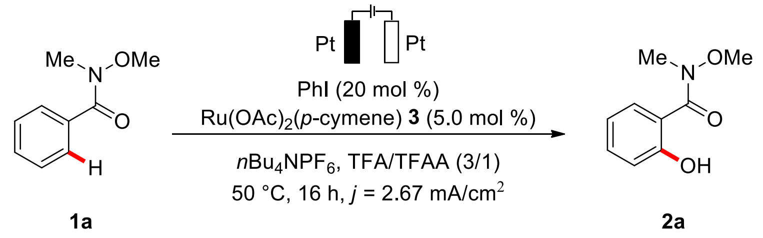

Entry	Deviation from standard conditions	Yield[b]
**1**	**---**	**80%**
2	$[RuCl_2(p\text{-cymene})]_2$ (2.5 mol %)	77%
3	$Ru(O_2CMes)_2(p\text{-cymene})$ (5.0 mol %)	59%
4	$[Ru(NC\mathit{t}Bu)_6][PF_6]_2$ (5.0 mol %)	---
5	$RuCl_3$ (5.0 mol %)	---
6	$j = 4.00\ mA{\cdot}cm^{-2}$	51%
7	$j = 1.33\ mA{\cdot}cm^{-2}$	37%
8	CPE at 2.0 V	86%[c]
9	CPE at 1.5 V	60%[c]
10	CPE at 1.0 V	---[c]
11	RVC anode instead of Pt	24%
12	Steel electrodes instead of Pt	---
13	Ni electrodes instead of Pt	---
14	No current	---
15	Without [Ru]	---

Entry	Deviation from standard conditions	Yield[b]
16	Without PhI	---
17	RVC anode instead of Pt, without PhI	28%
18	Under N_2	70%
19	25 ℃ instead of 50 ℃	50%
20	nBu$_4$NPF$_6$ (0.5 equiv)	52%
21	4-$CH_3OC_6H_4I$ instead of PhI	55%
22	PhBr instead of PhI	---
23	PhCl instead of PhI	---
24	1,4-Benzoquinone instead of PhI	---
25	PhS-SPh or PhSe-SePh instead of PhI	---
26	*m*CPBA instead of electricity	15%
27	Oxone instead of electricity	32%

[a] Undivided cell, **1a** (0.50 mmol), iodobenzene (20 mol %), **3** (5.0 mol %) nBu$_4$NPF$_6$ (1.0 equiv), TFA (2.25 mL), TFAA (0.75 mL), 50 ℃, 16 h, Pt-plate electrodes (10 mm x 15 mm x 0.125 mm), constant current electrolysis (CCE) at 4 mA. [b] Yield of isolated product. [c] CPE = constant potential electrolysis vs Ag/Ag^+. TFA = trifluoroacetic acid. TFAA = trifluoroacetic anhydride.

Table S2. Optimization of ioda/ruthenaelectro-catalyzed C–H oxygenation of 2,2-dimethyl-1-phenylpropan-1-one (**4a**).[a]

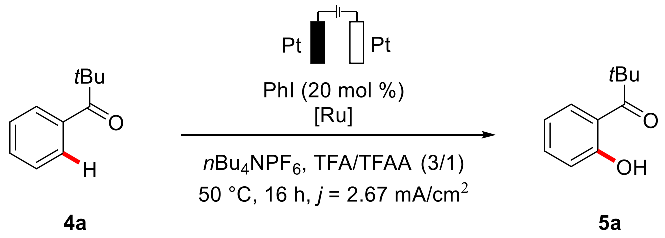

Entry	[Ru] (x mol %)	Time (h)	Yield[b]
**1**	$Ru(OAc)_2$(*p*-cymene) (5.0)	20	78%
**2**	$Ru(MesCOO)_2$(*p*-cymene) (5.0)	20	86%
**3**	[$RuCl_2$(*p*-cymene)]$_2$ (2.5)	20	85%
**4**	[$RuCl_2$(*p*-cymene)]$_2$ (2.5)	12	77%
**5**	[$RuCl_2$(*p*-cymene)]$_2$ (2.5)	16	84%

[a] Undivided cell, **4a** (0.50 mmol), iodobenzene (20 mol %), nBu$_4$NPF$_6$ (1.0 equiv), TFA (2.25 mL), TFAA (0.75 mL), 50 ℃, 16 h, Pt-plate electrodes (10 mm x 15 mm x 0.125 mm), constant current electrolysis (CCE) at 4 mA. [b] Yields of isolated products.

General Procedure A for Ioda/Ruthenaelectro-Catalyzed C–H Oxygenation of Amides

The electrocatalysis was carried out in an undivided cell with platinum electrodes (10 mm x 15mm x 0.125 mm). The cell was charged with amide **1** (0.50 mmol, 1.0 equiv), PhI (20.4 mg, 20 mol %), $Ru(OAc)_2$(*p*-cymene) (8.8 mg, 5.0 mol %) and *n*Bu_4NPF_6 (194 mg, 1.0 equiv) in TFA/TFAA (3:1, 3.0 mL). Electrocatalysis was performed at 50 ℃ with a constant current of 4 mA maintained for 16 h. After the reaction was completed, a saturated aqueous solution of $NaHCO_3$ (25 mL) was added and the reaction mixture was extracted with EtOAc (3 × 15 mL). The combined organic layers were washed with brine (25 mL), dried over Na_2SO_4, filtered and concentrated *in vacuo*. The crude product was purified by column chromatography on silica gel.

General Procedure B for Ioda/Ruthenaelectro-Catalyzed C–H Oxygenation of Ketones

The electrocatalysis was carried out in an undivided cell with platinum electrodes (10 mm x 15mm x 0.125 mm). The cell was charged with ketone **4** (0.50 mmol, 1.0 equiv), PhI (20.4 mg, 20 mol %), $[RuCl_2$(*p*-cymene)$]_2$ (7.7 mg, 2.5 mol %) and *n*Bu_4NPF_6 (194 mg, 1.0 equiv) in TFA/TFAA (3:1, 3.0 mL). Electrocatalysis was performed at 50 ℃ with a constant current of 4 mA maintained for 16-24 h. After the reaction was completed, a saturated aqueous solution of $NaHCO_3$ (25 mL) was added and the reaction mixture was extracted with EtOAc (3 × 15 mL). The combined organic layers were washed with brine (25 mL), dried over Na_2SO_4, filtered and concentrated *in vacuo*. The crude product was purified by column chromatography on silica gel.

General Procedure C for Ioda/Ruthenaelectro-Catalyzed C–H Oxygenation in *para*-Position

The electrocatalysis was carried out in an undivided cell with platinum electrodes (10 mm x 15 mm x 0.125 mm). The cell was charged with PhI (122 mg, 1.2 equiv), and nBu$_4$NPF$_6$ (194 mg, 1.0 equiv) in TFA/TFAA/DCE (1:2:15, 3.6 mL). Electrocatalysis was performed at room temperature with a constant current of 8 mA for 5 h. Subsequently, anisole **8** (0.50 mmol, 1.0 equiv) and Ru(OAc)$_2$(*p*-cymene) (8.8 mg, 5.0 mol %) were added and the reaction was heated at 80 °C for 3 h. After the reaction was completed, water (25 mL) was added and the reaction mixture was extracted with EtOAc (3 × 15 mL). The combined organic layers were washed with brine (25 mL), dried over Na_2SO_4, filtered and concentrated *in vacuo*. The crude product was purified by column chromatography on silica gel.

Characterization Data of Products

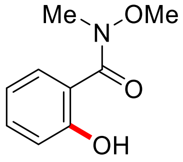

2-Hydroxy-*N*-methoxy-*N*-methylbenzamide (2a): The general procedure **A** was followed using *N*-methoxy-*N*-methylbenzamide **1a** (82.5 mg, 0.50 mmol). Isolation by column chromatography (*n*hexane/EtOAc: 5/1→3/1) yielded **2a** (72.5 mg, 80%) as a colourless oil. The same procedure without the addition of electrolyte (nBu_4NPF_6) yielded **2a** (49.8 mg, 55%) as a colourless oil. **^{1}H-NMR** (400 MHz, $CDCl_3$) δ = 10.40 (s, 1H), 7.89 (dd, J = 8.1, 1.7 Hz, 1H), 7.34 (ddd, J = 8.4, 7.2, 1.7 Hz, 1H), 6.95 (dd, J = 8.4, 1.3 Hz, 1H), 6.82 (ddd, J = 8.1, 7.2, 1.3 Hz, 1H), 3.61 (s, 3H), 3.37 (s, 3H). **^{13}C-NMR** (100 MHz, $CDCl_3$) δ = 169.8 (C_q), 160.5 (C_q), 133.7 (CH), 129.4 (CH), 118.6 (CH), 117.9 (CH), 114.4 (C_q), 61.2 (CH_3), 34.1 (CH_3). **IR** (ATR): 2936, 1625, 1590, 1452, 1249, 973, 754, 527 cm^{-1}. **MS** (ESI) *m/z* (relative intensity): 385 (30) $[2M+Na]^+$, 204 (100) $[M+Na]^+$, 182 (100) $[M+H]^+$. **HR-MS** (ESI) *m/z* calc. for $C_9H_{12}NO_3^+$ $[M+H]^+$ 182.0814, found 182.0812. The analytical data are in accordance with these reported in the literature.[1]

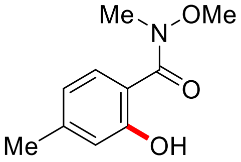

2-Hydroxy-*N*-methoxy-*N*,4-dimethylbenzamide (2b): The general procedure **A** was followed using *N*-methoxy-*N*,4-dimethylbenzamide **1b** (89.6 mg, 0.50 mmol). Isolation by column chromatography (*n*hexane/EtOAc: 5/1→3/1) yielded **2b** (71.6 mg, 73%) as a pale yellow oil. **^{1}H-NMR** (400 MHz, $CDCl_3$) δ = 11.36 (s, 1H), 7.88 (d, J = 8.3 Hz, 1H), 6.82 (d, J = 1.8 Hz, 1H), 6.67 (dd, J = 8.3, 1.8 Hz, 1H), 3.67 (s, 3H), 3.41 (s, 3H), 2.34 (s, 3H). **^{13}C-NMR** (100 MHz, $CDCl_3$) δ = 170.0 (C_q), 161.2 (C_q), 144.9 (C_q), 129.3 (CH), 119.7 (CH), 118.2 (CH), 111.5 (C_q), 61.1 (CH_3), 34.0 (CH_3), 21.6 (CH_3). **IR** (ATR): 2933, 1584, 1502, 1432, 1351, 1201, 949, 594 cm^{-1}. **MS** (ESI)

m/z (relative intensity): 413 (10) [2M+Na]$^+$, 218 (90) [M+Na]$^+$, 196 (100) [M+H]$^+$. **HR-MS** (ESI) *m/z* calc. for $C_{10}H_{14}NO_3^+$ [M+H]$^+$ 196.0969, found 196.0968. The analytical data are in accordance with these reported in the literature.[1]

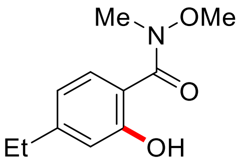

4-Ethyl-2-hydroxy-*N*-methoxy-*N*-methylbenzamide (2c): The general procedure **A** was followed using 4-ethyl-*N*-methoxy-*N*-methylbenzamide **1c** (96.6 mg, 0.50 mmol). Isolation by column chromatography (*n*hexane/EtOAc: 5/1→3/1) yielded **2c** (74.5 mg, 71%) as a yellow oil. The same procedure without the addition of electrolyte (nBu_4NPF_6) yielded **2c** (52.3 mg, 50%) as yellow oil. **^{1}H-NMR** (400 MHz, $CDCl_3$) δ = 11.37 (s, 1H), 7.90 (d, J = 8.4 Hz, 1H), 6.84 (d, J = 1.7 Hz, 1H), 6.70 (dd, J = 8.4, 1.7 Hz, 1H), 3.67 (s, 3H), 3.41 (s, 3H), 2.64 (q, J = 7.6 Hz, 2H), 1.25 (t, J = 7.6 Hz, 3H). **^{13}C-NMR** (100 MHz, $CDCl_3$) δ = 170.0 (C_q), 161.4 (C_q), 151.0 (C_q), 129.4 (CH), 118.5 (CH), 116.9 (CH), 111.7 (C_q), 61.1 (CH_3), 34.1 (CH_3), 28.8 (CH_2), 14.7 (CH_3). **IR** (ATR): 2968, 1632, 1584, 1499, 1433, 1356, 1202, 974 cm^{-1}. **MS** (ESI) *m/z* (relative intensity): 232 (100) [M+Na]$^+$, 210 (100) [M+H]$^+$. **HR-MS** (ESI) *m/z* calc. for $C_{11}H_{16}NO_3^+$ [M+H]$^+$ 210.1129, found 210.1125. The analytical data are in accordance with these reported in the literature.[1]

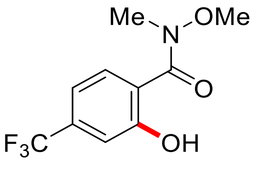

2-Hydroxy-*N*-methoxy-*N*-methyl-4-(trifluoromethyl)benzamide (2d): The general procedure **A** was followed using *N*-methoxy-*N*-methyl-4-(trifluoromethyl)benzamide (**1d**) (116 mg, 0.50 mmol). Purification by column chromatography on silica gel (*n*hexane/EtOAc: 5/1) yielded **2d** (82.5 mg, 66%) as a light viscous liquid. **^{1}H-NMR** (400 MHz, $CDCl_3$) δ = 11.27 (s, 1H), 8.06 (d, J = 8.5 Hz, 1H), 7.21 (d, J = 1.9 Hz, 1H), 7.05 (dd, J = 8.5, 1.9 Hz, 1H), 3.62 (s, 3H), 3.39 (s, 3H). **^{13}C-NMR** (100 MHz,

$CDCl_3$) δ = 168.4 (C_q), 160.9 (C_q), 135.0 (q, $^2J_{C–F}$ = 32.8 Hz, C_q), 130.3 (CH), 123.3 (q, $^1J_{C–F}$ = 272.8 Hz, C_q), 117.1 (C_q), 115.2 (q, $^3J_{C–F}$ = 4.0 Hz, CH), 114.9 (q, $^3J_{C–F}$ = 3.7 Hz, CH), 61.4 (CH_3), 33.7 (CH_3). **^{19}F-NMR** (282 MHz, $CDCl_3$) δ = –63.76 (s). **IR** (ATR): 2941, 1635, 1593, 1424, 1235, 1165, 1073, 923 cm^{-1}. **MS** (ESI) *m/z* (relative intensity): 248 (100) [M–H]$^-$, 218 (30), 161 (20). **HR-MS** (ESI) *m/z* calc. for $C_{10}H_9F_3NO_3^-$ [M–H]$^-$ 248.0529, found 248.0535. The analytical data are in accordance with these reported in the literature.[1]

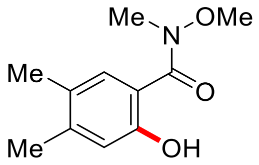

2-Hydroxy-*N*-methoxy-*N*-4,5-trimethylbenzamide (2e): The general procedure **A** was followed using *N*-methoxy-*N*-3,4-trimethylbenzamide (**1e**) (96.5 mg, 0.50 mmol). Purification by column chromatography on silica gel (*n*hexane/EtOAc: 5/1) yielded **2e** (61.5 mg, 59%) as a white solid. **M.p.**: 102–104 °C. **^{1}H-NMR** (400 MHz, $CDCl_3$) δ = 10.38 (s, 1H), 7.67 (s, 1H), 6.76 (s, 1H), 3.63 (s, 3H), 3.36 (s, 3H), 2.21 (s, 3H), 2.16 (s, 3H). **^{13}C-NMR** (100 MHz, $CDCl_3$) δ = 170.2 (C_q), 159.1 (C_q), 143.5 (C_q), 129.7 (CH), 126.6 (C_q), 118.6 (CH), 111.7 (C_q), 61.0 (CH_3), 34.2 (CH_3), 20.1 (CH_3), 19.0 (CH_3). **IR** (ATR): 2985, 1736, 1373, 1235, 1044, 916, 730, 607 cm^{-1}. **MS** (ESI) *m/z* (relative intensity): 438 (26) [2M+Na]$^+$, 232 (100) [M+Na]$^+$, 210 (90) [M+H]$^+$. **HR-MS** (ESI) *m/z* calc. for $C_{11}H_{16}NO_3^+$ [M+H]$^+$ 210.1127, found 210.1125.

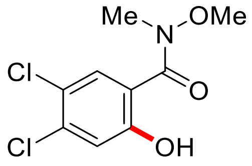

4,5-Dichloro-2-hydroxy-*N*-methoxy-*N*-methylbenzamide (2f): The general procedure **A** was followed using 3,4-dichloro-*N*-methoxy-*N*-methylbenzamide (**1f**) (117 mg, 0.50 mmol). Purification by column chromatography on silica gel (*n*hexane/EtOAc: 5/1) yielded **2f** (77.2 mg, 62%) as a white solid. The same procedure without the addition of electrolyte (nBu$_4$NPF$_6$) yielded **2f** (57.5 mg, 46%)

as a white solid. **M.p.**: 114–117 °C. **^{1}H-NMR** (400 MHz, $CDCl_3$) δ = 11.51 (s, 1H), 8.17 (s, 1H), 7.14 (s, 1H), 3.70 (s, 3H), 3.42 (s, 3H). **^{13}C-NMR** (100 MHz, $CDCl_3$) δ = 167.8 (C_q), 160.4 (C_q), 137.6 (C_q), 130.7 (CH), 121.9 (C_q), 119.8 (CH), 113.7 (C_q), 61.5 (CH_3), 33.7 (CH_3). **IR** (ATR): 2936, 1625, 1570, 1454, 1334, 1180, 978, 649 cm^{-1}. **MS** (ESI) *m/z* (relative intensity): 521 (10) $[2M+Na]^+$ (^{35}Cl), 251 (100) $[M+H]^+$ (^{35}Cl). **HR-MS** (ESI) *m/z* calc. for $C_9H_{10}{}^{35}Cl_2NO_3^+$ $[M+H]^+$ 250.0030, found 250.0032.

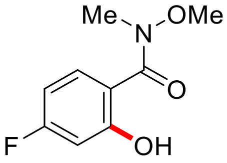

4-Fluoro-2-hydroxy-*N*-methoxy-*N*-methylbenzamide (2g): The general procedure **A** was followed using 4-fluoro-*N*-methoxy-*N*-methylbenzamide (**1g**) (91.6 mg, 0.50 mmol). Purification by column chromatography on silica gel (*n*hexane/EtOAc: 5/1) yielded **2g** (65.0 mg, 65%) as a light viscous liquid. **^{1}H-NMR** (400 MHz, $CDCl_3$) δ = 11.79 (s, 1H), 8.02 (dd, J = 9.1, 6.7 Hz, 1H), 6.63 (dd, J = 10.4, 2.6 Hz, 1H), 6.52 (ddd, J = 9.1, 8.1, 2.6 Hz, 1H), 3.62 (s, 3H), 3.36 (s, 3H). **^{13}C-NMR** (100 MHz, $CDCl_3$) δ = 169.0 (C_q), 165.7 (d, $^1J_{C-F}$ = 253.2 Hz, C_q), 163.8 (d, $^3J_{C-F}$ = 13.7 Hz, C_q), 131.7 (d, $^3J_{C-F}$ = 10.8 Hz, CH), 110.6 (d, $^4J_{C-F}$ = 2.8 Hz, C_q), 106.3 (d, $^2J_{C-F}$ = 22.1 Hz, CH), 104.7 (d, $^2J_{C-F}$ = 23.9 Hz, CH), 61.1 (CH_3), 33.8 (CH_3). **^{19}F-NMR** (376 MHz, $CDCl_3$) δ = -103.81– -104.20 (m). **IR** (ATR): 2939, 1621, 1578, 1503, 1264, 1186, 1144, 996 cm^{-1}. **MS** (ESI) *m/z* (relative intensity): 421 (16) $[2M+Na]^+$, 222 (16) $[M+Na]^+$, 200 (100) $[M+H]^+$. **HR-MS** (ESI) *m/z* calc. for $C_9H_{11}FNO_3^+$ $[M+H]^+$ 200.0717, found 200.0719. The analytical data are in accordance with these reported in the literature.[1]

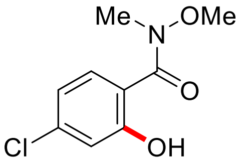

4-Chloro-2-hydroxy-*N*-methoxy-*N*-methylbenzamide (2h): The general procedure

A was followed using 4-chloro-*N*-methoxy-*N*-methylbenzamide (**1h**) (99.8 mg, 0.50 mmol). Purification by column chromatography on silica gel (*n*hexane/EtOAc: 5/1) yielded **2h** (87.1 mg, 81%) as a white solid. **M.p.**: 97-98 ℃. **^{1}H-NMR** (400 MHz, $CDCl_3$) δ = 11.55 (s, 1H), 7.93 (d, *J* = 8.8 Hz, 1H), 6.97 (d, *J* = 2.2 Hz, 1H), 6.79 (dd, *J* = 8.8, 2.2 Hz, 1H), 3.62 (s, 3H), 3.37 (s, 3H). **^{13}C-NMR** (100 MHz, $CDCl_3$) δ = 168.9 (C_q), 162.1 (C_q), 139.3 (C_q), 130.6 (CH), 119.0 (CH), 118.1 (CH), 112.6 (C_q), 61.2 (CH_3), 33.8 (CH_3). **IR** (ATR): 2935, 1624, 1459, 1241, 1201, 1085, 974, 915 cm^{-1}. **MS** (ESI) *m/z* (relative intensity): 238 (28) $[M+Na]^+$ (^{35}Cl), 216 (100) $[M+H]^+$ (^{35}Cl). **HR-MS** (ESI) *m/z* calc. for $C_9H_{11}{}^{35}ClNO_3^+$ $[M+H]^+$ 216.0422, found 216.0424. The analytical data are in accordance with these reported in the literature.[1]

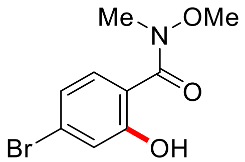

4-Bromo-2-hydroxy-*N*-methoxy-*N*-methylbenzamide (2i): The general procedure **A** was followed using 4-bromo-*N*-methoxy-*N*-methylbenzamide (**1i**) (121 mg, 0.50 mmol). Purification by column chromatography on silica gel (*n*hexane/EtOAc: 5/1) yielded **2i** (107 mg, 82%) as a white solid. The same procedure without the addition of electrolyte (nBu$_4$NPF$_6$) yielded **2i** (74.8 mg, 58%) as a white solid. **M.p.**: 92–95 ℃. **^{1}H-NMR** (300 MHz, $CDCl_3$) δ = 11.21 (s, 1H), 7.89 (d, *J* = 8.7 Hz, 1H), 7.19 (d, *J* = 2.0 Hz, 1H), 6.99 (dd, *J* = 8.7, 2.0 Hz, 1H), 3.66 (s, 3H), 3.41 (s, 3H). **^{13}C-NMR** (100 MHz, $CDCl_3$) δ = 169.0 (C_q), 161.9 (C_q), 130.6 (CH), 127.8 (C_q), 121.9 (CH), 121.2 (CH), 113.0 (C_q), 61.3 (CH_3), 33.8 (CH_3). **IR** (ATR): 2935, 1621, 1481, 1343, 1236, 1075, 861, 590 cm^{-1}. **MS** (ESI) *m/z* (relative intensity): 543 (35) $[2M+Na]^+$ (^{81}Br), 280 (10) $[M+Na]^+$ (^{81}Br), 262 (100) $[M+H]^+$ (^{81}Br). **HR-MS** (ESI) *m/z* calc. for $C_9H_{11}{}^{81}BrNO_3^+$ $[M+H]^+$ 261.9898, found 261.9897.

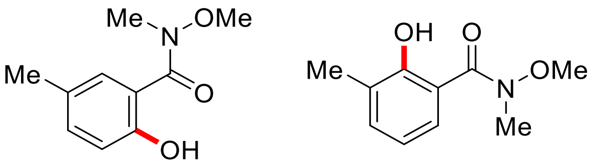

2j **2j'**

2-Hydroxy-*N*-methoxy-*N*,5-dimethylbenzamide (2j): The general procedure **A** was followed using *N*-methoxy-*N*,3-dimethylbenzamide (**1j**) (89.5 mg, 0.50 mmol). Purification by column chromatography on silica gel (*n*hexane/EtOAc: 5/1) yielded **2j** (58.0 mg, 59%) as a colorless liquid, and a isomer **2j'** (2.0 mg, 2%) as a colorless liquid. Characterization data of **2j**: **^{1}H-NMR** (400 MHz, $CDCl_3$) δ = 10.76 (s, 1H), 7.69 (d, J = 2.3, 1H), 7.16 (dd, J = 8.4, 2.3, 1H), 6.86 (d, J = 8.4 Hz, 1H), 3.62 (s, 3H), 3.37 (s, 3H), 2.25 (s, 3H). **^{13}C-NMR** (100 MHz, $CDCl_3$) δ = 170.0 (C_q), 158.5 (C_q), 134.5 (CH), 129.3 (CH), 127.5 (C_q), 117.6 (CH), 114.2 (C_q), 61.1 (CH_3), 34.1 (CH_3), 20.6 (CH_3). **IR** (ATR): 2931, 1631, 1578, 1484, 1431, 1246, 974, 820 cm^{-1}. **MS** (ESI) *m/z* (relative intensity): 413 (2) $[2M+Na]^+$, 218 (100) $[M+Na]^+$, 196 (90) $[M+H]^+$. **HR-MS** (ESI) *m/z* calc. for $C_{10}H_{14}NO_3^+$ $[M+H]^+$ 196.0968, found 196.0971. The analytical data are in accordance with these reported in the literature.[1]

Characterization data of **2j'**: **^{1}H-NMR** (400 MHz, $CDCl_3$) δ = 11.28 (s, 1H), 7.75 (dd, J = 8.2, 1.7 Hz, 1H), 7.22 (dd, J = 7.3, 1.7 Hz, 1H), 6.73 (dd, J = 8.2, 7.3 Hz, 1H), 3.62 (s, 3H), 3.39 (s, 3H), 2.25 (s, 3H). **^{13}C-NMR** (100 MHz, $CDCl_3$) δ = 170.4 (C_q), 159.2 (C_q), 134.6 (CH), 127.0 (CH), 126.8 (C_q), 117.8 (CH), 113.6 (C_q), 61.2 (CH_3), 34.3 (CH_3), 16.0 (CH_3). **IR** (ATR): 2962, 1587, 1474, 1401, 1292, 1193, 1052, 747 cm^{-1}. **MS** (ESI) *m/z* (relative intensity): 218 (100) $[M+Na]^+$, 196 (90) $[M+H]^+$. **HR-MS** (ESI) *m/z* calc. for $C_{10}H_{14}NO_3^+$ $[M+H]^+$ 196.0968, found 196.0968. The analytical data are in accordance with these reported in the literature.[1]

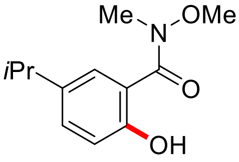

2-Hydroxy-5-isopropyl-*N*-methoxy-*N*-methylbenzamide (2k): The general procedure **A** was followed using 3-isopropyl-*N*-methoxy-*N*-methylbenzamide (**1k**) (104 mg, 0.50 mmol). Purification by column chromatography on silica gel

($n$hexane/EtOAc: 5/1) yielded **2k** (82.7 mg, 74%) as a colourless liquid. **^{1}H-NMR** (400 MHz, $CDCl_3$) δ = 9.96 (s, 1H), 7.74 (d, J = 2.3 Hz, 1H), 7.23 (dd, J = 8.6, 2.3 Hz, 1H), 6.89 (d, J = 8.6 Hz, 1H), 3.63 (s, 3H), 3.39 (s, 3H), 2.82 (h, J = 6.9 Hz, 1H), 1.20 (d, J = 6.9 Hz, 6H). **^{13}C-NMR** (100 MHz, $CDCl_3$) δ = 170.1 (C_q), 158.2 (C_q), 139.0 (C_q), 132.1 (CH), 126.8 (CH), 117.6 (CH), 114.2 (C_q), 61.2 (CH_3), 34.3 (CH_3), 33.3 (CH), 24.1 (CH_3). **IR** (ATR): 2959, 1630, 1579, 1485, 1460, 1350, 1250, 976 cm^{-1}. **MS** (ESI) m/z (relative intensity): 246 (100) $[M+Na]^+$, 224 (80) $[M+H]^+$. **HR-MS** (ESI) m/z calc. for $C_{12}H_{18}NO_3^+$ $[M+H]^+$ 224.1281, found 224.1281.

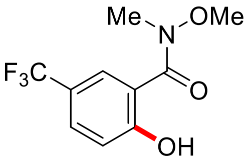

2-Hydroxy-*N*-methoxy-*N*-methyl-5-(trifluoromethyl)benzamide (2l): The general procedure **A** was followed using *N*-methoxy-*N*-methyl-3-(trifluoromethyl)benzamide (**1l**) (117 mg, 0.50 mmol). Purification by column chromatography on silica gel ($n$hexane/EtOAc: 5/1) yielded **2l** (56.0 mg, 45%) as a yellow oil. **^{1}H-NMR** (400 MHz, $CDCl_3$) δ = 11.83 (s, 1H), 8.37 (d, J = 2.3 Hz, 1H), 7.63 (dd, J = 8.8, 2.3 Hz, 1H), 7.12–7.06 (m, 1H), 3.70 (s, 3H), 3.45 (s, 3H). **^{13}C-NMR** (100 MHz, $CDCl_3$) δ = 168.5 (C_q), 163.9 (C_q), 130.5 (q, $^{3}J_{C-F}$ = 3.4 Hz, CH), 127.5 (q, $^{3}J_{C-F}$ = 3.4 Hz, CH), 124.1 (q, $^{1}J_{C-F}$ = 271.1 Hz, C_q), 120.8 (q, $^{2}J_{C-F}$ = 33.0 Hz, C_q), 118.7 (CH), 113.8 (C_q), 61.4 (CH_3), 33.8 (CH_3). **^{19}F-NMR** (376 MHz, $CDCl_3$) δ = –61.74 (s). **IR** (ATR): 2956, 1747, 1460, 1374, 1237, 1048, 742, 385 cm^{-1}. **MS** (ESI) m/z (relative intensity): 271 (80) $[M+Na]^+$, 250 (100) $[M+H]^+$. **HR-MS** (ESI) m/z calc. for $C_{10}H_{11}F_3NO_3^+$ $[M+H]^+$ 250.0684, found 250.0686.

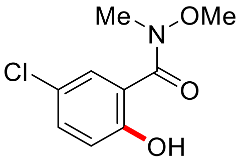

5-Chloro-2-hydroxy-*N*-methoxy-*N*-methylbenzamide (2m): The general procedure **A** was followed using 3-chloro-*N*-methoxy-*N*-methylbenzamide (**1m**) (99.8 mg,

0.50 mmol). Purification by column chromatography on silica gel (*n*hexane/EtOAc: 5/1) yielded **2m** (90.3 mg, 84%) as a white solid. **M.p.**: 74–77 °C. **^{1}H-NMR** (400 MHz, $CDCl_3$) δ = 11.21 (s, 1H), 8.00 (d, *J* = 2.6 Hz, 1H), 7.5 (dd, *J* = 8.9, 2.6 Hz, 1H), 6.95 (d, *J* = 8.9 Hz, 1H), 3.69 (s, 3H), 3.42 (s, 3H). **^{13}C-NMR** (100 MHz, $CDCl_3$) δ = 168.5 (C_q), 159.7 (C_q), 133.7 (CH), 129.0 (CH), 123.3 (C_q), 119.6 (CH), 115.1 (C_q), 61.4 (CH_3), 33.8 (CH_3). **IR** (ATR): 2958, 2253, 1630, 1586, 1469, 1249, 903, 722 cm^{-1}. **MS** (ESI) *m/z* (relative intensity): 453 (15) $[2M+Na]^+$ (^{35}Cl), 238 (30) $[M+Na]^+$ (^{35}Cl), 216 (100) $[M+H]^+$ (^{35}Cl). **HR-MS** (ESI) *m/z* calc. for $C_9H_{11}{}^{35}ClNO_3^+$ $[M+H]^+$ 216.0415, found 216.0422.

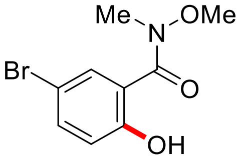

5-Bromo-2-hydroxy-*N*-methoxy-*N*-methylbenzamide (2n): The general procedure **A** was followed using 3-bromo-*N*-methoxy-*N*-methylbenzamide (**1n**) (121 mg, 0.50 mmol). Purification by column chromatography on silica gel (*n*hexane/EtOAc: 5/1) yielded **2n** (97.4 mg, 75%) as a yellow oil. **^{1}H-NMR** (400 MHz, $CDCl_3$) δ = 11.19 (s, 1H), 8.10 (d, *J* = 2.5 Hz, 1H), 7.43 (dd, *J* = 8.9, 2.5 Hz, 1H), 6.86 (d, *J* = 8.9 Hz, 1H), 3.65 (s, 3H), 3.38 (s, 3H). **^{13}C-NMR** (100 MHz, $CDCl_3$) δ = 168.4 (C_q), 160.1 (C_q), 136.5 (CH), 131.9 (CH), 119.9 (CH), 115.7 (C_q), 110.2 (C_q), 61.4 (CH_3), 33.8 (CH_3). **IR** (ATR): 2985, 1737, 1447, 1372, 1233, 1043, 847, 608 cm^{-1}. **MS** (ESI) *m/z* (relative intensity): 543 (20) $[2M+Na]^+$ (^{79}Br), 260 (100) $[M+H]^+$ (^{79}Br). **HR-MS** (ESI) *m/z* calc. for $C_9H_{11}{}^{79}BrNO_3^+$ $[M+H]^+$ 259.9914, found 259.9917.

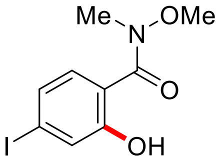

4-Iodo-2-hydroxy-*N*-methoxy-*N*-methylbenzamide (2o): The general procedure **A** was followed using 4-iodo-*N*-methoxy-*N*-methylbenzamide (**1o**) (146 mg, 0.50 mmol).

Purification by column chromatography on silica gel (*n*hexane/EtOAc: 5/1) yielded **2o** (99.7 mg, 65%) as a yellow liquid. **^{1}H-NMR** (400 MHz, $CDCl_3$) δ = 10.68 (s, 1 H), 7.65 (d, J = 8.6 Hz, 1H), 7.36 (d, J = 1.8 Hz, 1H), 7.16 (dd, J = 8.6, 1.8 Hz, 1H), 3.61 (s, 3H), 3.36 (s, 3H). **^{13}C-NMR** (100 MHz, $CDCl_3$) δ = 169.1 (C_q), 161.2 (C_q), 130.4 (CH), 127.8 (CH), 127.2 (CH), 113.6 (C_q), 100.3 (C_q), 61.3 (CH_3), 33.8 (CH_3). **IR** (ATR): 2934, 1618, 1476, 1341, 1233, 972, 809, 586 cm^{-1}. **MS** (ESI) *m/z* (relative intensity): 634 (16) $[2M+Na]^+$, 330 (16) $[M+Na]^+$, 308 (100) $[M+H]^+$. **HR-MS** (ESI) *m/z* calc. for $C_9H_{11}INO_3^+$ $[M+H]^+$ 307.9780, found 307.9778. The analytical data are in accordance with these reported in the literature.[1]

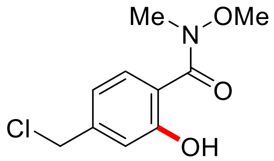

4-(Chloromethyl)-2-hydroxy-*N*-methoxy-*N*-methylbenzamide (2p): The general procedure **A** was followed using 4-(chloromethyl)-*N*-methoxy-*N*-methylbenzamide (**1p**) (107 mg, 0.50 mmol). Purification by column chromatography on silica gel (*n*hexane/EtOAc: 5/1) yielded **2p** (88.2 mg, 77%) as an orange oil. **^{1}H-NMR** (400 MHz, $CDCl_3$) δ = 11.36 (s, 1H), 7.99 (d, J = 8.4 Hz, 1H), 7.02 (d, J = 1.8 Hz, 1H), 6.89 (dd, J = 8.4, 1.8 Hz, 1H), 4.54 (s, 2H), 3.67 (s, 3H), 3.42 (s, 3H). **^{13}C-NMR** (100 MHz, $CDCl_3$) δ = 169.3 (C_q), 161.3 (C_q), 143.2 (C_q), 130.1 (CH), 118.5 (CH), 117.8 (CH), 114.0 (C_q), 61.3 (CH_3), 45.3 (CH_2), 33.9 (CH_3). **IR** (ATR): 2985, 1636, 1589, 1265, 904, 724, 650, 534 cm^{-1}. **MS** (ESI) *m/z* (relative intensity): 252 (20) $[M+Na]^+$ (^{35}Cl), 230 (100) $[M+H]^+$ (^{35}Cl). **HR-MS** (ESI) *m/z* calc. for $C_{10}H_{13}{}^{35}ClNO_3^+$ $[M+H]^+$ 230.0585, found 230.0578. The analytical data are in accordance with these reported in the literature.[1]

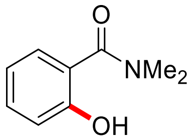

2-Hydroxy-*N*,*N*-dimethylbenzamide (2q): The general procedure **A** was followed

using *N*,*N*-dimethylbenzamide (**1q**) (74.6 mg, 0.50 mmol). Purification by column chromatography on silica gel (*n*hexane/EtOAc: 5/1→3/1) yielded **2q** (51.0 mg, 62%) as a white solid. **M.p.**: 164–165 ℃. **^{1}H-NMR** (400 MHz, $CDCl_3$) δ = 9.92 (s, 1H), 7.44–7.21 (m, 2H), 6.95 (dd, J = 8.7, 1.2 Hz, 1H), 6.86–6.75 (m, 1H), 3.12 (s, 6H). **^{13}C-NMR** (100 MHz, $CDCl_3$) δ = 171.8 (C_q), 159.0 (C_q), 132.4 (CH), 128.5 (CH), 118.2 (CH), 117.8 (CH), 117.2 (C_q), 38.3 (CH_3). **IR** (ATR): 3145, 2935, 1595, 1494, 1452, 1262, 1199, 754 cm^{-1}. **MS** (ESI) *m/z* (relative intensity): 353 (8) $[2M+Na]^+$, 188 (40) $[M+Na]^+$, 166 (100) $[M+H]^+$. **HR-MS** (ESI) *m/z* calc. for $C_9H_{12}NO_2^+$ $[M+H]^+$ 166.0863, found 166.0863. The analytical data are in accordance with these reported in the literature.[2]

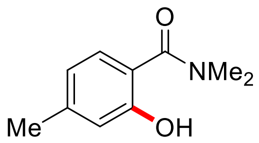

2-Hydroxy-*N*,*N*,4-trimethylbenzamide (2r): The general procedure **A** was followed using *N*,*N*,4-trimethylbenzamide (**1r**) (81.5 mg, 0.50 mmol). Purification by column chromatography on silica gel (*n*hexane/EtOAc: 5/1→3/1) yielded **2r** (75.2 mg, 84%) as a white solid. **M.p.**: 118–120 ℃. **^{1}H-NMR** (300 MHz, $CDCl_3$) δ = 9.05 (s, 1H), 7.22 (d, J = 8.0 Hz, 1H), 6.82 (d, J = 1.6 Hz, 1H), 6.67 (dd, J = 8.0, 1.6 Hz, 1H), 3.16 (s, 6H), 2.33 (s, 3H). **^{13}C-NMR** (100 MHz, $CDCl_3$) δ = 172.1 (C_q), 159.1 (C_q), 143.4 (C_q), 128.4 (CH), 119.2 (CH), 118.1 (CH), 114.2 (C_q), 38.4 (CH_3), 21.5 (CH_3). **IR** (ATR): 2935, 1595, 1494, 1452, 1400, 1262, 1199, 754 cm^{-1}. **MS** (ESI) *m/z* (relative intensity): 202 (100) $[M+Na]^+$, 180 (85) $[M+H]^+$. **HR-MS** (ESI) *m/z* calc. for $C_{10}H_{14}NO_2^+$ $[M+H]^+$ 180.1017, found 180.1019. The analytical data are in accordance with these reported in the literature.[3]

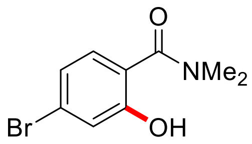

4-Bromo-2-hydroxy-*N*,*N*-dimethylbenzamide (2s): The general procedure **A** was

followed using 4-bromo-*N*,*N*-dimethylbenzamide (**1s**) (114 mg, 0.50 mmol). Purification by column chromatography on silica gel (*n*hexane/EtOAc: 5/1→3/1) yielded **2s** (101 mg, 83%) as a white solid. **M.p.**: 106–107 °C. **^{1}H-NMR** (400 MHz, $CDCl_3$) δ = 10.16 (s, 1H), 7.20–7.06 (m, 2H), 6.95 (dd, J = 8.3, 1.9 Hz, 1H), 3.09 (s, 6H). **^{13}C-NMR** (100 MHz, $CDCl_3$) δ = 171.1 (C_q), 159.6 (C_q), 129.4 (CH), 126.0 (C_q), 121.6 (CH), 121.0 (CH), 116.4 (C_q), 38.2 (CH_3). **IR** (ATR): 3070, 2969, 1577, 1445, 1251, 1086, 876, 755 cm^{-1}. **MS** (ESI) *m/z* (relative intensity): 268 (98) $[M+Na]^+$ (^{81}Br), 246 (100) $[M+H]^+$ (^{81}Br). **HR-MS** (ESI) *m/z* calc. for $C_9H_{11}{}^{81}BrNO_2^+$ $[M+H]^+$ 243.9968, found 243.9968.

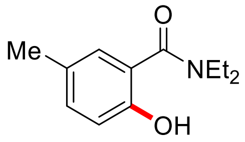

***N*,*N*-Diethyl-2-hydroxy-5-methylbenzamide (2t):** The general procedure **A** was followed using *N*,*N*-diethyl-3-methylbenzamide (**1t**) (95.6 mg, 0.50 mmol). Purification by column chromatography on silica gel (*n*hexane/EtOAc: 5/1→3/1) yielded **2t** (66.3 mg, 64%) as a white solid. **M.p.**: 116–119 °C. **^{1}H-NMR** (400 MHz, $CDCl_3$) δ = 9.19 (s, 1H), 7.13 (dd, J = 8.3, 2.1 Hz, 1H), 7.07 (d, J = 2.1 Hz, 1H), 6.91 (d, J = 8.3 Hz, 1H), 3.54 (q, J = 7.1 Hz, 4H), 2.30 (s, 3H), 1.29 (t, J = 7.1 Hz, 6H). **^{13}C-NMR** (100 MHz, $CDCl_3$) δ = 171.5 (C_q), 156.1 (C_q), 132.9 (CH), 127.6 (C_q), 127.4 (CH), 118.2 (C_q), 117.7 (CH), 42.1 (CH_2), 20.6 (CH_3), 13.4 (CH_3). **IR** (ATR): 3160, 2973, 1586, 1486, 1440, 1286, 1244, 820 cm^{-1}. **MS** (ESI) *m/z* (relative intensity): 437 (10) $[M+2Na]^+$, 230 (108) $[M+Na]^+$, 208 (80) $[M+H]^+$. **HR-MS** (ESI) *m/z* calc. for $C_{12}H_{18}NO_2^+$ $[M+H]^+$ 208,1337, found 208.1332. The analytical data are in accordance with these reported in the literature.[4]

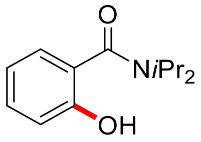

2-Hydroxy-*N*,*N*-diisopropylbenzamide (2u): The general procedure **A** was followed

using *N*,*N*-diisopropylbenzamide (**1u**) (103 mg, 0.50 mmol). Purification by column chromatography on silica gel (*n*hexane/EtOAc: 5/1→3/1) yielded **2u** (61.0 mg, 55%) as a white solid. **M.p.**: 141–142 ℃. **^{1}H-NMR** (400 MHz, $CDCl_3$) δ = 9.18 (s, 1H), 7.21 (m, 1H), 7.12 (dd, J = 7.7, 1.7 Hz, 1H), 6.91 (dd, J = 8.2, 1.1 Hz, 1H), 6.79 (m, 1H), 3.88 (s, 2H), 1.35 (d, J = 6.8 Hz, 12H). **^{13}C-NMR** (100 MHz, $CDCl_3$) δ = 170.9 (C_q), 157.4 (C_q), 131.2 (CH), 126.6 (CH), 120.9 (C_q), 118.5 (CH), 117.7 (CH), 48.9 (CH), 20.9 (CH_3). **IR** (ATR): 3153, 2967, 1711, 1584, 1445, 1347, 1209, 850 cm^{-1}. **MS** (ESI) *m/z* (relative intensity): 465 (30) $[M+2Na]^+$, 244 (40) $[M+Na]^+$, 222 (100) $[M+H]^+$. **HR-MS** (ESI) *m/z* calc. for $C_{13}H_{20}NO_2^+$ $[M+H]^+$ 222.1489, found 222.1492. The analytical data are in accordance with these reported in the literature.[2]

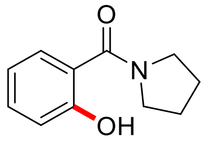

(2-Hydroxyphenyl)(pyrrolidin-1-yl)methanone (2v): The general procedure **A** was followed using phenyl(pyrrolidin-1-yl)methanone (**1v**) (87.6 mg, 0.50 mmol). Purification by column chromatography on silica gel (*n*hexane/EtOAc: 5/1→3/1) yielded **2v** (87.0 mg, 91%) as a white solid. The same procedure without the addition of electrolyte (*n*Bu_4NPF_6) yielded **2v** (58.3 mg, 61%) as a white solid. **M.p.**: 132–133 ℃. **^{1}H-NMR** (400 MHz, $CDCl_3$) δ = 11.21 (s, 1H), 7.40 (dd, J = 7.9, 1.7 Hz, 1H), 7.32–7.21 (m, 1H), 6.91 (dd, J = 8.3, 1.2 Hz, 1H), 6.77 (m, 1H), 3.72–3.51 (m, 4H), 1.88 (m, 4H). **^{13}C-NMR** (100 MHz, $CDCl_3$) δ = 169.9 (C_q), 160.0 (C_q), 132.6 (CH), 128.1 (CH), 117.9 (CH), 117.5 (CH), 117.2 (C_q), 50.4 (CH_2), 47.2 (CH_2), 26.4 (CH_2), 24.0 (CH_2). **IR** (ATR): 2972, 2877, 1579, 1454, 1360, 1249, 1158, 756 cm^{-1}. **MS** (ESI) *m/z* (relative intensity): 214 (100) $[M+Na]^+$, 192 (85) $[M+H]^+$. **HR-MS** (ESI) *m/z* calc. for $C_{11}H_{14}NO_2^+$ $[M+H]^+$ 192.1019, found 192.1020. The analytical data are in accordance with these reported in the literature.[2]

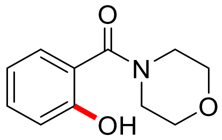

(2-Hydroxyphenyl)(morpholino)methanone (2w): The general procedure **A** was followed using morpholino(phenyl)methanone (**1w**) (95.6 mg, 0.50 mmol). Purification by column chromatography on silica gel (*n*hexane/EtOAc: 5/1→3/1) yielded **2w** (62.1 mg, 60%) as a white solid. **M.p.**: 174–176 ℃. **^{1}H-NMR** (400 MHz, $CDCl_3$) δ = 9.49 (s, 1H), 7.31 (ddd, J = 8.3, 7.3, 1.6 Hz, 1H), 7.20 (dd, J = 7.8, 1.6 Hz, 1H), 6.98 (dd, J = 8.3, 1.2 Hz, 1H), 6.89–6.80 (m, 1H), 3.71 (m, 8H). **^{13}C-NMR** (100 MHz, $CDCl_3$) δ = 170.8 (C_q), 159.0 (C_q), 132.8 (CH), 128.2 (CH), 118.6 (CH), 118.1 (CH), 116.6 (C_q), 66.8 (CH_2), 46.2 (CH_2). **IR** (ATR): 3152, 2967, 1592, 1447, 1360, 1225, 1020, 755 cm^{-1}. **MS** (ESI) *m/z* (relative intensity): 437 (5) $[M+2Na]^+$, 230 (50) $[M+Na]^+$, 208 (100) $[M+H]^+$. **HR-MS** (ESI) *m/z* calc. for $C_{11}H_{14}NO_3^+$ $[M+H]^+$ 208.0968, found 208.0973. The analytical data are in accordance with these reported in the literature.[2]

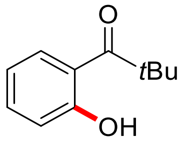

1-(2-Hydroxyphenyl)ethan-1-one (5a): The general procedure **B** was followed using acetophenone (**4a**) (81.1 mg, 0.50 mmol). Purification by column chromatography on silica gel (*n*hexane/EtOAc: 50/1) yielded **5a** (75.7 mg, 85%) as a colorless liquid. **^{1}H-NMR** (400 MHz, $CDCl_3$) δ = 12.67 (s, 1H), 8.00 (dd, J = 8.4, 1.6 Hz, 1H), 7.40 (ddd, J = 8.6, 7.1, 1.6 Hz, 1H), 6.99 (dd, J = 8.6, 1.3 Hz, 1H), 6.83 (ddd, J = 8.4, 7.1, 1.3 Hz, 1H), 1.44 (s, 9H). **^{13}C-NMR** (100 MHz, $CDCl_3$) δ = 212.1 (C_q), 163.6 (C_q), 135.3 (CH), 130.8 (CH), 119.3 (CH), 117.7 (CH), 117.5 (C_q), 44.6 (C_q), 28.7 (CH_3). **IR** (ATR): 2974, 1627, 1477, 1443, 1298, 1249, 960, 753 cm^{-1}. **MS** (ESI) *m/z* (relative intensity): 381 (100) $[2M+Na]^+$, 179 (40) $[M+H]^+$. **HR-MS** (ESI) *m/z* calc. for $C_{11}H_{15}O_2^+$ $[M+H]^+$ 179.1067, found 179.1067. The analytical data are in accordance with these reported in the literature.[5]

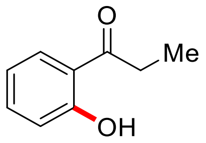

1-(2-Hydroxyphenyl)propan-1-one (5b): The general procedure **B** was followed using propiophenone (**4b**) (67.0 mg, 0.50 mmol). Purification by column chromatography on silica gel (*n*hexane/EtOAc: 80/1) yielded **5b** (45.1 mg, 60%) as a colorless liquid. **^{1}H-NMR** (400 MHz, $CDCl_3$) δ = 12.33 (s, 1H), 7.74 (dd, J = 8.0, 1.6 Hz, 1H), 7.43 (ddd, J = 8.4, 7.2, 1.6 Hz, 1H), 6.95 (dd, J = 8.4, 1.2 Hz, 1H), 6.87 (ddd, J = 8.0, 7.2, 1.2 Hz, 1H), 3.02 (q, J = 7.3 Hz, 2H), 1.22 (t, J = 7.3 Hz, 3H). **^{13}C-NMR** (100 MHz, $CDCl_3$) δ = 207.1 (C_q), 162.3 (C_q), 136.1 (CH), 129.8 (CH), 119.2 (C_q), 118.8 (CH), 118.4 (CH), 31.5 (CH_2), 8.16 (CH_3). **IR** (ATR): 2984, 1641, 1488, 1265, 1206, 906, 272, 649 cm^{-1}. **MS** (ESI) *m/z* (relative intensity): 323 (50) $[2M+Na]^+$, 151 (100) $[M+H]^+$. **HR-MS** (ESI) *m/z* calc. for $C_9H_{11}O_2^+$ $[M+H]^+$ 151.0753, found 151.0754. The analytical data are in accordance with these reported in the literature.[6]

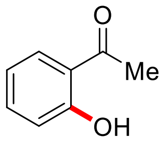

1-(2-Hydroxyphenyl)ethan-1-one (5c): The general procedure **B** was followed using acetophenone (**4c**) (60.0 mg, 0.50 mmol). Purification by column chromatography on silica gel (*n*hexane/EtOAc: 50/1) yielded **5c** (45.0 mg, 66%) as a colorless liquid. **^{1}H-NMR** (400 MHz, $CDCl_3$) δ = 12.24 (s, 1H), 7.72 (dd, J = 8.1, 1.7 Hz, 1H), 7.45 (ddd, J = 8.7, 7.2, 1.7 Hz, 1H), 6.96 (dd, J = 8.7, 1.2 Hz, 1H), 6.88 (ddd, J = 8.1, 7.2, 1.2 Hz, 1H), 2.62 (s, 3H). **^{13}C-NMR** (100 MHz, $CDCl_3$) δ = 204.5 (C_q), 162.4 (C_q), 136.4 (CH), 130.7 (CH), 119.7 (C_q), 118.9 (CH), 118.4 (CH), 26.6 (CH_3). **IR** (ATR): 3054, 1637, 1615, 1446, 1243, 1218, 752, 619 cm^{-1}. **MS** (ESI) *m/z* (relative intensity): 283 (8), 157 (7), 135 (100) $[M-H]^-$, 121 (5). **HR-MS** (ESI) *m/z* calc. for $C_8H_8O_2$ $[M-H]^-$ 135.0452, found 135.0443. The analytical data are in accordance with these

reported in the literature.[6]

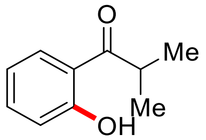

1-(2-Hydroxyphenyl)-2-methylpropan-1-one (5d): The general procedure **B** was followed using 2-methyl-1-phenylpropan-1-one (**4d**) (74.0 mg, 0.50 mmol). Purification by column chromatography on silica gel (*n*hexane/EtOAc: 100/1) yielded **5d** (54.2 mg, 66%) as a yellow oil. ^{1}**H-NMR** (300 MHz, $CDCl_3$) δ = 12.55 (s, 1H), 7.82 (dd, J = 8.1, 1.5 Hz, 1H), 7.56–7.43 (m, 1H), 7.02 (d, J = 8.4 Hz, 1H), 6.93 (dd, J = 8.1, 7.1 Hz, 1H), 3.63 (h, J = 6.9 Hz, 1H), 1.28 (d, J = 6.8 Hz, 6H). ^{13}**C-NMR** (100 MHz, $CDCl_3$) δ = 210.8 (C_q), 163.1 (C_q), 136.2 (CH), 129.8 (CH), 118.8 (CH), 118.7 (CH), 118.2 (C_q), 34.9 (CH), 19.3 (CH_3). **IR** (ATR): 2975, 1636, 1446, 1273, 1208, 1151, 982, 754 cm^{-1}. **MS** (ESI) *m/z* (relative intensity): 351 (50) $[2M+Na]^+$, 165 (100) $[M+H]^+$. **HR-MS** (ESI) *m/z* calc. for $C_{10}H_{13}O_2^+$ $[M+H]^+$ 165.0904, found 165.0910. The analytical data are in accordance with these reported in the literature.[5]

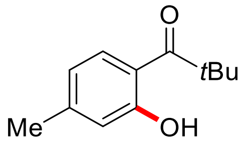

1-(2-Hydroxy-4-methylphenyl)-2,2-dimethylpropan-1-one (5e): The general procedure **B** was followed using 2,2-dimethyl-1-(*p*-tolyl)propan-1-one (**4e**) (88.0 mg, 0.50 mmol). Purification by column chromatography on silica gel (*n*hexane/EtOAc: 50/1) yielded **5e** (49.2 mg, 51%) as a colorless liquid. ^{1}**H-NMR** (400 MHz, $CDCl_3$) δ = 12.77 (s, 1H), 7.88 (d, J = 8.4 Hz, 1H), 6.79 (d, J = 1.8 Hz, 1H), 6.64 (dd, J = 8.4, 1.8 Hz, 1H), 2.31 (s, 3H), 1.42 (s, 9H). ^{13}**C-NMR** (100 MHz, $CDCl_3$) δ = 211.5 (C_q), 163.9 (C_q), 146.7 (C_q), 130.7 (CH), 119.2 (CH), 119.0 (CH), 115.1 (C_q), 44.4 (C_q), 28.7 (CH_3), 21.7 (CH_3). **IR** (ATR): 2978, 1632, 1301, 1190, 1136, 973, 801, 403 cm^{-1}. **MS** (ESI) *m/z* (relative intensity): 407 (30) $[2M+Na]^+$, 215 (20) $[M+Na]^+$, 193 (100) $[M+H]^+$. **HR-MS** (ESI) *m/z* calc. for $C_{12}H_{17}O_2^+$ $[M+H]^+$

193.1225, found 193.1223. The analytical data are in accordance with these reported in the literature.[5]

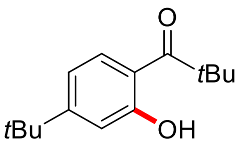

1-(2-Hydroxyphenyl)ethan-1-one (5f): The general procedure **B** was followed using 1-[4-(*tert*-butyl)phenyl]-2,2-dimethylpropan-1-one (**4f**) (109 mg, 0.50 mmol). Purification by column chromatography on silica gel (*n*hexane/EtOAc: 50/1) yielded **5f** (80.0 mg, 68%) as a colorless liquid. **^{1}H-NMR** (400 MHz, $CDCl_3$) δ = 12.77 (s, 1H), 7.93 (d, J = 8.7 Hz, 1H), 6.99 (d, J = 2.1 Hz, 1H), 6.86 (dd, J = 8.7, 2.1 Hz, 1H), 1.43 (s, 9H), 1.29 (s, 9H). **^{13}C-NMR** (100 MHz, $CDCl_3$) δ = 211.4 (C_q), 163.8 (C_q), 159.6 (C_q), 130.6 (CH), 115.8 (CH), 115.4 (CH), 115.0 (C_q), 44.4 (C_q), 35.1 (C_q), 30.7 (CH_3), 28.7 (CH_3). **IR** (ATR): 2964, 1624, 1558, 1300, 1233, 1185, 968, 808 cm^{-1}. **MS** (ESI) *m/z* (relative intensity): 257 (50) $[M+Na]^+$, 235 (100) $[M+H]^+$. **HR-MS** (ESI) *m/z* calc. for $C_{15}H_{23}O_2^+$ $[M+H]^+$ 235.1693, found 235.1693. The analytical data are in accordance with these reported in the literature.[7]

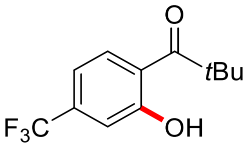

1-[2-Hydroxy-4-(trifluoromethyl)phenyl]-2,2-dimethylpropan-1-one (5g): The general procedure **B** was followed using 2,2-dimethyl-1-[4-(trifluoromethyl)phenyl]propan-1-one (**4g**) (115 mg, 0.50 mmol). Purification by column chromatography on silica gel (*n*hexane/EtOAc: 50/1) yielded **5f** (79.9 mg, 65%) as a light yellow liquid. **^{1}H-NMR** (400 MHz, $CDCl_3$) δ = 12.62 (s, 1H), 8.51–8.08 (m, 1H), 7.25 (d, J = 2.0 Hz, 1H), 7.11–7.03 (m, 1H), 1.44 (s, 9H). **^{13}C-NMR** (100 MHz, $CDCl_3$) δ = 211.9 (C_q), 163.5 (C_q), 136.2 (q, $^{2}J_{C–F}$ = 33.0 Hz, C_q), 131.5 (CH), 123.0 (q, $^{1}J_{C–F}$ = 273.1 Hz, C_q), 119.5 (C_q), 116.7 (q, $^{3}J_{C–F}$ = 4.0 Hz, CH), 114.1 (q, $^{3}J_{C–F}$ = 3.6 Hz, CH), 44.9 (C_q), 28.5 (CH_3). **^{19}F-NMR** (376 MHz,

$CDCl_3$) δ = –64.07 (s). **IR** (ATR): 2978, 1642, 1417, 1330, 1235, 1164, 1126, 964 cm^{-1}. **MS** (ESI) *m/z* (relative intensity): 245 (100) $[M–H]^-$. **HR-MS** (ESI) *m/z* calc. for $C_{12}H_{12}F_3O_2^-$ $[M–H]^-$ 245.0795, found 245.0796. The analytical data are in accordance with these reported in the literature.[5]

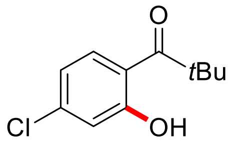

1-(4-Chloro-2-hydroxyphenyl)-2,2-dimethylpropan-1-one (5h): The general procedure **B** was followed using 1-(4-chlorophenyl)-2,2-dimethylpropan-1-one (**4h**) (98.4 mg, 0.50 mmol). Purification by column chromatography on silica gel (*n*hexane/EtOAc: 50/1) yielded **5h** (65.0 mg, 61%) as a colorless liquid. **^{1}H-NMR** (400 MHz, $CDCl_3$) δ = 12.83 (s, 1H), 7.92 (d, *J* = 8.8 Hz, 1H), 6.99 (d, *J* = 2.2 Hz, 1H), 6.80 (dd, *J* = 8.8, 2.2 Hz, 1H), 1.41 (s, 9H). **^{13}C-NMR** (100 MHz, $CDCl_3$) δ = 211.4 (C_q), 164.5 (C_q), 140.9 (C_q), 131.8 (CH), 119.2 (CH), 118.4 (CH), 115.9 (C_q), 44.6 (C_q), 28.6 (CH_3). **IR** (ATR): 2970, 1625, 1561, 1487, 1343, 1179, 960, 799 cm^{-1}. **MS** (ESI) *m/z* (relative intensity): 279 (10), 245 (8), 211 (100) $[M–H]^-$ (^{35}Cl). **HR-MS** (ESI) *m/z* calc. for $C_{11}H_{12}{}^{35}ClO_2^-$ $[M–H]^-$ 211.0531, found 211.0525. The analytical data are in accordance with these reported in the literature.[5]

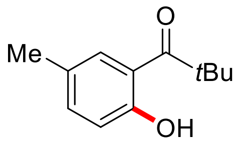

1-(2-Hydroxy-5-methylphenyl)-2,2-dimethylpropan-1-one (5i): The general procedure **B** was followed using 2,2-dimethyl-1-(m-tolyl)propan-1-one (**4i**) (88.1 mg, 0.50 mmol) at a constant current of 3 mA. Purification by column chromatography on silica gel (*n*hexane/EtOAc: 50/1) yielded **5h** (62.0 mg, 64%) as a colorless liquid. **^{1}H-NMR** (400 MHz, $CDCl_3$) δ = 12.48 (s, 1H), 7.77 (s, 1H), 7.21 (d, *J* = 8.5 Hz, 1H), 6.89 (d, *J* = 8.5 Hz, 1H), 2.29 (s, 3H), 1.43 (s, 9H). **^{13}C-NMR** (100 MHz, $CDCl_3$) δ = 211.9 (C_q), 161.4 (C_q), 136.3 (CH), 130.5 (CH), 126.6 (C_q), 119.0 (CH),

117.2 (C_q), 44. 6 (C_q), 28.8 (CH_3), 20.8 (CH_3). **IR** (ATR): 2973, 1632, 1477, 1292, 1251, 1205, 971, 779 cm^{-1}. **MS** (ESI) *m/z* (relative intensity): 407 (50) $[2M+Na]^+$, 193 (100) $[M+H]^+$. **HR-MS** (ESI) *m/z* calc. for $C_{12}H_{17}O_2^+$ $[M+H]^+$ 193.1223, found 193.1222. The analytical data are in accordance with these reported in the literature.[5]

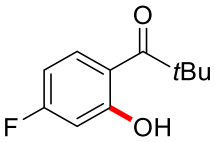

1-(4-Fluoro-2-hydroxyphenyl)-2,2-dimethylpropan-1-one (5j): The general procedure **B** was followed using 1-(4-fluorophenyl)-2,2-dimethylpropan-1-one (**4j**) (90.1 mg, 0.50 mmol). Purification by column chromatography on silica gel (*n*hexane/EtOAc: 50/1) yielded **5j** (63.9 mg, 65%) as a colorless liquid. **^{1}H-NMR** (400 MHz, $CDCl_3$) δ = 13.06 (d, J = 1.5 Hz, 1H), 8.01 (dd, J = 9.2, 6.5 Hz, 1H), 6.65 (dd, J = 10.4, 2.7 Hz, 1H), 6.54 (ddd, J = 9.2, 7.9, 2.7 Hz, 1H), 1.41 (s, 9H). **^{13}C-NMR** (100 MHz, $CDCl_3$) δ = 211.0 (C_q), 166.5 (d, $^3J_{C–F}$ = 14.1 Hz, C_q), 166.4 (d, $^1J_{C–F}$ = 256.2 Hz, C_q), 133.2 (d, $^3J_{C–F}$ = 11.5 Hz, CH), 114.4 (d, $^4J_{C–F}$ = 2.6 Hz, C_q), 106.0 (d, $^2J_{C–F}$ = 22.5 Hz, CH), 105.5 (d, $^2J_{C–F}$ = 23.1 Hz, CH), 44.5 (C_q), 28.7 (CH_3). **^{19}F-NMR** (376 MHz, $CDCl_3$) δ = –100.81 (ddd, J = 10.4, 7.9, 6.5 Hz). **IR** (ATR): 2975, 1632, 1693, 1259, 1188, 1148, 986, 576 cm^{-1}. **MS** (ESI) *m/z* (relative intensity): 195 (100) $[M–H]^-$. **HR-MS** (ESI) *m/z* calc. for $C_{11}H_{12}FO_2^-$ $[M–H]^-$ 195.0827, found 195.0828. The analytical data are in accordance with these reported in the literature.[3]

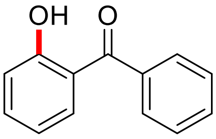

(2-Hydroxyphenyl)(phenyl)methanone (5k): The general procedure **B** was followed using benzophenone (**4k**) (91.1 mg, 0.50 mmol). The reaction time was 8 h. Purification by column chromatography on silica gel (*n*hexane/EtOAc: 50/1) yielded **5k** (56.5 mg, 57%) as a light yellow liquid. **^{1}H-NMR** (400 MHz, $CDCl_3$) δ = 12.02 (s, 1H), 7.70–7.64 (m, 2H), 7.62–7.55 (m, 2H), 7.53–7.45 (m, 3H), 7.13–7.04 (m, 1H),

6.86 (ddd, J = 8.2, 7.2, 1.2 Hz, 1H). **^{13}C-NMR** (100 MHz, $CDCl_3$) δ = 201.6 (C_q), 163.2 (C_q), 137.9 (C_q), 136.3 (CH), 133.6 (CH), 131.9 (CH), 129.1 (CH), 128.3 (CH), 119.1 (C_q), 118.6 (CH), 118.4 (CH). **IR** (ATR): 3058, 1625, 1483, 1445, 1330, 1243, 1222, 775 cm^{-1}. **MS** (ESI) *m/z* (relative intensity): 419 (20) $[2M+Na]^+$, 199 (100) $[M+H]^+$. **HR-MS** (ESI) *m/z* calc. for $C_{13}H_{11}O_2^+$ $[M+H]^+$ 199.0754, found 199.0753. The analytical data are in accordance with these reported in the literature.[5]

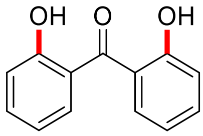

(2-Hydroxyphenyl)(phenyl)methanone (5k'): The general procedure **B** was followed using benzophenone (**4k'**) (91.1 mg, 0.50 mmol). The reaction time is 24 h. Purification by column chromatography on silica gel (*n*hexane/EtOAc: 50/1) yielded **5k'** (63.1 mg, 59%) as a light yellow liquid. **^{1}H-NMR** (400 MHz, $CDCl_3$) δ = 10.57 (s, 2H), 7.60 (dd, J = 8.0, 1.7 Hz, 2H), 7.50 (ddd, J = 8.6, 7.2, 1.7 Hz, 2H), 7.07 (dd, J = 8.6, 1.2 Hz, 2H), 6.92 (ddd, J = 8.0, 7.2, 1.2 Hz, 2H). **^{13}C-NMR** (100 MHz, $CDCl_3$) δ = 202.3 (C_q), 161.7 (C_q), 135.9 (CH), 133.0 (CH), 119.8 (C_q), 118.8 (CH), 118.6 (CH). **IR** (ATR): 3043, 1614, 1588, 1482, 1254, 1232, 938, 755 cm^{-1}. **MS** (ESI) *m/z* (relative intensity): 234 (50) $[M+Na]^+$, 215 (100) $[M+H]^+$. **HR-MS** (ESI) *m/z* calc. for $C_{13}H_{11}O_3^+$ $[M+H]^+$ 215.0703, found 215.0701. The analytical data are in accordance with these reported in the literature.[5]

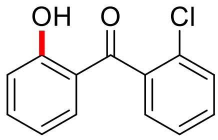

(2-Chlorophenyl)(2-hydroxyphenyl)methanone (5l): The general procedure **B** was followed using (2-chlorophenyl)(phenyl)methanone (**4l**) (108 mg, 0.50 mmol). Purification by column chromatography on silica gel (*n*hexane/EtOAc: 50/1) yielded **5l** (91.0 mg, 78%) as a colorless viscous liquid. **^{1}H-NMR** (400 MHz, $CDCl_3$) δ = 11.93 (s, 1H), 7.53–7.30 (m, 5H), 7.23 (dd, J = 8.0, 1.8 Hz, 1H), 7.05 (dd, J = 8.5,

1.2, 1H), 6.81 (dd, J = 8.2, 7.2 Hz, 1H). **^{13}C-NMR** (100 MHz, $CDCl_3$) δ = 200.6 (C_q), 163.2 (C_q), 137.3 (C_q), 137.2 (CH), 133.5 (CH), 131.2 (CH), 130.8 (C_q), 130.1 (CH), 128.5 (CH), 126.7 (CH), 119.4 (C_q), 119.1 (CH), 118.3 (CH). **IR** (ATR): 3056, 1625, 1433, 1308, 1241, 1219, 935, 754 cm^{-1}. **MS** (ESI) *m/z* (relative intensity): 487 (40) $[2M+Na]^+$, 255 (40) $[M+Na]^+$, 233 (100) $[M+H]^+$. **HR-MS** (ESI) *m/z* calc. for $C_{13}H_{10}ClO_2^+$ $[M+H]^+$ 233.0364, found 233.0366. The analytical data are in accordance with these reported in the literature.[5]

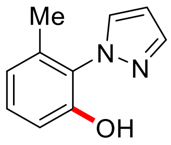

3-Methyl-2-(1*H*-pyrazol-1-yl)phenol (7a): The general procedure **A** was followed using 1-(*o*-tolyl)-1*H*-pyrazole (**6a**) (79.1 mg, 0.50 mmol). Purification by column chromatography on silica gel (*n*hexane/EtOAc: 5/1) yielded **7a** (46.2 mg, 53%) as a white solid. **M.p.**: 127-129 °C. **^{1}H-NMR** (400 MHz, $CDCl_3$) δ = 7.75 (d, J = 2.4 Hz, 1H), 7.63 (d, J = 2.4 Hz, 1H), 7.12 (dd, J = 8.2, 7.7 Hz, 1H), 6.88 (dd, J = 8.2, 1.5 Hz, 1H), 6.80 (dd, J = 7.7, 1.5 Hz, 1H), 6.49 (dd, J = 2.4, 2.4 Hz, 1H), 2.23 (s, 3H). **^{13}C-NMR** (100 MHz, $CDCl_3$) δ = 151.4 (C_q), 140.7 (CH), 132.4 (C_q), 132.1(CH), 128.9 (CH), 126.6 (C_q), 122.5 (CH), 115.3 (CH), 106.5 (CH), 18.6 (CH_3). **IR** (ATR): 3121, 1590, 1473, 1401, 1299, 1193, 1022, 755 cm^{-1}. **MS** (ESI) *m/z* (relative intensity): 371 (5) $[2M+Na]^+$, 197 (20) $[M+Na]^+$, 175 (100) $[M+H]^+$. **HR-MS** (ESI) *m/z* calc. for $C_{10}H_{11}N_2O^+$ $[M+H]^+$ 175.0866, found 175.0868.

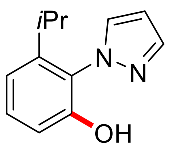

3-Isopropyl-2-(1*H*-pyrazol-1-yl)phenol (7b): The general procedure **A** was followed using 1-(2-isopropylphenyl)-1*H*-pyrazole (**6b**) (93.1 mg, 0.50 mmol). Purification by column chromatography on silica gel (*n*hexane/EtOAc: 5/1) yielded **7b** (47.5 mg,

47%) as a white solid. **M.p.**: 122-124 ℃. **^{1}H-NMR** (400 MHz, $CDCl_3$) δ = 7.74 (d, J = 2.2, 1H), 7.55 (d, J = 2.2, 1H), 7.19 (dd, J = 7.9, 8.1 Hz, 1H), 6.90 (dd, J = 7.9, 1.4 Hz, 1H), 6.78 (dd, J = 8.1, 1.4 Hz, 1H), 6.48 (t, J = 2.2 Hz, 1H), 2.73 (hept, J = 6.8 Hz, 1H), 1.15 (d, J = 6.8 Hz, 6H). **^{13}C-NMR** (100 MHz, $CDCl_3$) δ = 151.7 (C_q), 145.0 (C_q), 141.0 (CH), 132.5 (CH), 129.8 (CH), 125.9 (C_q), 117.7 (CH), 114.7 (CH), 106.7 (CH), 28.0 (CH), 24.0 (CH_3). **IR** (ATR): 3119, 1527, 1432, 1401, 1332, 1167, 1053, 753 cm^{-1}. **MS** (ESI) *m/z* (relative intensity): 225 (20) $[M+Na]^+$, 203 (100) $[M+H]^+$. **HR-MS** (ESI) *m/z* calc. for $C_{12}H_{15}N_2O^+$ $[M+H]^+$ 203.1179, found 203.1180.

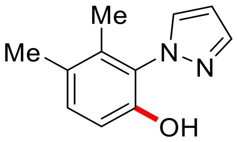

3,4-Dimethyl-2-(1*H*-pyrazol-1-yl)phenol (7c): The general procedure **A** was followed using 1-(2,3-dimethylphenyl)-1*H*-pyrazole (**6c**) (86.1 mg, 0.50 mmol). Purification by column chromatography on silica gel (*n*hexane/EtOAc: 5/1) yielded **7c** (43.3 mg, 46%) as a white solid. **M.p.**: 113-115 ℃. **^{1}H-NMR** (400 MHz, $CDCl_3$) δ = 7.76 (d, J = 2.1 Hz, 1H), 7.57 (d, J = 2.1 Hz, 1H), 7.04 (d, J = 8.2 Hz, 1H), 6.78 (d, J = 8.2 Hz, 1H), 6.49 (dd, J = 2.1, 2.1 Hz, 1H), 2.23 (s, 3H), 2.05 (s, 3H). **^{13}C-NMR** (100 MHz, $CDCl_3$) δ = 149.6 (C_q), 141.0 (CH), 132.5 (CH), 131.4 (C_q), 130.4 (CH), 129.0 (C_q), 127.0 (C_q), 114.3 (CH), 106.6 (CH), 19.7 (CH_3), 15.2 (CH_3). **IR** (ATR): 3129, 1521, 1472, 1401, 1298, 1194, 1014, 756 cm^{-1}. **MS** (ESI) *m/z* (relative intensity): 211 (35) $[M+Na]^+$, 189 (100) $[M+H]^+$. **HR-MS** (ESI) *m/z* calc. for $C_{11}H_{13}N_2O^+$ $[M+H]^+$ 189.1022, found 189.1022.

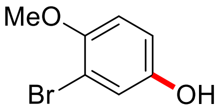

3-Bromo-4-methoxyphenol (9a): The general procedure **C** was followed using 1-bromo-2-methoxybenzene (**8a**) (93.0 mg, 0.50 mmol). Purification by column chromatography on silica gel (*n*hexane/EtOAc: 5/1) yielded **9a** (52.8 mg, 52%) as a

green oil. ^{1}**H-NMR** (600 MHz, $CDCl_3$) δ = 7.06 (d, J = 2.8 Hz, 1H), 6.77 (d, J = 8.8 Hz, 1H), 6.74 (dd, J = 8.8, 2.8 Hz, 1H), 4.47 (s, 1H), 3.81 (s, 3H). ^{13}**C-NMR** (150 MHz, $CDCl_3$) δ = 150.4 (C_q), 149.8 (C_q), 120.5 (CH), 115.0 (CH), 113.2 (CH), 112.0 (C_q), 56.9 (CH_3). **IR** (ATR): 3363, 2942, 2837, 1495, 1276, 1206, 1048, 744 cm^{-1}. **MS** (ESI) *m/z* (relative intensity): 203 (95) $[M+H]^+$ (^{79}Br), 201 (100). **HR-MS** (ESI) *m/z* calc. for $C_7H_8{}^{79}BrO_2^+$ $[M+H]^+$ 202.9537, found 202.9536.[8]

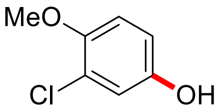

3-Chloro-4-methoxyphenol (9b): The general procedure **C** was followed using 1-chloro-2-methoxybenzene (**8b**) (71.0 mg, 0.50 mmol). Purification by column chromatography on silica gel (*n*hexane/EtOAc: 5/1) yielded **9b** (31.7 mg, 40%) as a green oil. ^{1}**H-NMR** (600 MHz, $CDCl_3$) δ = 6.89 (d, J = 3.0 Hz, 1H), 6.79 (d, J = 8.8 Hz, 1H), 6.68 (dd, J = 8.8, 3.0 Hz, 1H), 4.35 (s, 1H), 3.82 (s, 3H). ^{13}**C-NMR** (150 MHz, $CDCl_3$) δ = 149.6 (C_q), 149.4 (C_q), 123.0 (C_q), 117.6 (CH), 114.2 (CH), 113.5 (CH), 56.8 (CH_3). **IR** (ATR): 3125, 2916, 1501, 1265, 1059, 908, 729, 650 cm^{-1}. **MS** (ESI) *m/z* (relative intensity): 157 (100) $[M-H]^-$ (^{35}Cl), 142 (50). **HR-MS** (ESI) *m/z* calc. for $C_7H_6{}^{35}ClO_2^-$ $[M-H]^-$ 157.0062, found 157.0062. The analytical data are in accordance with these reported in the literature.[9]

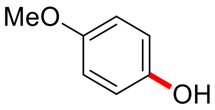

4-Methoxyphenol (9c): The general procedure **C** was followed using anisole (**8c**) (54.1 mg, 0.50 mmol). Purification by column chromatography on silica gel (*n*hexane/EtOAc: 5/1) yielded **9c** (33.0 mg, 53%) as a white solid. **M.p.**: 55-57 °C. ^{1}**H-NMR** (600 MHz, $CDCl_3$) δ = 6.85–6.72 (m, 4H), 5.60 (s, 1H), 3.75 (s, 3H). ^{13}**C-NMR** (150 MHz, $CDCl_3$) δ = 153.5 (C_q), 149.5 (C_q), 116.1 (CH), 114.9 (CH), 55.8 (CH_3). **IR** (ATR): 3397, 2955, 1512, 1273, 1237, 1040, 807, 736 cm^{-1}. **MS** (EI) *m/z* (relative intensity): 124 (90) $[M^+]$, 109 (100), 81 (50), 53 (27). **HR-MS** (EI) *m/z* calc. for $C_7H_8O_2$ $[M^+]$ 124.0524, found 124.0528. The analytical data are in

accordance with these reported in the literature.[10]

Mechanistic Studies

H/D Exchange Experiment

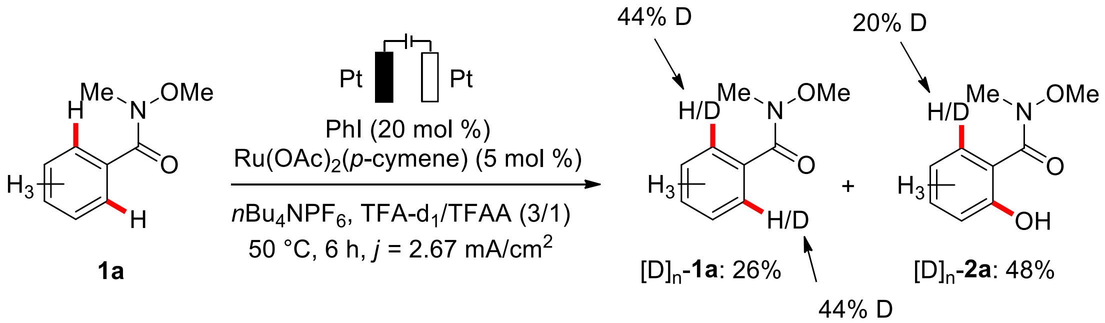

In an undivided cell with Pt electrodes (10 mm x 15 mm x 0.125 mm), **1a** (82.5 mg, 0.50 mmol), PhI (20.4 mg, 20 mol %), $Ru(OAc)_2$(*p*-cymene) (8.8 mg, 5.0 mol %) and *n*Bu_4NPF_6 (194 mg, 1.0 equiv) were added in TFA-d_1/TFAA (3:1, 3.0 mL). Electrocatalysis was performed at 50 ℃ with a constant current of 4 mA maintained for 6 h. Thereafter, a saturated aqueous $NaHCO_3$ (25 mL) was added and the reaction mixture was extracted with EtOAc (3 × 15 mL). The combined organic layers were washed with brine (25 mL), dried over Na_2SO_4, filtered and concentrated *in vacuo*. Column chromatography (*n*hexane/EtOAc: 5/1) yielded $[D]_n$-**1a** (21.5 mg, 26%) as a colourless oil and $[D]_n$-**2a** (43.4 mg, 46%) as a colorless oil. The D-incorporation was determined by ^{1}H-NMR spectroscopy.

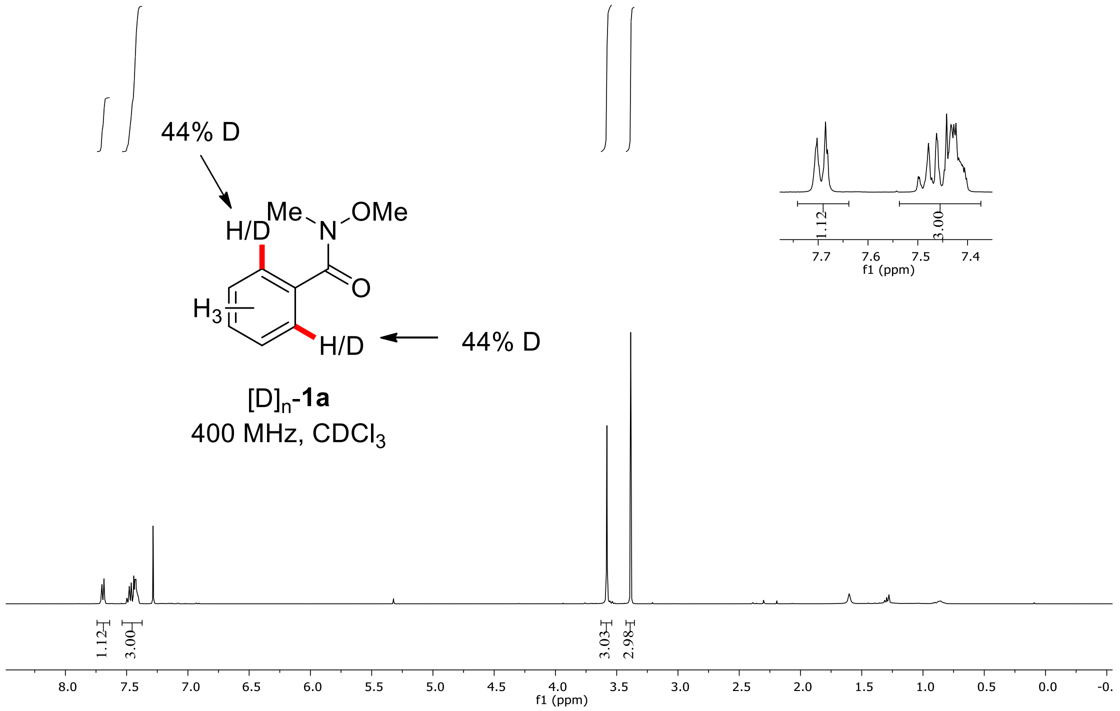

Figure S1. ^{1}H-NMR of $[D]_n$-**1a** from the deuteration study.

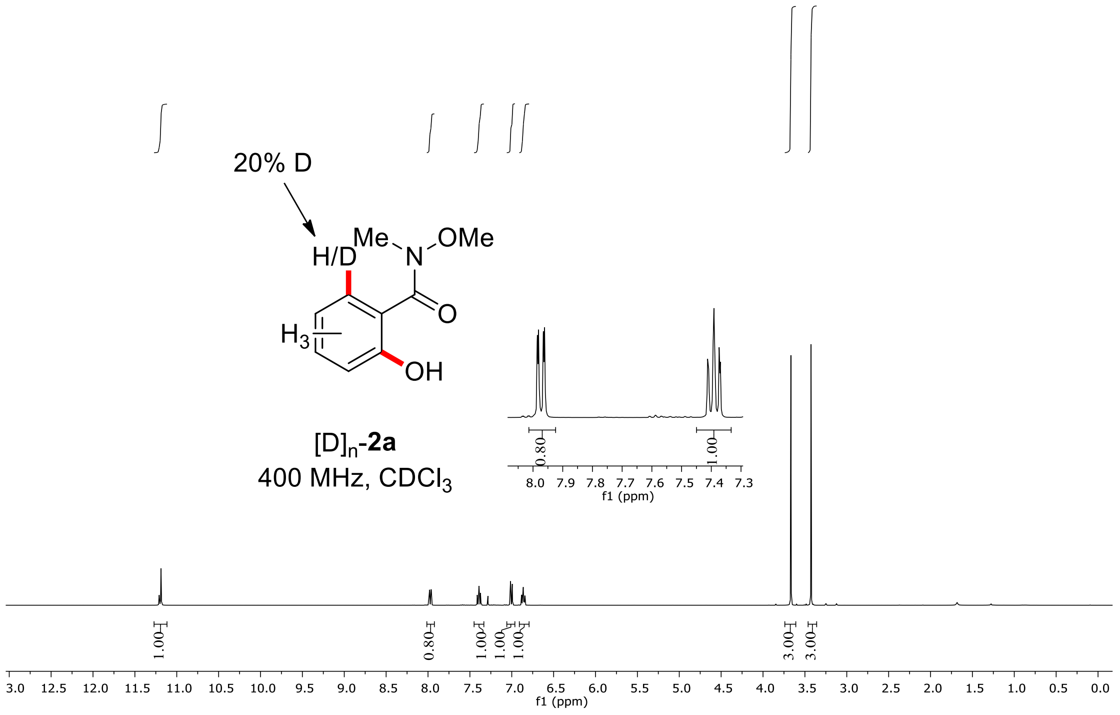

Figure S2. ^{1}H-NMR of $[D]_n$-**2a** from the deuteration study.

Kinetic Isotope Effect (KIE)

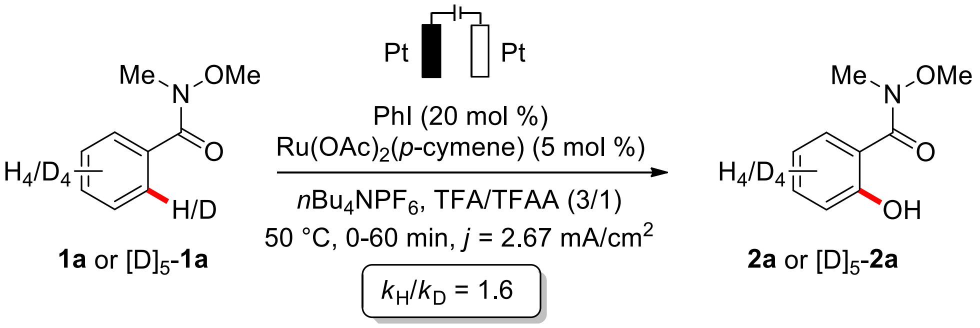

Two parallel reactions using substrates **1a** and $[D]_5$–**1a** (0.50 mmol each) were carried out to determine the KIE by comparison of the initial rates. In undivided cells with Pt electrodes (10 mm x 15 mm x 0.125 mm), **1a** (82.5 mg, 0.50 mmol) or $[D]_5$–**1a** (85.1 mg, 0.50 mmol) respectevely, PhI (20.4 mg, 20 mol %), $Ru(OAc)_2$(*p*-cymene) (8.8 mg, 5.0 mol %) and nBu_4NPF_6 (194 mg, 1.0 equiv) were added in TFA/TFAA (3:1, 4.0 mL). Electrocatalysis was performed at 50 ℃ with a constant current of 4 mA. After 20, 40 and 60 minutes, aliquots of 0.2 mL were collected from the cell. Each aliquot was then treated with a saturated aqueous $NaHCO_3$ (2.0 mL) and extracted with CH_2Cl_2 (3 x 1.0 mL). After evaporation of the solvents, the crude mixture was analized by ^{1}H-NMR spectroscopy using CH_2Br_2 as the internal standard. The measured yields of **2a** and $[D]_5$–**2a** were plotted and a linear fit revealed a KIE of $k_H/k_D \approx 1.6$ (Figure S3).

***t*/ min**	**20**	**40**	**60**
**2a / %**	1	3	6
**$[D]_5$-2a / %**	0	1.6	3

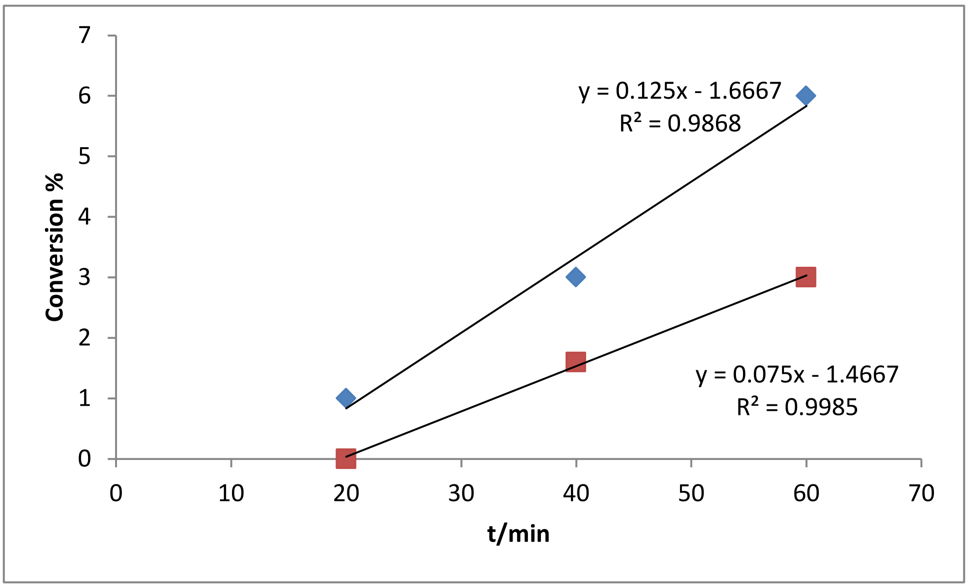

Figure S3. Linear fit for the reaction rates of **1a** and $[D]_5$–**1a**.

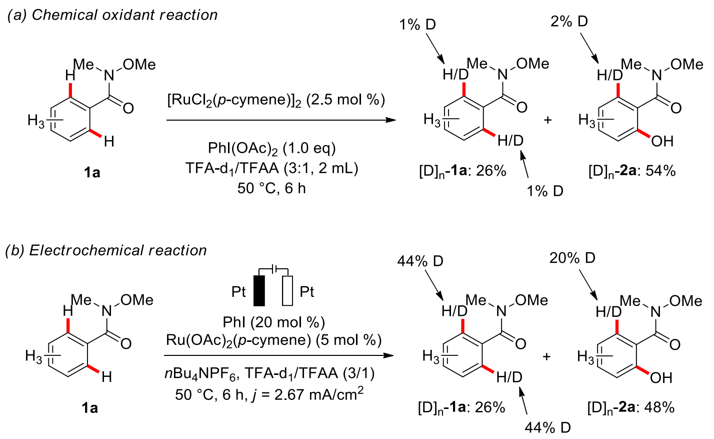

Scheme S1. Comparison of H/D exchange between the chemical oxidant reaction and the electrochemical reaction.

Competition Experiments

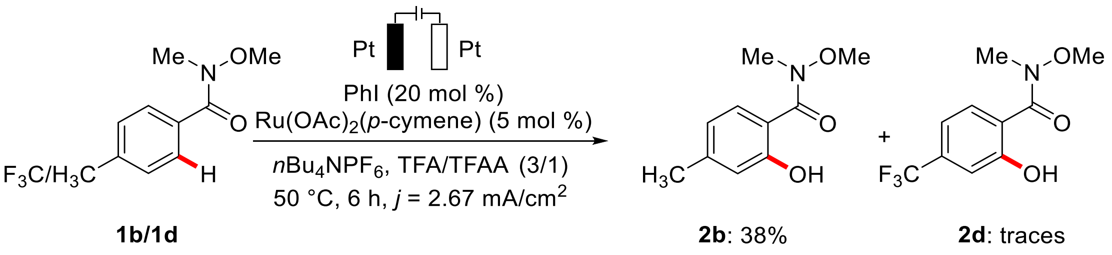

In an undivided cell with Pt electrodes (10 mm x 15 mm x 0.125 mm), **1b** (44.8 mg, 0.25 mmol), **1d** (58.5 mg, 0.25 mmol), PhI (20.4 mg, 20 mol %), $Ru(OAc)_2$(*p*-cymene) (8.8 mg, 5.0 mol %) and nBu_4NPF_6 (194 mg, 1.0 equiv) were added in TFA/TFAA (3:1, 3.0 mL). Electrocatalysis was performed at 50 ℃ with a constant current of 4 mA maintained for 6 h. Thereafter, a saturated aqueous $NaHCO_3$ (25 mL) was added and the reaction mixture was extracted with EtOAc (3 × 15 mL). The combined organic layers were washed with brine (25 mL), dried over Na_2SO_4, filtered and concentrated *in vacuo*. Purification by column chromatography on silica gel (*n*hexane/EtOAc: 10/1→5/1) yielded **2b** (31.8 mg, 38%) as a colourless solid. **2d** was obtained only in trace amount.

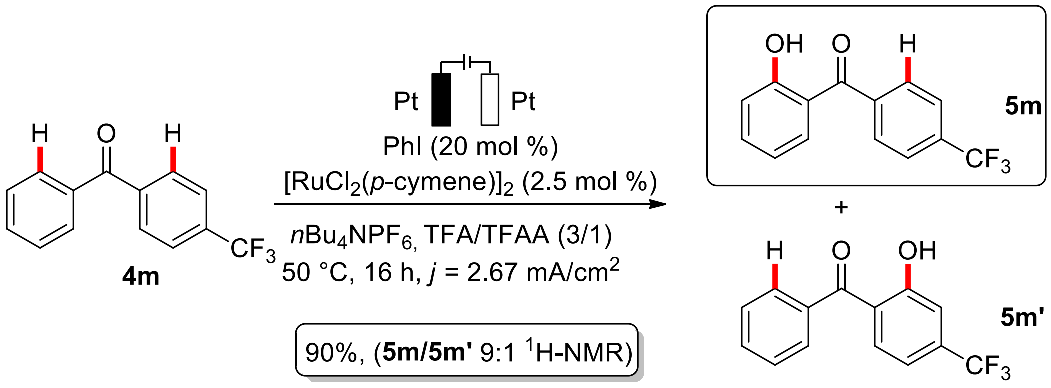

The general procedure **B** was followed using **4m** (125 mg, 0.50 mmol). Purification by column chromatography on silica gel (*n*hexane/EtOAc: 50/1) yielded **5m** and **5m′** (120 mg) as a mixture of 9:1 (according to ^{1}H-NMR spectroscopic analysis), pure **5m**

was obtained by recrystallization in CH_2Cl_2/*n*hexane, as a colorless solid. **M.p.**: 59-60 ℃. **^{1}H-NMR** (400 MHz, $CDCl_3$) δ = 11.85 (s, 1H), 7.76 (s, 4H), 7.59–7.42 (m, 2H), 7.08 (dd, J = 8.4, 1.2 Hz, 1H), 6.87 (ddd, J = 8.2, 7.2, 1.2 Hz, 1H). **^{13}C-NMR** (100 MHz, $CDCl_3$) δ = 200.4 (C_q), 163.4 (C_q), 141.0 (C_q), 137.0 (CH), 133.4 (q, $^2J_{C–F}$ = 32.8 Hz, C_q), 133.3 (CH), 129.3 (CH), 125.4 (d, $^3J_{C–F}$ = 3.8 Hz, CH), 123.6 (q, $^1J_{C–F}$ = 272.6 Hz, C_q), 118.9 (CH), 118.7 (C_q), 118.7 (CH). **^{19}F-NMR** (376 MHz, $CDCl_3$) δ = –63.08 (s). **IR** (ATR): 3074, 1623, 1323, 1306, 1207, 1137, 939, 848 cm^{-1}. **MS** (ESI) *m/z* (relative intensity): 265 (100) $[M–H]^-$. **HR-MS** (ESI) *m/z* calc. for $C_{14}H_8F_3O_2^-$ $[M–H]^-$ 265.0482, found 265.0483. The analytical data are in accordance with those reported in the literature.[11]

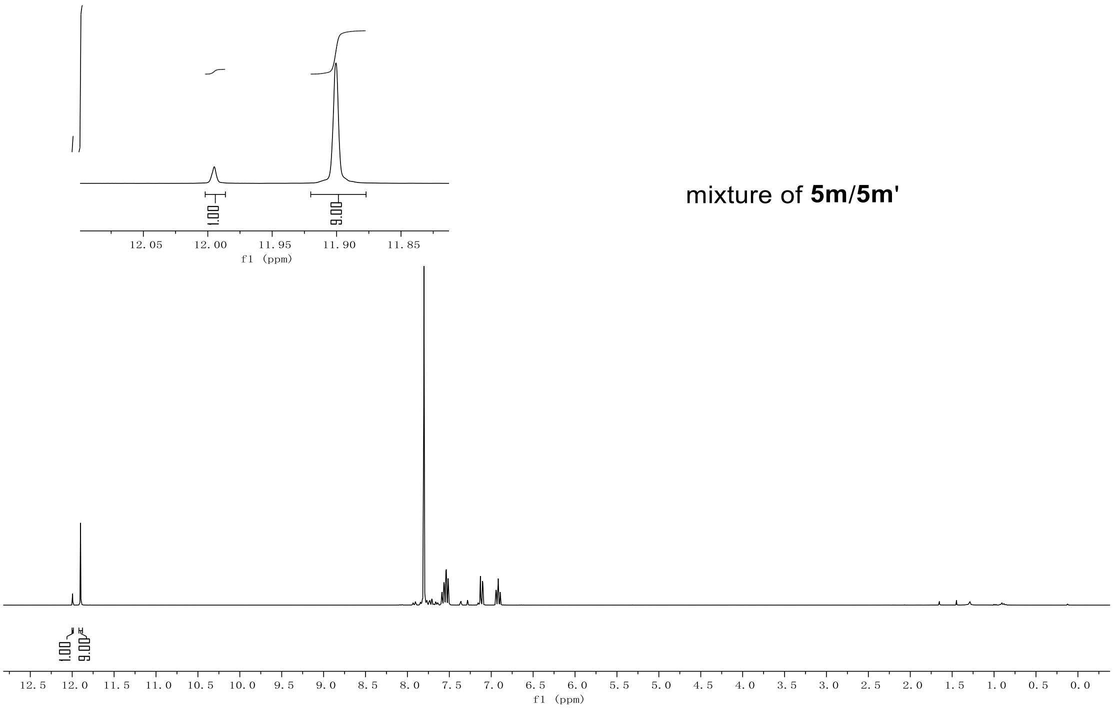

Figure S4. ^{1}H-NMR of **5m/5m'** from the competition experiment.

Directing Group Competition

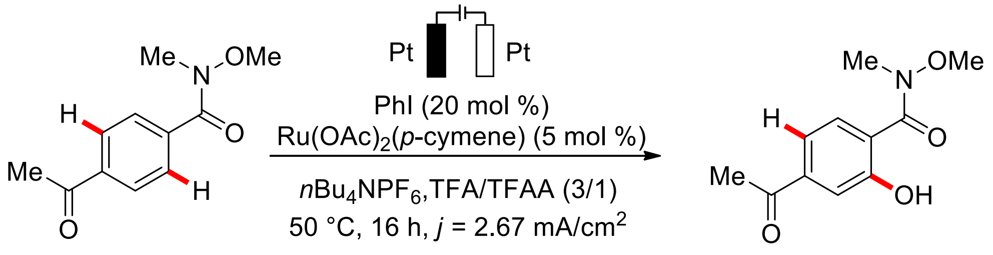

1x **2x**: 61%

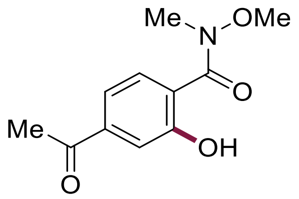

4-Acetyl-2-hydroxy-*N*-methoxy-*N*-methylbenzamide (2x): The general procedure **A** was followed using 4-acetyl-*N*-methoxy-*N*-methylbenzamide (**1x**) (104 mg, 0.50 mmol). Isolation by column chromatography (*n*hexane/EtOAc: 8/1→3/1) yielded **2x** (67.8 mg, 61%) as a colourless liquid. **^{1}H-NMR** (400 MHz, $CDCl_3$) δ = 10.97 (s, 1H), 8.06–7.90 (d, J = 8.4 Hz, 1H), 7.49 (d, J = 1.8 Hz, 1H), 7.38 (dd, J = 8.4, 1.8 Hz, 1H), 3.61 (s, 3H), 3.39 (s, 3H), 2.56 (s, 3H). **^{13}C-NMR** (100 MHz, $CDCl_3$) δ = 197.6 (C_q), 168.7 (C_q), 160.7 (C_q), 140.8 (C_q), 129.8 (CH), 118.0 (C_q), 117.9 (CH), 117.7 (CH), 61.4 (CH_3), 33.8 (CH_3), 26.7 (CH_3). **IR** (ATR): 2937, 1684, 1630, 1582, 1415, 1298, 1205, 823 cm^{-1}. **MS** (ESI) *m/z* (relative intensity): 246 (30) $[M+Na]^+$, 224 (100) $[M+H]^+$. **HR-MS** (ESI) *m/z* calc. for $C_{11}H_{14}NO_4^+$ $[M+H]^+$ 224.0917, found 224.0917.

Gram-Scale Synthesis of 2a

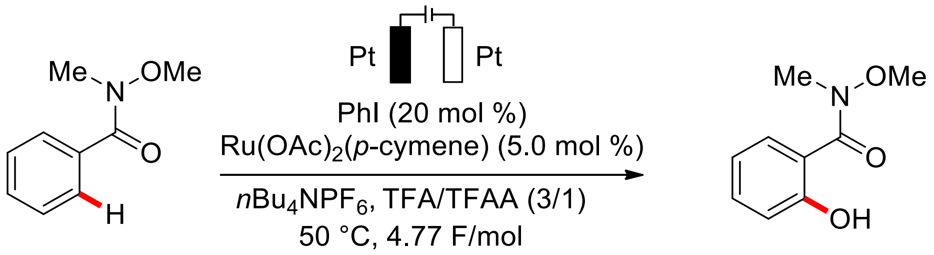

1a **2a**: 84%
1.29 g

To an undivided two-necked flask (diameter: 40 mm; length: 130 mm; volume: 120 mL) equipped with a teflon-coated magnetic stirring bar and teflon cap, platinum electrodes (25 mm x 50 mm x 0.125 mm), **1a** (1.40 g, 8.5 mmol), PhI (347 mg, 1.7 mmol), $Ru(OAc)_2$(*p*-cymene) (150 mg, 0.43 mmol), *n*Bu_4NPF_6 (3.29 g, 8.4 mmol) were added in TFA/TFAA (3:1, 40 mL). Electrocatalysis was performed at 50 ℃ with a constant current of 20 mA maintained for 72 h. Thereafter, a saturated aqueous $NaHCO_3$ (200 mL) was added and the reaction mixture was extracted with EtOAc (3 × 50 mL). The combined organic layers were washed with brine (100 mL), dried over Na_2SO_4, filtered and concentrated *in vacuo*. The crude product was purified by column chromatography on silica gel (*n*hexane/EtOAc: 6/1) and yielded **2a** (1.29 g, 84%) as a colorless oil.

Cyclic Voltammetry and Rotating Disc Electrode Experiments

The cyclic voltammetry measurements were carried out using a Metrohm Autolab PGSTAT204 workstation and following analysis was performed with Nova 2.1 software. For all experiments a glassy-carbon (GC) electrode (3 mm-diameter, disc-electrode) or a platinum electrode (3 mm-diameter, disc-electrode) was used as the working electrode, a platinum wire was used as the counter electrode and an Ag/Ag^+ electrode with ferrocene as an internal standard or a saturated calomel electrode (SCE) was used as a reference electrode, respectively. The measurements were recorded at a scan rate of 100 mVs^{-1}, if not indicated otherwise. The rotating-disc electrode (RDE) experiments were performed using a Metrohm Autolab PGSTAT204 workstation and a RRDE-3A Rotating Ring Disk Electrode Apparatus Ver.2.0, ALS Japan Co., Ltd. For the RDE experiments a glassy-carbon electrode (4 mm-diameter, disc-electrode) was used as the working electrode, a coiled platinum wire was used as the counter electrode and a non-aqueous $Ag/AgNO_3$ in acetonitrile was used as the reference electrode. The operation temperature was 298 K. All solutions were degassed via freeze-pump-thaw method prior to use and nitrogen was bubbled through the solutions for at least 5 min before the experiment was performed. The experiments were performed under inert conditions (constant flow of dry nitrogen).

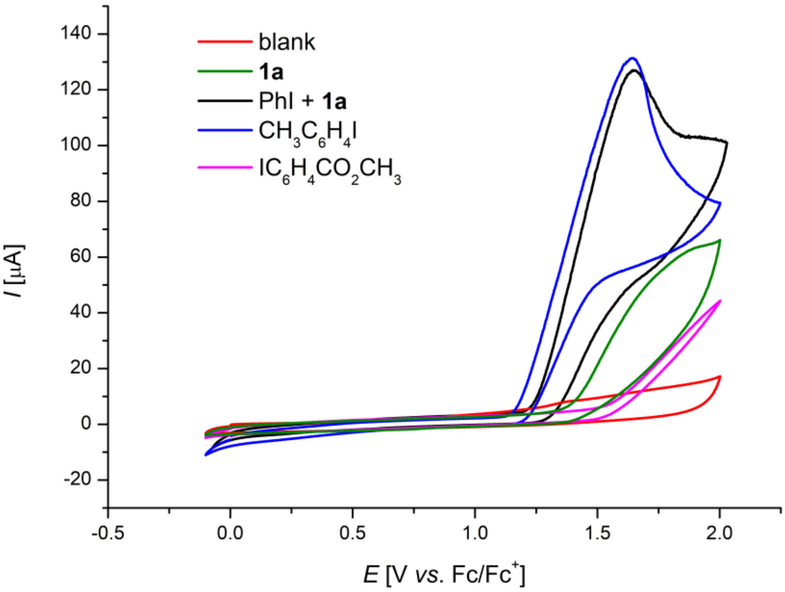

Figure S5. Cyclic voltammograms at 100 mVs^{-1} using TFA and nBu$_4$NPF$_6$ (0.1 M) as the electrolyte and a GC working electrode; the concentration of all substrates was 5.0 mM. (red) blank, (green) **1a**, (black) iodobenzene + **1a**, (blue) 4-iodotoluene, (magenta) methyl 4-iodobenzoate.

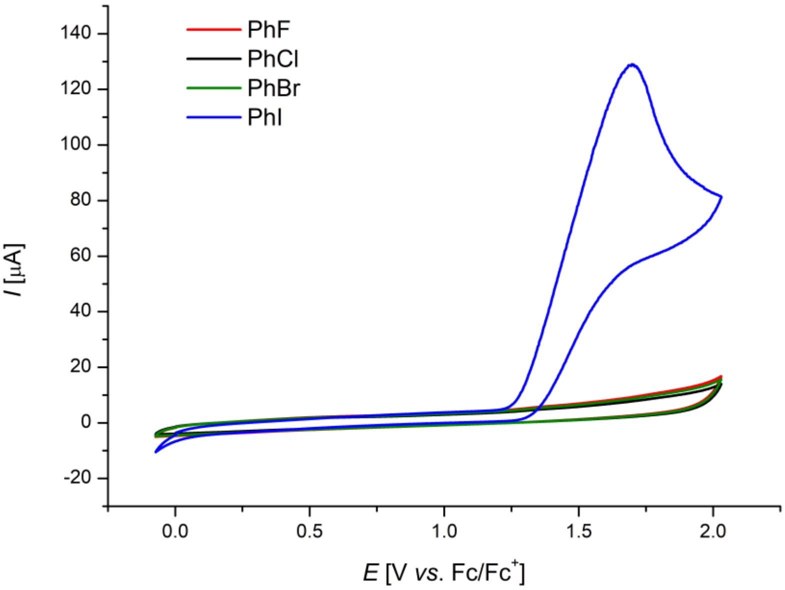

Figure S6. Cyclic voltammograms at 100 mVs^{-1} using TFA and nBu$_4$NPF$_6$ (0.1 M) as the electrolyte and a GC working electrode; the concentration of all substrates was 5.0 mM. (red) fluorobenzene, (black) chlorobenzene, (green) bromobenzene, (blue) iodobenzene.

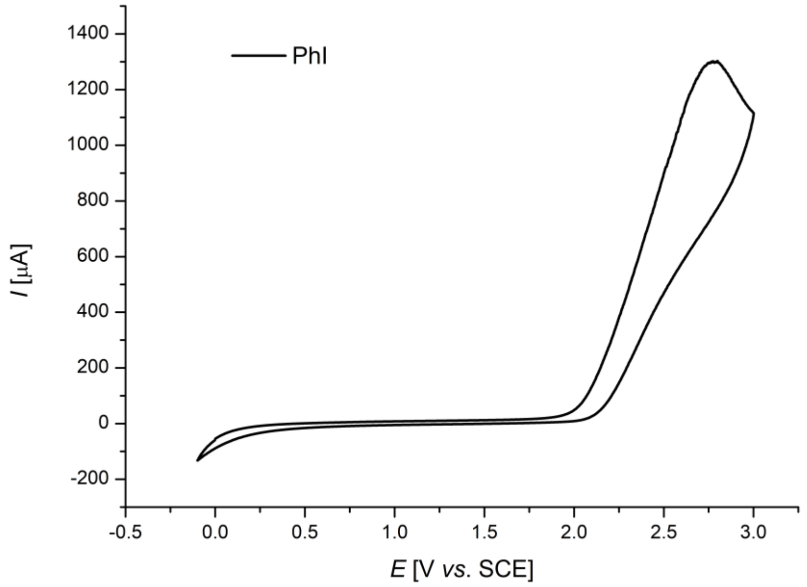

Figure S7. Cyclic voltammogram at 500 mVs^{-1} using MeCN and nBu$_4$NPF$_6$ (0.1 M) as the electrolyte and a GC working electrode; concentration of iodobenzene 5.0 mM.

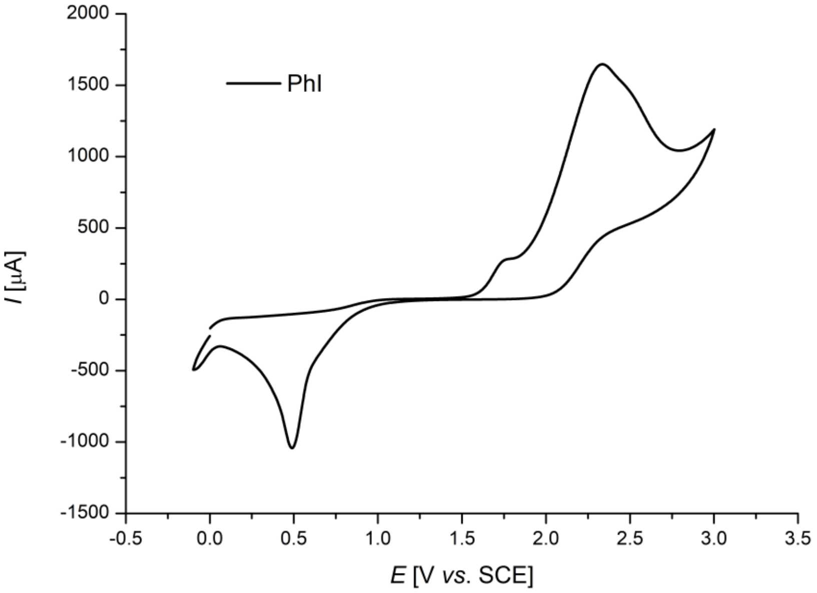

Figure S8. Cyclic voltammogram at 500 mVs^{-1} using MeCN and nBu$_4$NPF$_6$ (0.1 M) as the electrolyte, recorded with a platinum-disc electrode; concentration of iodobenzene 5.0 mM.

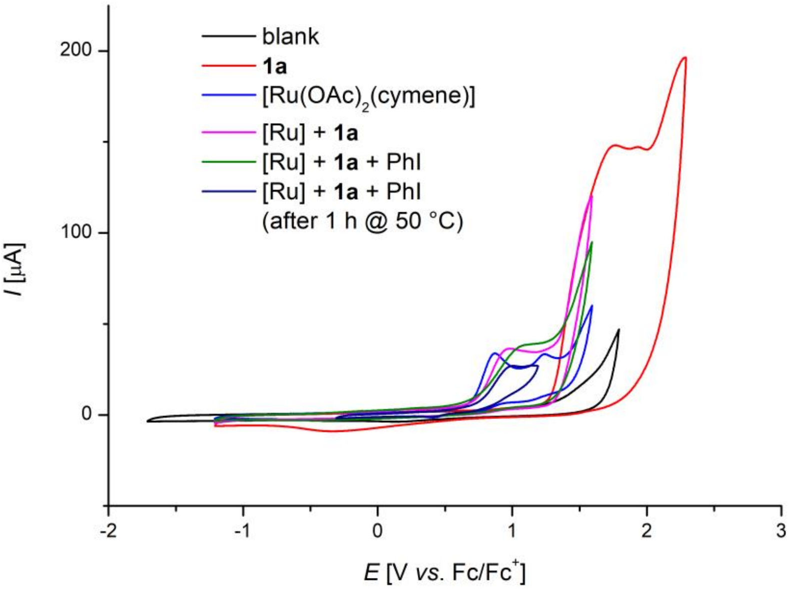

Figure S9. Cyclic voltammograms at 100 mVs^{-1} using DCE and nBu$_4$PF$_6$ (0.1 M) as the electrolyte and a GC working electrode; the concentration of all substrates was 5.0 mM. [Ru] = Ru(OAc)$_2$(*p*-cymene).

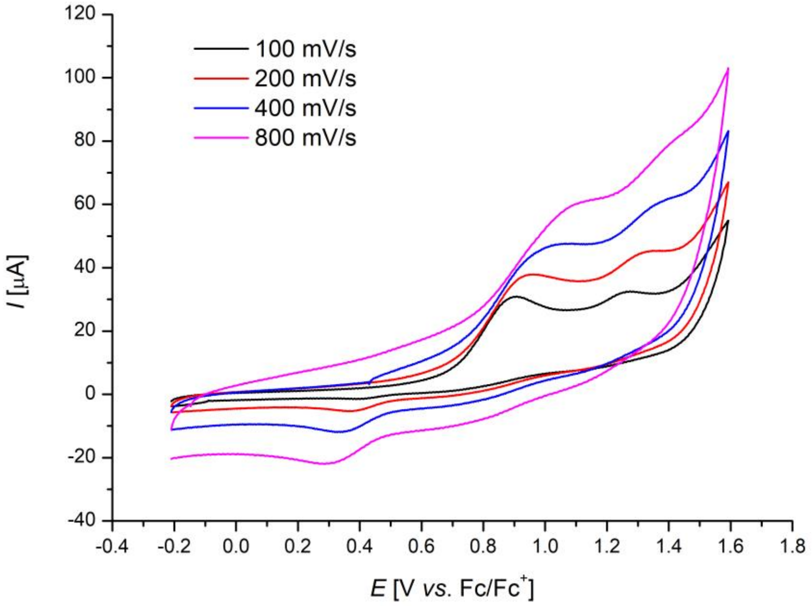

Figure S10. Cyclic voltammograms of [Ru(OAc)$_2$(*p*-cymene)] at different scan-rates using DCE and nBu$_4$PF$_6$ (0.1 M) as the electrolyte and a GC working electrode; concentration of Ru(OAc)$_2$(*p*-cymene) was 3.0 mM.

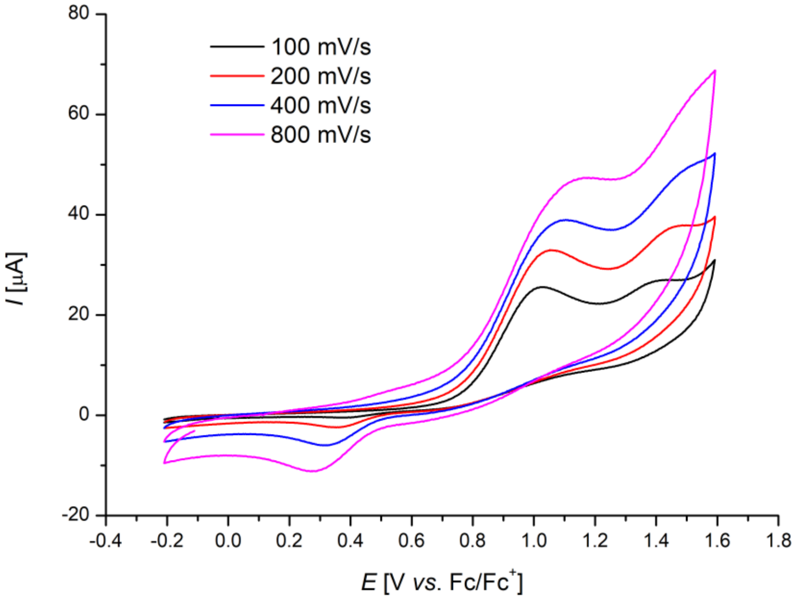

Figure S11. Cyclic voltammograms of [Ru(OAc)$_2$(cymene)] at different scan-rates, using DCE and *n*Bu$_4$PF$_6$ (0.1 M) as the electrolyte and a Pt working electrode; concentration of Ru(OAc)$_2$(*p*-cymene) was 3.0 mM.

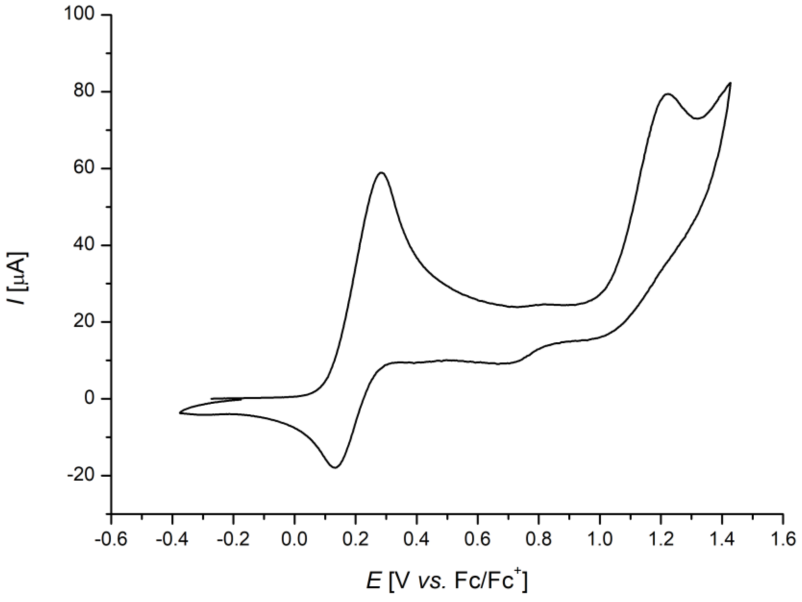

Figure S12. Cyclic voltammogram at 100 mVs^{-1} using DCE and *n*Bu$_4$NPF$_6$ (0.1 M) as the electrolyte and a GC working electrode; concentration of complex **10** was 3.86 mM. A non-aqueous Ag/AgNO$_3$ in acetonitrile was used as the reference electrode.

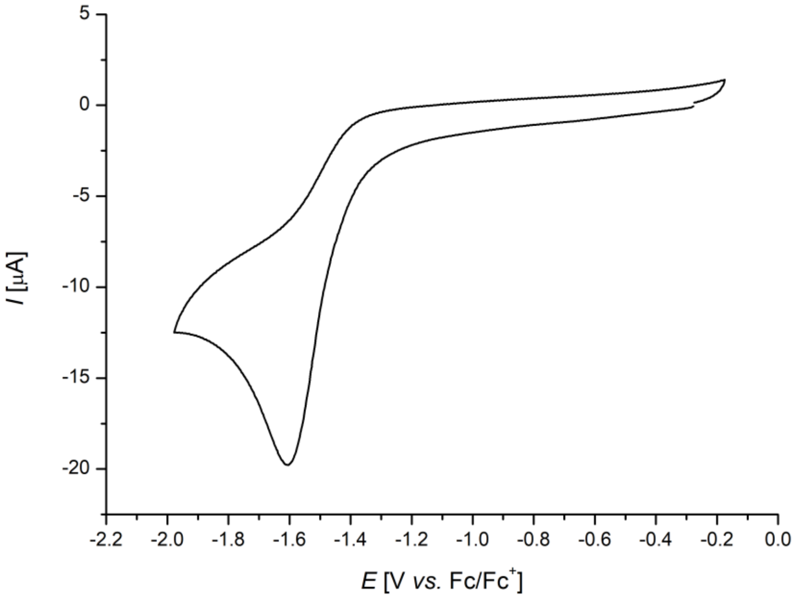

Figure S13. Cyclic voltammogram at 100 mVs^{-1} using DCE and nBu$_4$NPF$_6$ (0.1 M) as the electrolyte and a GC working electrode; concentration of complex **10** was 3.86 mM. A non-aqueous Ag/AgNO$_3$ in acetonitrile was used as the reference electrode.

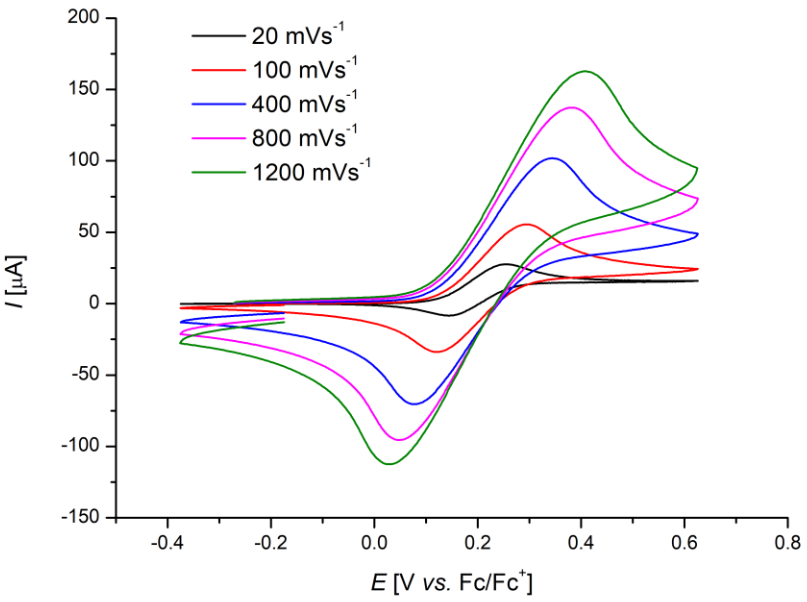

Figure S14. Cyclic voltammograms at different scan-rates using DCE and nBu$_4$NPF$_6$ (0.1 M) as the electrolyte and a GC working electrode; concentration of complex **10** was 3.86 mM. A non-aqueous Ag/AgNO$_3$ in acetonitrile was used as the reference electrode.

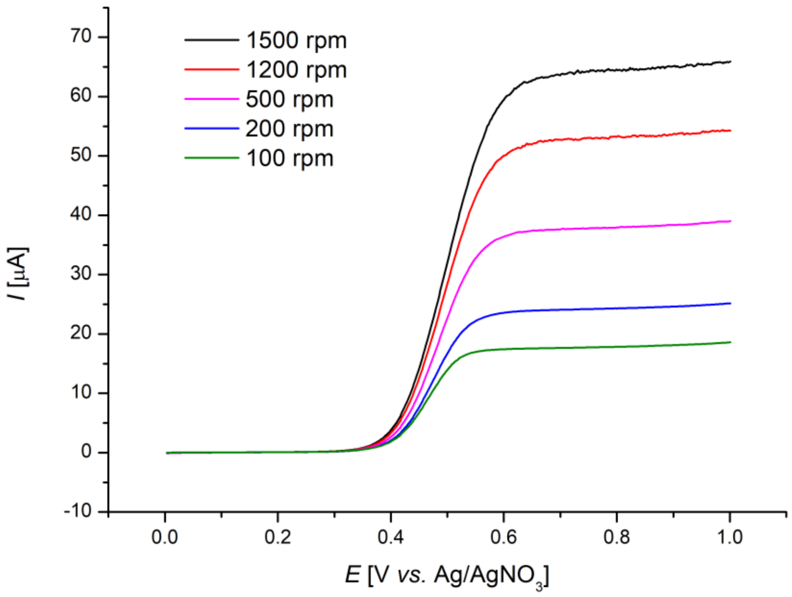

Figure S15. Linear sweep voltammetry of complex **10** at different rotation speeds, using a GC rotating-disc electrode. DCE and nBu$_4$NPF$_6$ (0.1 M) was used as the electrolyte; a scan-rate of 20 mVs^{-1} and a concentration of complex **10** 3.859 mM, was used. A non-aqueous Ag/AgNO$_3$ in acetonitrile was used as the reference electrode.

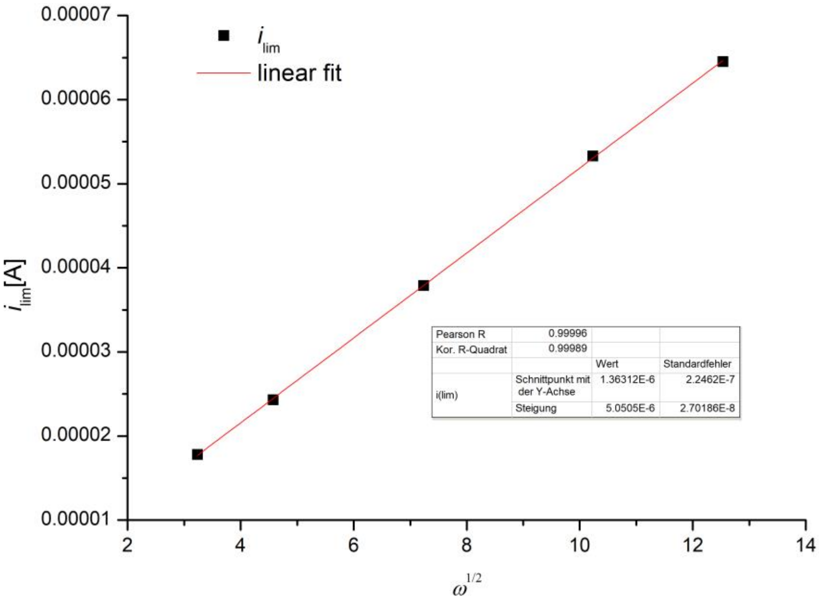

Figure S16. Linear fit for the Levich-equation.

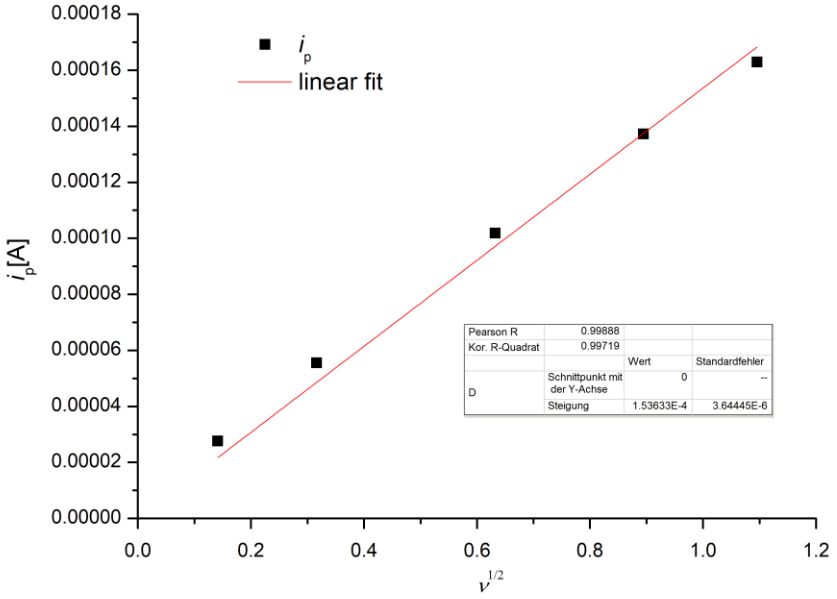

Figure S17. Linear fit for the Randles-Ševčík equation.

The transferred number of electrons *n* was determined through the combination of stationary and transient electrochemical techniques, according to the known method by Amatore *et al.*.[12]

<u>Randles-Ševčík Equation:</u>

$$i_{\mathrm{p}} = 0.4463\sqrt{\left(\frac{F^3}{RT}\right)}CAn^{\frac{3}{2}}\sqrt{D}\sqrt{v}$$

at T = 298 K:

$$i_{\mathrm{p}} = (2.687 * 10^5)CAn^{\frac{3}{2}}\sqrt{D}\sqrt{v}$$

with:

$$C = 3.9 * 10^{-6}\,\frac{\mathrm{mol}}{\mathrm{cm}^3}$$
$$A = 0.071\ \mathrm{cm}^2$$

<u>and the Levich Equation:</u>

$$i_{\mathrm{lim}} = 0.62FACnD^{\frac{2}{3}}v^{-\frac{1}{6}}\omega^{\frac{1}{2}}$$

with:

$$C = 1.0 * 10^{-6}\,\frac{\mathrm{mol}}{\mathrm{cm}^3}$$
$$A = 0.126\ \mathrm{cm}^2$$

$$\Rightarrow n = \frac{S_1^{\frac{4}{3}}}{S_2} = \mathbf{0.935 \pm 0.206}$$

Reaction Profile for the Formation of Hypervalent Iodine Reagents

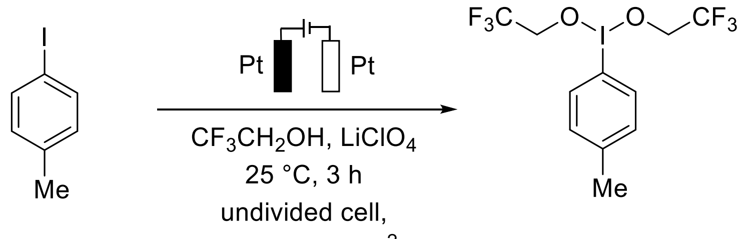

The flow-NMR study was carried out in an undivided cell with platinum electrodes (10 mm x 15 mm x 0.125 mm). The cell was charged with 4-iodotoluene (109 mg, 0.50 mmol, 1.00 equiv) and $LiClO_4$ (63.8 mg, 0.60 mmol, 1.20 equiv) in TFE (10 mL). After recording initial spectra, the electrolysis was initiated by applying a constant current of 10 mA at 25 ℃. The *in-line* electrolysis and NMR-studies were performed for 3 h. The reaction solution was circulated between the benchtop NMR and the electrochemical cell with a flow speed of 0.8 mL/min. The reaction profile was monitored with a ^{1}H-NMR spectrum recorded after every 1.5 minutes. After the electrolysis was stopped, the spectra were batch processed with the reaction monitoring wizard of the MestreNova 12.0.3 software. Arbitrary integral values were transformed to mmol and percentage values by referencing with dibromomethane as internal standard. Accordingly, the product *p*-tolylbis(2,2,2-trifluoroethoxy)-λ^3-iodane **11a** was formed in 78% NMR-conversion (see Figure S18). Signals at a chemical shift (referenced against the trifluoroethanol solvent peak 4.81 ppm) of 1.96 ppm and 2.19 ppm were identified to originate from the starting material and the product, respectively.

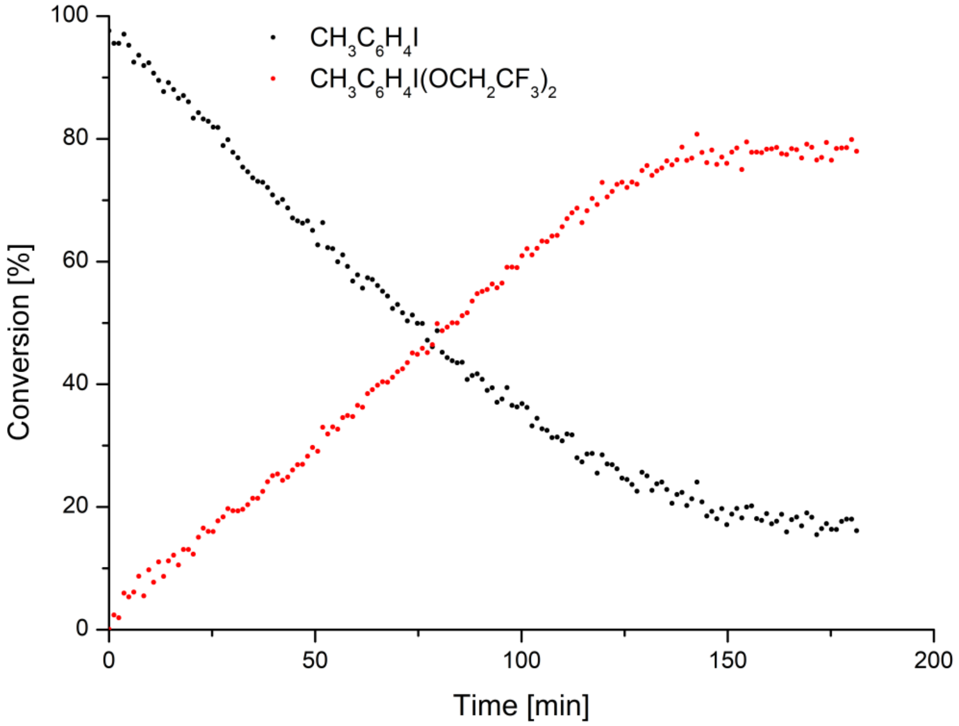

Figure S18. Reaction monitoring for the formation of *p*-tolylbis(2,2,2-trifluoroethoxy)-λ[3]-iodane **11a**.

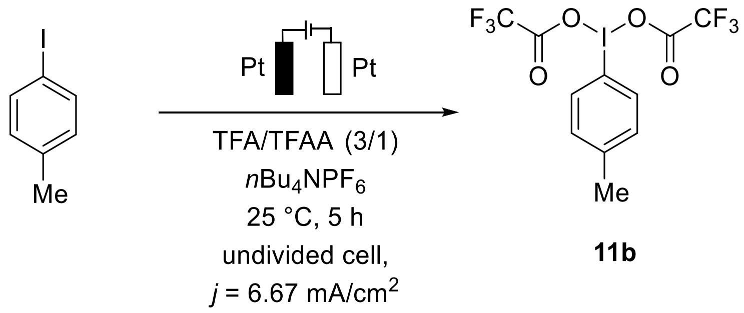

11b

The flow-NMR study was carried out in an undivided cell with platinum electrodes (10 mm x 15 mm x 0.125 mm). The cell was charged with 4-iodotoluene (109 mg, 0.50 mmol, 1.00 equiv) and *n*Bu_4NPF_6 (194 mg, 0.50 mmol, 1.00 equiv) in TFA/TFAA (10 mL, 3:1 v/v). After recording initial spectra, the electrolysis was initiated by applying a constant current of 10 mA at 25 °C. The *in-line* electrolysis and NMR-studies were performed for 5 h. The reaction solution was circulated

between the benchtop NMR and the electrochemical cell with a flow speed of 0.8 mL/min. The reaction profile was monitored with a ^{1}H NMR spectrum recorded after every 1.5 minutes. After the electrolysis was stopped, the spectra were batch processed with the reaction monitoring wizard of the MestreNova 12.0.3 software. Arbitrary integral values were transformed to mmol and percentage values by referencing with dibromomethane as internal standard. Accordingly, the product *p*-tolyl-λ^{3}-iodanediyl bis(2,2,2-trifluoroacetate) **11b** was formed in 76% NMR-conversion (see Figure S19). Resonances at a chemical shift (referenced against the trifluoroacetic acid residual solvent peak of 11.50 pm) of 8.08 ppm and 6.83 ppm were identified to originate from the starting material and the product, respectively.

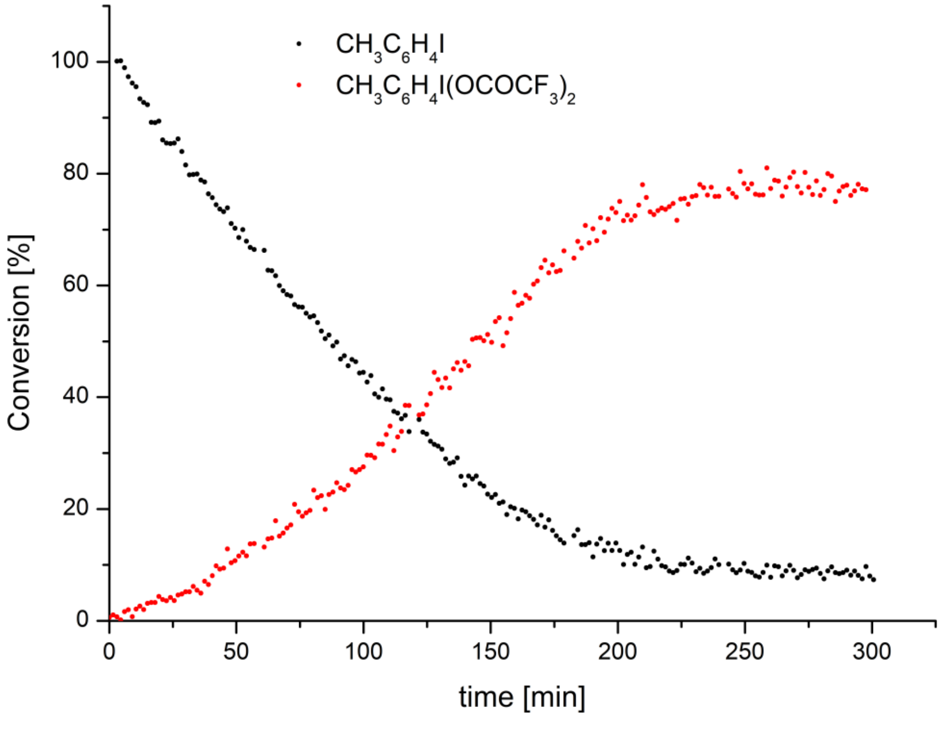

Figure S19. Reaction monitoring for the formation of *p*-tolyl-λ^{3}-iodanediyl bis(2,2,2-trifluoroacetate) **11b**.

Gas-Chromatography Headspace Analysis

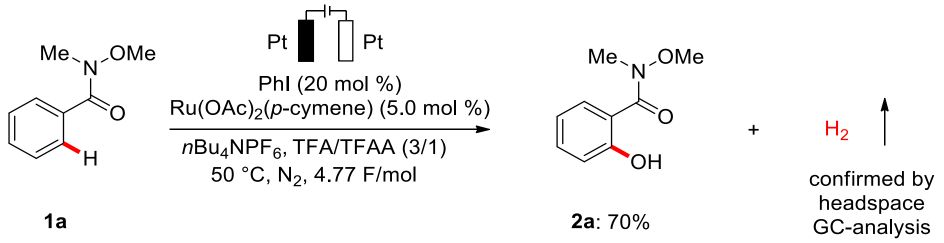

In a Schlenk tube equipped with platinum electrodes (10 mm x 15 mm x 0.125 mm), **1a** (82.5 mg, 0.50 mmol), PhI (20.4 mg, 20 mol %), Ru(OAc)$_2$(*p*-cymene) (8.8 mg, 5.0 mol %) and *n*Bu$_4$NPF$_6$ (194 mg, 1.0 equiv) were added in degassed TFA/TFAA (3:1, 3.0 mL) under nitrogen atmosphere. Electrocatalysis was performed at 50 ℃ with a constant current of 4 mA maintained for 16 h. After cooling to ambient temperature, 1.0 mL of the gas-phase above the reaction mixture was analyzed by GC. Next, a saturated aqueous NaHCO$_3$ (25 mL) was added and the reaction mixture was extracted with EtOAc (3 × 15 mL). The combined organic layers were washed with brine (25 mL), dried over Na$_2$SO$_4$, filtered and concentrated *in vacuo*. The crude product was purified by column chromatography on silica gel (*n*hexane/EtOAc: 5/1) yielding **2a** (63.4 mg, 70%) as yellow oil.

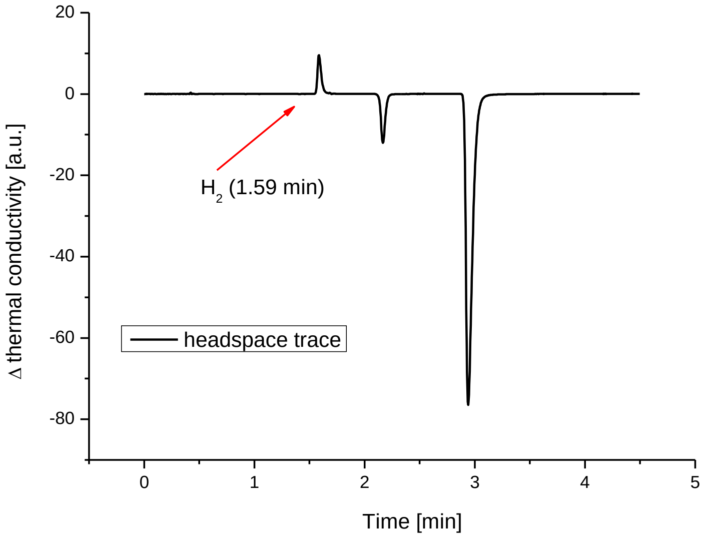

Plausible Catalytic Cycle

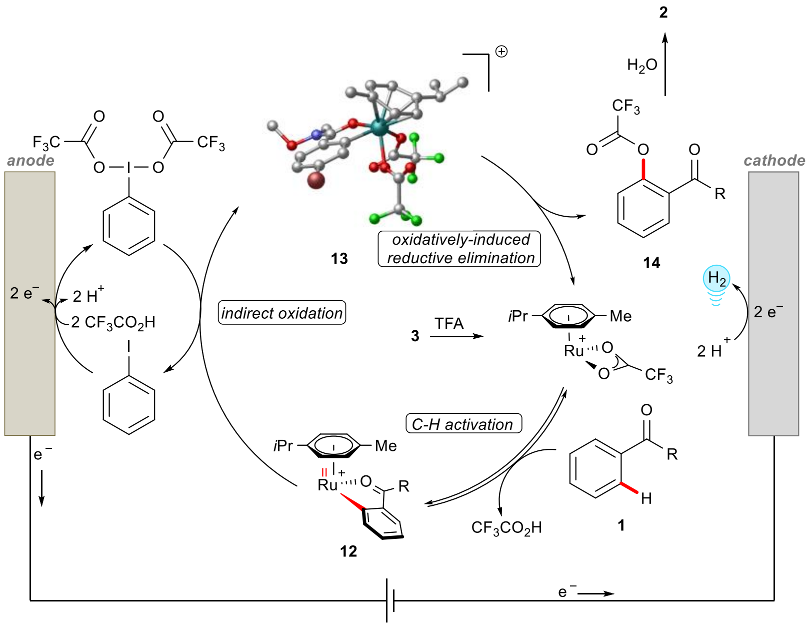

Scheme S2. Catalytic cycle involving Ru(II)/(IV) manifold.

Cost of Goods Analysis

The cost of PIFA, iodobenzene, and electrolyte are summarized below (Table S3). Cost for catalyst, electricity, solvent and starting material are not considered. The presented prices per mmol were calculated, based on the largest quantities available (reported in parenthesis). The price of electricity was calculated based on the industrial electricity price in Germany in March 2019 (https://www.globalpetrolprices.com/Germany/electricity_prices/, visited on the 24.11.2019)

Chemical oxidant reaction

PIFA (abcr GmbH, 1.0 Kg, 989.28 €)	Price: 0.43 €/mmol
	Price per gram of **2a**: 2.84 €/g

Electrochemical reaction

PhI (FluoroChem, 1.0 Kg, 202.95 €)	Price: 0.04 €/mmol
nBu_4NPF_6 (ChemImpex, 1.0 Kg, 307.87€)	Price: 0.12 €/mmol
Electricity (GlobalPetrolPrices.com, 1.0 kWh, 0.191€)	Price: 0.0005 €/g
	Price per gram of **2a**: 0.88 €/g

Electrochemical reaction without electrolyte

PhI (FluoroChem, 1.0 Kg, 202.95 €)	Price: 0.04 €/mmol
Electricity (GlobalPetrolPrices.com, 1.0 kWh, 0.191€)	Price: 0.0005 €/g
	Price per gram of **2a**: 0.08 €/g

Table S3. Comparison of the prices of different oxidation methods.

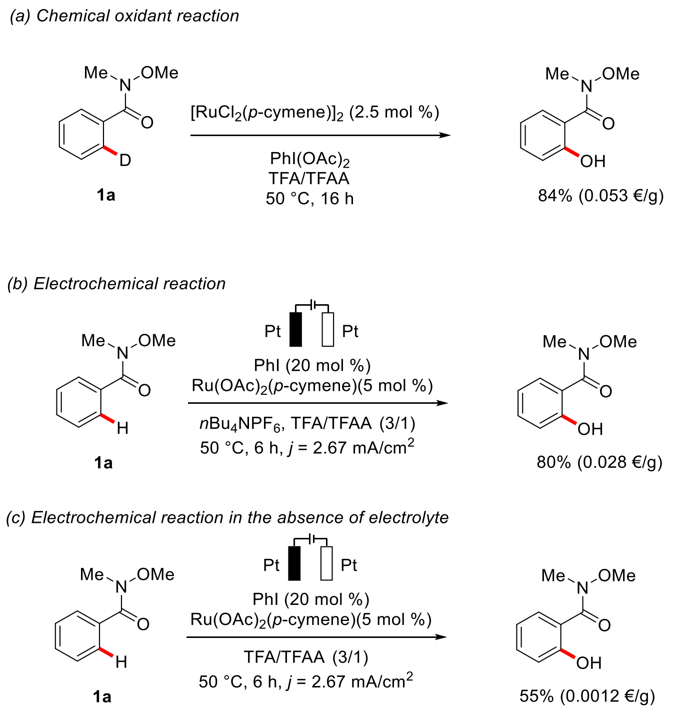

Scheme S3. Comparison of yield and price per gram of the chemical oxidant and electrochemical reactions.

Identification of the Trifluoroacetate Product after C–H Acyloxylation

In order to confirm the formation of the trifluoroacetate intermediate, we performed the following two reactions and analyzed their crude reaction mixture by ^{1}H-NMR spectroscopy. As expected, we could observe the desired trifluoroacetate product after ruthenaelectro-catalyzed C–H acyloxylation (Figure S20). However, attempts to isolate the pure form of the desired product was failed due to the sensitive to moisture.

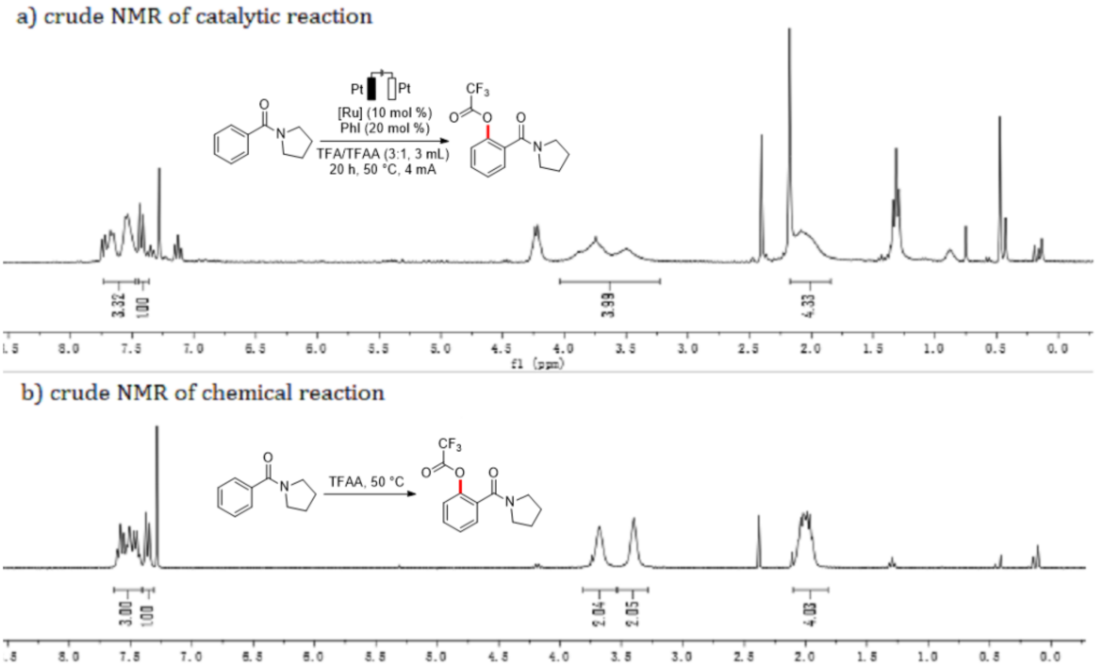

Figure S20. Crude ^{1}H- NMR of electrochemical and chemical oxidant reaction.

The following GC-MS analysis of these two crude reaction mixtures also gave strong support for the generation of the trifluoroacetate product (Fig. S21).

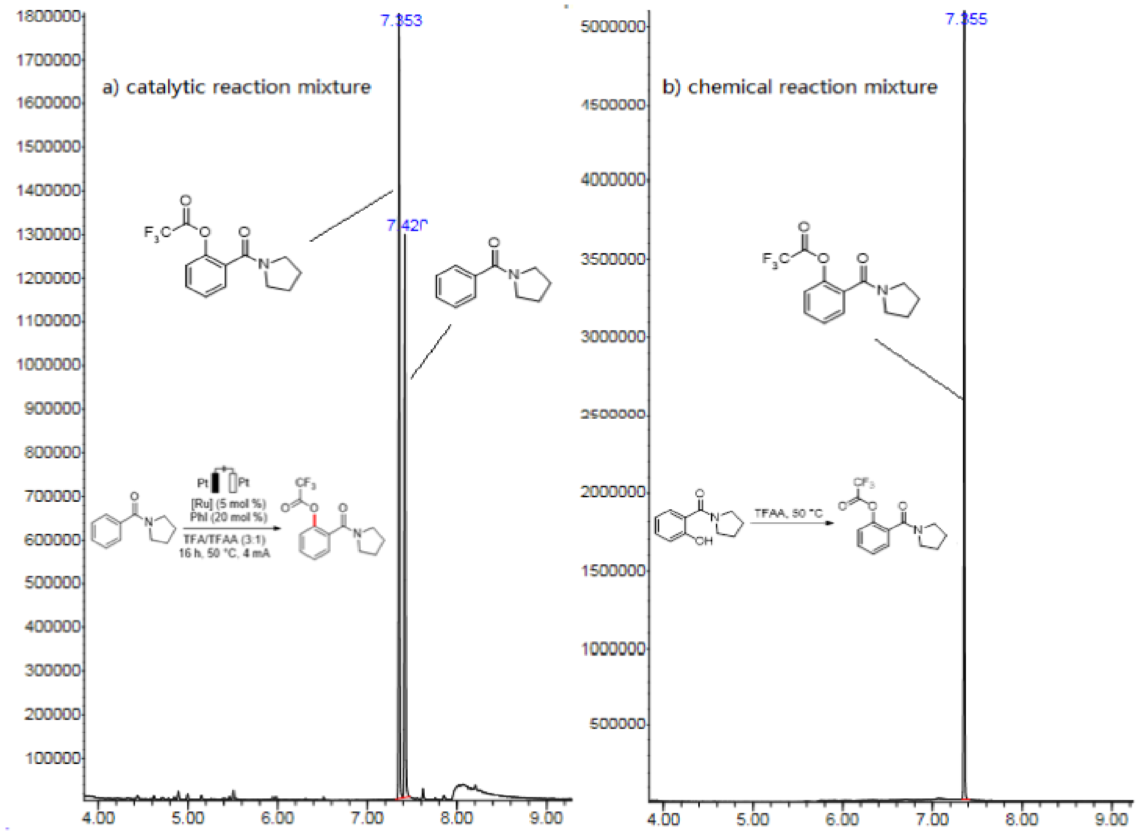

Figure S21. GC-MS data of the reaction mixtures.

IR Spectroscopic Analysis of the Carbon Anode

IR spectroscopic analysis has been carried out on the RVC anode. Two parallel reactions were conducted in undivided cells with RVC anodes (25 mm x 10 mm x 6 mm) and Pt cathodes (10 mm x 15 mm x 0.125 mm), **1a** (82.5 mg, 0.50 mmol, PhI (20.4 mg, 20 mol %), $Ru(OAc)_2$(*p*-cymene) (8.8 mg, 5.0 mol %) and $n Bu_4NPF_6$ (194 mg, 1.0 equiv) in TFA/TFAA (3:1, 3.0 mL). Reaction **B** was heated at 50 ℃ for 16 h without applying electricity. Reaction **C** was heated at 50 ℃ and a constant current of 4 mA was applied for 16 h. After the reaction the RVC electrodes were carefully washed with EtOAc and dried at 70 ℃ overnight.

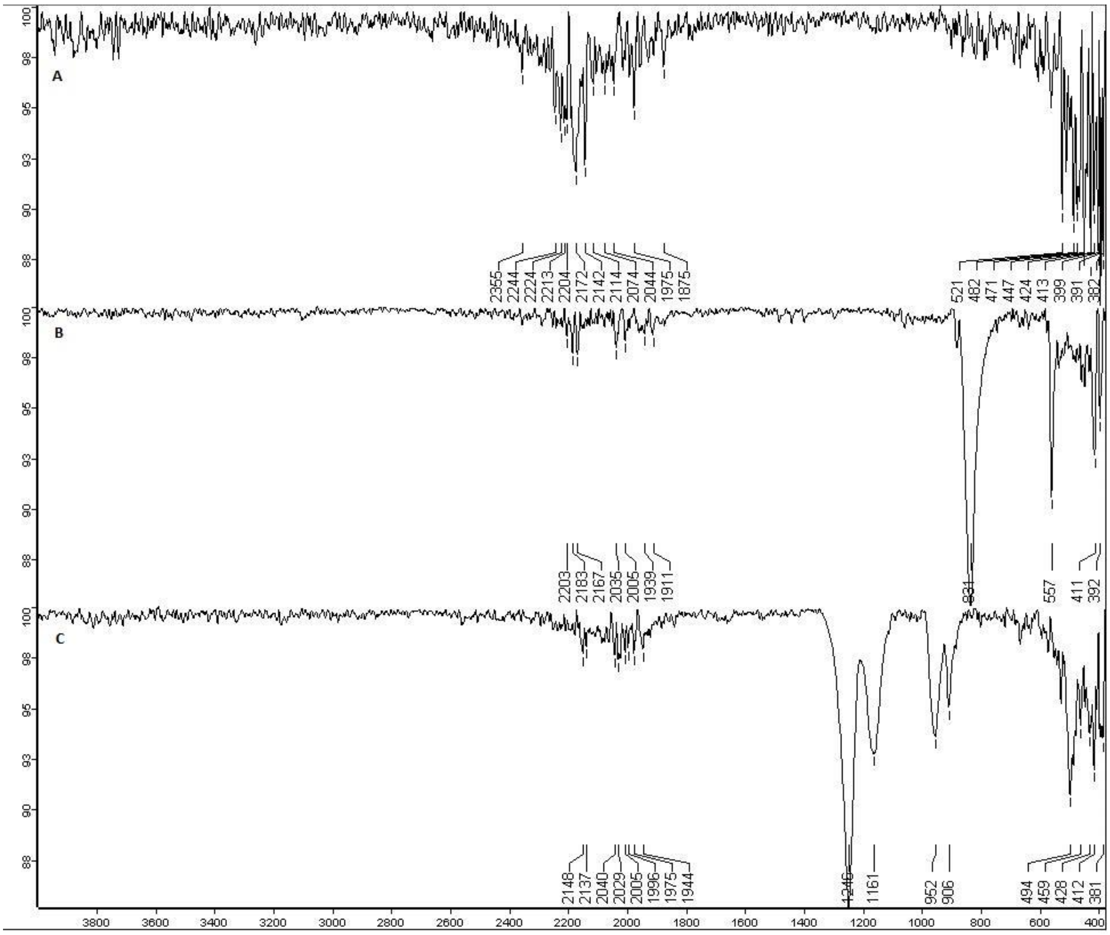

Figure S22. IR spectra of RVC anodes. (A) RVC before reaction; (B) RVC after reaction B without applied current; (C) RVC after reaction C.

Synthesis of the Cyclometalated Complex 10

Synthesis of diaryl mercury complex 15

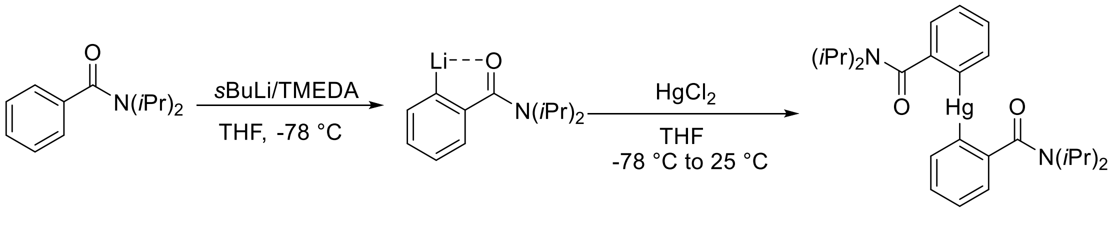

15

Synthesis of complex **15** was performed accordingly to literature procedures reported for similar compounds[13–15] in the following way: a Schlenk tube was loaded with diisopropylbenzamide (500 mg, 2.44 mmol) and dry THF (10 mL) and the solution was cooled to –78 ℃. Subsequently, a solution of *s*BuLi (2.24 mL, 1.2 M in cyclohexane, 2.68 mmol) and TMEDA (365 µL, 2.44 mmol) was prepared, which was then added dropwise to the diisopropylbenzamide solution at –78 ℃. The resulted solution was stirred at –78 ℃ for 5 hours to form the lithiated amide compound. Subsequently, the lithiated amide solution was added dropwise to a cold (–78 ℃) solution of $HgCl_2$ (331 mg, 1.22 mmol) in THF (10 mL). The resulted mixture was stirred at –78 ℃ for two hours followed by an additional two hours of stirring at room temperature. The resulted mixture was then dried under reduced pressure and the residue was extracted with CH_2Cl_2. The filtrate was then dried under reduced pressure to give a yellow, oily substance. Addition of hexane followed by sonication resulted in a white solid which was isolated by filtration. The solid was then washed with pentane and dried under reduced pressure to give complex **15** (464 mg, 62%) as a white air stable solid. Crystals of **15** suitable for an X-ray crystallographic analysis, were grown through slow evaporation of a CH_2Cl_2 solution. **^{1}H-NMR** (400 MHz, CD_2Cl_2) δ = 7.58–7.36 (m, 4H), 7.35–7.19 (m, 4H), 3.78 (s, 4H), 1.37 (s, 24H). **^{13}C-NMR** (100 MHz, CD_2Cl_2) δ = 174.4 (C_q), 168.5 (CH), 147.2 (C_q), 138.4 (CH), 129.0 (CH), 127.5 (CH), 125.5 (C_q), 21.2 (CH_3). **MS** (ESI) *m/z* (relative intensity): 1241.5 (100) $[2M+Na]^+$, 633.2 (90) $[M+Na]^+$, 611.3 (70) $[M+H]^+$. **HR-MS** (ESI) *m/z*

calc. for $C_{26}H_{36}HgN_2O_2^+$ $[M+Na]^+$ 633.2381, found 633.2380.

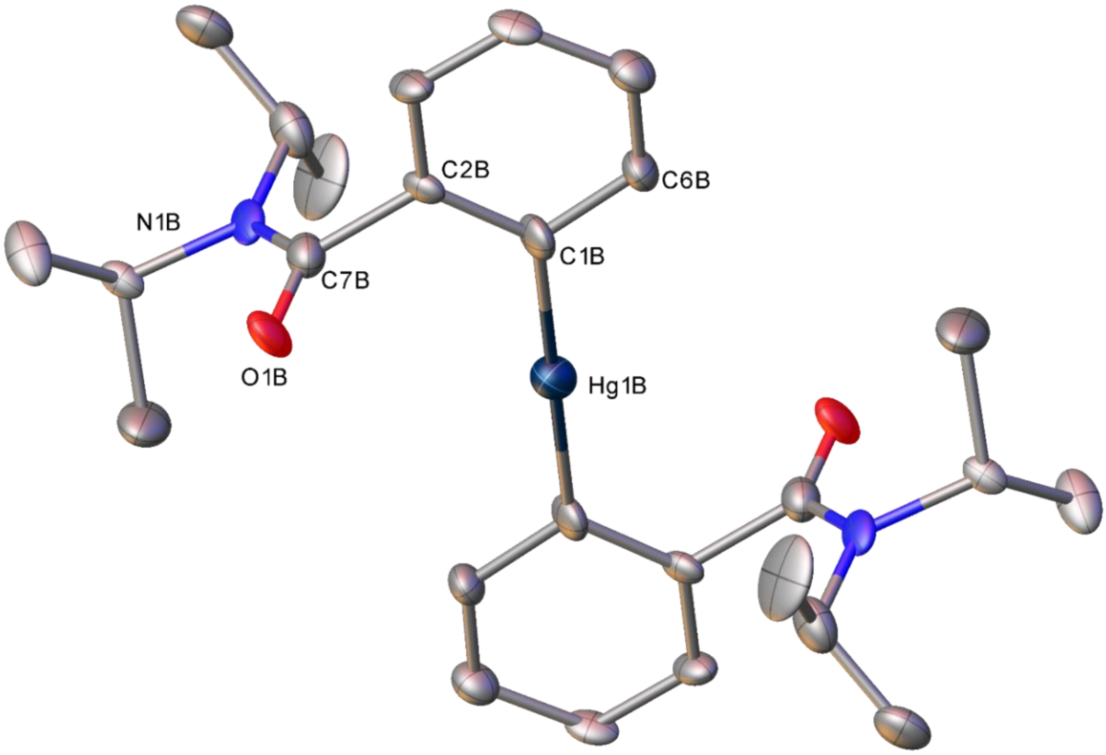

Figure S23. Molecular structure of complex **15**. Thermal ellipsoids are drawn at the 50% probability level and H atoms as well as disorder are omitted for clarity.

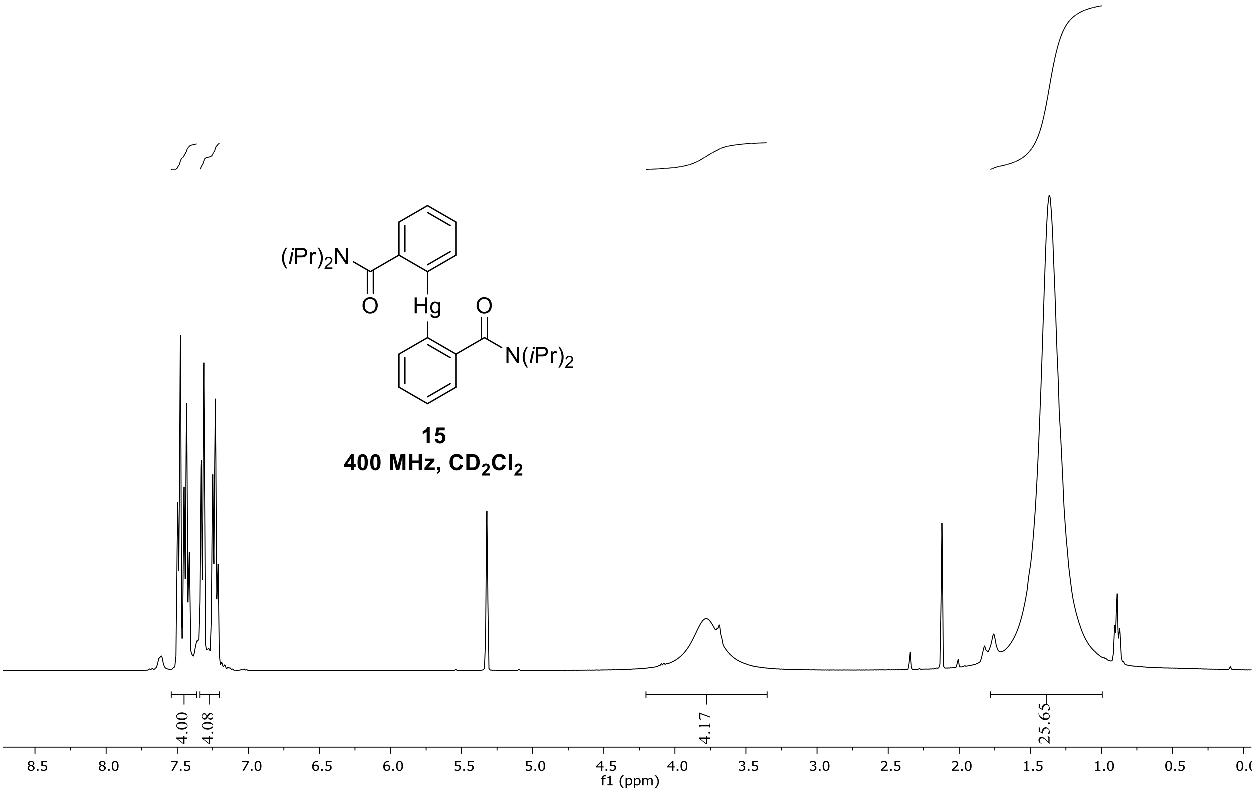

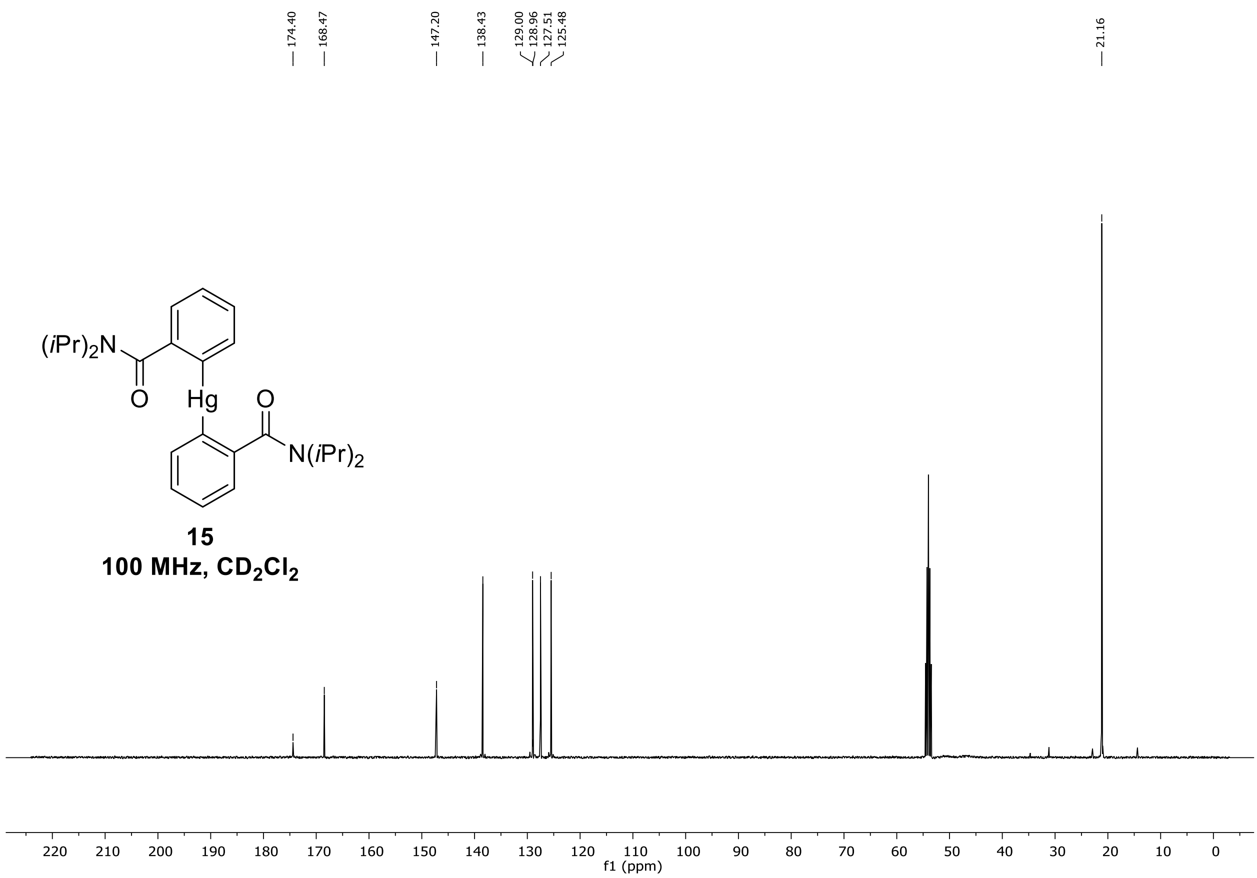

Synthesis of Ruthenacycle 10

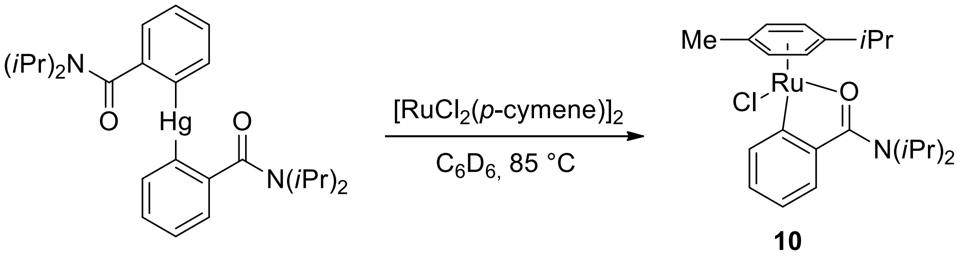

The synthesis of **10** was based on the work by Abbenhuis *et al.*[16] In an argon filled glove box, an NMR tube was loaded with [RuCl$_2$(*p*-cymene)]$_2$ (15.7 mg, 0.026 mmol), complex **15** (15.6 mg, 0.026 mmol) and C_6D_6 (0.6 mL). The mixture was heated at 90 °C for 12 hours and after standing for seven days at room temperature, dark red crystals of starting material [RuCl$_2$(*p*-cymene)]$_2$ formed (according to an X-ray crystallographic analysis). After filtration of the solids, the solution was dried under

reduced pressure resulting in the formation of an orange oil which was then dissolved in CH_2Cl_2 (0.5 mL). After diffusion with pentane for three days dark red crystals formed which did not correspond to the target complex according to an ^{1}H-NMR spectroscopic analysis. Therefore the solution was decanted and stored at -30 °C. After a maximum time of two months bright red crystals of **10** formed (5.5 mg, 23%) which were found to be suitable for an X-ray crystallographic analysis. **^{1}H-NMR** (300 MHz, CD_2Cl_2) δ = 8.23 (dd, J = 7.6, 1.3 Hz, 1H), 7.34 (dd, J = 7.9, 1.3 Hz, 1H), 7.19 (td, J = 7.4, 1.3 Hz, 1H), 6.92 (ddd, J = 8.4, 7.2, 1.3 Hz, 1H), 5.37 (d, J = 5.8 Hz, 1H), 5.08 (d, J = 5.7 Hz, 1H), 4.18 (sbr, $\nu_{1/2}$ = 175 Hz, 2H), 2.56 (sept., J = 6.9 Hz, 1H), 2.02 (s, 3H), 1.39 (d, J = 6.8 Hz, 12H), 1.09 (d, J = 6.9 Hz, 6H). **^{1}H-NMR** (400 MHz, CD_6D_6) δ = 8.37 (dd, J = 7.6, 1.2 Hz, 1H), 7.40–7.22 (m, 2H), 6.96 (td, J = 7.5, 1.3 Hz, 1H), 5.09 (dd, J = 5.7, 1.1 Hz, 1H), 5.01 (dd, J = 5.9, 1.1 Hz, 1H), 4.94 (dd, J = 5.8, 1.1 Hz, 1H), 4.76 (dd, J = 5.8, 1.1 Hz, 1H), 3.87 (sbr, $\nu_{1/2}$ = 321 Hz, 2H), 2.55 (h, J = 6.9 Hz, 1H), 1.84 (s, 3H), 1.27 (sbr, $\nu_{1/2}$ = 125 Hz, 6H), 1.09 (sbr, $\nu_{1/2}$ = 63 Hz, 6H), 1.00 (t, J = 6.7 Hz, 6H). **^{13}C-NMR** (100 MHz, CD_2Cl_2) δ = 185.2 (C_q), 180.4 (C_q), 140.3 (C_q), 139.4 (CH), 129.9 (CH), 127.4 (CH), 121.6 (CH), 98.9 (C_q), 96.0 (C_q), 87.0 (CH), 86.6 (CH), 83.5 (CH), 82.1 (CH), 31.5 (CH), 22.9 (CH_3), 22.3 (CH_3), 21.5 (CH_3), 21.4 (CH_3), 19.0 (CH_3).

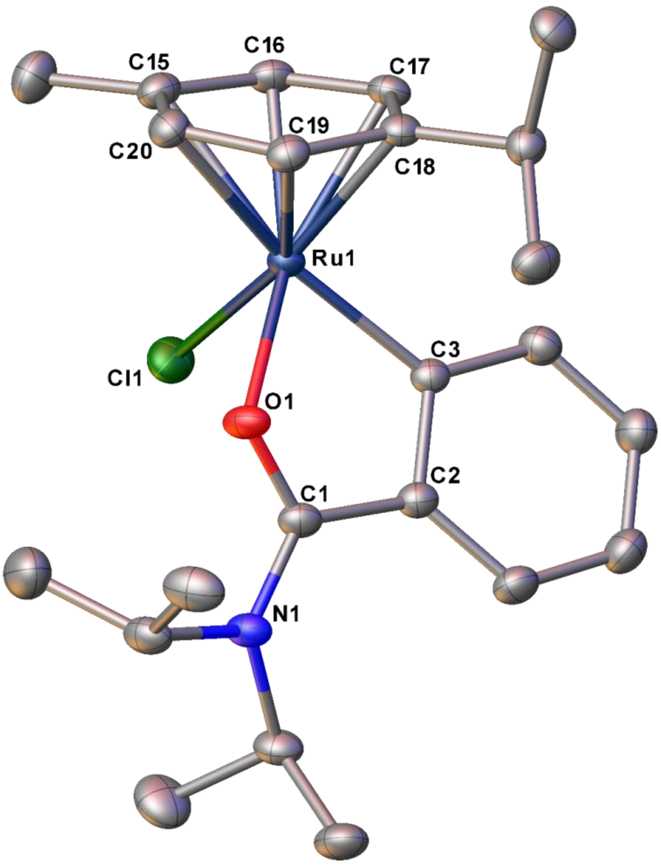

Figure S24. Molecular structure of complex **10**. Thermal ellipsoids are drawn at the 50% probability level and H atoms as well as disorder are omitted for clarity.

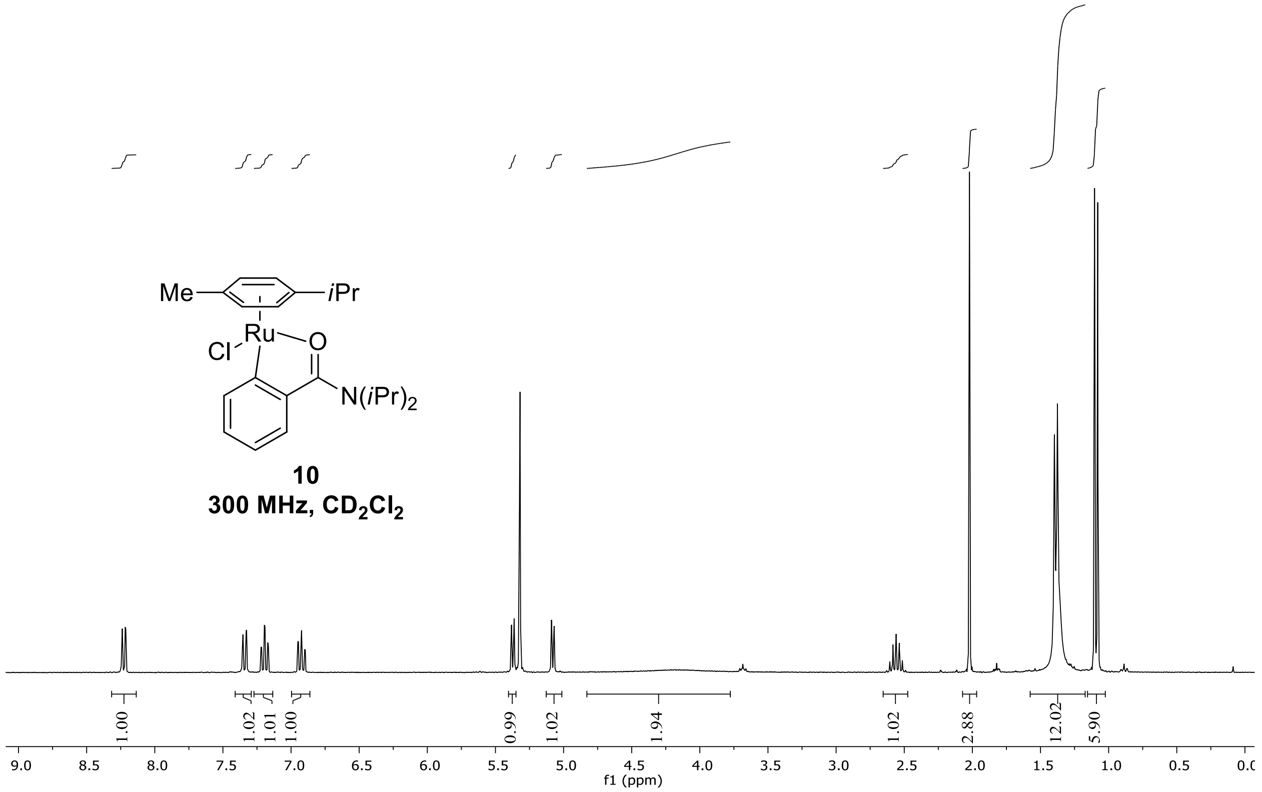
Me
iPr
Ru
Cl
O
N(iPr)2
10
300 MHz, CD2Cl2
f1 (ppm)

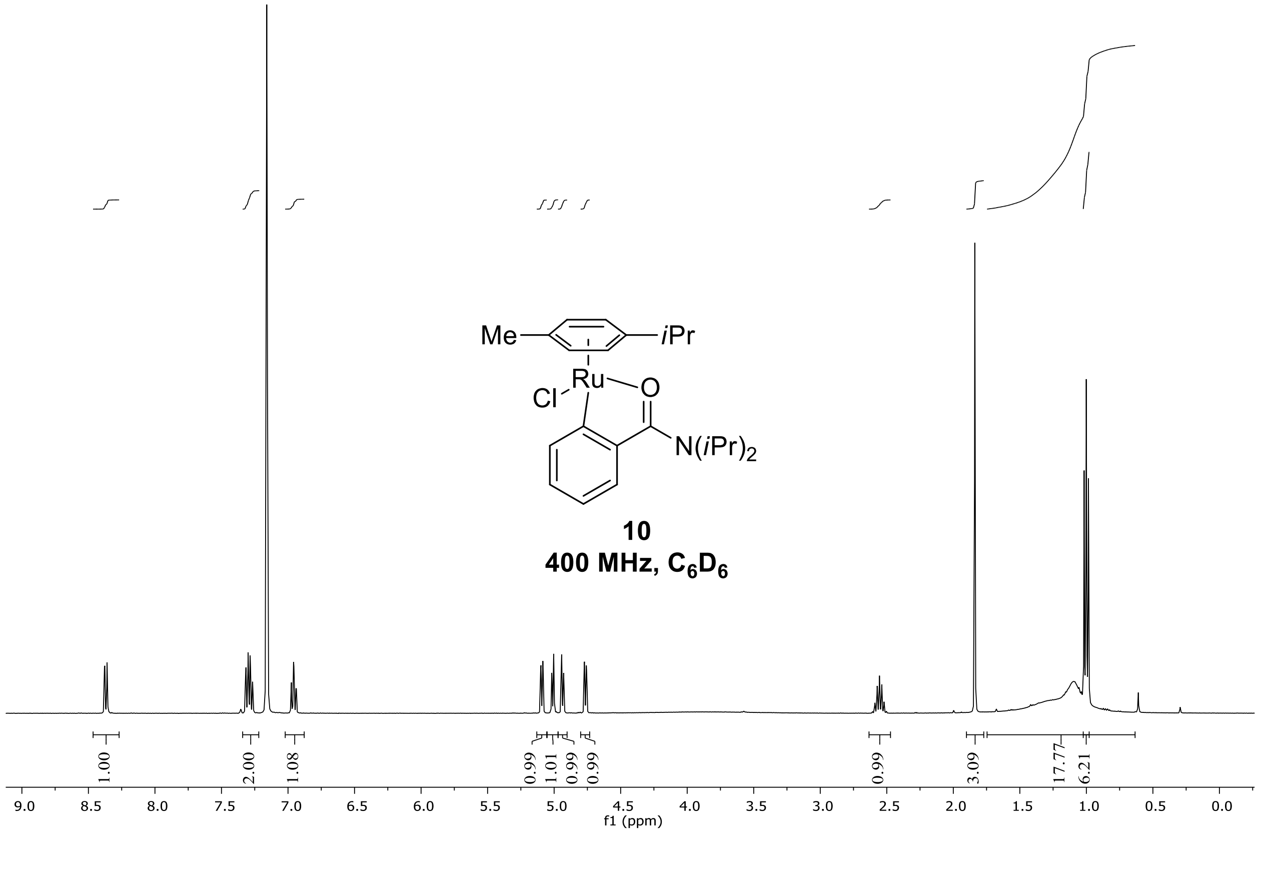
Me
iPr
Ru
Cl
O
N(iPr)2
10
400 MHz, C6D6
f1 (ppm)

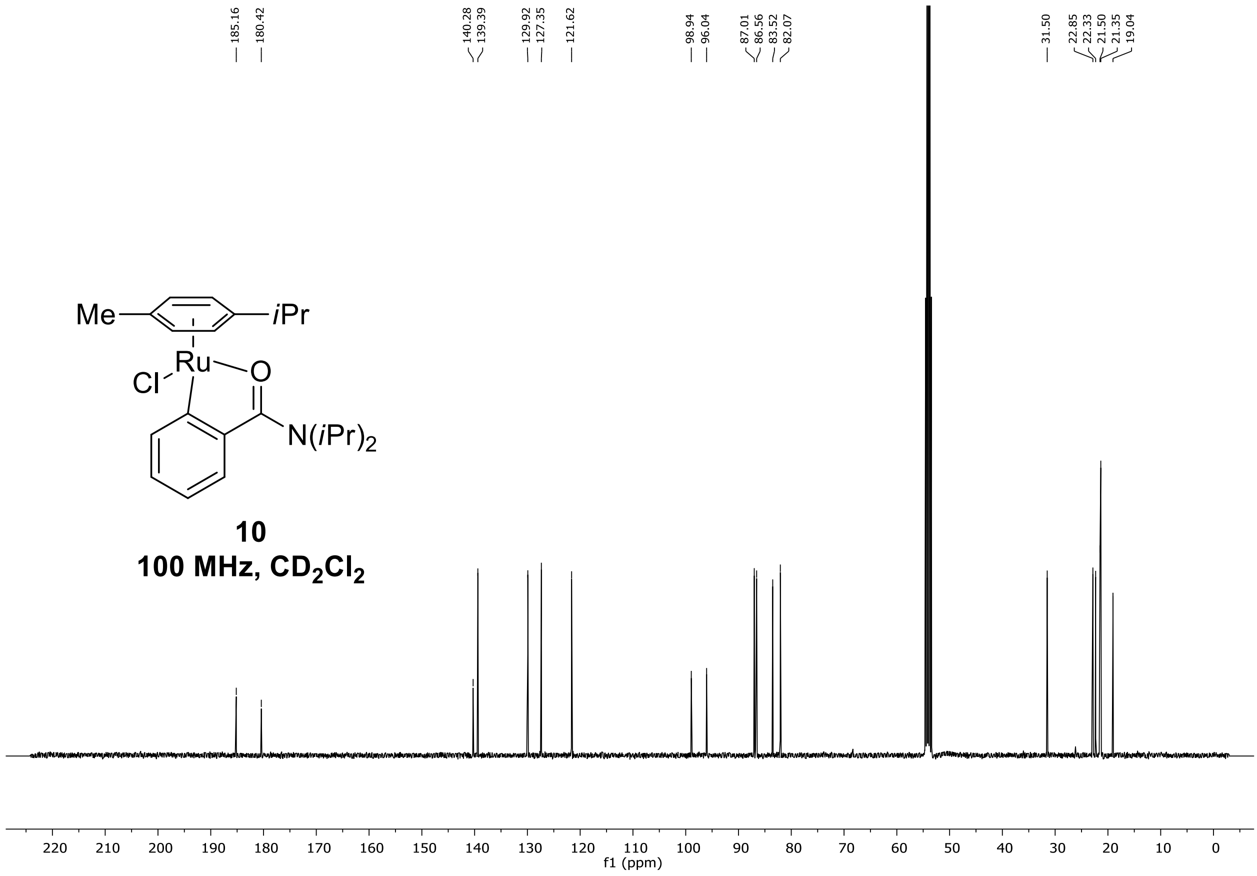

10
100 MHz, CD_2Cl_2

Crystallographic data

X-ray diffraction experiments for all of the compounds were carried out at 100(2) K on a Bruker D8 Venture four-circle-diffractometer from Bruker AXS GmbH equipped with a Photon II detector purchased from Bruker AXS GmbH and using microfocus IµS Cu/Mo radiation from Incoatec GmbH with HELIOS mirror optics and single-hole collimator from Bruker AXS GmbH. Intensities were integrated[17] and absorption corrections based on equivalent reflections were applied using SADABS.[18] The structures were all solved using SHELXT[19] and refined against all F2 in SHELXL[20-21] using Olex 2.[22] All of the non-hydrogen atoms were refined anisotropically while the hydrogen atoms were located geometrically and refined using a riding model. Crystal structure and refinement data are given in Table S4. Crystallographic data for the compounds have been deposited with the Cambridge Crystallographic Data Centre as supplementary publication CCDC (**15**: 1964169; **10**: 1964170). Copies of the data can be obtained free of charge on application to CCDC, 12 Union Road, Cambridge CB2 1EZ, UK (fax(+44) 1223 336033, e-mail:

deposit@ccdc.cam.ac.uk).

Table S1. Crystal data and structure refinement for complexes **15** and **10**.

**Compound**	**15**	**10**
Empirical formula	$C_{26}H_{36}HgN_2O_2$	$C_{23}H_{32}ClNORu$
Formula weight	609.16	475.01
Temperature/K	100.01	150.0
Crystal system	monoclinic	triclinic
Space group	$P2_1/c$	P-1
a/Å	11.5115(7)	9.9914(6)
b/Å	7.4063(5)	11.6889(7)
c/Å	14.6355(8)	20.0716(9)
α/ °	90	76.096(2)
β/ °	100.395(2)	85.803(2)
γ/ °	90	76.590(2)
Volume/Å^3	1227.31(13)	2213.0(2)
Z	2	4
ρ_{calc}g/cm^3	1.648	1.426
μ/mm^{-1}	6.294	0.841
F(000)	604.0	984.0
Crystal size/mm^3	0.207 × 0.155 × 0.052	0.507 × 0.352 × 0.234
Radiation	MoKα (λ = 0.71073)	MoKα (λ = 0.71073)
2θ range for data collection/°	5.66 to 59.258	4.65 to 61.222
Index ranges	$-15 \le h \le 16, -10 \le k \le 10, -20 \le l \le 20$	$-14 \le h \le 14, -16 \le k \le 16, -26 \le l \le 28$
Reflections collected	38352	85267
R_{int} / R_{sigma}	3442 [R_{int} = 0.0338, R_{sigma} = 0.0161]	13535 [R_{int} = 0.0238, R_{sigma} = 0.0160]
Data/restraints/parameters	3442/111/291	13535/0/501
Goodness-of-fit on F^2	1.103	1.072
Final R indexes [I>=2σ (I)]	R_1 = 0.0214, wR_2 = 0.0521	R_1 = 0.0218, wR_2 = 0.0510
Final R indexes [all data]	R_1 = 0.0240, wR_2 = 0.0536	R_1 = 0.0244, wR_2 = 0.0525
Largest diff. peak/hole / e Å^{-3}	0.96/-1.20	0.68/-0.64

Computational Studies

All the calculations were carried out using DFT with the Gaussian 16, Revision A.03 package.[23] Geometry optimization of all the stationary points were carried out at the TPSS[24] level of theory in combination with D3 dispersion corrections with Becke-Johnson damping scheme (D3BJ).[25] All atoms were described with def2-TZVP[26] basis set. Default ECP of def2-TZVP was used for both Ru and iodine atoms. Analytical frequency calculations were carried out at the same level of theory in order to identify all the intermediates (no imaginary frequencies) and to provide thermal and non-thermal corrections to the free energy in gas-phase at 298.15 K and 1 atm.

The electronic energy was then refined through PBE0[27] single-point calculations on the optimized geometries including dispersion corrections (D3BJ) using def2-QZVP basis set for all atoms.[26] Default ECP of def2-QZVP was used for both Ru and iodine atoms. Solvent effects were taken into consideration in the single point calculations. These were implicitly included through the use of the CPCM[28] model with a dielectric constant of ε =35.688, which corresponds to acetonitrile.

Unless and otherwise stated, all the reported energies are Gibbs free energies in kcal mol^{-1}, which were calculated by adding the gas-phase thermal and non-thermal corrections at 298.15 K to the single-point energies.

Oxidation potentials were calculated using following equation[29]

$$E_{1/2}^{\mathrm{o,calc}} = -\frac{\Delta G_{1/2}^{o}}{n_e F} - E_{1/2}^{\mathrm{o,SHE}} - E_{1/2}^{\mathrm{o,SCE}} \qquad (1)$$

Where

$$\Delta G_{1/2}^{o} = G_{298.15}(reduced) - G_{298.15}(oxidized) \qquad (2)$$

In the equation 1, n_e is the number of electrons transferred during the oxidation process, F is the Faraday constant with a value of 23.061 kcal mol^{-1} V^{-1}, $E_{1/2}^{\mathrm{o,SHE}}$ is

the absolute value of the standard hydrogen electrode potential (4.281 V), and $E_{1/2}^{\mathrm{o,SCE}}$ is the standard potential of the saturated calomel electrode relative to SHE in sat. KCl (0.244 V).

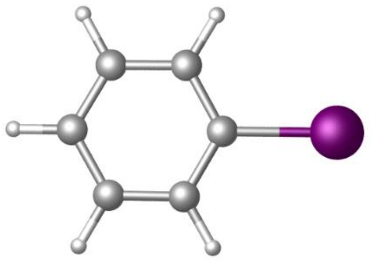

Figure S25. Computed structure for PhI molecule.

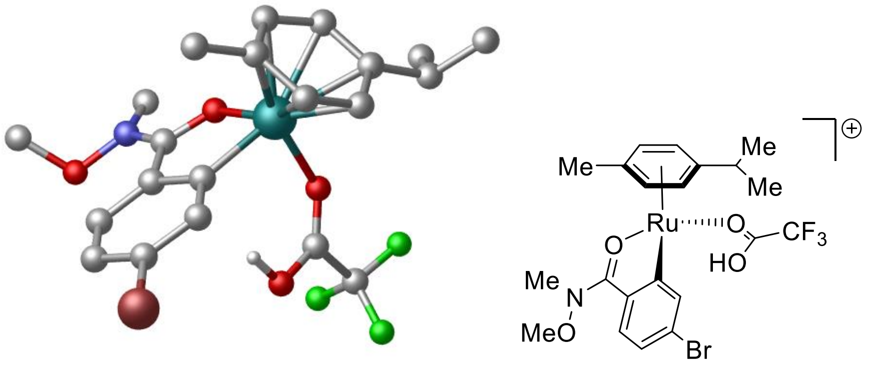

Figure S26. Computed structure for ruthenium complex **12**. The H atoms were omitted for clarity.

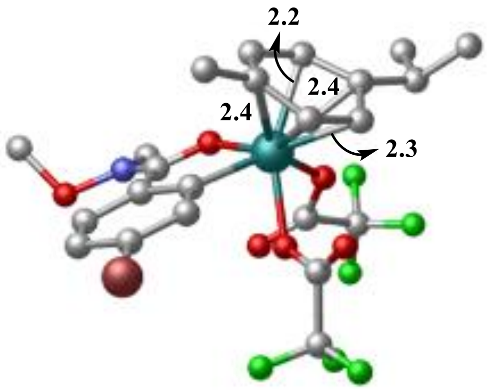

Figure S27. Computed structure for ruthenium complex **13** at

TPSS-D3(BJ)/def2-TZVP level of theory. The H atoms were omitted for clarity.

Table S5. Calculated oxidation potentials.

Redox pair	Potential (V *vs.* SCE)
**PhI → PhI^{+} + $1e^{-}$**	2.06
**12 → 12^{+} + $1e^{-}$**	1.40
**12 → 12^{2+} + $2e^{-}$**	2.25

Table S6. Calculated electronic energies at the PBE0-D3(BJ)/def2-QZVP+CPCM (acetonitrile) //TPSS-D3(BJ)/def2-TZVP level of theory and total Gibbs free energies with dispersion corrections for all structures (in Hartree)

Structure	Electronic Energy	Total Gibbs Free Energy
**PhI**	-529.197345	-529.140690
**PhI^{+}**	-528.953760	-528.898384
**12**	-4137.668145	-4137.324852
**12^{+}**	-4137.449772	-4137.106909
**12^{2+}**	-4137.171658	-4136.826686

Cartesian coordinates of the optimized structures

PhI

Lowest frequency = 148.010 cm^{-1}

Charge=0, Multiplicity=1

C	-0.801651	0.998576	0.000047
C	0.594125	0.989413	0.000557
C	1.277797	2.205155	0.000022

C	0.594224	3.420960	-0.001001
C	-0.801535	3.411921	-0.001499
C	-1.501393	2.205274	-0.000977
H	-1.338971	0.054424	0.000465
H	1.138748	0.051537	0.001343
H	1.139022	4.358735	-0.001411
H	-1.338842	4.356079	-0.002289
H	-2.587146	2.205366	-0.001371
I	3.390581	2.205119	0.000780

PhI^+

Lowest frequency = 120.330 cm^{-1}

Charge=1, Multiplicity=2

C	-0.788934	0.974599	0.000067
C	0.590463	0.961734	0.000570
C	1.279230	2.205156	0.000021
C	0.590571	3.448640	-0.001022
C	-0.788826	3.435895	-0.001508
C	-1.482570	2.205277	-0.000969
H	-1.342769	0.041480	0.000466
H	1.145189	0.030031	0.001365
H	1.145379	4.380294	-0.001419
H	-1.342580	4.369062	-0.002304
H	-2.568723	2.205324	-0.001359
I	3.328529	2.205068	0.000757

12

Lowest frequency = 8.430 cm^{-1}

Charge=1, Multiplicity=1

Ru	-0.897953	-0.229032	-0.838712

C	-0.769843	-2.199880	-1.723383
C	-0.480018	-1.216977	-2.728355
C	-1.443197	-0.185293	-2.909591
C	-2.700487	-0.171558	-2.214152
C	-2.977562	-1.133024	-1.234899
C	-1.963818	-2.125266	-0.971220
O	-1.474872	0.072807	1.206573
C	-0.903195	-0.091087	2.284094
O	0.327015	-0.483003	2.484532
C	-1.664850	0.202657	3.602653
C	1.634446	1.210384	-0.275558
C	2.987214	1.409664	0.046078
C	1.089693	-0.109329	-0.350551
C	3.826741	0.326079	0.281525
H	3.389503	2.408518	0.135647
C	1.952251	-1.186086	-0.089477
C	3.302944	-0.964219	0.207799
H	4.870764	0.482010	0.526876
H	1.587951	-2.207988	-0.118716
C	0.615592	2.230077	-0.537094
H	-1.205264	0.634991	-3.580037
H	-2.115268	-2.824101	-0.153852
H	-3.386232	0.650349	-2.381758
H	-0.037071	-2.966093	-1.493270
C	0.795889	-1.255674	-3.518651
H	1.620291	-1.650115	-2.919258
H	0.667058	-1.907249	-4.390944
H	1.066570	-0.258844	-3.875627
C	-4.229807	-1.124821	-0.386272
H	-3.926319	-1.445577	0.619267

C	-5.215341	-2.176342	-0.940641
H	-4.756628	-3.168669	-0.996264
H	-6.093111	-2.240770	-0.291033
H	-5.549344	-1.896549	-1.945159
C	-4.886967	0.253809	-0.268394
H	-5.717301	0.204860	0.441407
H	-4.175587	1.004895	0.089061
H	-5.298282	0.586025	-1.228005
Br	4.433074	-2.452676	0.522414
O	-0.575225	1.820803	-0.788141
N	0.793926	3.564720	-0.561911
C	-0.306570	4.526136	-0.617865
H	-0.556695	4.859711	0.393807
H	0.003781	5.380570	-1.222138
H	-1.162631	4.034780	-1.076552
O	2.004530	4.100947	-0.104209
C	2.761122	4.653589	-1.215162
H	3.026078	3.863902	-1.924795
H	2.194552	5.445784	-1.712752
H	3.653848	5.072273	-0.749015
H	0.789063	-0.596550	1.602272
F	-1.247235	1.390369	4.088496
F	-1.425025	-0.750504	4.514959
F	-2.982395	0.268182	3.370737

12^{+}

Lowest frequency = 10.940 cm^{-1}

Charge=2, Multiplicity=2

Ru	-0.990510	-0.500109	-0.418406
C	-1.008114	-2.731988	-0.464850

C	-0.607188	-2.352507	-1.777422
C	-1.384055	-1.348408	-2.411206
C	-2.637211	-0.918137	-1.862400
C	-3.155541	-1.467312	-0.663799
C	-2.243780	-2.274871	0.081724
O	-1.033801	0.204662	1.538337
C	-0.274244	0.463339	2.483293
O	1.002188	0.225574	2.552780
C	-0.856022	1.184986	3.734001
C	1.398320	1.155954	-0.866374
C	2.755025	1.516747	-0.823359
C	1.014720	-0.169078	-0.502900
C	3.722965	0.590466	-0.446203
H	3.055558	2.527742	-1.060509
C	1.989162	-1.087773	-0.105748
C	3.342963	-0.707228	-0.083359
H	4.768779	0.875938	-0.422029
H	1.726892	-2.100050	0.182490
C	0.241911	1.995595	-1.164133
H	-1.044235	-0.920877	-3.348860
H	-2.513801	-2.583130	1.087987
H	-3.192019	-0.148655	-2.388026
H	-0.387890	-3.406915	0.116421
C	0.610146	-2.919266	-2.435375
H	1.306684	-3.355234	-1.717885
H	0.275358	-3.724418	-3.103753
H	1.125957	-2.175544	-3.046564
C	-4.513196	-1.126776	-0.115493
H	-4.449158	-1.201895	0.977114
C	-5.500808	-2.222908	-0.605013

H	-5.178249	-3.223296	-0.302969
H	-6.482594	-2.028515	-0.165151
H	-5.596308	-2.197090	-1.694833
C	-5.005047	0.276345	-0.494329
H	-5.944448	0.479480	0.025178
H	-4.284574	1.049013	-0.205007
H	-5.204562	0.359513	-1.567862
Br	4.642062	-1.958208	0.442549
O	-0.923119	1.419481	-1.045953
N	0.235472	3.272929	-1.534480
C	-0.972641	4.099389	-1.661954
H	-0.814992	5.006024	-1.074103
H	-1.147785	4.351627	-2.710449
H	-1.815245	3.530565	-1.272724
O	1.451429	3.949871	-1.564105
C	1.755082	4.453750	-2.905097
H	1.794362	3.625057	-3.617170
H	1.023136	5.206231	-3.206266
H	2.735200	4.915149	-2.785888
H	1.336710	-0.167005	1.700391
F	-0.715641	2.509655	3.525814
F	-0.201288	0.830234	4.835831
F	-2.154076	0.891139	3.855463

12^{2+}

Lowest frequency = 16.320 cm^{-1}

Charge=3, Multiplicity=1

Ru	-0.486327	-0.563905	-0.231063
C	-0.159945	-2.762406	-0.569289
C	0.258709	-2.144875	-1.786441

C	-0.621042	-1.160022	-2.312875
C	-1.977981	-1.048880	-1.847903
C	-2.486630	-1.895924	-0.832706
C	-1.474461	-2.570150	-0.062131
O	-1.110754	0.048495	1.646147
C	-1.908775	0.683596	2.356947
O	-1.754516	0.890716	3.626569
C	-3.252528	1.265769	1.818649
C	1.769549	1.304536	-0.371032
C	3.066163	1.752752	-0.111572
C	1.450648	-0.091213	-0.147812
C	4.041862	0.852432	0.320643
H	3.321927	2.797740	-0.222698
C	2.439385	-0.988150	0.258216
C	3.724546	-0.510325	0.536982
H	5.056101	1.198770	0.496432
H	2.225590	-2.036026	0.428309
C	0.594625	2.070647	-0.745808
H	-0.297190	-0.530849	-3.136061
H	-1.748019	-3.063726	0.866706
H	-2.625874	-0.316196	-2.318608
H	0.516949	-3.419677	-0.031327
C	1.562880	-2.456705	-2.441001
H	2.292796	-2.883391	-1.751814
H	1.351936	-3.219703	-3.206180
H	1.986563	-1.591533	-2.955769
C	-3.932103	-2.036003	-0.502259
H	-4.048871	-1.976515	0.588531
C	-4.295491	-3.521351	-0.890058
H	-3.683549	-4.249948	-0.352006

H	-5.342763	-3.673026	-0.615520
H	-4.183174	-3.672890	-1.966477
C	-4.867871	-1.054570	-1.209120
H	-5.892056	-1.230777	-0.873359
H	-4.621717	-0.011981	-0.989766
H	-4.852483	-1.204210	-2.293968
Br	5.050955	-1.655044	1.174960
O	-0.528119	1.413819	-0.766115
N	0.545837	3.374346	-1.055786
C	-0.702112	4.099564	-1.339912
H	-0.717338	5.001234	-0.723125
H	-0.753856	4.359933	-2.400600
H	-1.527631	3.438273	-1.081860
O	1.718338	4.089458	-0.952810
C	1.904073	5.110691	-2.001437
H	1.874827	4.633559	-2.984067
H	1.156759	5.899141	-1.900795
H	2.896921	5.499130	-1.777935
H	-0.941401	0.483452	4.001011
F	-3.142931	1.440765	0.484258
F	-3.539936	2.416852	2.398457
F	-4.211684	0.359139	2.059928

13

Lowest frequency = 14.820 cm^{-1}

Charge=1, Multiplicity=1

Ru	-0.544466	-0.135377	-0.815545
C	0.134424	-1.826264	-2.141216
C	0.479452	-0.671530	-2.893517
C	-0.517925	0.330107	-2.994558

C	-1.865876	0.071572	-2.604994
C	-2.264666	-1.206486	-2.123345
C	-1.214264	-2.087149	-1.756750
O	0.037118	-0.906532	1.023484
C	0.397727	-2.141438	1.180114
O	0.515893	-3.035523	0.355564
C	0.753982	-2.393466	2.684114
C	1.549544	1.852373	-0.104101
C	2.819469	2.327974	0.273847
C	1.403415	0.497870	-0.477936
C	3.916618	1.480505	0.267352
H	2.937407	3.353353	0.594571
C	2.490420	-0.364050	-0.443038
C	3.743035	0.138861	-0.086127
H	4.894889	1.845834	0.557342
H	2.382755	-1.422553	-0.646295
C	0.278746	2.551000	-0.092133
H	-0.263614	1.303878	-3.400745
H	-1.420960	-2.998451	-1.207980
H	-2.600177	0.860659	-2.712508
H	0.886191	-2.560311	-1.880445
C	1.836524	-0.481214	-3.492281
H	2.584715	-1.121886	-3.024649
H	1.769597	-0.756528	-4.553623
H	2.161767	0.559838	-3.434547
C	-3.690978	-1.562836	-1.816740
H	-3.698018	-1.918479	-0.777956
C	-4.094594	-2.751019	-2.729114
H	-3.423950	-3.606491	-2.606545
H	-5.106439	-3.068577	-2.464640

H	-4.090445	-2.451168	-3.781737
C	-4.673883	-0.398105	-1.948172
H	-5.671047	-0.732924	-1.652310
H	-4.397689	0.436144	-1.298255
H	-4.738798	-0.044537	-2.983945
Br	5.223300	-1.037663	-0.064364
O	-0.744366	1.880961	-0.510706
N	0.030746	3.799432	0.305945
C	-1.311078	4.339551	0.534933
H	-1.277117	4.896307	1.472827
H	-1.616309	4.992464	-0.287729
H	-1.997206	3.498797	0.622115
O	1.094444	4.590390	0.739409
C	1.368893	5.653911	-0.215045
H	1.649014	5.230088	-1.184495
H	0.504431	6.316316	-0.312292
H	2.205712	6.195478	0.226757
F	1.896332	-1.728467	2.987169
F	0.950784	-3.697052	2.915879
F	-0.222720	-1.954577	3.502250
O	-2.222474	-0.082971	0.331915
C	-2.326577	0.569606	1.470868
O	-1.549142	1.289814	2.050013
C	-3.774045	0.339878	2.032929
F	-3.841539	0.668382	3.323602
F	-4.189844	-0.938246	1.890674
F	-4.640886	1.134903	1.343116

References

[1] F. Yang, L. Ackermann, *Org. Lett.* **2013**, *15*, 718–720.

[2] V. S. Thirunavukkarasu, J. Hubrich, L. Ackermann, *Org. Lett.* **2012**, *14*, 4210–4213.

[3] K. C. Nicolaou, J. Becker, Y. H. Lim, A. Lemire, T. Neubauer, A. Montero, *J. Am. Chem. Soc.* **2009**, *131*, 14812–14826.

[4] Y. Zhao, V. Snieckus, *J. Am. Chem. Soc.* **2014**, *136*, 11224–11227.

[5] V. S. Thirunavukkarasu, L. Ackermann, *Org. Lett.* **2012**, *14*, 6206–6209.

[6] G. Shan, X. Han, Y. Lin, S. Yu, Y. Rao, *Org. Biomol. Chem.* **2013**, *11*, 2318–2322.

[7] T. Kitagawa, A. Miyabo, H. Fujii, T. Okazaki, T. Mori, M. Matsudou, T. Sugie, K. i. Takeuchi, *J. Org. Chem.* **1997**, *62*, 888–892.

[8] D. R. Henton, K. Anderson, M. J. Manning, J. S. Swenton, *J. Org. Chem.* **1980**, *45*, 3422–3433.

[9] K. V. Chuang, R. Navarro, S. E. Reisman, *Chem. Sci.* **2011**, *2*, 1086–1089.

[10] W. Liu, L. Ackermann, *Org. Lett.* **2013**, *15*, 3484–3486.

[11] X. Yang, H. Wang, X. Zhou, X. Li, *Org. Biomol. Chem.* **2016**, *14*, 5233–5237.

[12] C. Amatore, M. Azzabi, P. Calas, A. Jutand, C. Lefrou, Y. Rollin, *J. Electroanal. Chem*. **1990**, *288*, 45–63

[13] J. N. Reed, V. Snieckus, *Tetrahedron Lett.* **1983**, *24*, 3795–3798.

[14] P. Beak, R. A. Brown, *J. Org. Chem.* **1977**, *42*, 1823–1824.

[15] A. F. M. J. Van Der Ploeg, C. E. M. Van Der Kolk, G. Van Koten, *J. Organomet. Chem.***1981**, *212*, 283–290.

[16] H. C. L. Abbenhuis, M. Pfeffer, J. P. Sutter, A. de Cian, J. Fischer, H. L. Ji, J. H. Nelson, *Organometallics* **1993**, *12*, 4464–4472.

[17] Bruker, SAINT+ v8.38A Integration Engine, Data Reduction Software, Bruker Analytical X-ray Instruments Inc., Madison, WI, USA, 2015.

[18] Bruker, SADABS 2016/2, Bruker AXS area detector scaling and absorption correction, Bruker Analytical X-ray Instruments Inc., Madison, Wisconsin, USA, 2016/2.

[19] Sheldrick, G. SHELXT - Integrated space-group and crystal-structure determination. *Acta Cryst. A* **2015**, *71*, 3-8.

[20] Sheldrick, G. Crystal structure refinement with SHELXL. *Acta Cryst. C* **2015**, *71*, 3–8.

[21] Sheldrick, G. A short history of SHELX. *Acta Cryst. A* **2008**, *64*, 112-122.

[22] Dolomanov, O. V., Bourhis, L. J., Gildea, R. J., Howard, J. A. K. & Puschmann, H. OLEX2: a complete structure solution, refinement and analysis program. *J. Appl. Crystallogr.***2009**, 42, 339-34.

[23] Gaussian 16, Revision A.03, M. J. Frisch, G. W. Trucks, H. B. Schlegel, G. E. Scuseria, M. A. Robb, J. R. Cheeseman, G. Scalmani, V. Barone, G. A. Petersson, H. Nakatsuji, X. Li, M. Caricato, A. V. Marenich, J. Bloino, B. G. Janesko, R. Gomperts, B. Mennucci, H. P. Hratchian, J. V. Ortiz, A. F. Izmaylov, J. L. Sonnenberg, D. Williams-Young, F. Ding, F. Lipparini, F. Egidi, J. Goings, B. Peng, A. Petrone, T. Henderson, D. Ranasinghe, V. G. Zakrzewski, J. Gao, N. Rega, G. Zheng, W. Liang, M. Hada, M. Ehara, K. Toyota, R. Fukuda, J. Hasegawa, M. Ishida, T. Nakajima, Y. Honda, O. Kitao, H. Nakai, T. Vreven, K. Throssell, J. A. Montgomery, Jr., J. E. Peralta, F. Ogliaro, M. J. Bearpark, J. J. Heyd, E. N. Brothers, K. N. Kudin, V. N. Staroverov, T. A. Keith, R. Kobayashi, J. Normand, K. Raghavachari, A. P. Rendell, J. C. Burant, S. S. Iyengar, J. Tomasi, M. Cossi, J. M. Millam, M. Klene, C. Adamo, R. Cammi, J. W. Ochterski, R. L. Martin, K. Morokuma, O. Farkas, J. B. Foresman, and D. J. Fox, Gaussian, Inc., Wallingford CT, **2016**.

[24] J. Tao, J. P. Perdew, V. N. Staroverov, G. E. Scuseria, *Phys. Rev. Lett.* **2003**, *91*, 46401.

[25] a) S. Grimme, S. Ehrlich, L. Goerigk, *J. Comput. Chem.* **2011**, *32*, 1456–1465; b) S. Grimme, J. Antony, S. Ehrlich, H. Krieg, *J. Chem. Phys.* **2010**, *132*, 154104–154119.

[26] a) F. Weigend, R. Ahlrichs, *Phys. Chem. Chem. Phys.* **2005**, *7*, 3297–3305; b) A. Peterson, *J. Chem. Phys.* **2003**, *119*, 11113–11123; c) D. Andrae, U. Häußermann, M. Dolg, H. Stoll, H. Preuß, *Theor. Chim. Acta* **1990**, *77*, 123–141.

[27] C. Adamo, V. Barone, *J. Chem. Phys.* **1999**, *110*, 6158–6159.

[28] a) M. Cossi, N. Rega, G. Scalmani, V. Barone, *J. Comp. Chem.* **2003**, *24*, 669–681; b) V. Barone, M. Cossi, *J. Phys. Chem. A* **1998**, *102*, 1995–2001.

[29] H. G. Roth, N. A. Romero, D. A. Nicewicz, *Synlett* **2016**, *27*, 714–723.

NMR Spectra

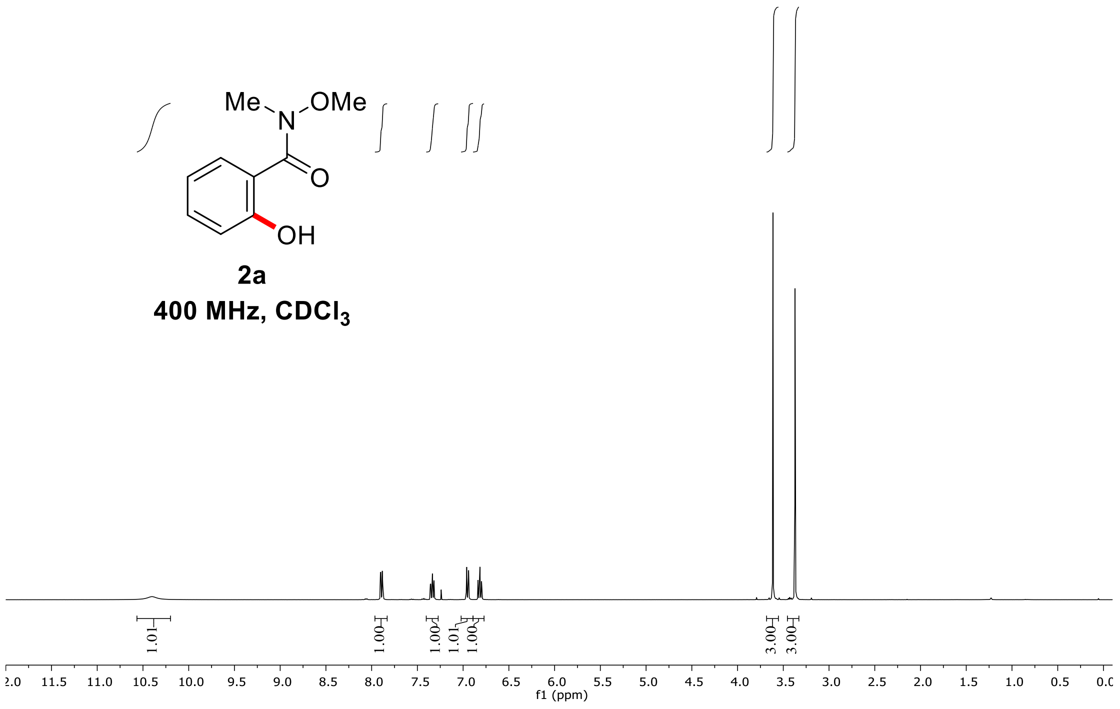
Me N OMe
O
OH
2a
400 MHz, $CDCl_3$
1.01
1.00
1.00
1.01
1.00
3.00
3.00
f1 (ppm)

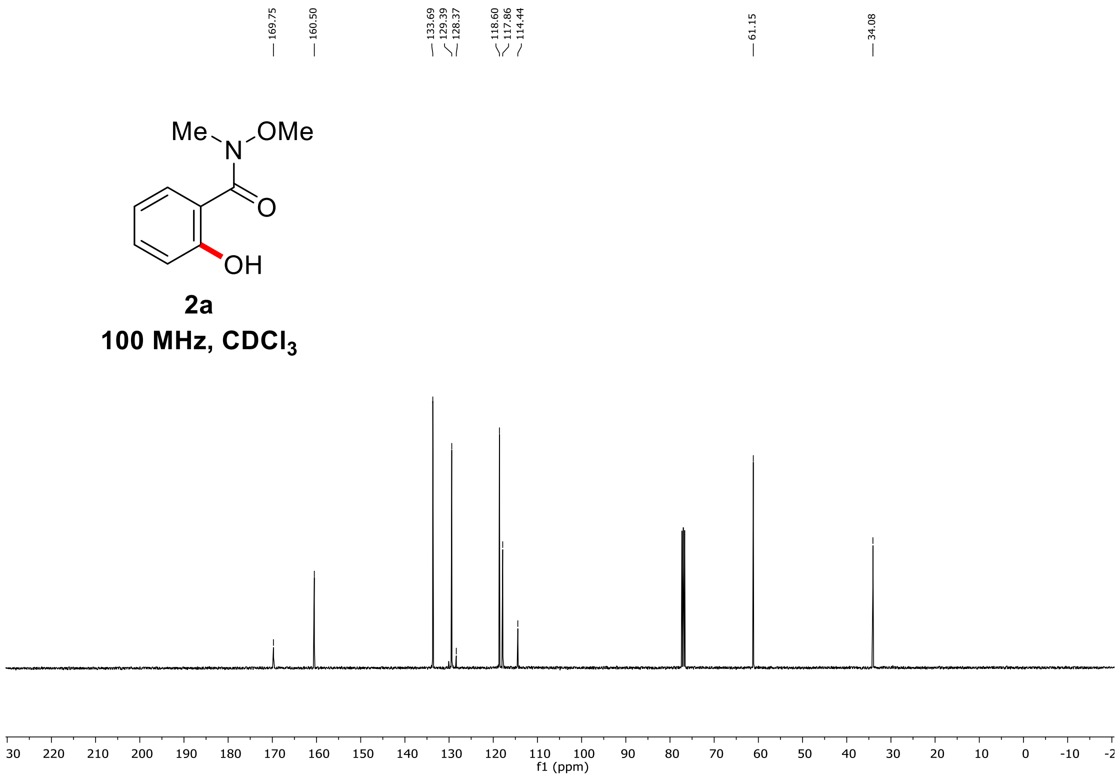
169.75
160.50
133.69
129.39
128.37
118.60
117.86
114.44
61.15
34.08
Me N OMe
O
OH
2a
100 MHz, $CDCl_3$
f1 (ppm)

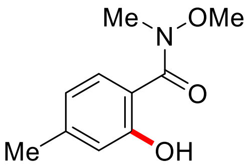

2b

400 MHz, $CDCl_3$

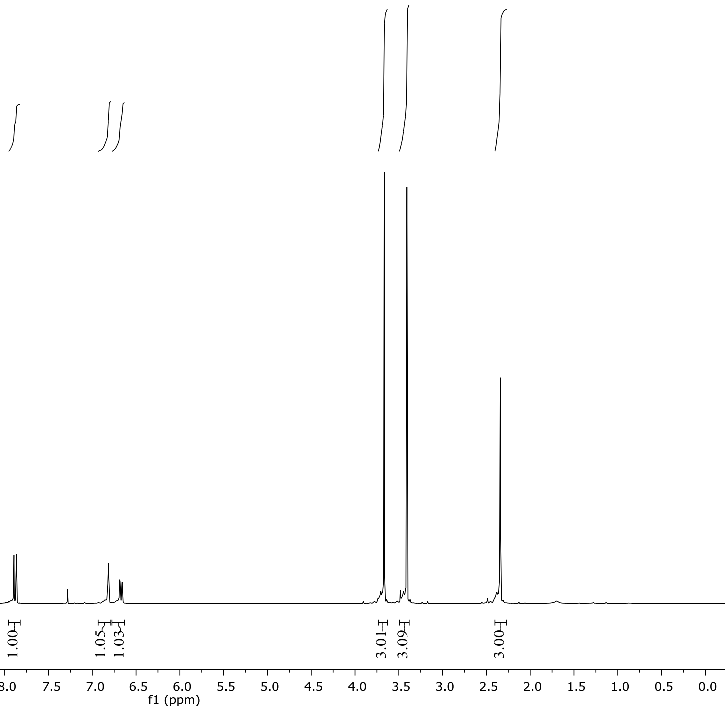

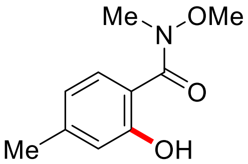

2b

100 MHz, $CDCl_3$

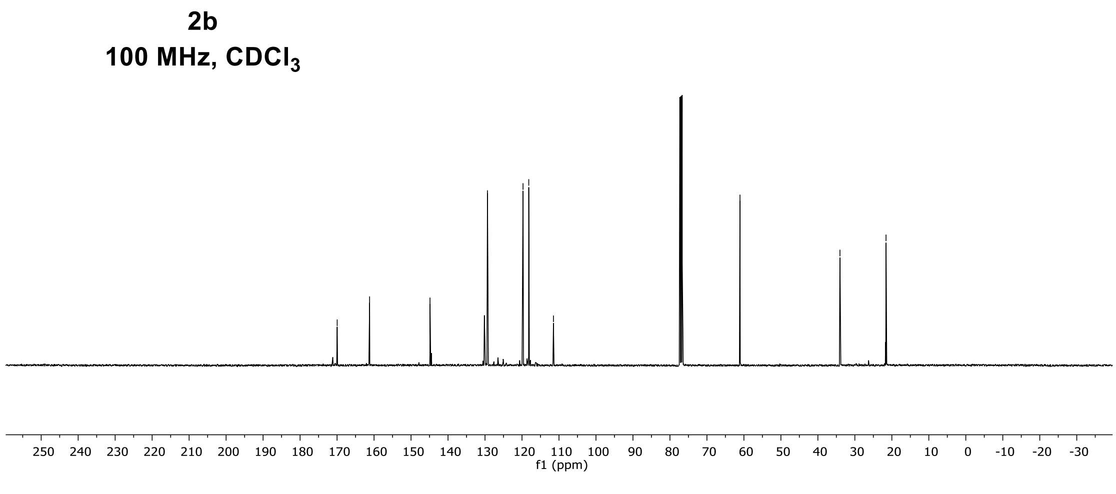

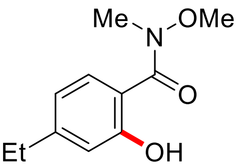

2c
400 MHz, $CDCl_3$

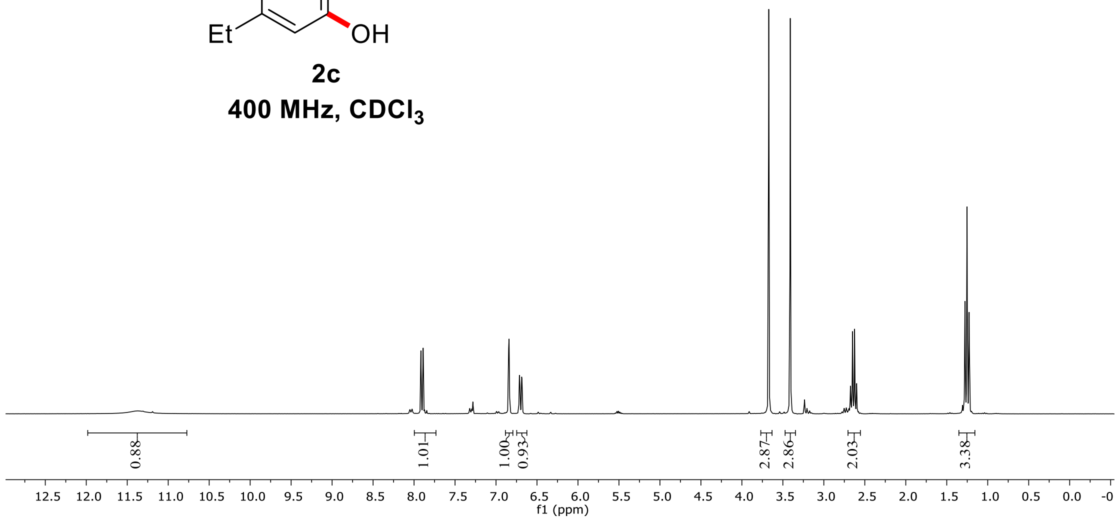

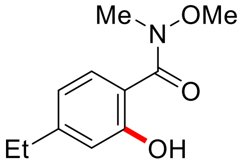

2c
100 MHz, $CDCl_3$

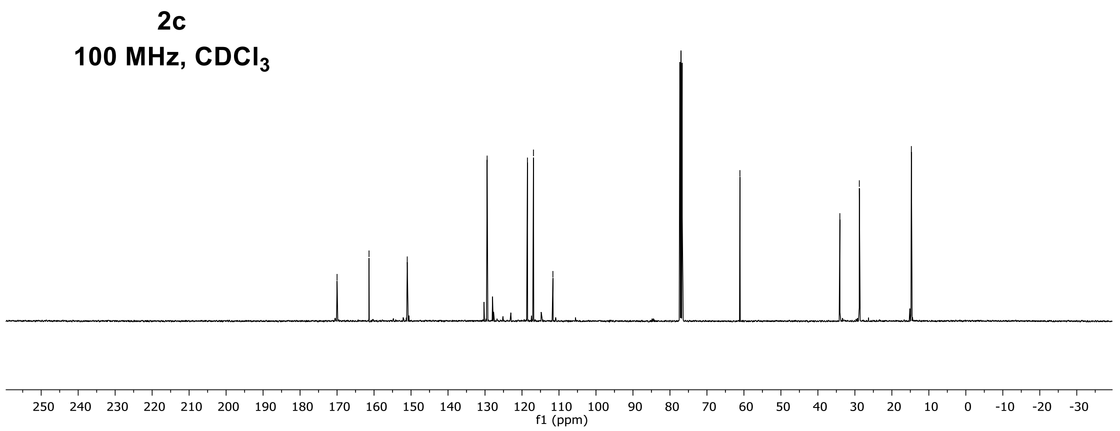

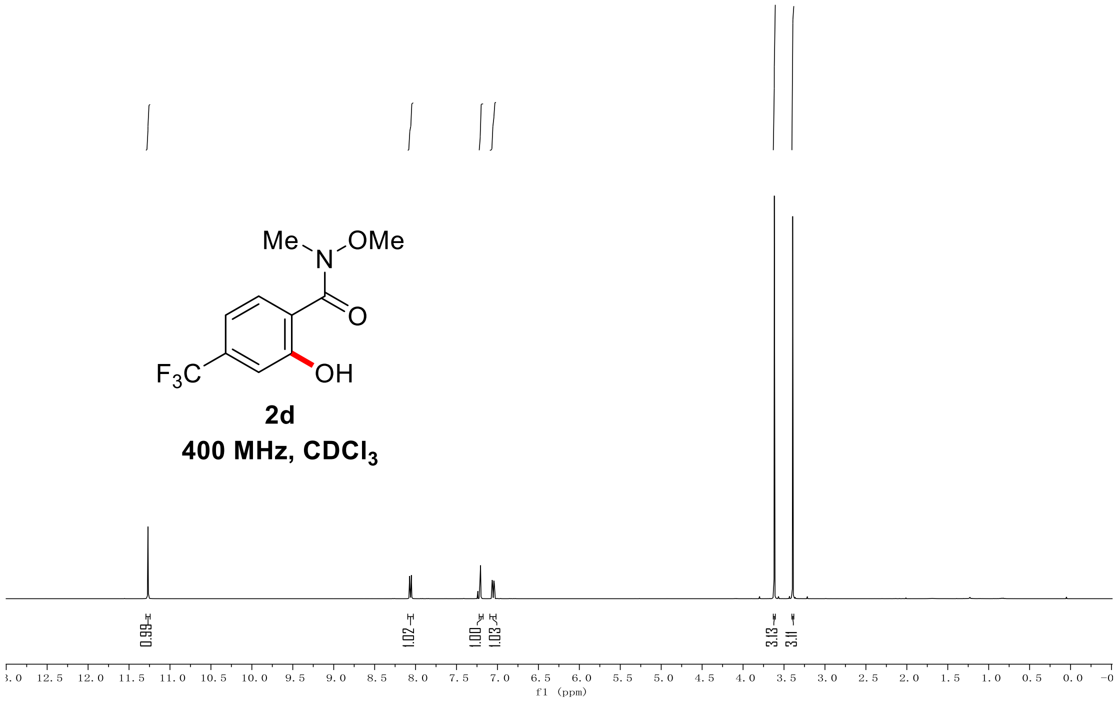

Me
N
OMe
O
F3C
OH
2d
400 MHz, CDCl3
0.99
1.02
1.00
1.03
3.13
3.11
f1 (ppm)

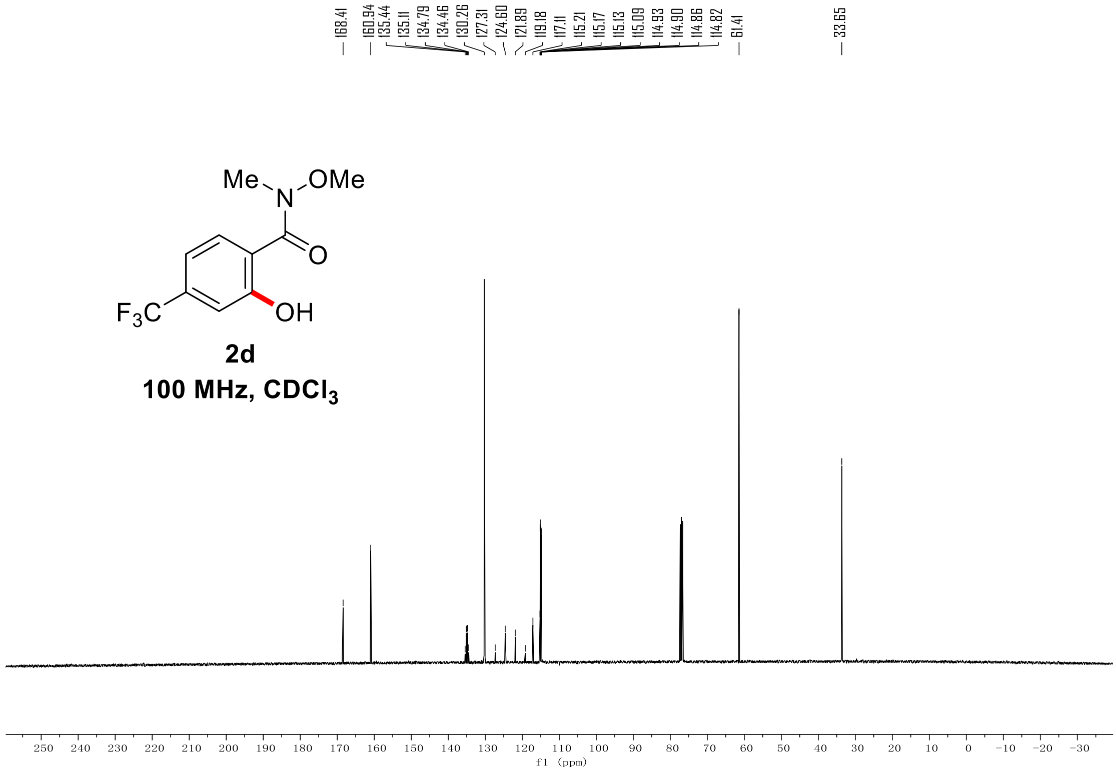

168.41
160.94
135.44
135.11
134.79
134.46
130.26
127.31
124.60
121.89
119.18
117.11
115.21
115.17
115.13
115.09
114.93
114.90
114.86
114.82
61.41
33.65
Me
N
OMe
O
F3C
OH
2d
100 MHz, CDCl3
f1 (ppm)

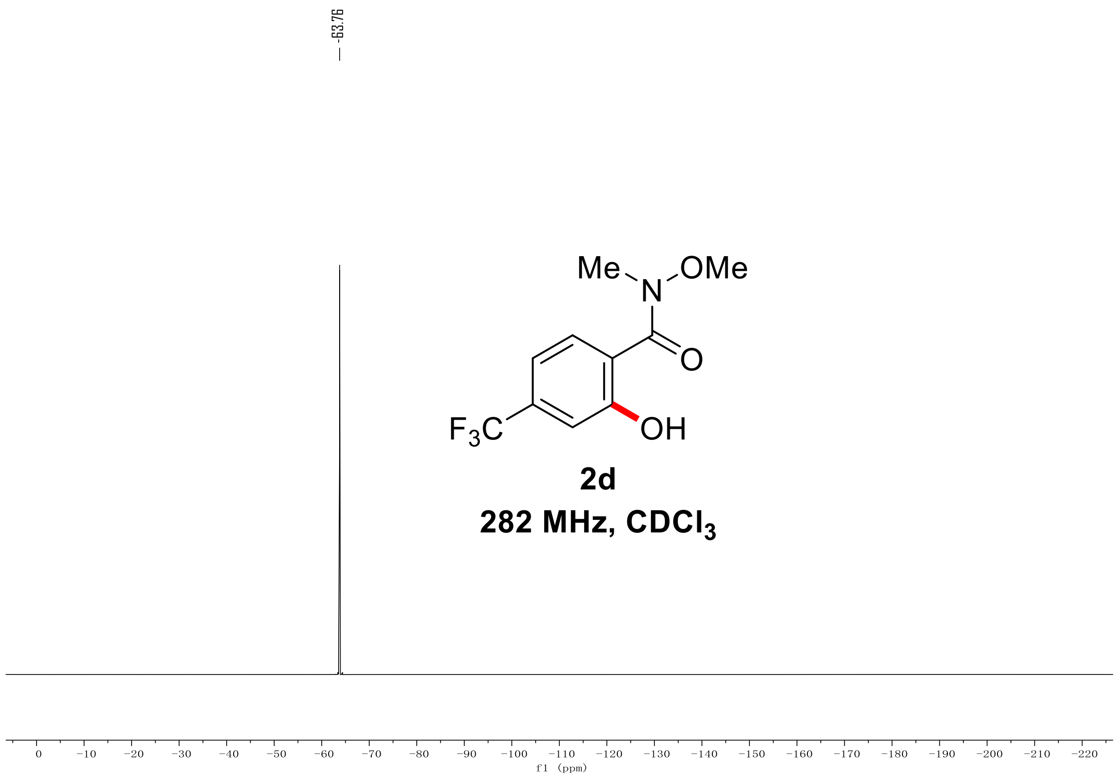

-63.76
Me
N
OMe
O
F3C
OH
2d
282 MHz, CDCl3
0
−10
−20
−30
−40
−50
−60
−70
−80
−90
−100
−110
−120
−130
−140
−150
−160
−170
−180
−190
−200
−210
−220
f1 (ppm)

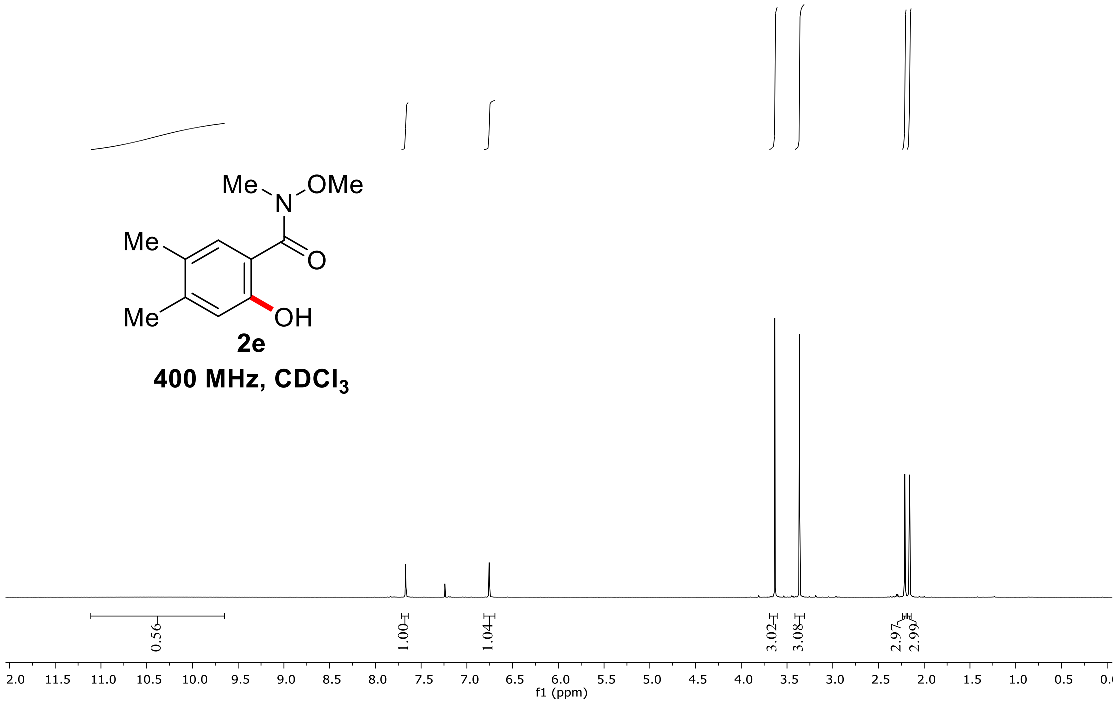

Me
N
OMe
Me
O
Me
OH
2e
400 MHz, CDCl3
0.56
1.00
1.04
3.02
3.08
2.97
2.99
f1 (ppm)

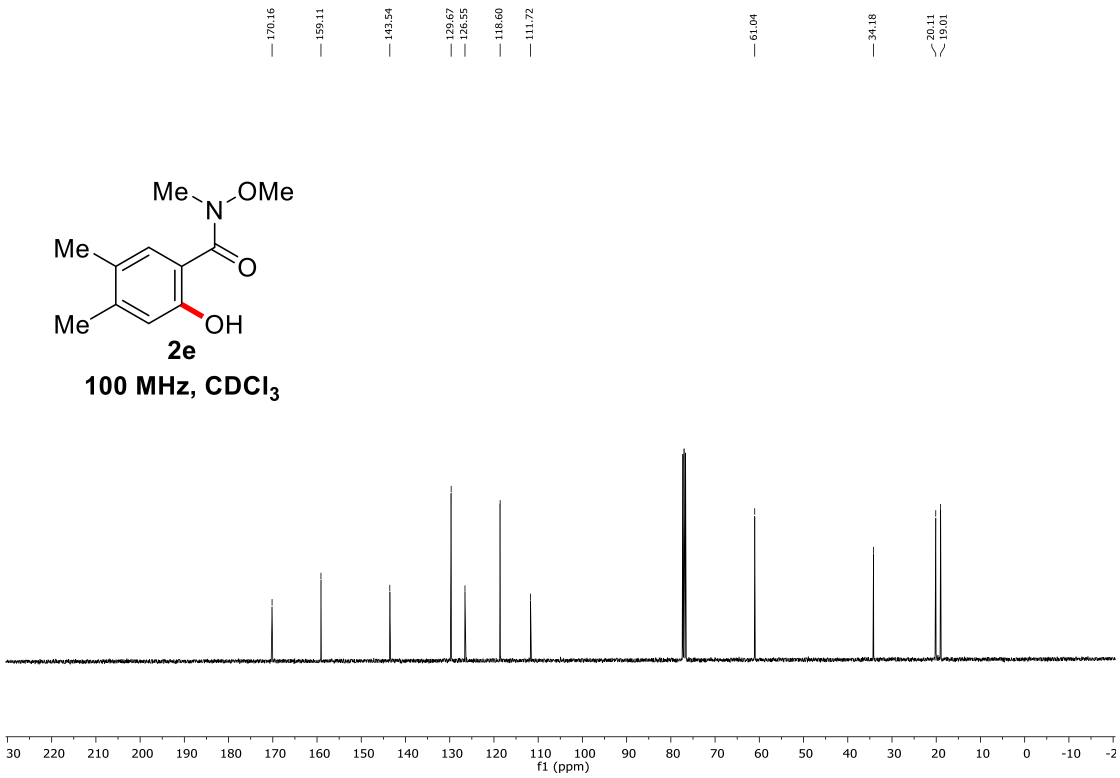

170.16
159.11
143.54
129.67
126.55
118.60
111.72
61.04
34.18
20.11
19.01
Me
N
OMe
Me
O
Me
OH
2e
100 MHz, CDCl3
f1 (ppm)

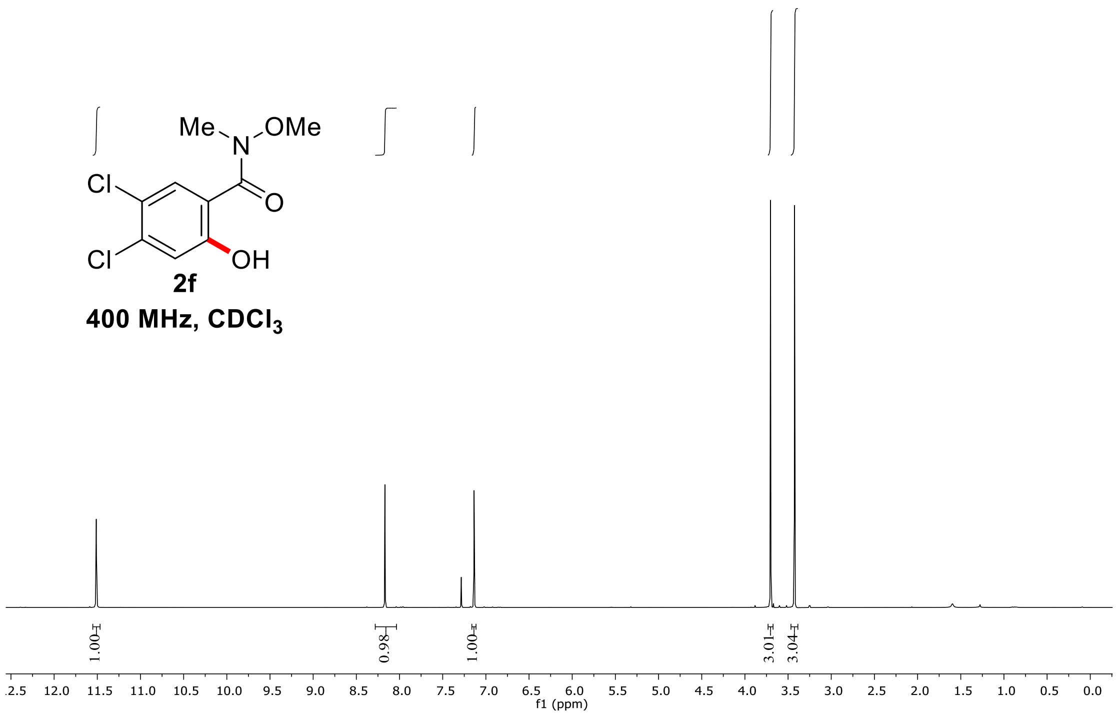
Me
N
OMe
Cl
O
Cl
OH
2f
400 MHz, CDCl3
1.00
0.98
1.00
3.01
3.04
f1 (ppm)

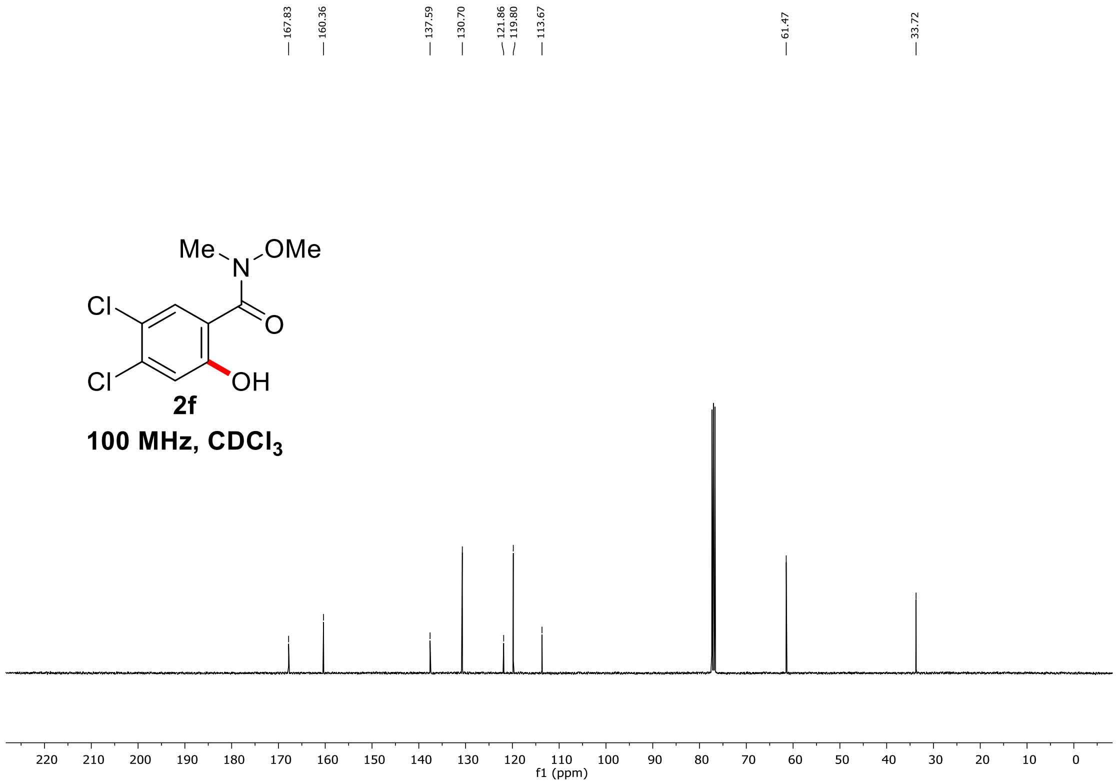
167.83
160.36
137.59
130.70
121.86
119.80
113.67
61.47
33.72
Me
N
OMe
Cl
O
Cl
OH
2f
100 MHz, CDCl3
f1 (ppm)

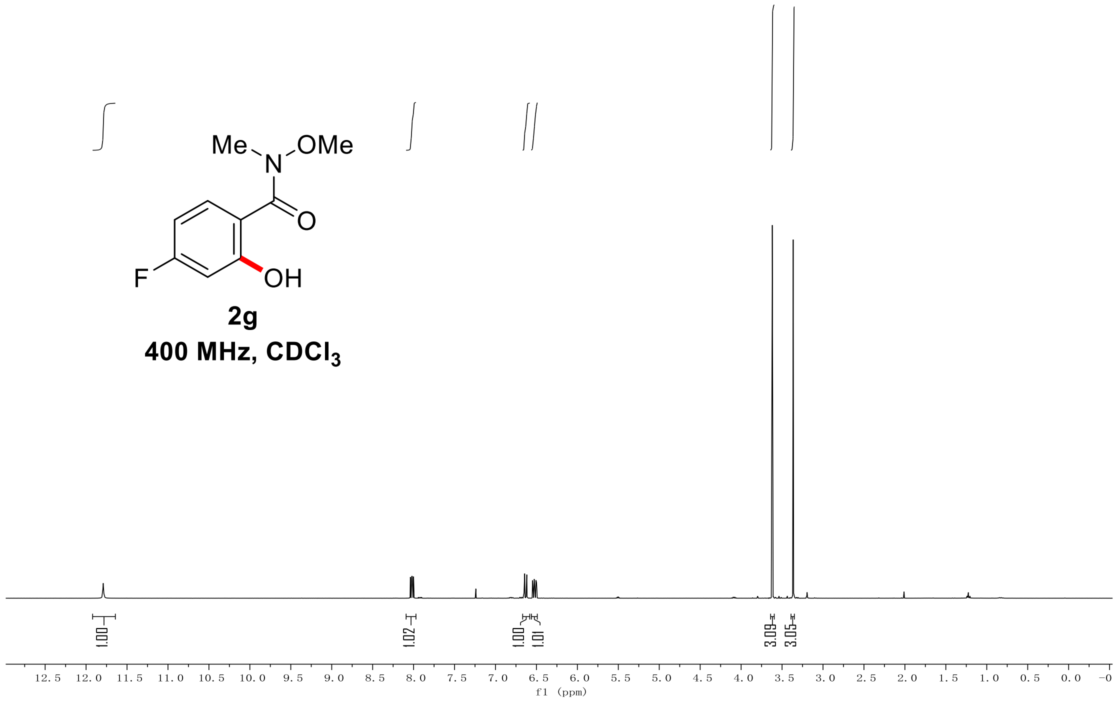

Me
N
OMe
O
F
OH
2g
400 MHz, CDCl3
1.00
1.02
1.00
1.01
3.09
3.05
12.5 12.0 11.5 11.0 10.5 10.0 9.5 9.0 8.5 8.0 7.5 7.0 6.5 6.0 5.5 5.0 4.5 4.0 3.5 3.0 2.5 2.0 1.5 1.0 0.5 0.0
f1 (ppm)

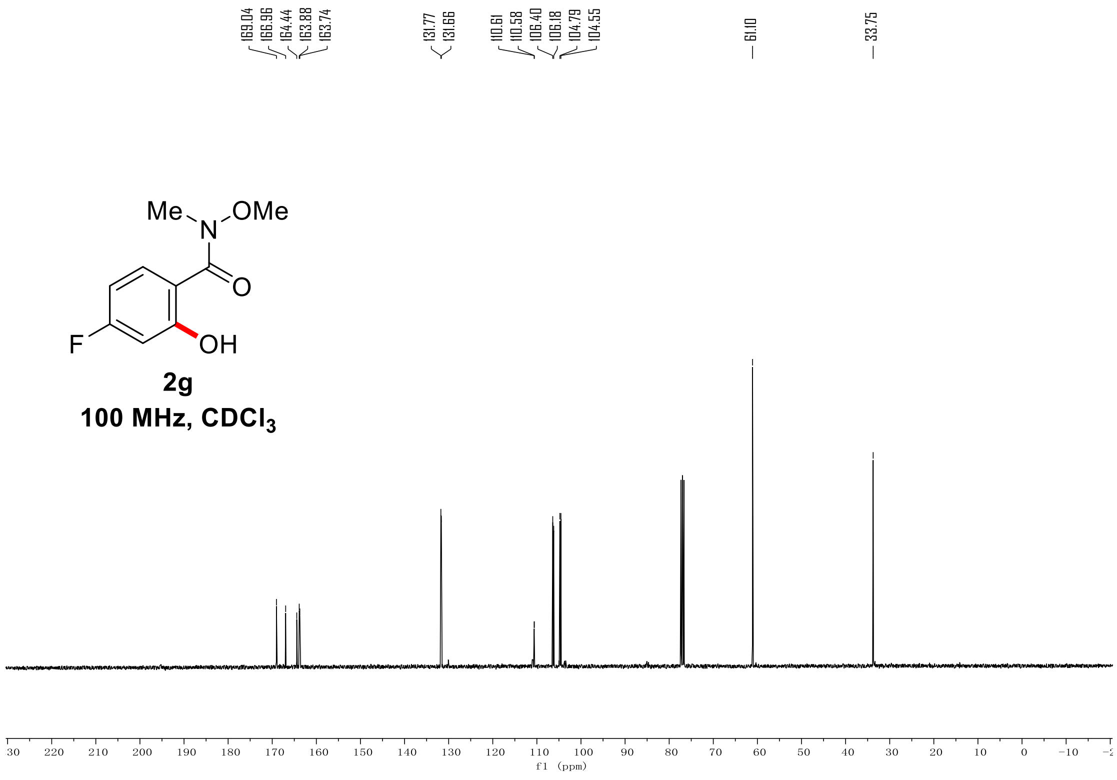

169.04
166.96
164.44
163.88
163.74
131.77
131.66
110.61
110.58
106.40
106.18
104.79
104.55
61.10
33.75
Me
N
OMe
O
F
OH
2g
100 MHz, CDCl3
220 210 200 190 180 170 160 150 140 130 120 110 100 90 80 70 60 50 40 30 20 10 0 −10
f1 (ppm)

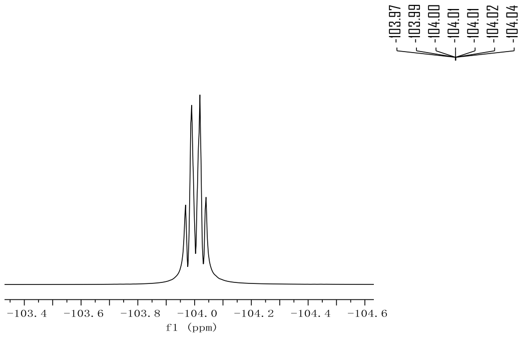

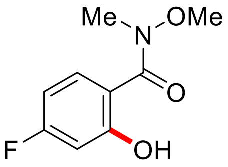

2g
376 MHz, $CDCl_3$

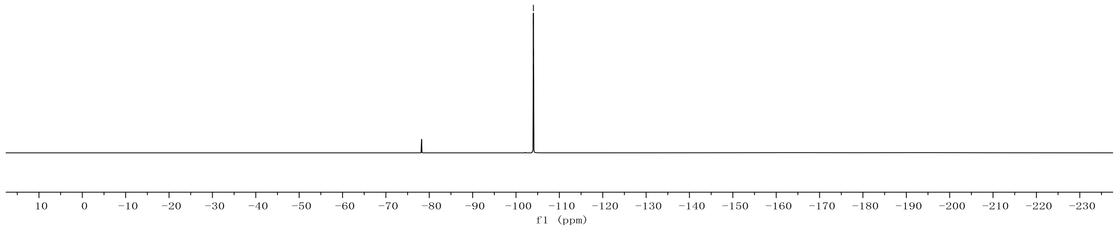

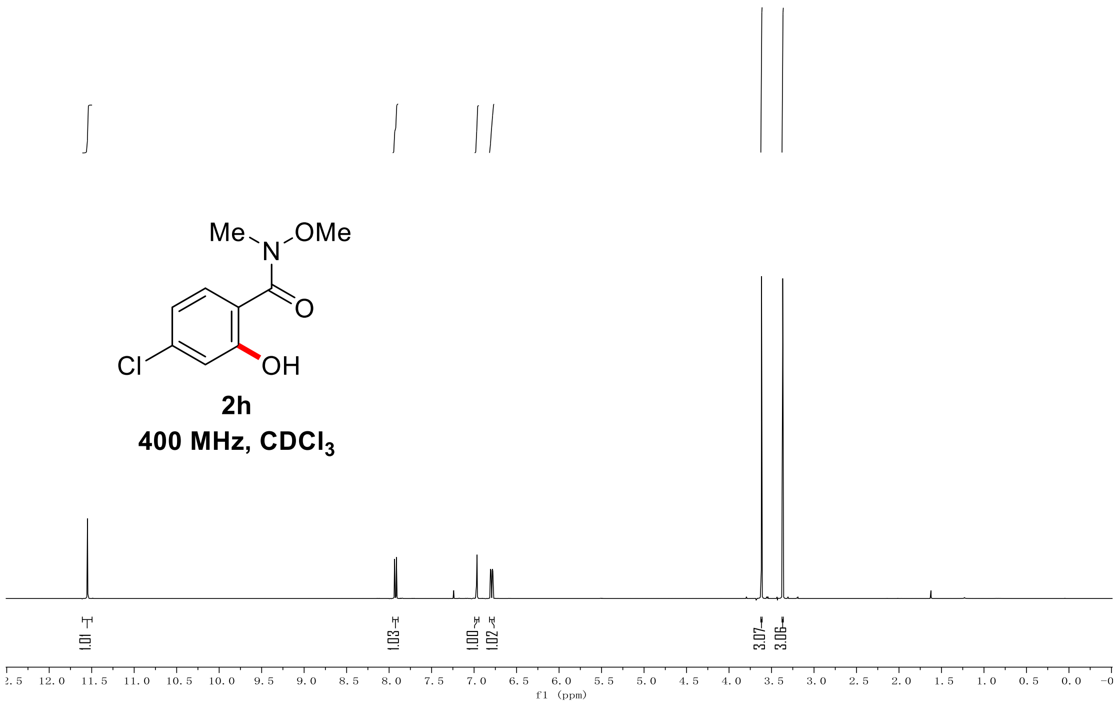

Me
N
OMe
O
Cl
OH
2h
400 MHz, CDCl3
1.01
1.03
1.00
1.02
3.07
3.06
f1 (ppm)

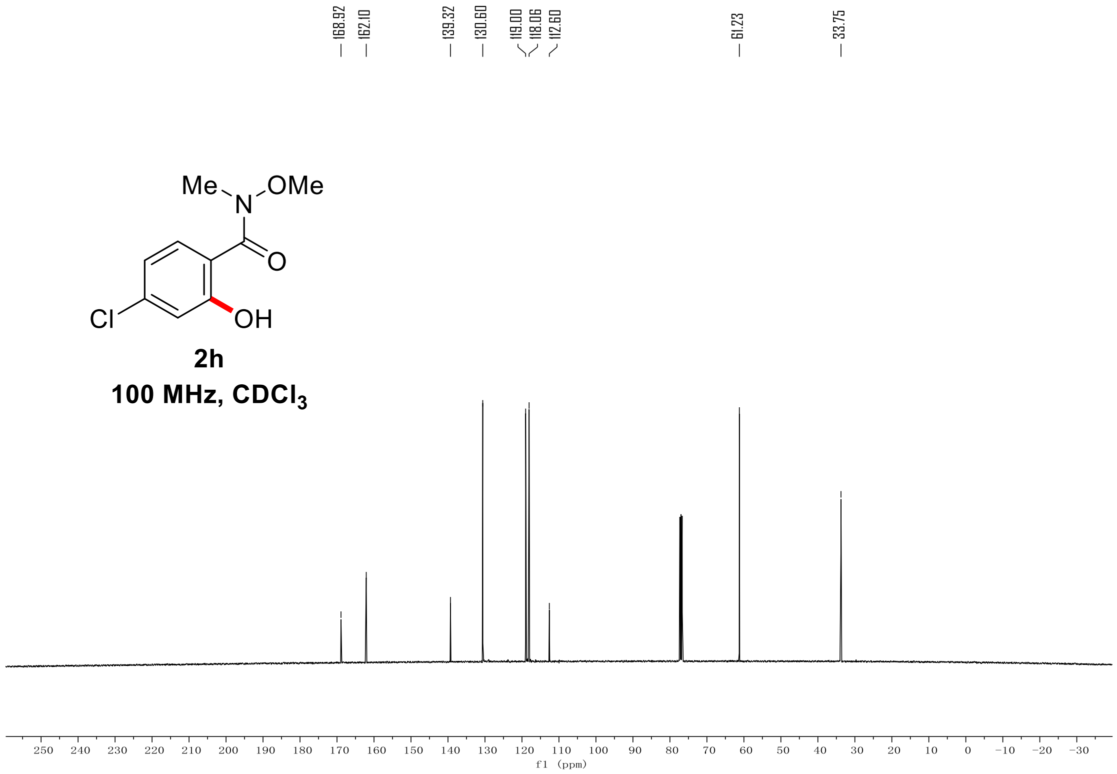

168.92
162.10
139.32
130.60
119.00
118.06
112.60
61.23
33.75
Me
N
OMe
O
Cl
OH
2h
100 MHz, CDCl3
f1 (ppm)

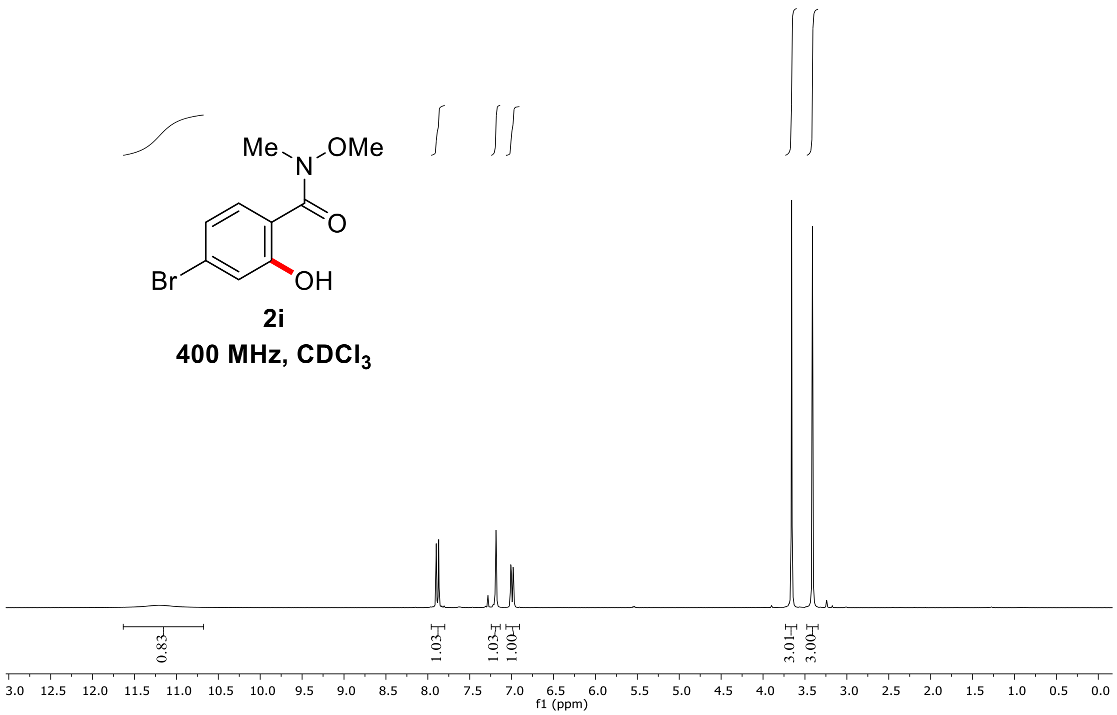

Me N OMe
O
Br OH
2i
400 MHz, CDCl3
0.83
1.03
1.03
1.00
3.01
3.00
f1 (ppm)

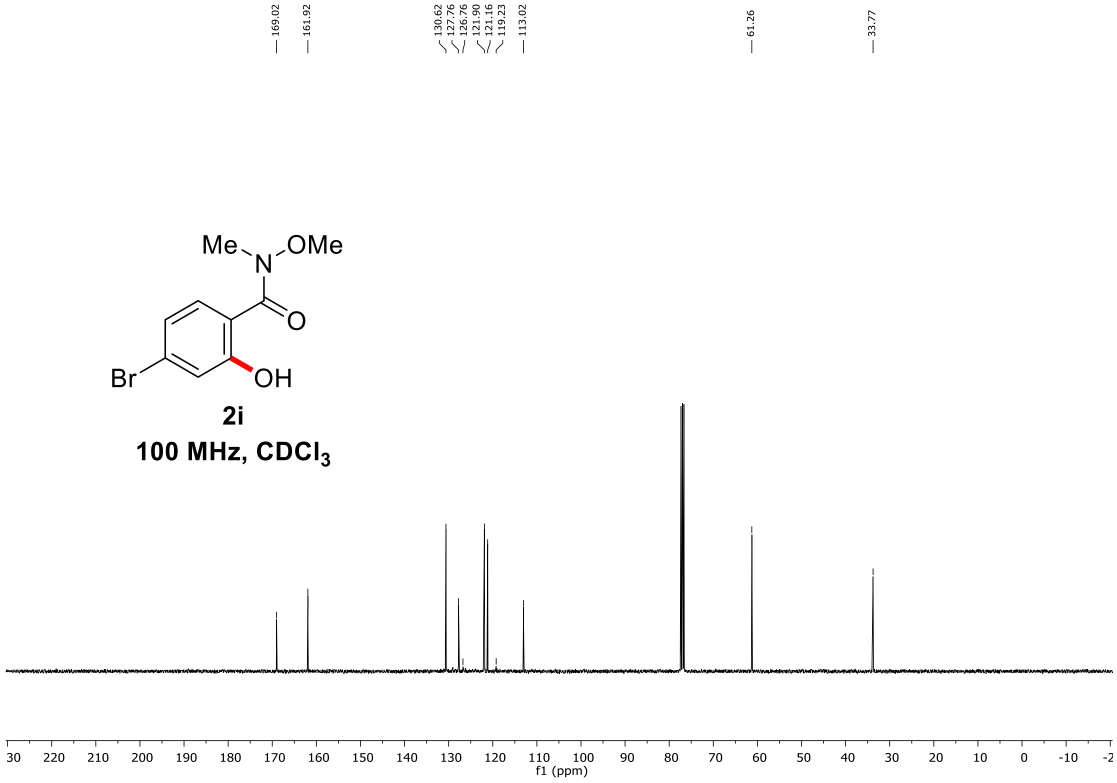

169.02
161.92
130.62
127.76
126.76
121.90
121.16
119.23
113.02
61.26
33.77
Me N OMe
O
Br OH
2i
100 MHz, CDCl3
f1 (ppm)

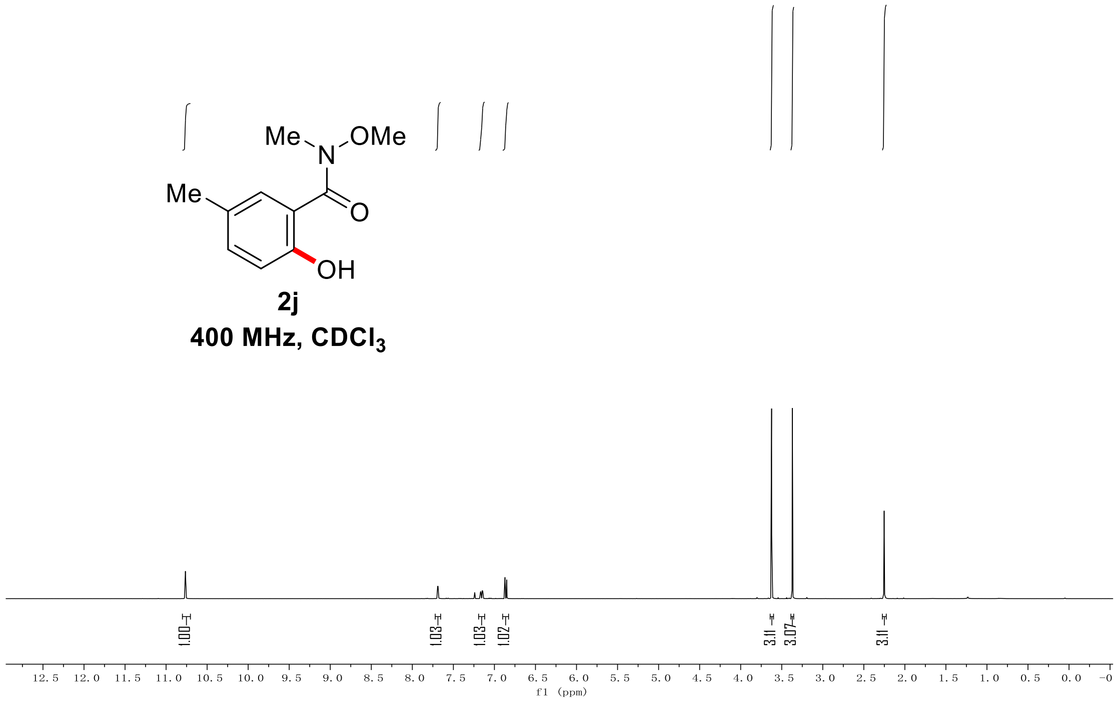
Me
N
OMe
Me
O
OH
2j
400 MHz, $CDCl_3$
1.00
1.03
1.03
1.02
3.11
3.07
3.11
12.5 12.0 11.5 11.0 10.5 10.0 9.5 9.0 8.5 8.0 7.5 7.0 6.5 6.0 5.5 5.0 4.5 4.0 3.5 3.0 2.5 2.0 1.5 1.0 0.5 0.0
f1 (ppm)

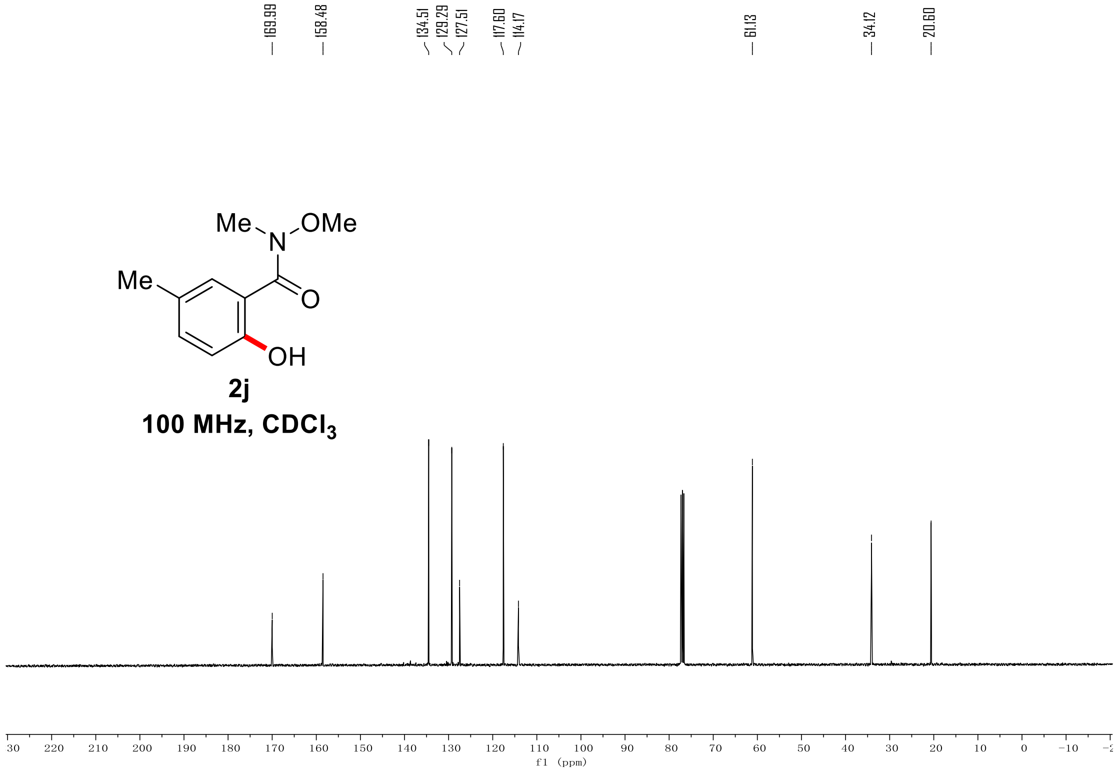
169.99
158.48
134.51
129.29
127.51
117.60
114.17
61.13
34.12
20.60
Me
N
OMe
Me
O
OH
2j
100 MHz, $CDCl_3$
220 210 200 190 180 170 160 150 140 130 120 110 100 90 80 70 60 50 40 30 20 10 0 -10
f1 (ppm)

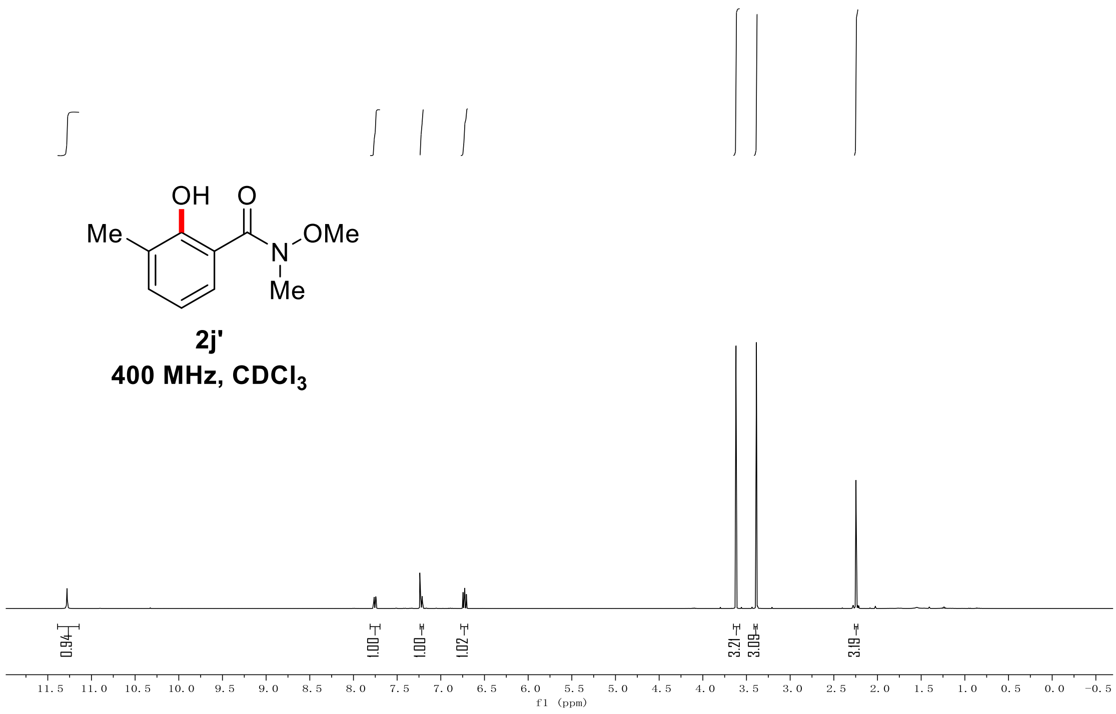

OH
O
Me
N
OMe
Me
2j'
400 MHz, CDCl3
0.94
1.00
1.00
1.02
3.21
3.09
3.19
11.5
11.0
10.5
10.0
9.5
9.0
8.5
8.0
7.5
7.0
6.5
6.0
5.5
5.0
4.5
4.0
3.5
3.0
2.5
2.0
1.5
1.0
0.5
0.0
-0.5
f1 (ppm)

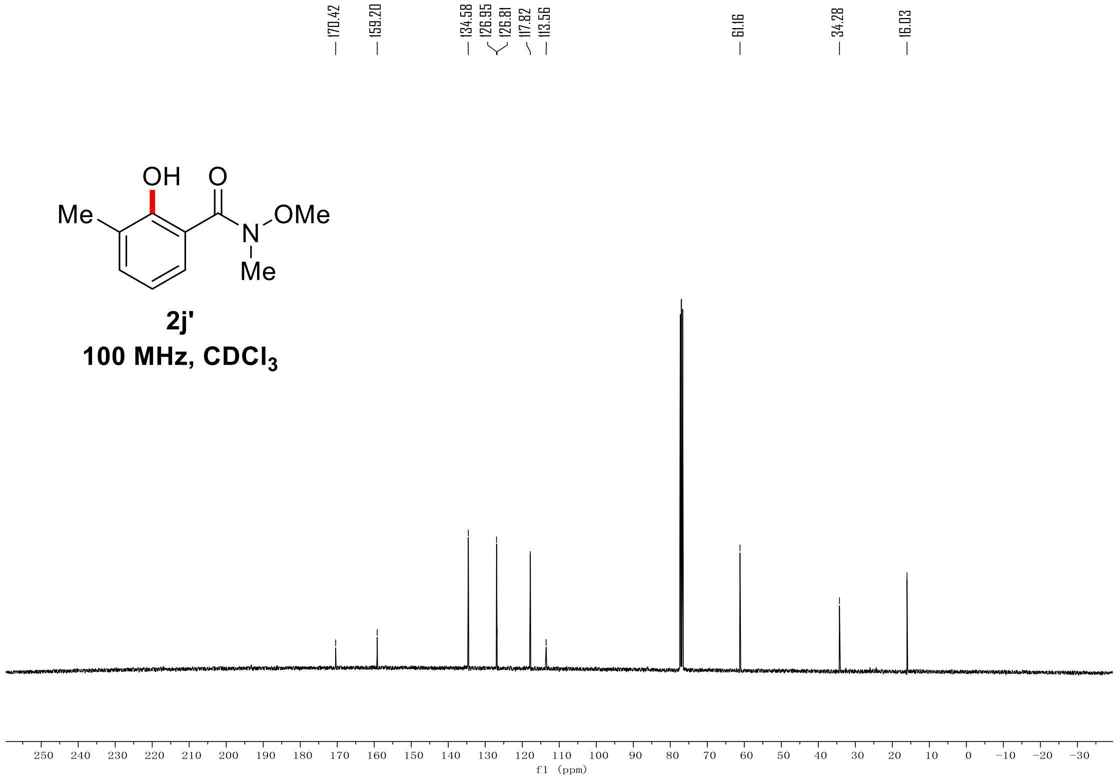

170.42
159.20
134.58
126.95
126.81
117.82
113.56
61.16
34.28
16.03
OH
O
Me
N
OMe
Me
2j'
100 MHz, CDCl3
250
240
230
220
210
200
190
180
170
160
150
140
130
120
110
100
90
80
70
60
50
40
30
20
10
0
-10
-20
-30
f1 (ppm)

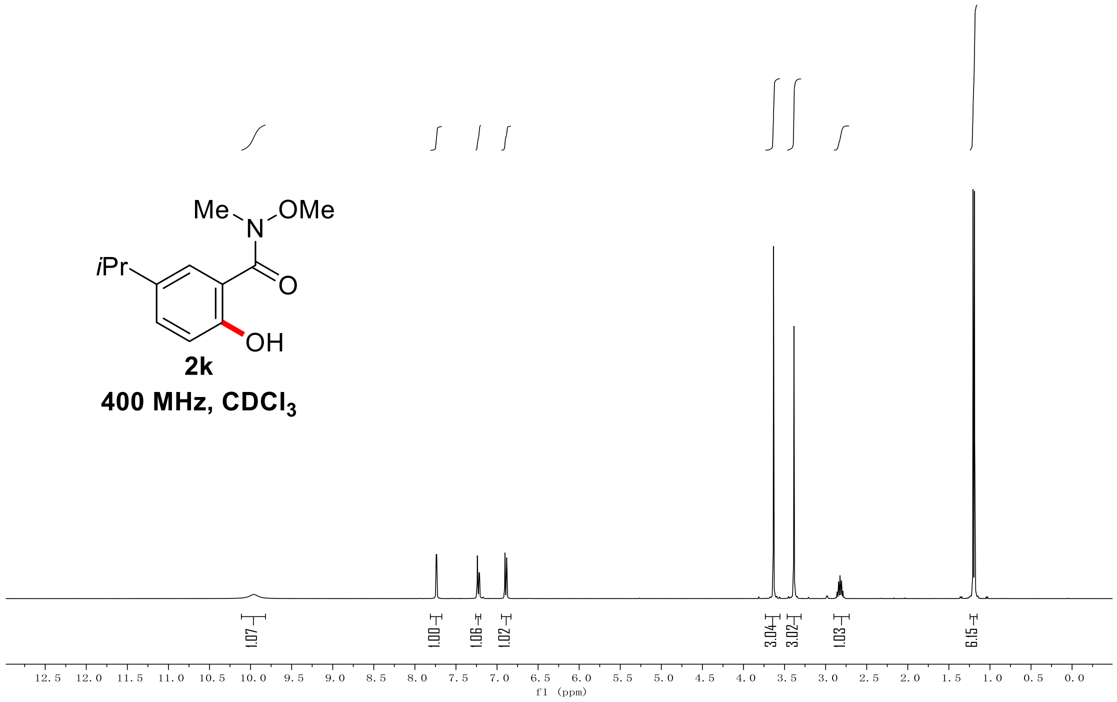
Me
N
OMe
iPr
O
OH
2k
400 MHz, CDCl3
1.07
1.00
1.06
1.02
3.04
3.02
1.03
6.15
12.5 12.0 11.5 11.0 10.5 10.0 9.5 9.0 8.5 8.0 7.5 7.0 6.5 6.0 5.5 5.0 4.5 4.0 3.5 3.0 2.5 2.0 1.5 1.0 0.5 0.0
f1 (ppm)

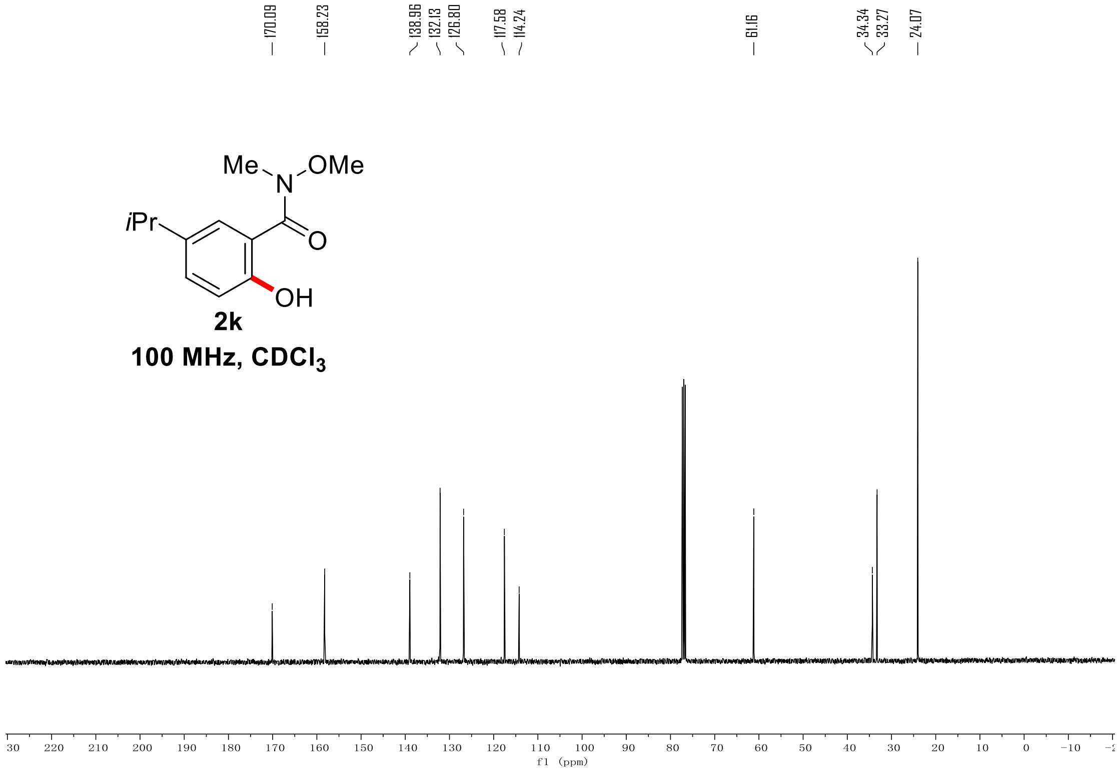
170.09
158.23
138.96
132.13
126.80
117.58
114.24
61.16
34.34
33.27
24.07
Me
N
OMe
iPr
O
OH
2k
100 MHz, CDCl3
220 210 200 190 180 170 160 150 140 130 120 110 100 90 80 70 60 50 40 30 20 10 0 -10
f1 (ppm)

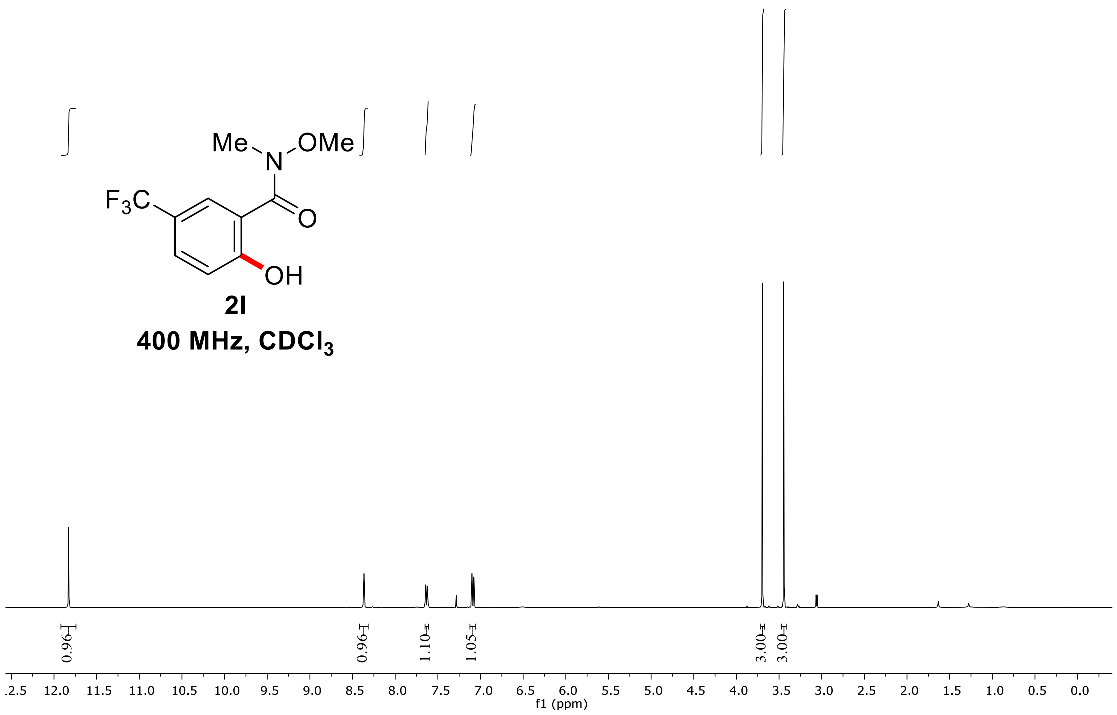
Me
N
OMe
F_3C
O
OH
2l
400 MHz, $CDCl_3$
0.96
0.96
1.10
1.05
3.00
3.00
f1 (ppm)

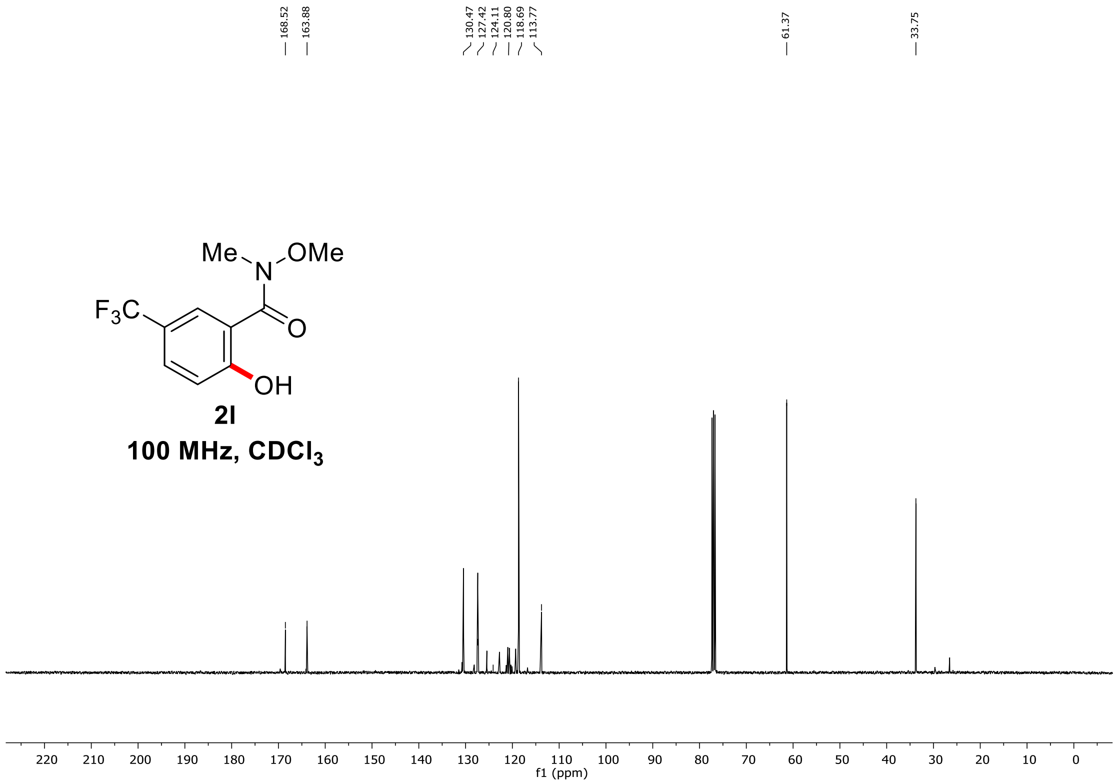
168.52
163.88
130.47
127.42
124.11
120.80
118.69
113.77
61.37
33.75
Me
N
OMe
F_3C
O
OH
2l
100 MHz, $CDCl_3$
f1 (ppm)

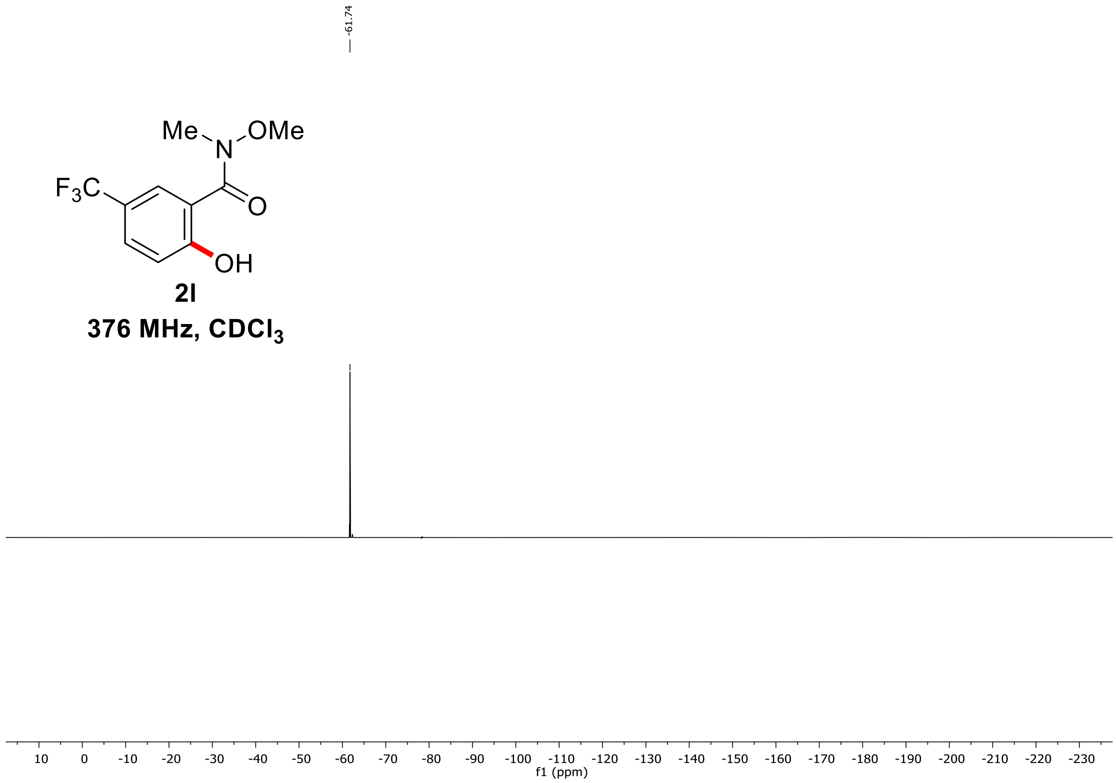
-61.74
Me
N
OMe
F_3C
O
OH
2l
376 MHz, $CDCl_3$
10 0 -10 -20 -30 -40 -50 -60 -70 -80 -90 -100 -110 -120 -130 -140 -150 -160 -170 -180 -190 -200 -210 -220 -230
f1 (ppm)

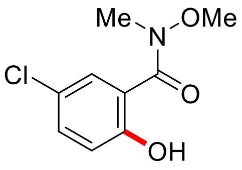

2m

400 MHz, $CDCl_3$

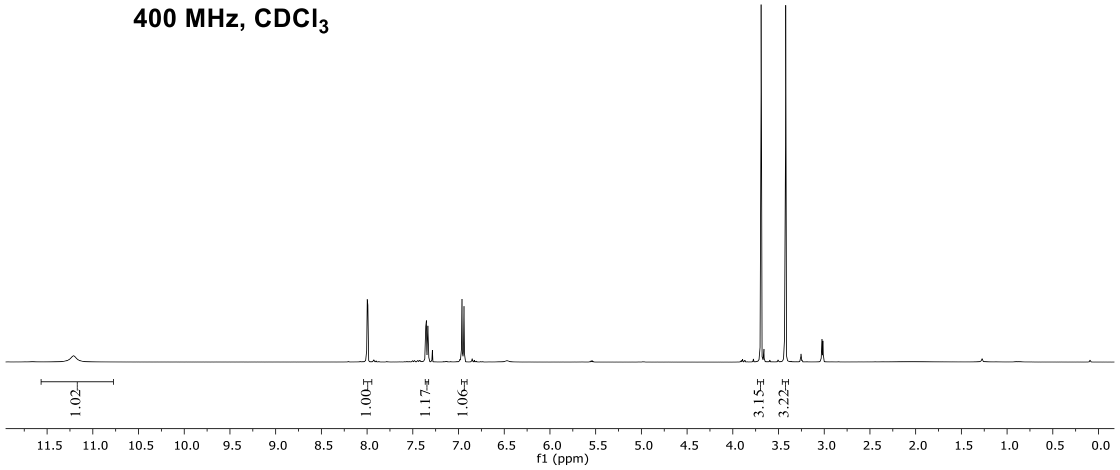

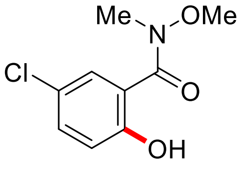

2m

100 MHz, $CDCl_3$

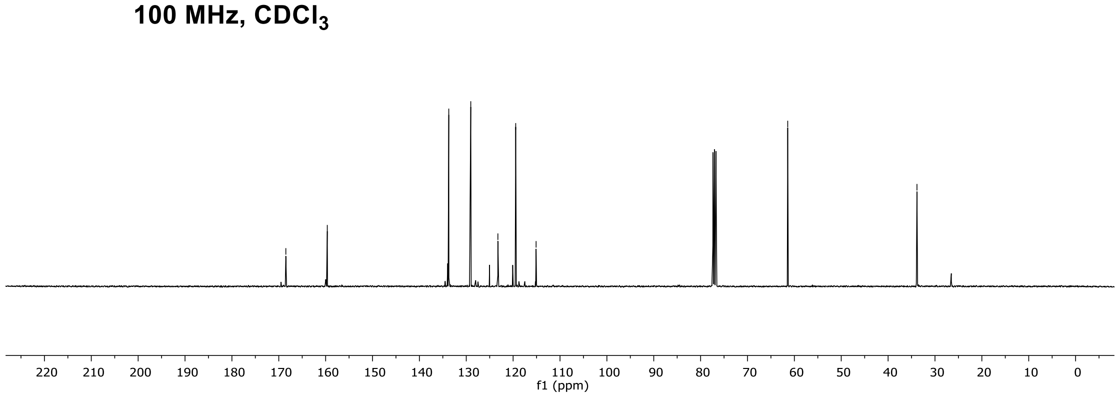

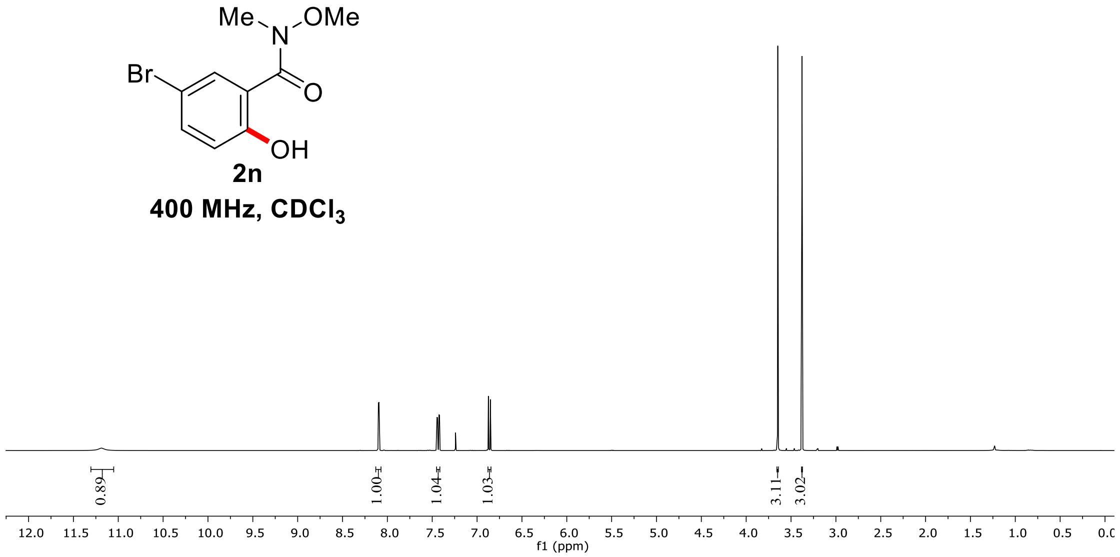
Me
N
OMe
Br
O
OH
2n
400 MHz, CDCl3
0.89
1.00
1.04
1.03
3.11
3.02
12.0 11.5 11.0 10.5 10.0 9.5 9.0 8.5 8.0 7.5 7.0 6.5 6.0 5.5 5.0 4.5 4.0 3.5 3.0 2.5 2.0 1.5 1.0 0.5 0.0
f1 (ppm)

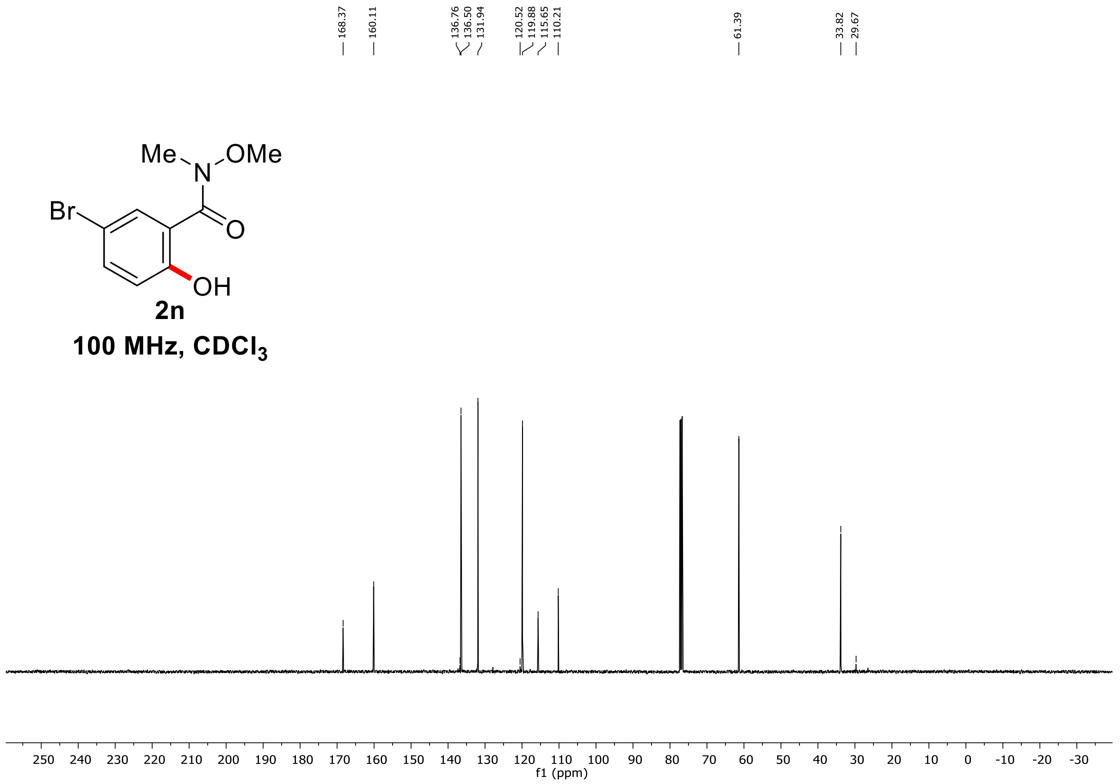
168.37
160.11
136.76
136.50
131.94
120.52
119.88
115.65
110.21
61.39
33.82
29.67
Me
N
OMe
Br
O
OH
2n
100 MHz, CDCl3
250 240 230 220 210 200 190 180 170 160 150 140 130 120 110 100 90 80 70 60 50 40 30 20 10 0 -10 -20 -30
f1 (ppm)

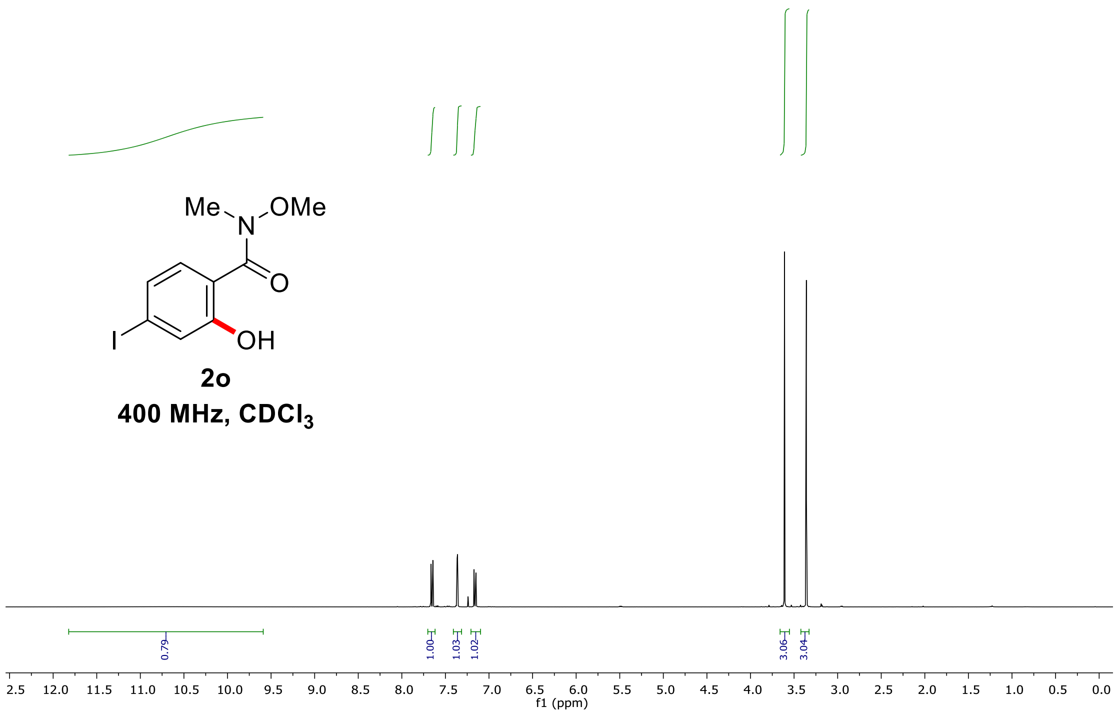
Me
N
OMe
O
OH
I
2o
400 MHz, CDCl3
0.79
1.00
1.03
1.02
3.06
3.04
f1 (ppm)

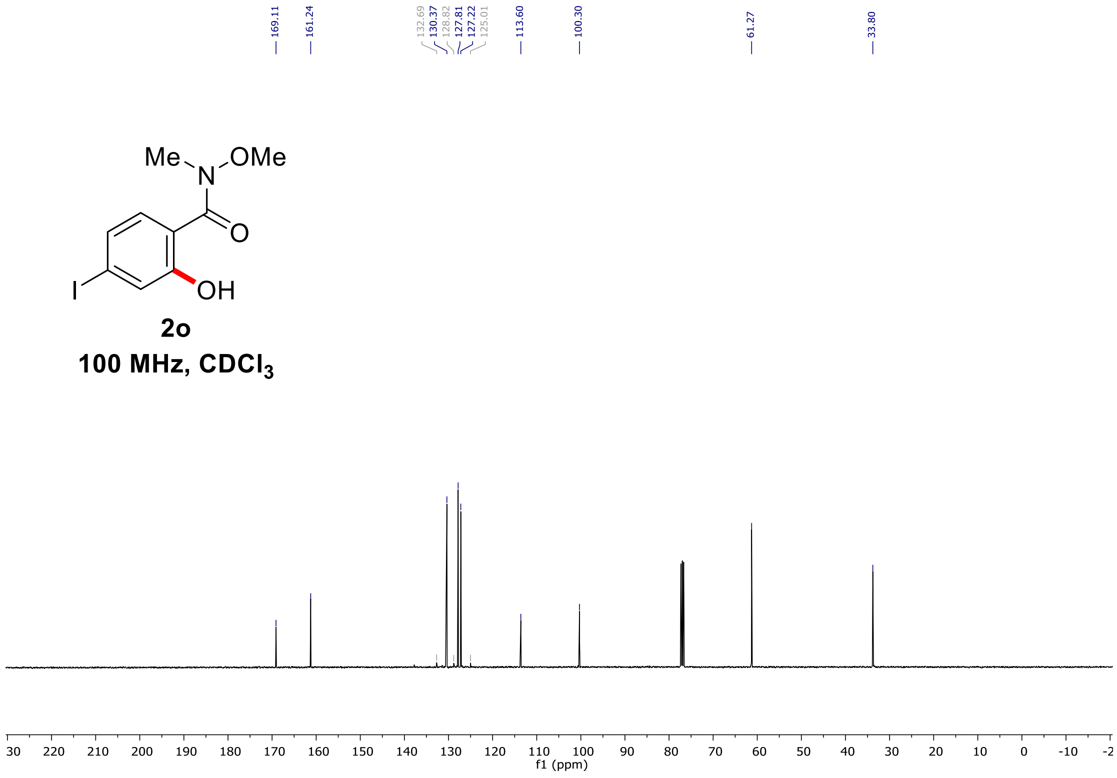
169.11
161.24
132.69
130.37
128.82
127.81
127.22
125.01
113.60
100.30
61.27
33.80
Me
N
OMe
O
OH
I
2o
100 MHz, CDCl3
f1 (ppm)

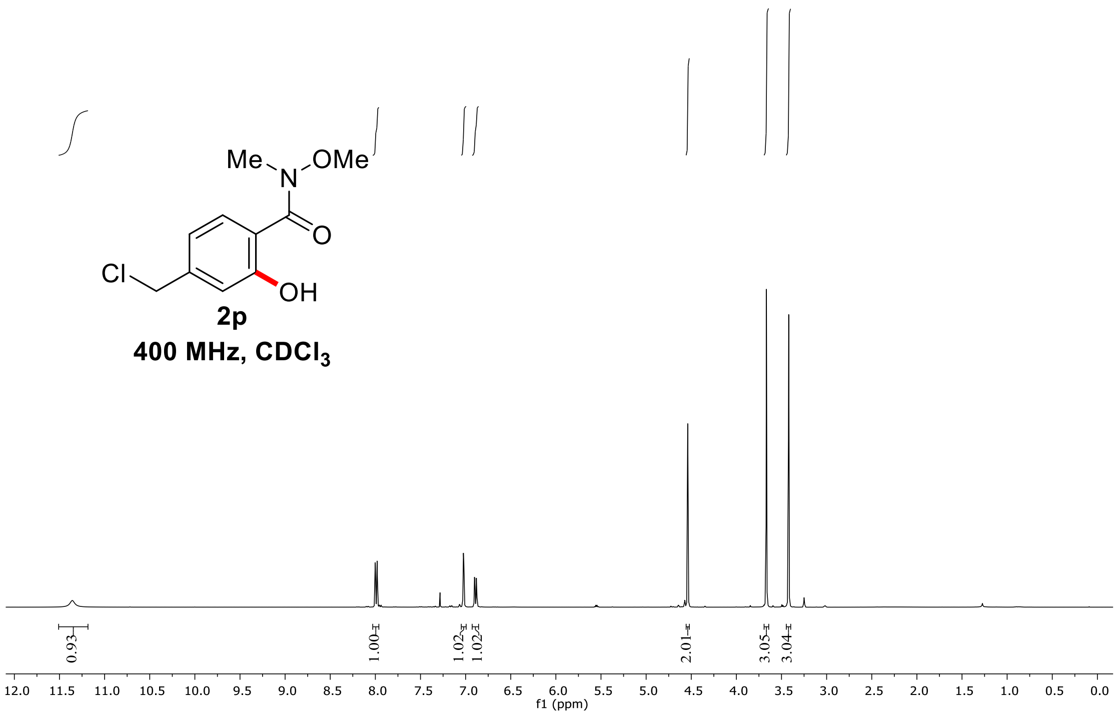

2p

400 MHz, CDCl$_3$

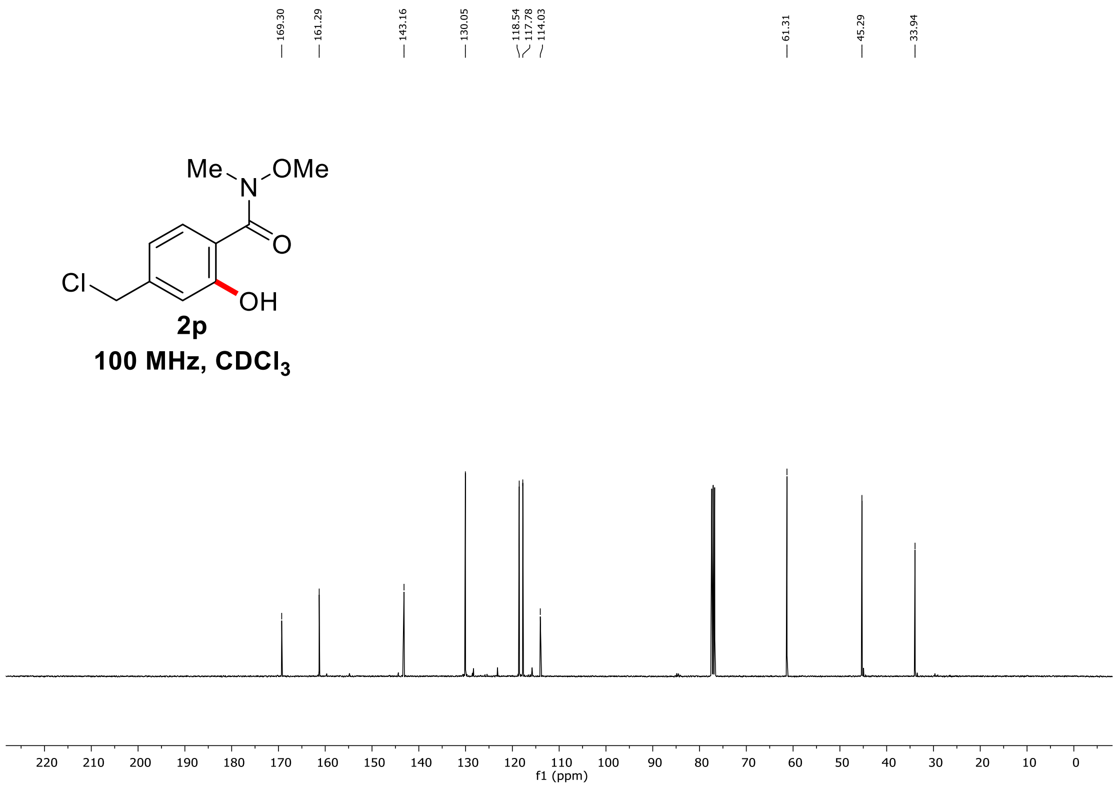

2p

100 MHz, CDCl$_3$

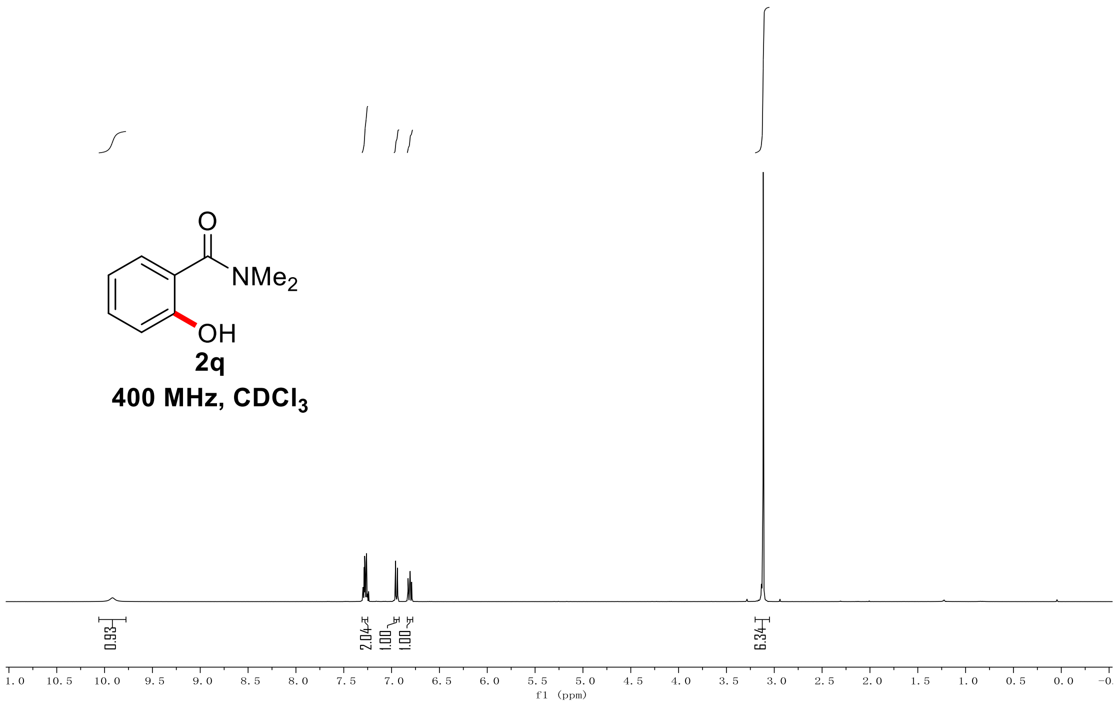
O
NMe2
OH
2q
400 MHz, CDCl3
0.93
2.04
1.00
1.00
6.34
f1 (ppm)

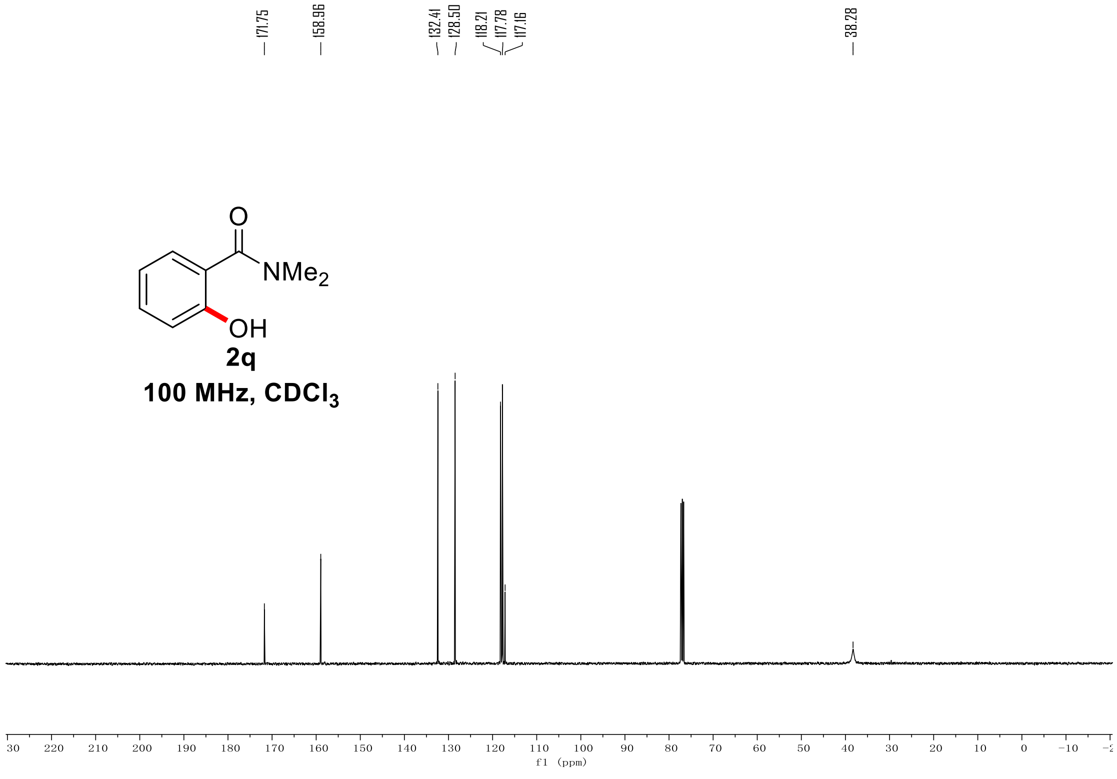
171.75
158.96
132.41
128.50
118.21
117.78
117.16
38.28
O
NMe2
OH
2q
100 MHz, CDCl3
f1 (ppm)

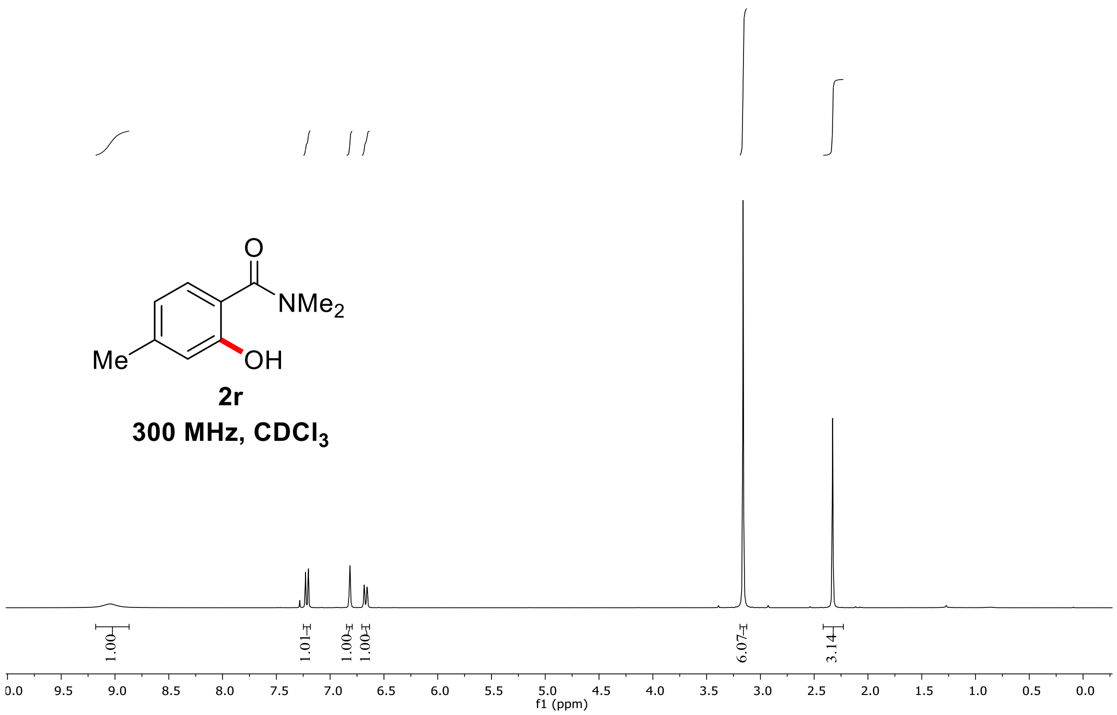
O
NMe2
Me
OH
2r
300 MHz, CDCl3
1.00
1.01
1.00
1.00
6.07
3.14
f1 (ppm)

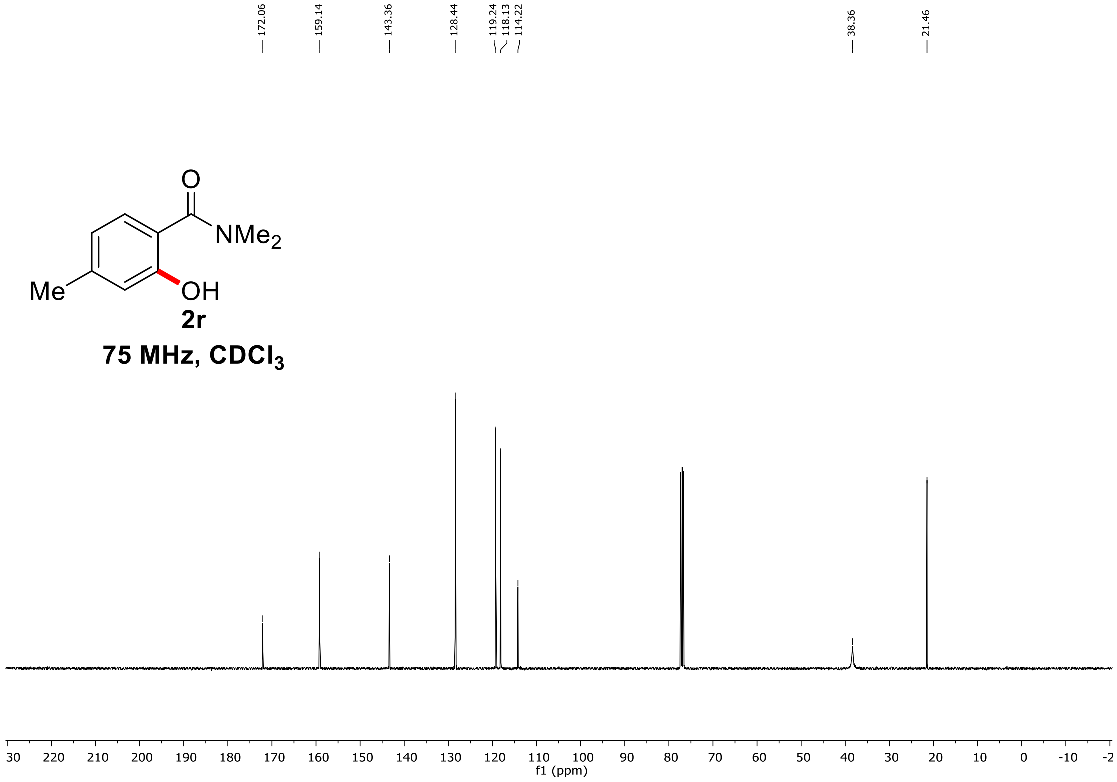
172.06
159.14
143.36
128.44
119.24
118.13
114.22
38.36
21.46
O
NMe2
Me
OH
2r
75 MHz, CDCl3
f1 (ppm)

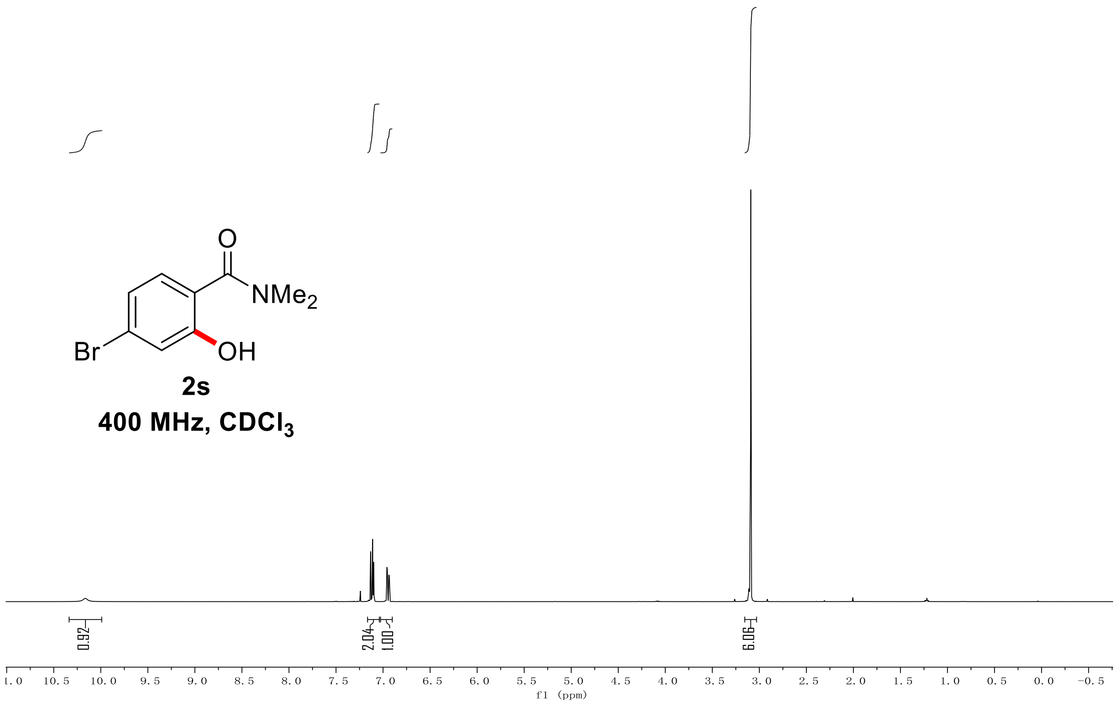
O
NMe2
Br
OH
2s
400 MHz, CDCl3
0.92
2.04
1.00
6.06
f1 (ppm)

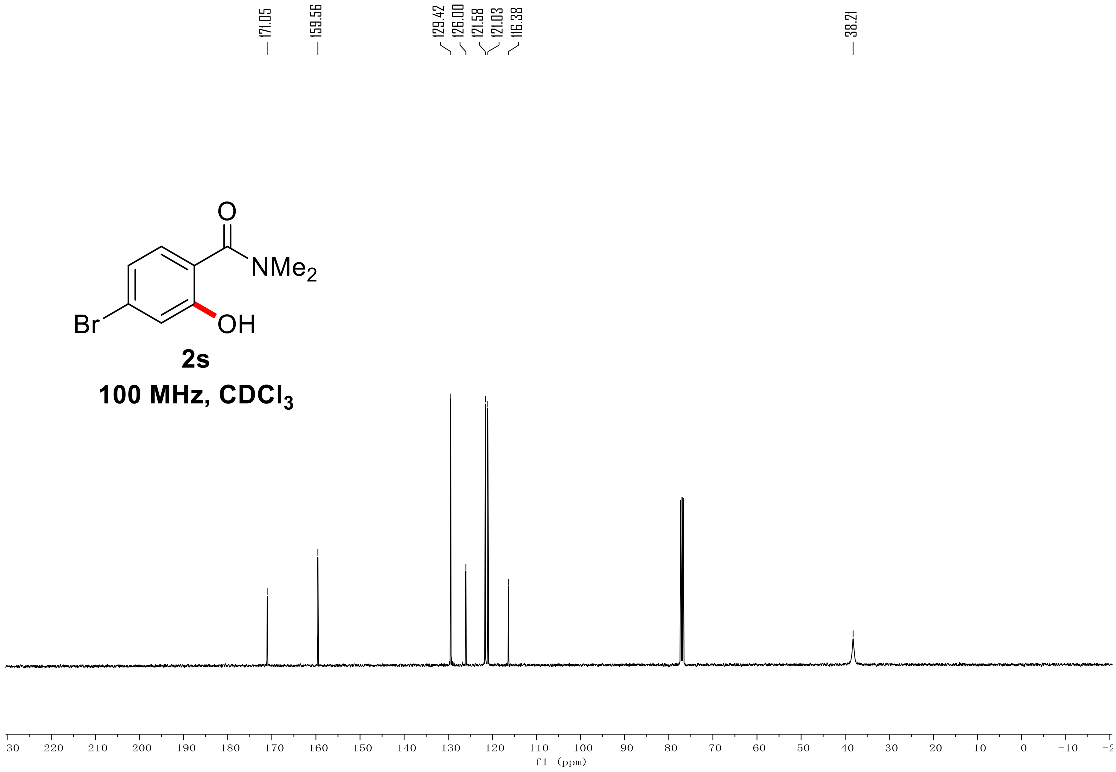
171.05
159.56
129.42
126.00
121.58
121.03
116.38
38.21
O
NMe2
Br
OH
2s
100 MHz, CDCl3
f1 (ppm)

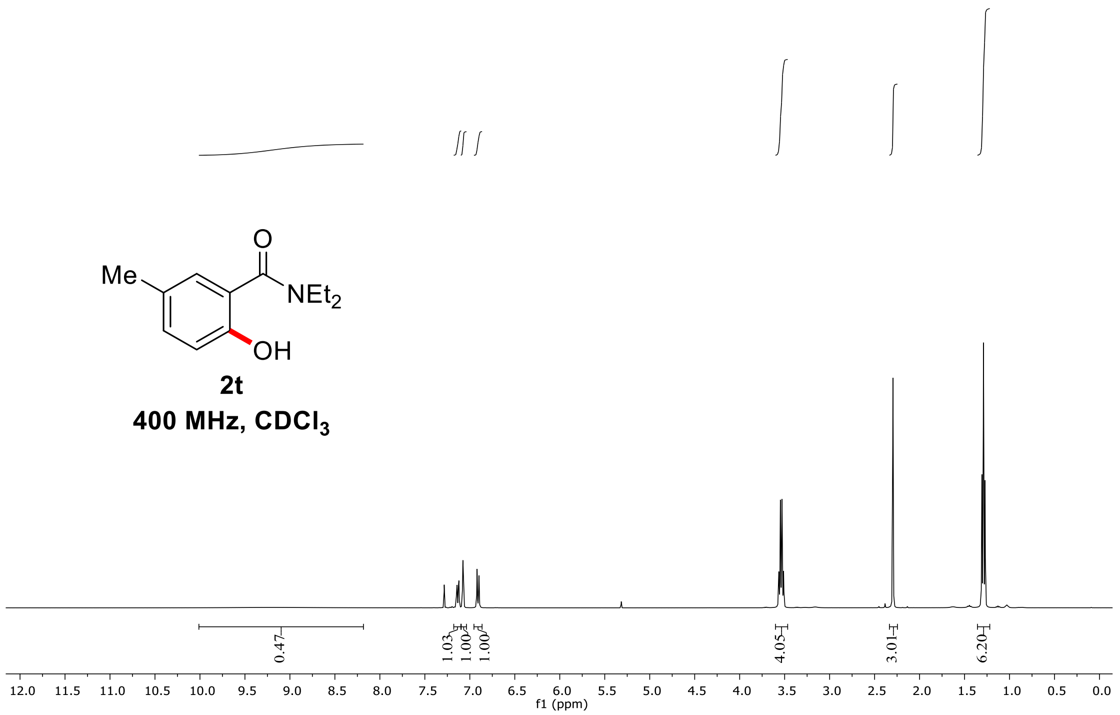
Me
O
NEt$_2$
OH
2t
400 MHz, CDCl$_3$
0.47
1.03
1.00
1.00
4.05
3.01
6.20
f1 (ppm)

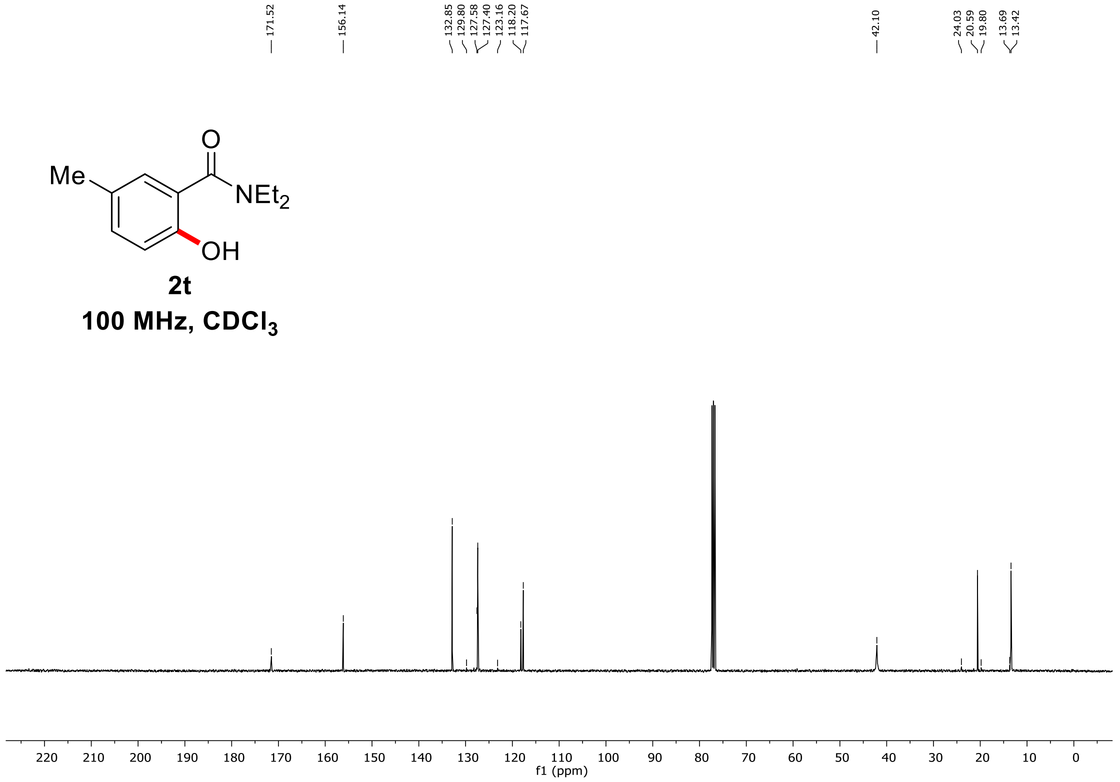
171.52
156.14
132.85
129.80
127.58
127.40
123.16
118.20
117.67
42.10
24.03
20.59
19.80
13.69
13.42
Me
O
NEt$_2$
OH
2t
100 MHz, CDCl$_3$
f1 (ppm)

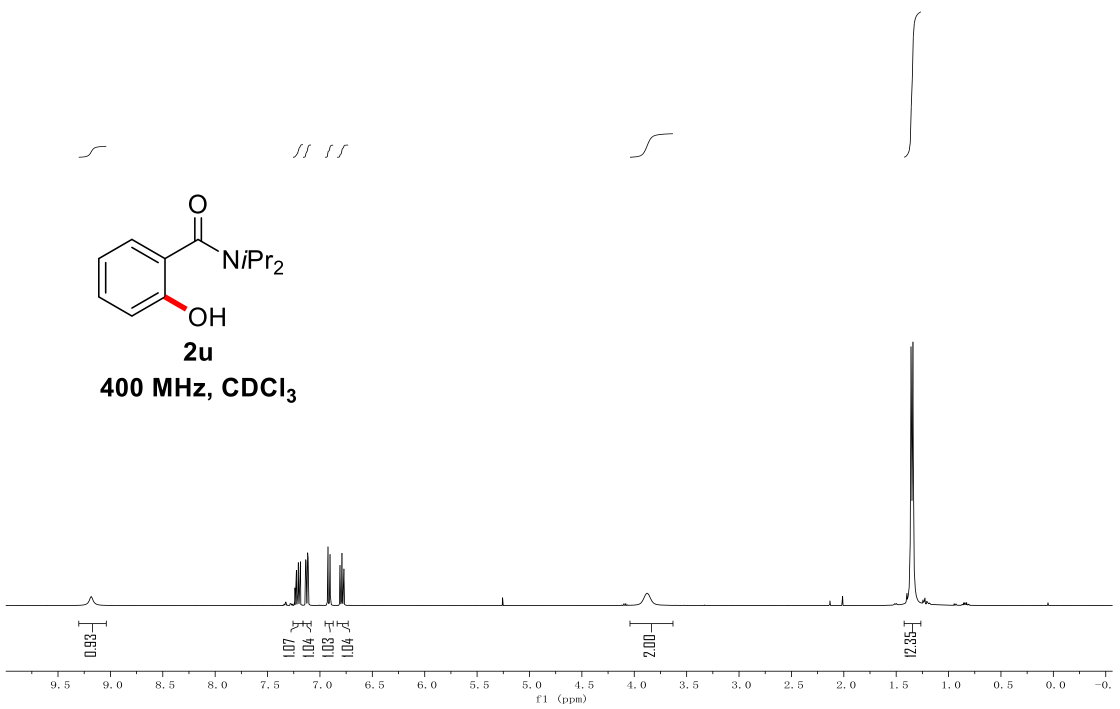

2u

400 MHz, $CDCl_3$

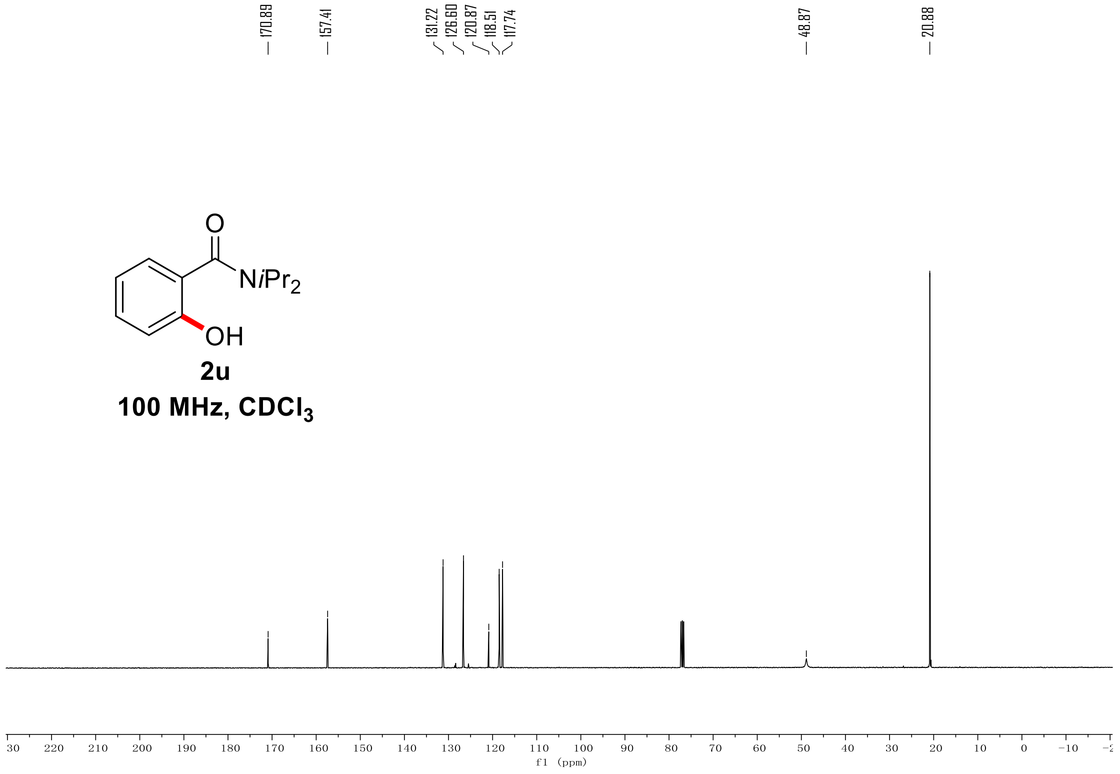

2u

100 MHz, $CDCl_3$

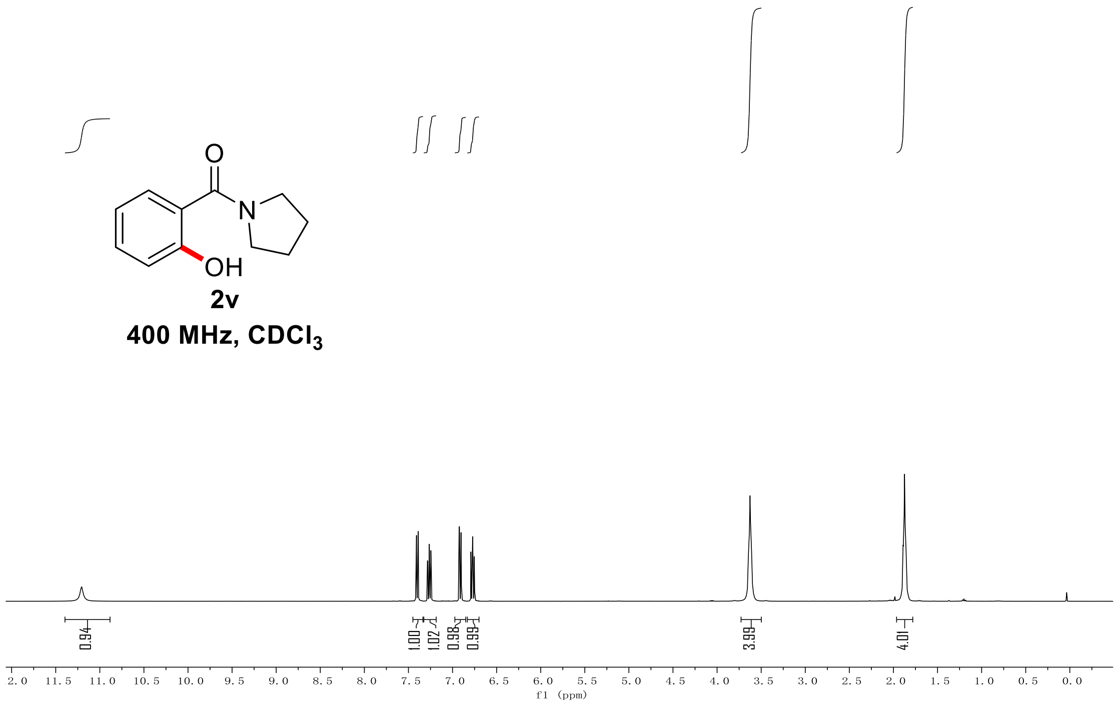
OH
2v
400 MHz, CDCl3
0.94
1.00
1.02
0.98
0.99
3.99
4.01
f1 (ppm)

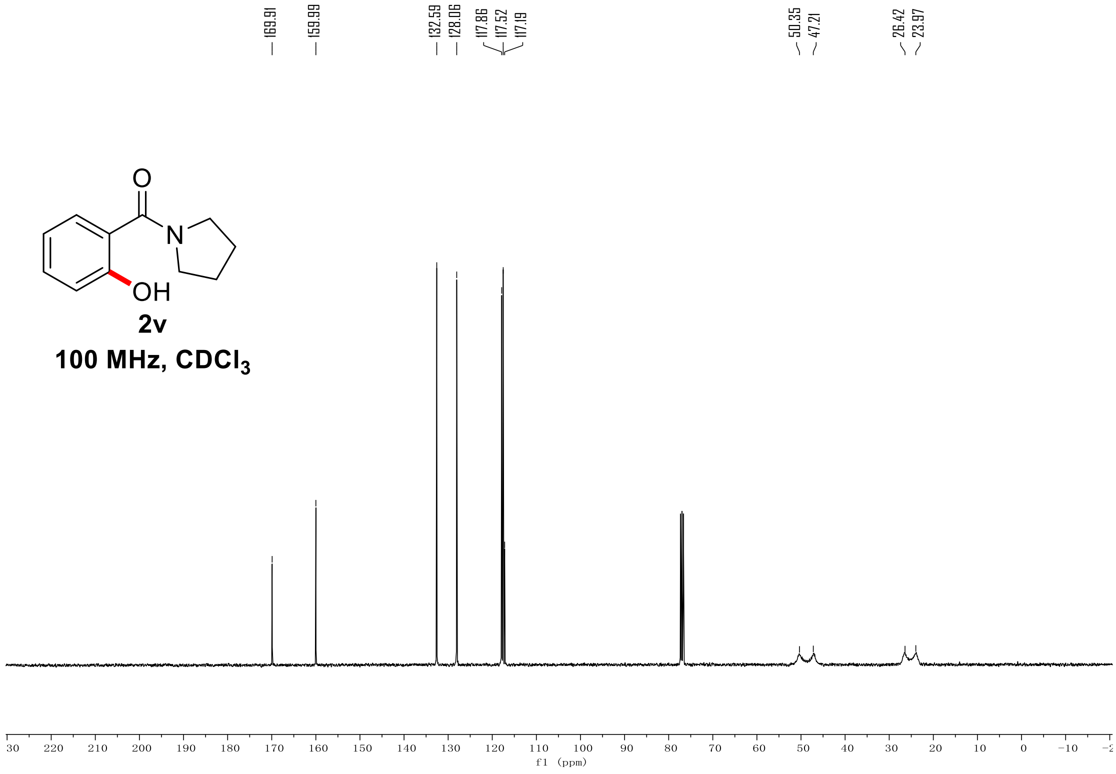
169.91
159.99
132.59
128.06
117.86
117.52
117.19
50.35
47.21
26.42
23.97
OH
2v
100 MHz, CDCl3
f1 (ppm)

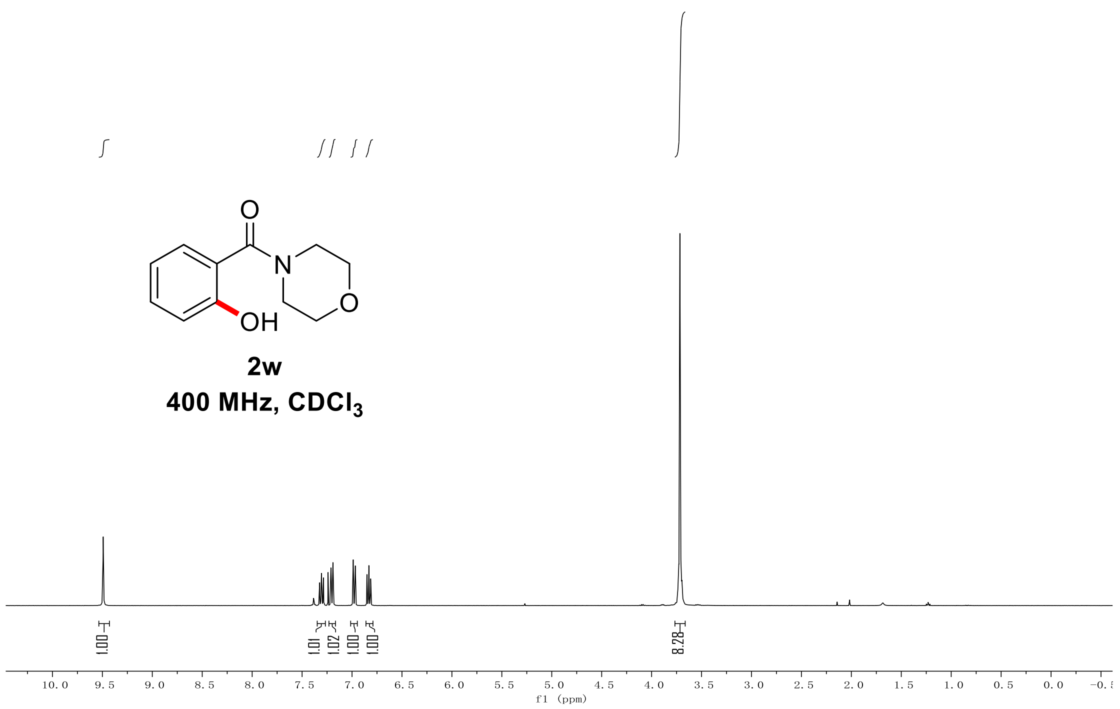
2w
400 MHz, CDCl3
1.00
1.01
1.02
1.00
1.00
8.28
10.0 9.5 9.0 8.5 8.0 7.5 7.0 6.5 6.0 5.5 5.0 4.5 4.0 3.5 3.0 2.5 2.0 1.5 1.0 0.5 0.0
f1 (ppm)

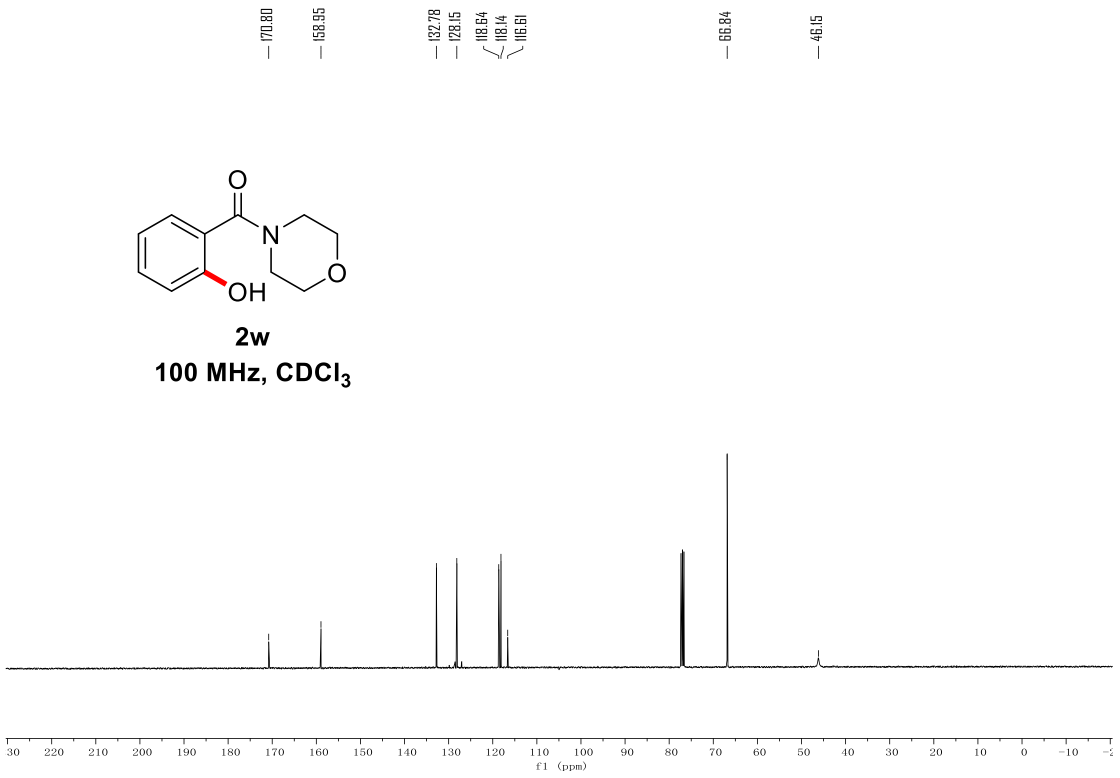
170.80
158.95
132.78
128.15
118.64
118.14
116.61
66.84
46.15
2w
100 MHz, CDCl3
220 210 200 190 180 170 160 150 140 130 120 110 100 90 80 70 60 50 40 30 20 10 0 -10
f1 (ppm)

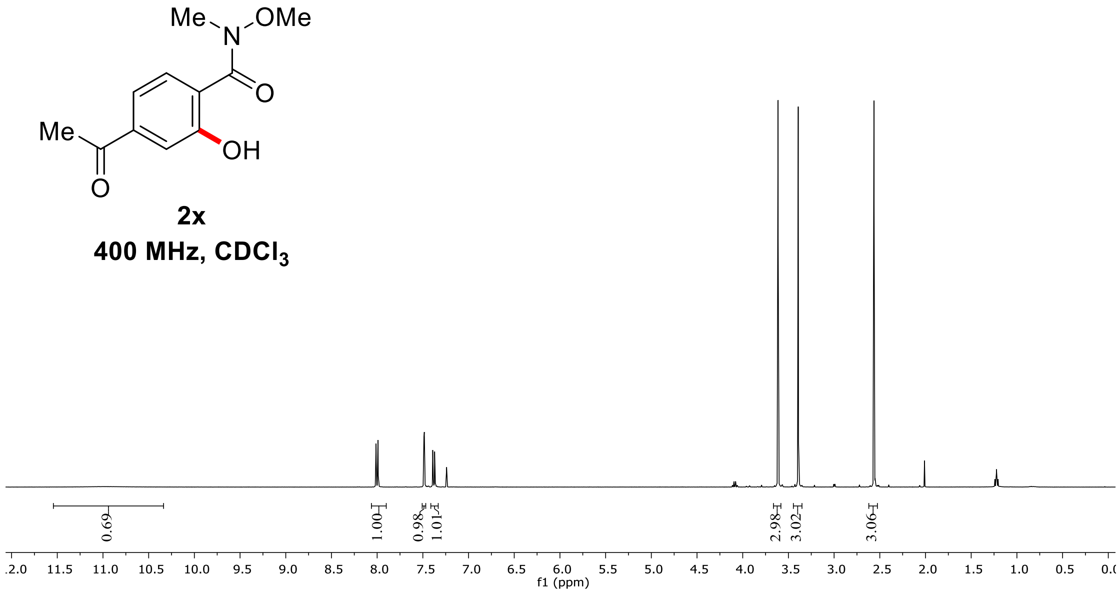

2x
400 MHz, $CDCl_3$

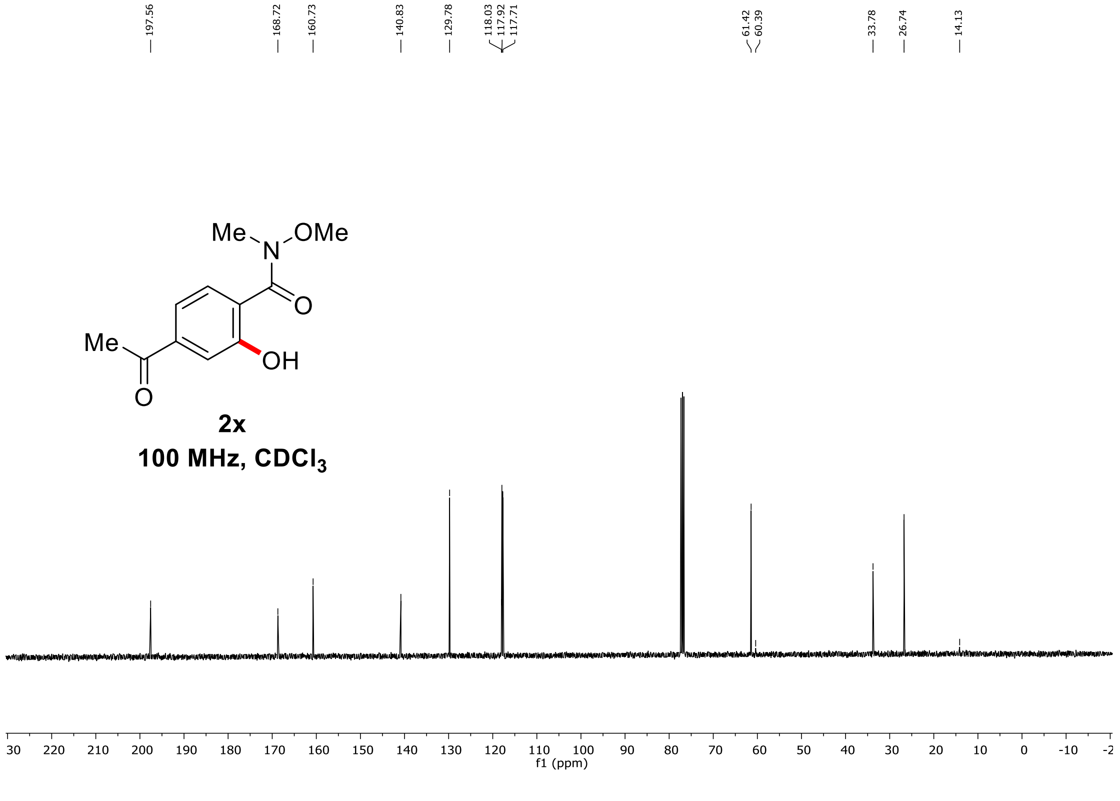

2x
100 MHz, $CDCl_3$

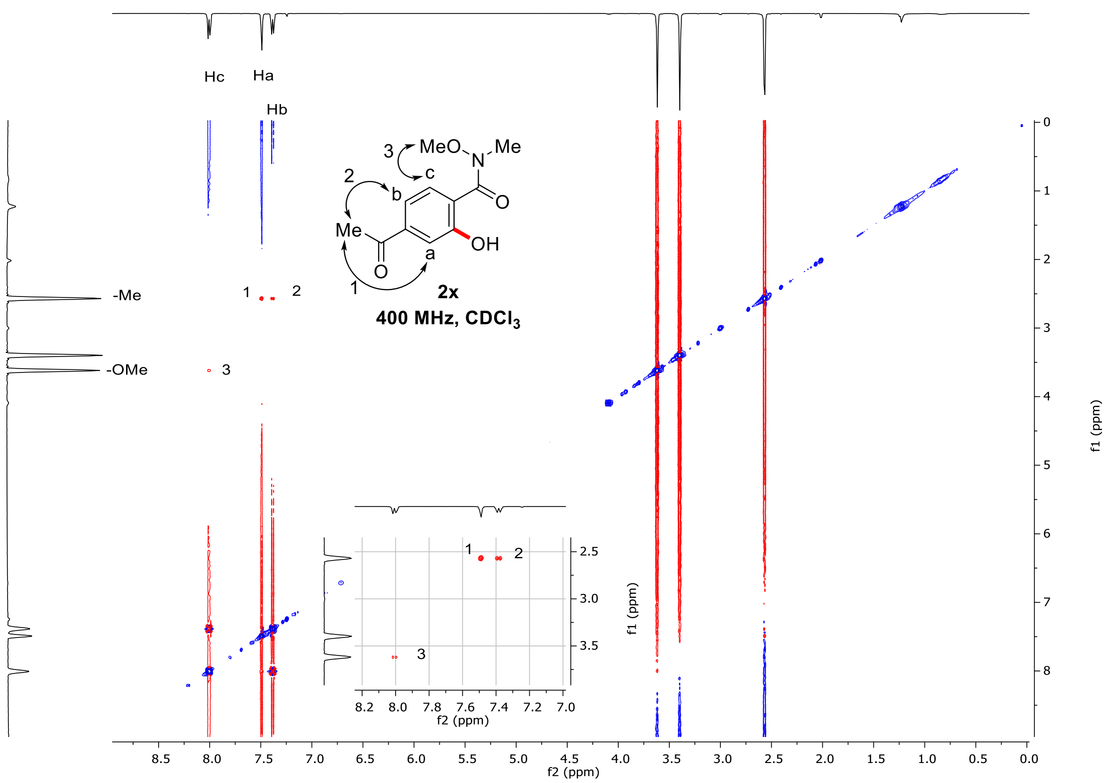

Hc
Ha
Hb
3
MeO
N
Me
c
2
b
O
Me
OH
a
1
O
2x
400 MHz, CDCl3
-Me
-OMe
1
2
3
f1 (ppm)
f2 (ppm)

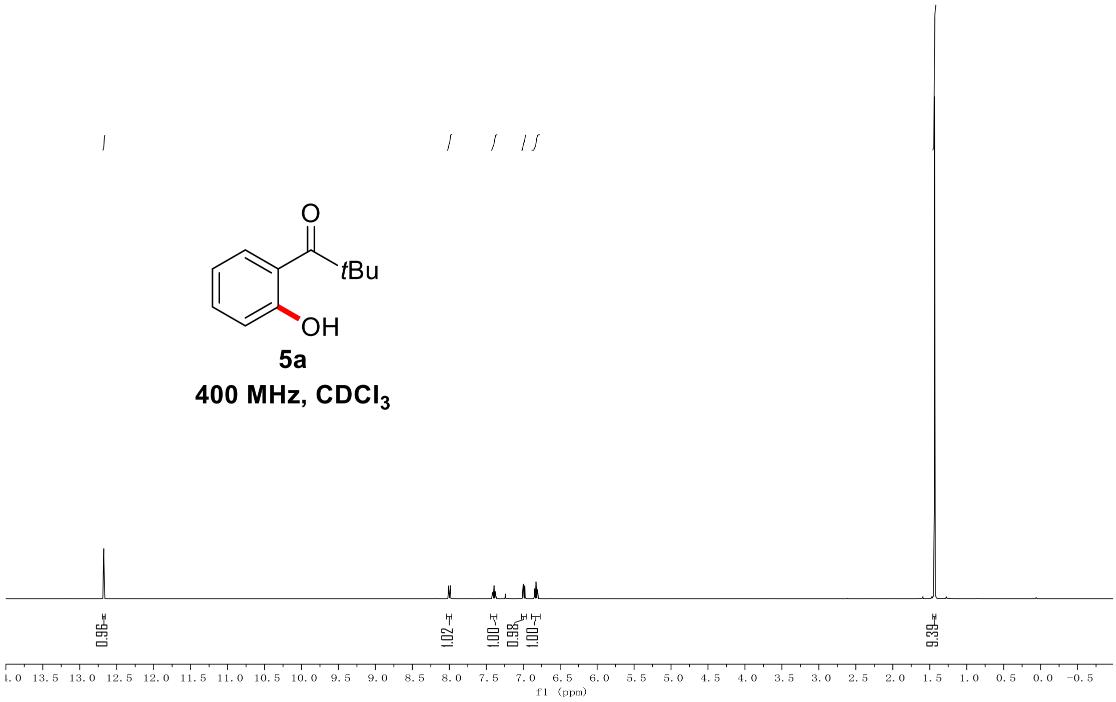
O
tBu
OH
5a
400 MHz, CDCl3
0.96
1.02
1.00
0.98
1.00
9.39
f1 (ppm)

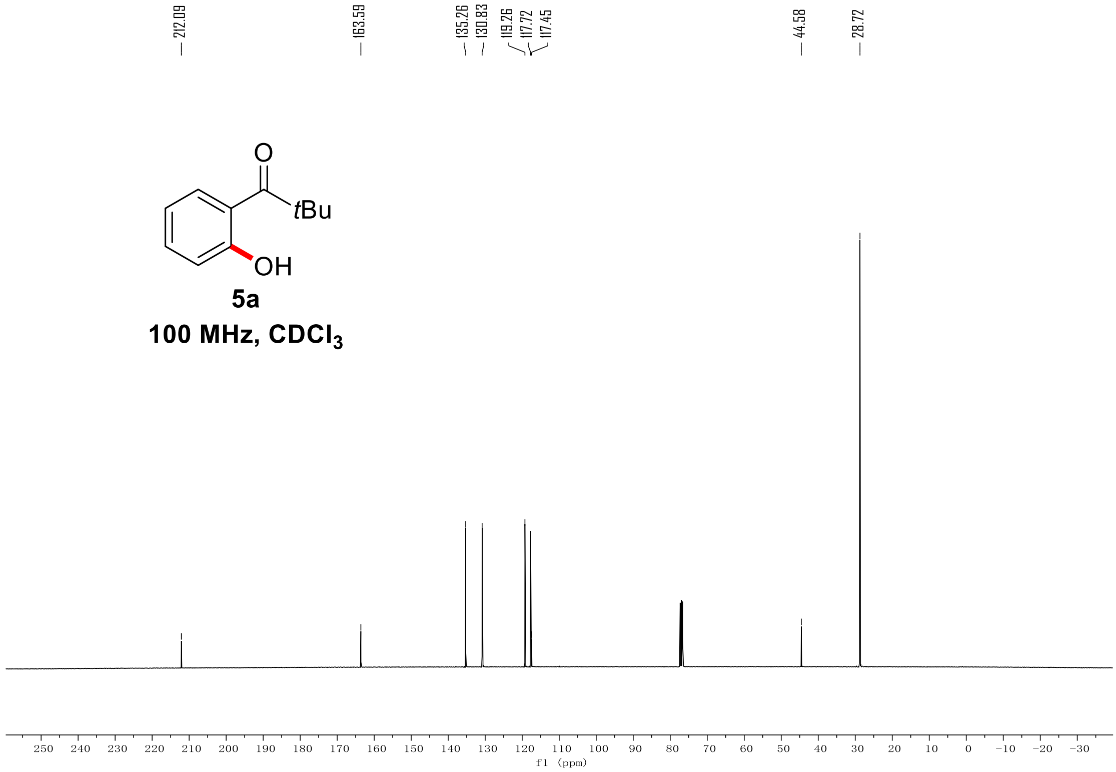
212.09
163.59
135.26
130.83
119.26
117.72
117.45
44.58
28.72
O
tBu
OH
5a
100 MHz, CDCl3
f1 (ppm)

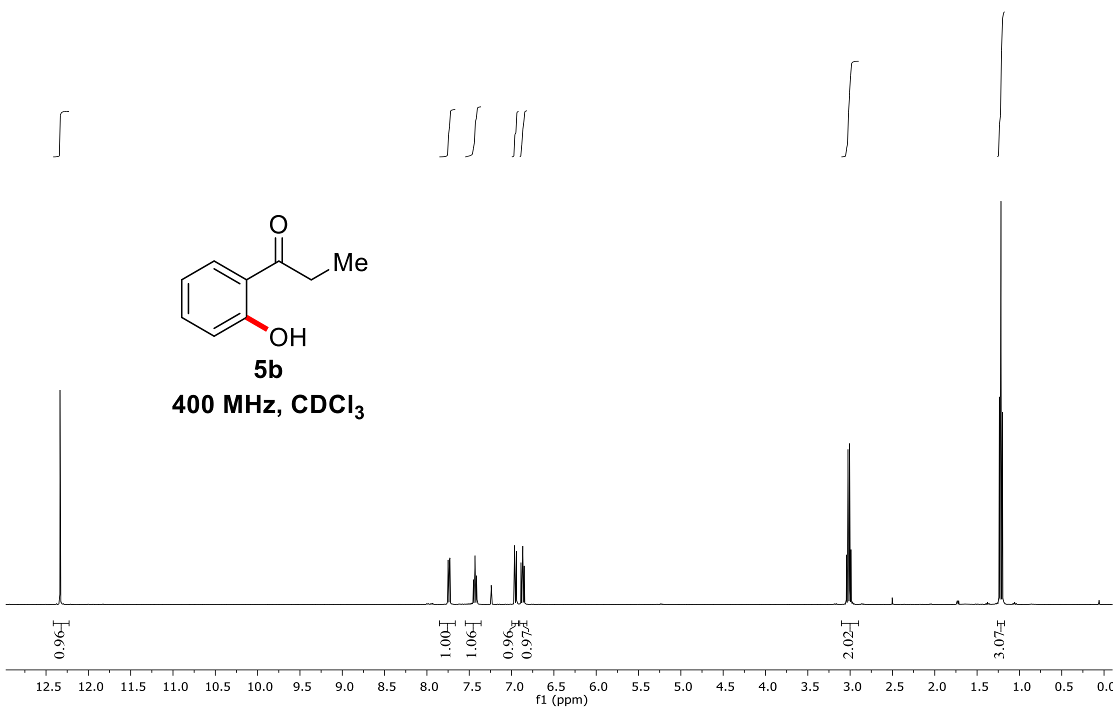

O
Me
OH
5b
400 MHz, CDCl3
0.96
1.00
1.06
0.96
0.97
2.02
3.07
12.5 12.0 11.5 11.0 10.5 10.0 9.5 9.0 8.5 8.0 7.5 7.0 6.5 6.0 5.5 5.0 4.5 4.0 3.5 3.0 2.5 2.0 1.5 1.0 0.5 0.0
f1 (ppm)

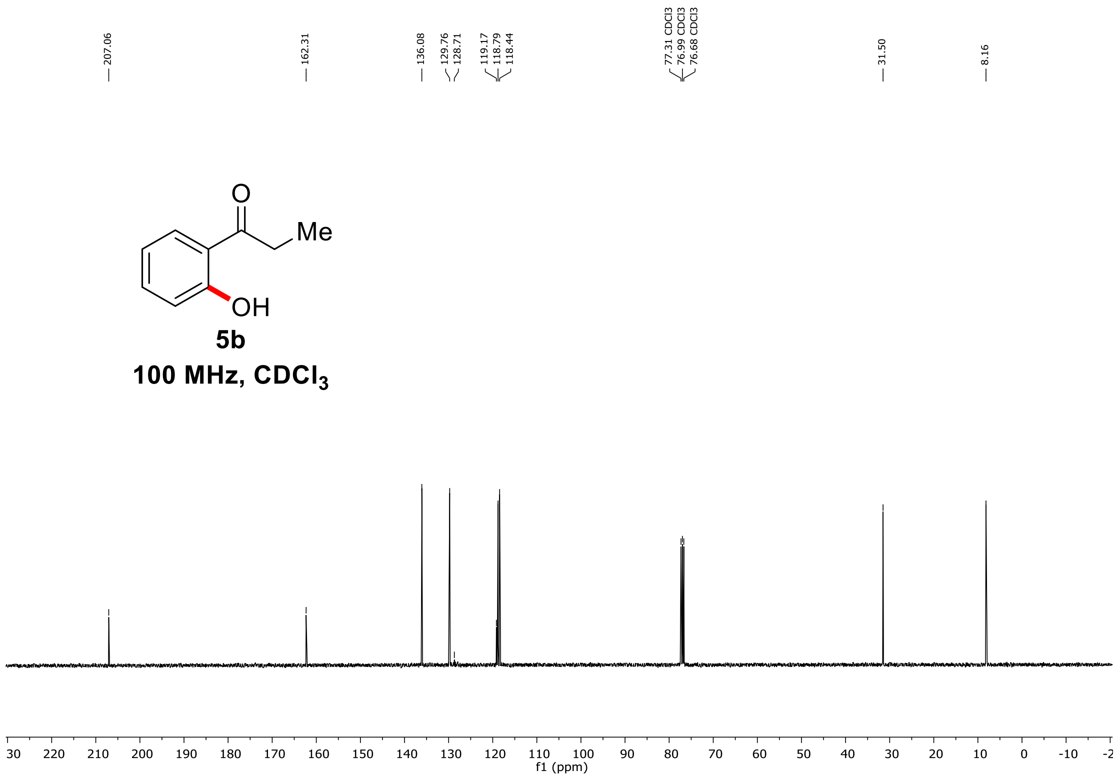

207.06
162.31
136.08
129.76
128.71
119.17
118.79
118.44
77.31 CDCl3
76.99 CDCl3
76.68 CDCl3
31.50
8.16
O
Me
OH
5b
100 MHz, CDCl3
230 220 210 200 190 180 170 160 150 140 130 120 110 100 90 80 70 60 50 40 30 20 10 0 -10 -20
f1 (ppm)

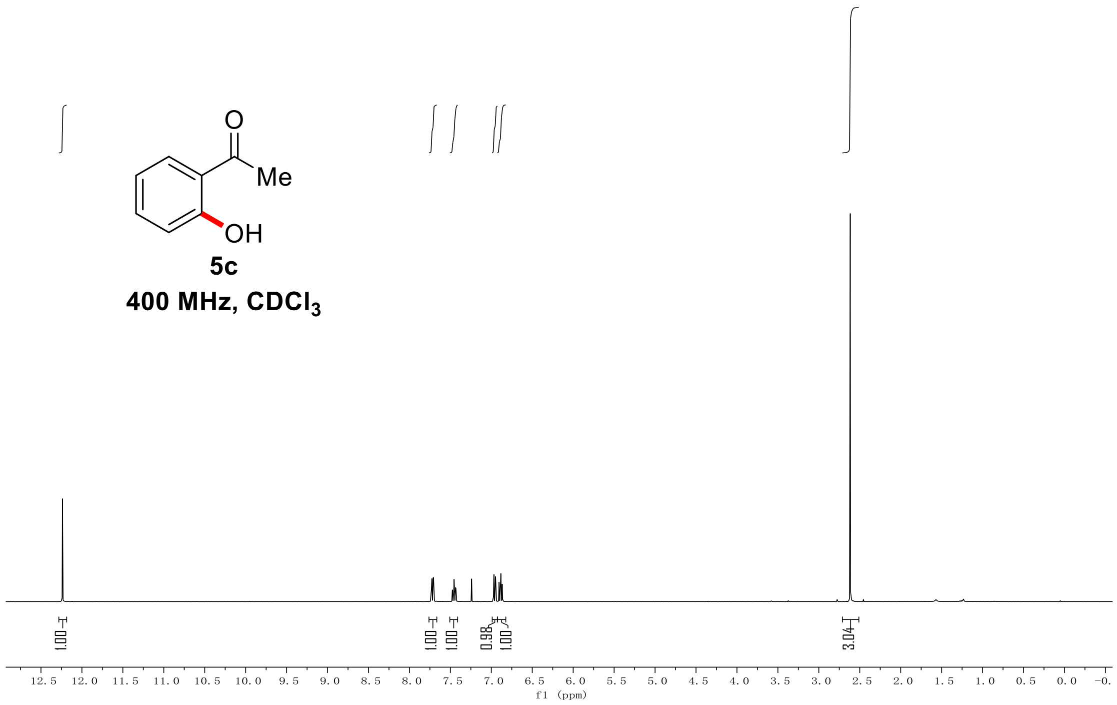

O
Me
OH
5c
400 MHz, CDCl3
1.00
1.00
1.00
0.98
1.00
3.04
12.5 12.0 11.5 11.0 10.5 10.0 9.5 9.0 8.5 8.0 7.5 7.0 6.5 6.0 5.5 5.0 4.5 4.0 3.5 3.0 2.5 2.0 1.5 1.0 0.5 0.0
f1 (ppm)

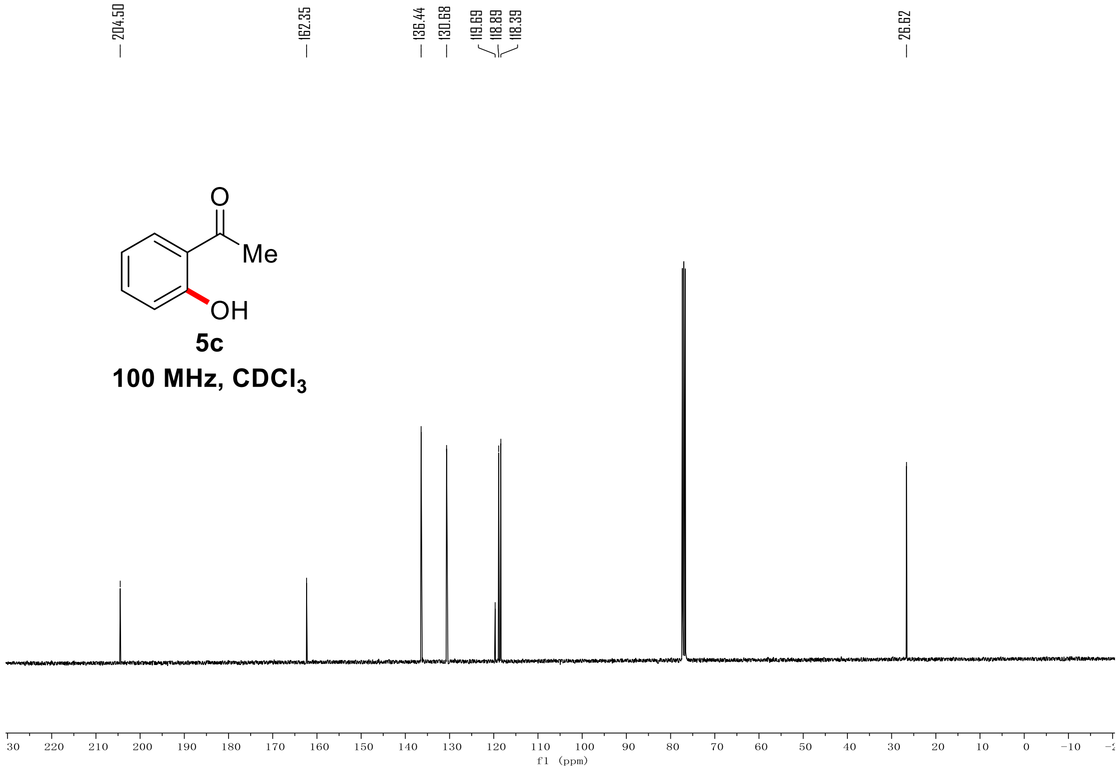

204.50
162.35
136.44
130.68
119.69
118.89
118.39
26.62
O
Me
OH
5c
100 MHz, CDCl3
220 210 200 190 180 170 160 150 140 130 120 110 100 90 80 70 60 50 40 30 20 10 0 −10
f1 (ppm)

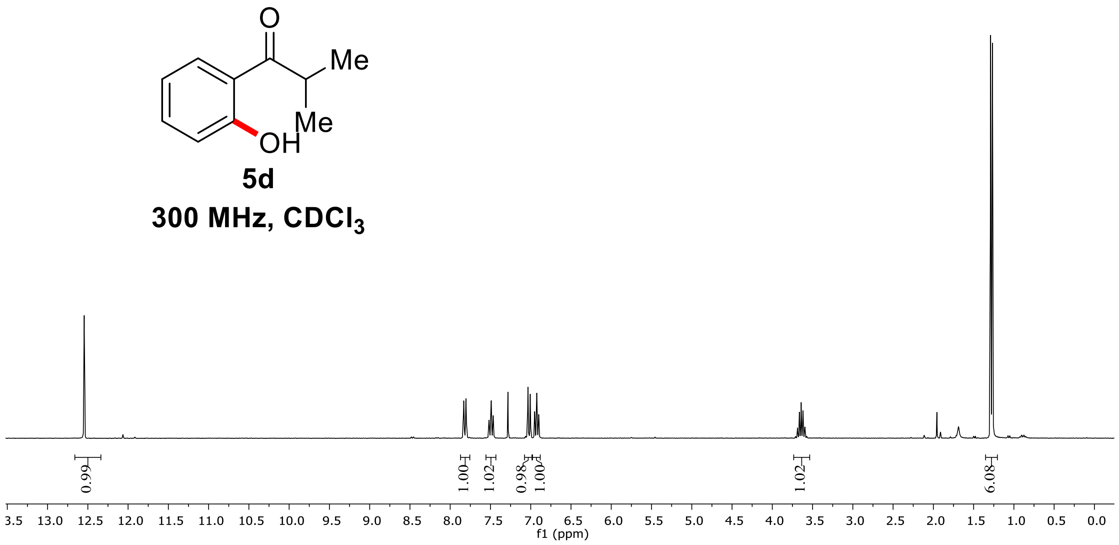

O
Me
Me
OH
5d
300 MHz, CDCl3
0.99
1.00
1.02
0.98
1.00
1.02
6.08
f1 (ppm)

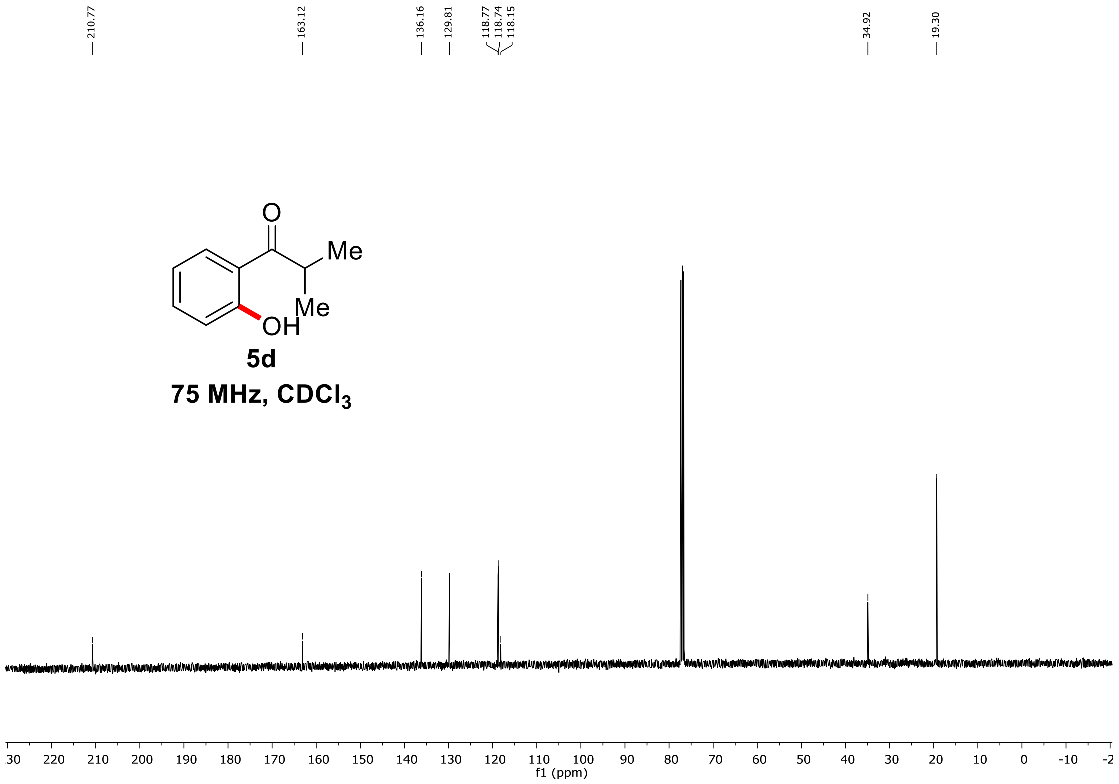

210.77
163.12
136.16
129.81
118.77
118.74
118.15
34.92
19.30
O
Me
Me
OH
5d
75 MHz, CDCl3
f1 (ppm)

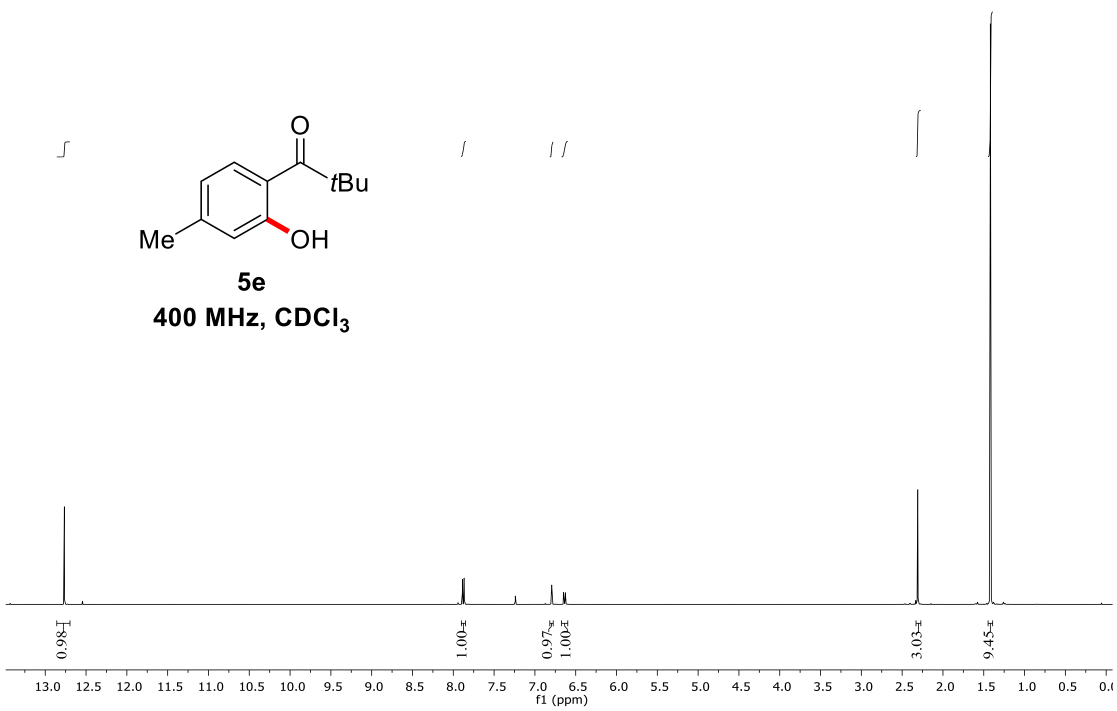

O
tBu
Me
OH
5e
400 MHz, CDCl3
0.98
1.00
0.97
1.00
3.03
9.45
13.0 12.5 12.0 11.5 11.0 10.5 10.0 9.5 9.0 8.5 8.0 7.5 7.0 6.5 6.0 5.5 5.0 4.5 4.0 3.5 3.0 2.5 2.0 1.5 1.0 0.5
f1 (ppm)

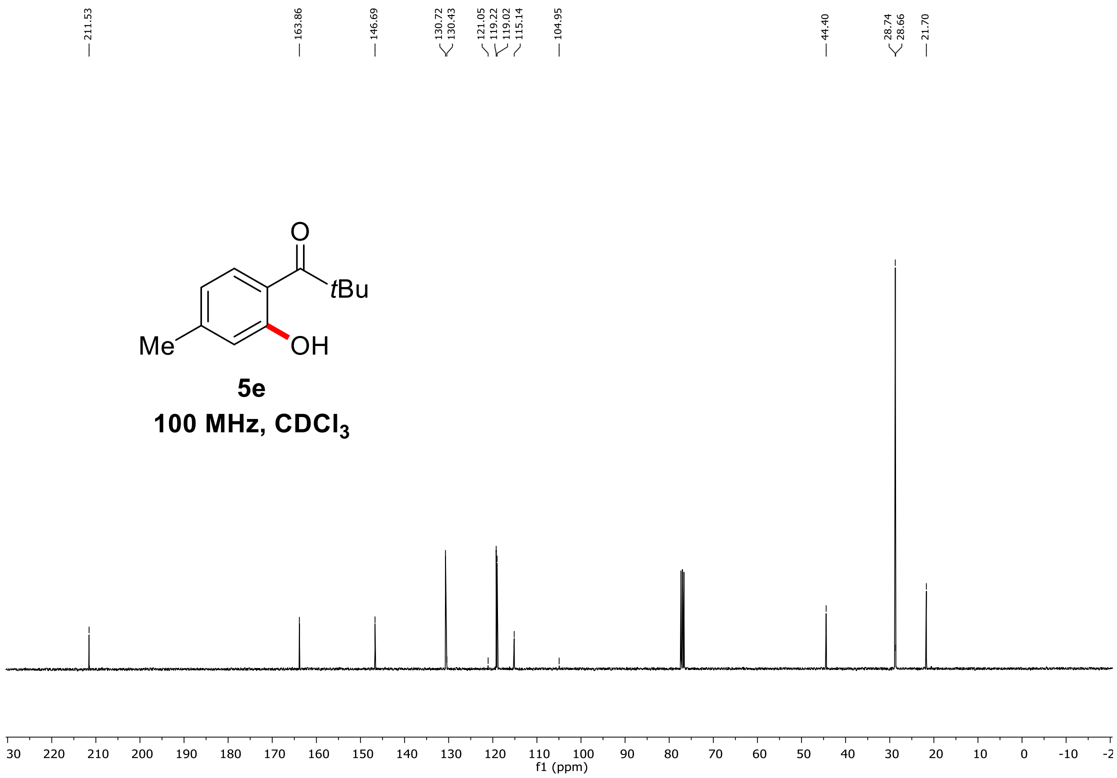

211.53
163.86
146.69
130.72
130.43
121.05
119.22
119.02
115.14
104.95
44.40
28.74
28.66
21.70
O
tBu
Me
OH
5e
100 MHz, CDCl3
220 210 200 190 180 170 160 150 140 130 120 110 100 90 80 70 60 50 40 30 20 10 0 -10
f1 (ppm)

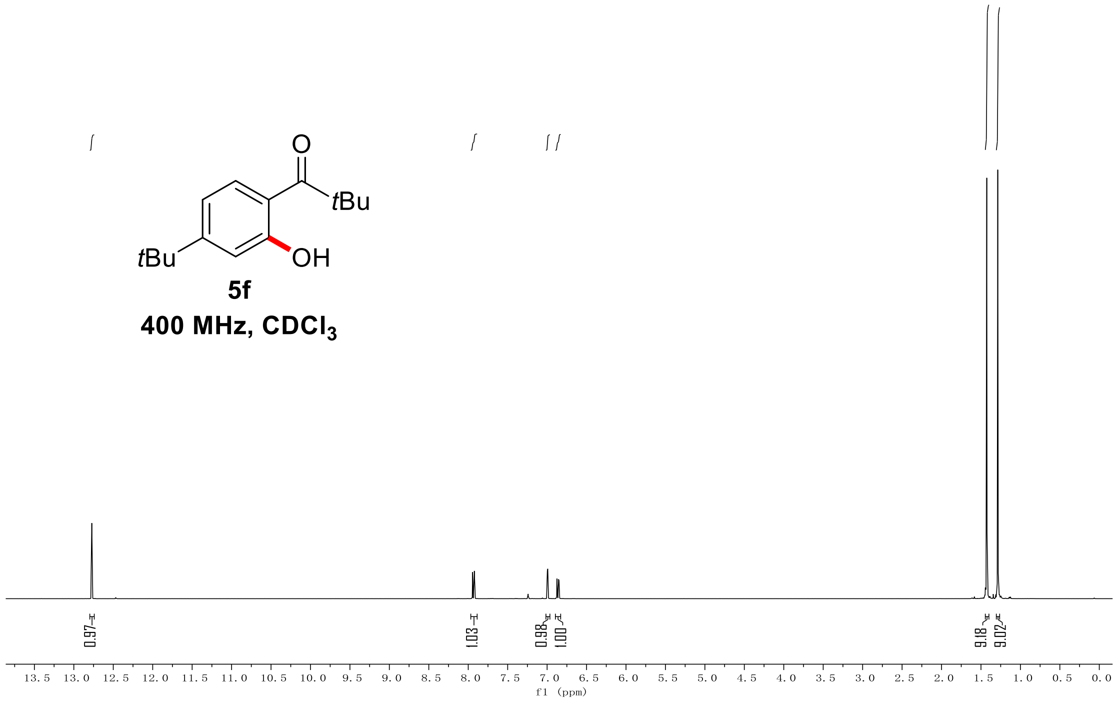

O
tBu
tBu
OH
5f
400 MHz, CDCl$_3$
0.97
1.03
0.98
1.00
9.18
9.02
13.5 13.0 12.5 12.0 11.5 11.0 10.5 10.0 9.5 9.0 8.5 8.0 7.5 7.0 6.5 6.0 5.5 5.0 4.5 4.0 3.5 3.0 2.5 2.0 1.5 1.0 0.5 0.0
f1 (ppm)

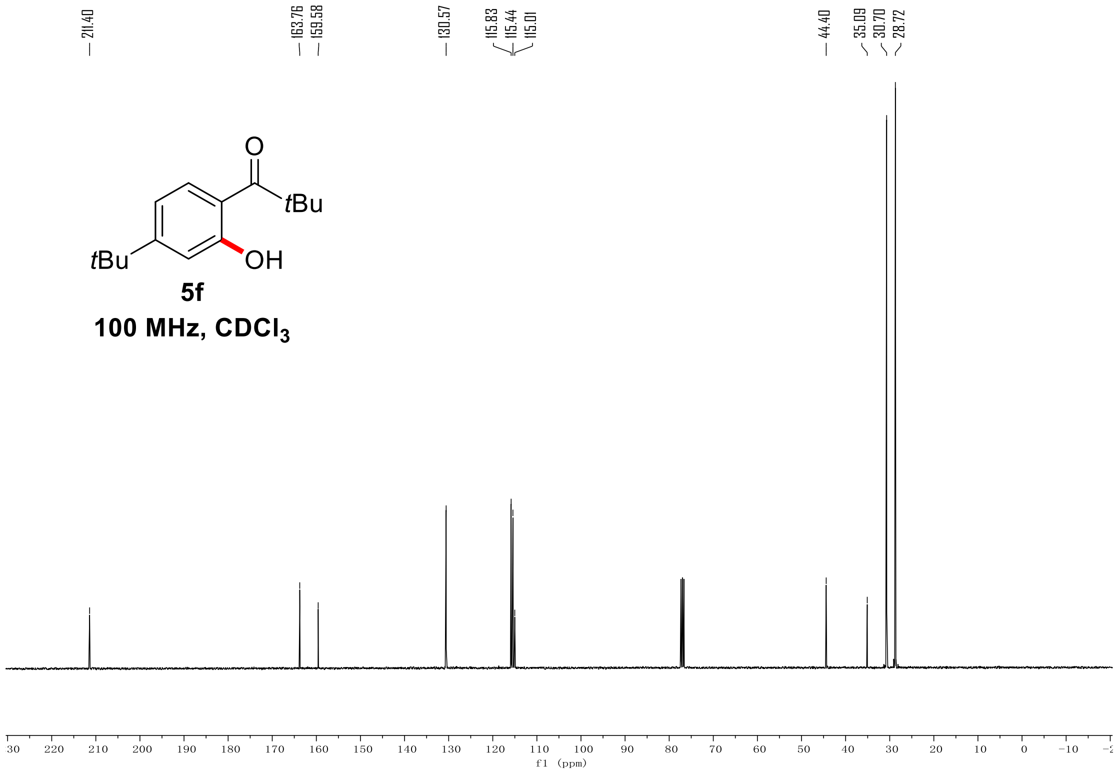

211.40
163.76
159.58
130.57
115.83
115.44
115.01
44.40
35.09
30.70
28.72
O
tBu
tBu
OH
5f
100 MHz, CDCl$_3$
220 210 200 190 180 170 160 150 140 130 120 110 100 90 80 70 60 50 40 30 20 10 0 -10
f1 (ppm)

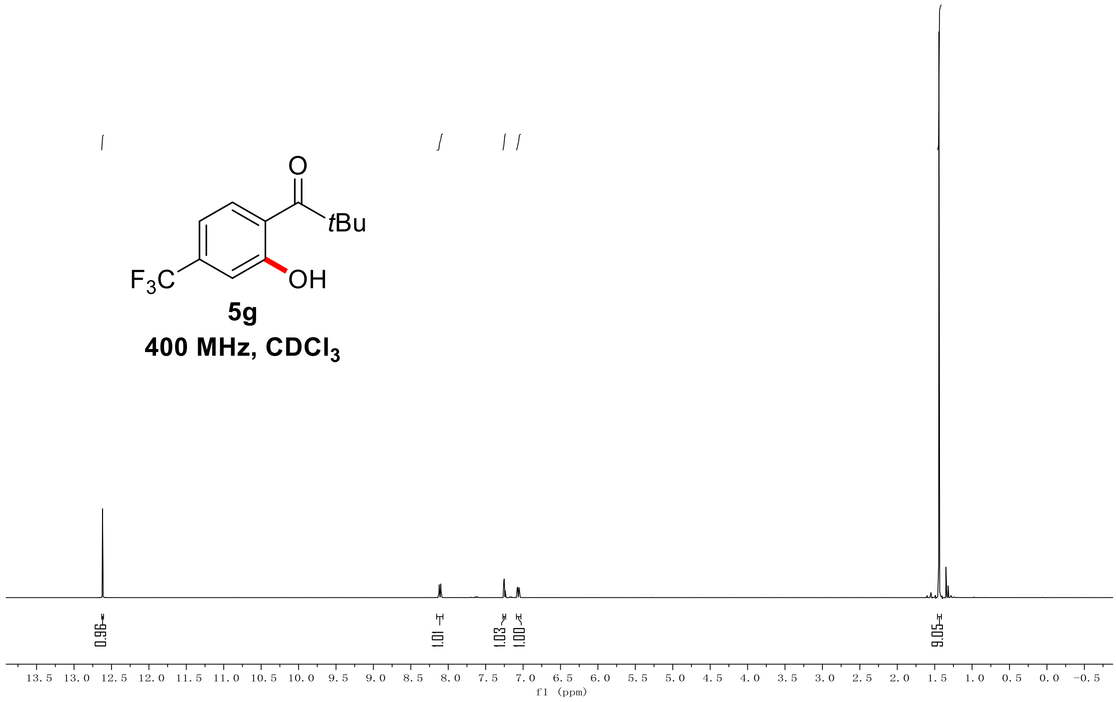
O
tBu
F3C
OH
5g
400 MHz, CDCl3
0.96
1.01
1.03
1.00
9.05
f1 (ppm)

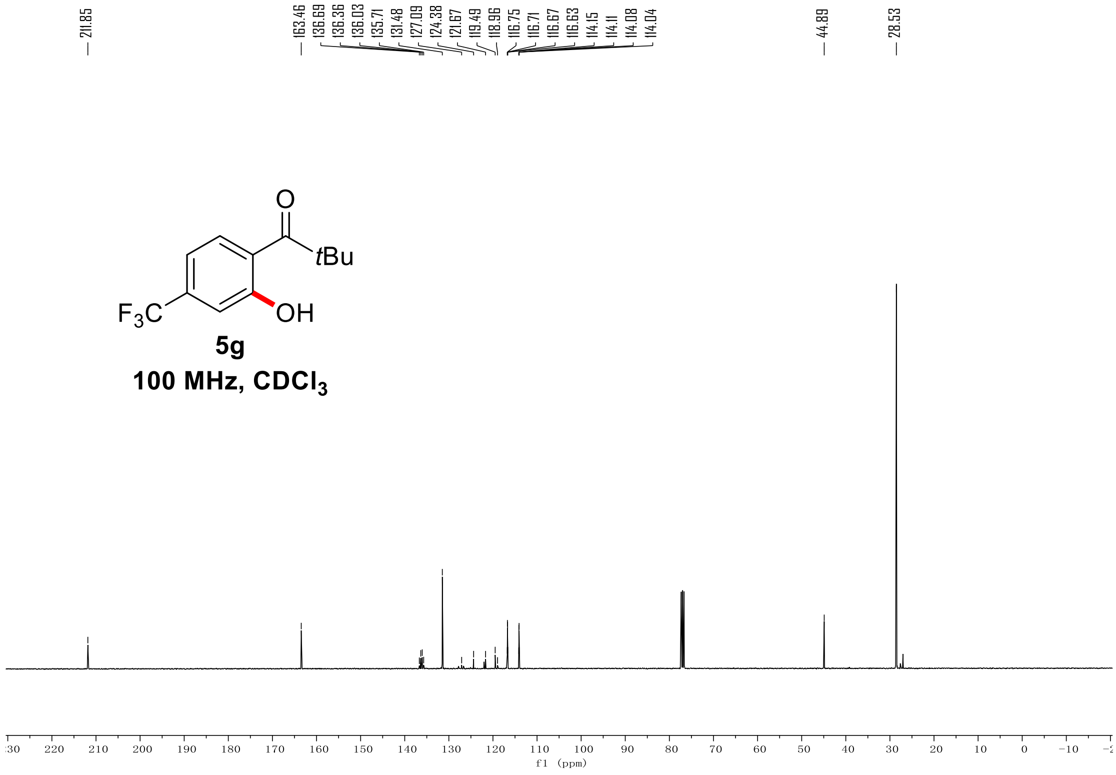
211.85
163.46
136.69
136.36
136.03
135.71
131.48
127.09
124.38
121.67
119.49
118.96
116.75
116.71
116.67
116.63
114.15
114.11
114.08
114.04
44.89
28.53
O
tBu
F3C
OH
5g
100 MHz, CDCl3
f1 (ppm)

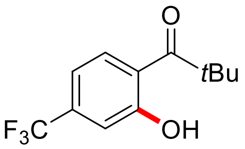

5g

376 MHz, $CDCl_3$

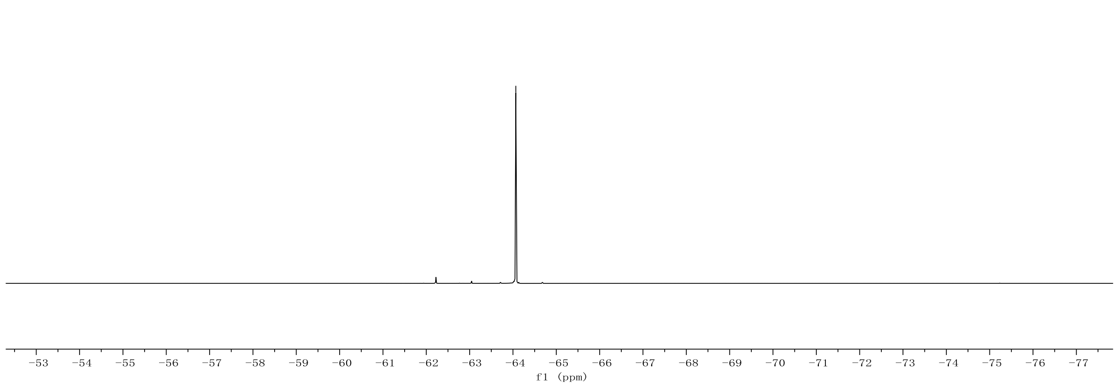

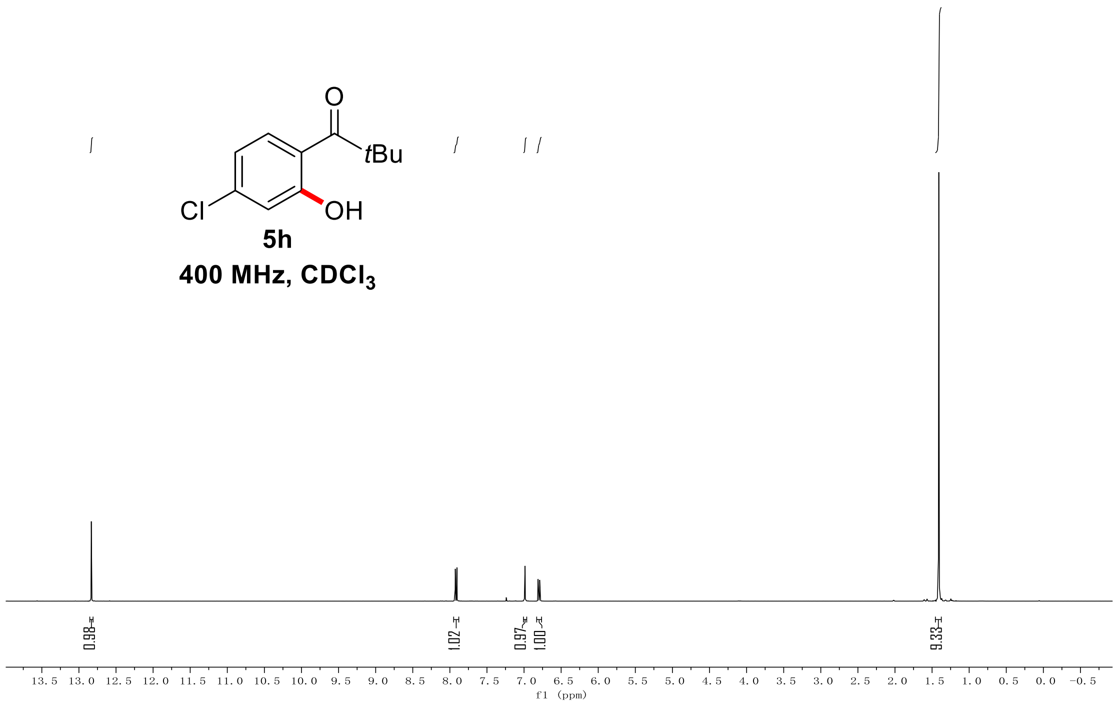
O
tBu
Cl
OH
5h
400 MHz, CDCl3
0.98
1.02
0.97
1.00
9.33
13.5 13.0 12.5 12.0 11.5 11.0 10.5 10.0 9.5 9.0 8.5 8.0 7.5 7.0 6.5 6.0 5.5 5.0 4.5 4.0 3.5 3.0 2.5 2.0 1.5 1.0 0.5 0.0 -0.5
f1 (ppm)

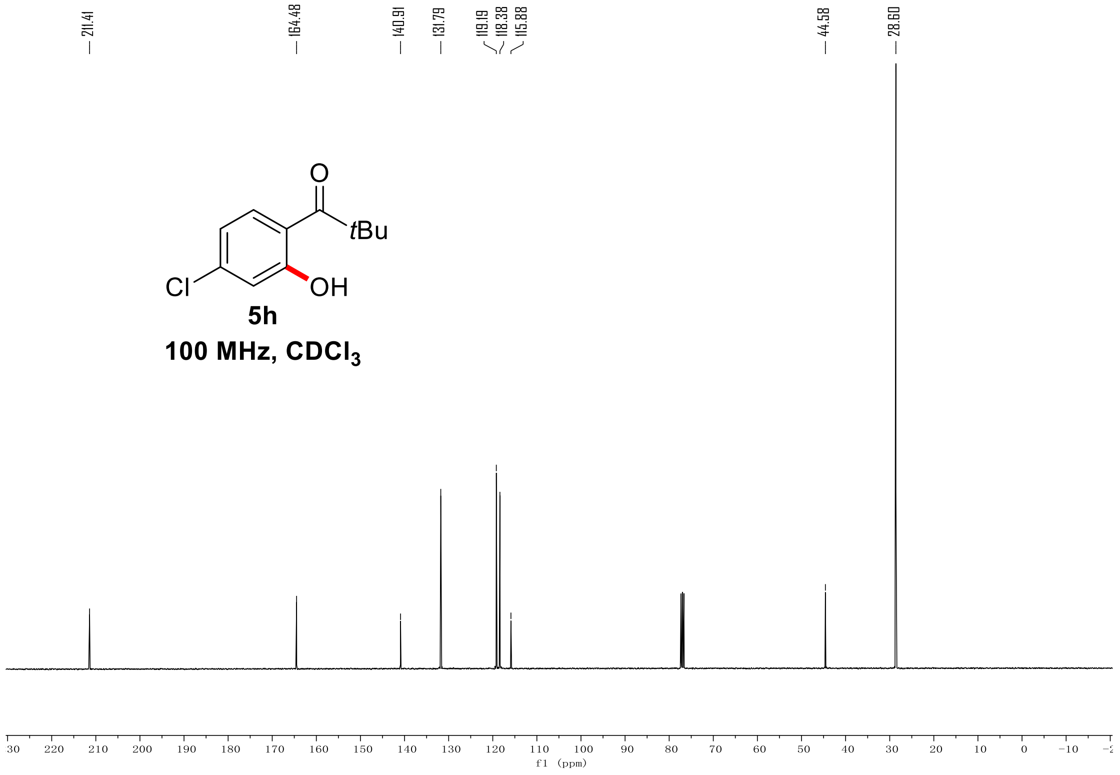
211.41
164.48
140.91
131.79
119.19
118.38
115.88
44.58
28.60
O
tBu
Cl
OH
5h
100 MHz, CDCl3
220 210 200 190 180 170 160 150 140 130 120 110 100 90 80 70 60 50 40 30 20 10 0 -10
f1 (ppm)

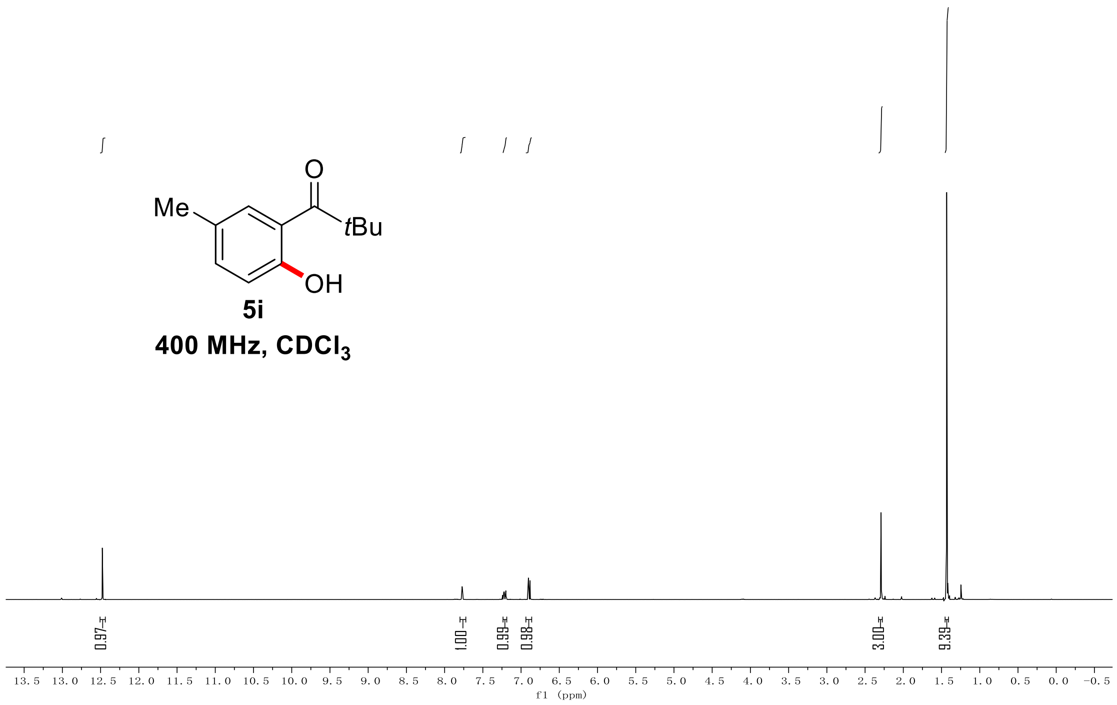
Me
O
tBu
OH
5i
400 MHz, CDCl3
0.97
1.00
0.99
0.98
3.00
9.39
13.5 13.0 12.5 12.0 11.5 11.0 10.5 10.0 9.5 9.0 8.5 8.0 7.5 7.0 6.5 6.0 5.5 5.0 4.5 4.0 3.5 3.0 2.5 2.0 1.5 1.0 0.5 0.0 -0.5
f1 (ppm)

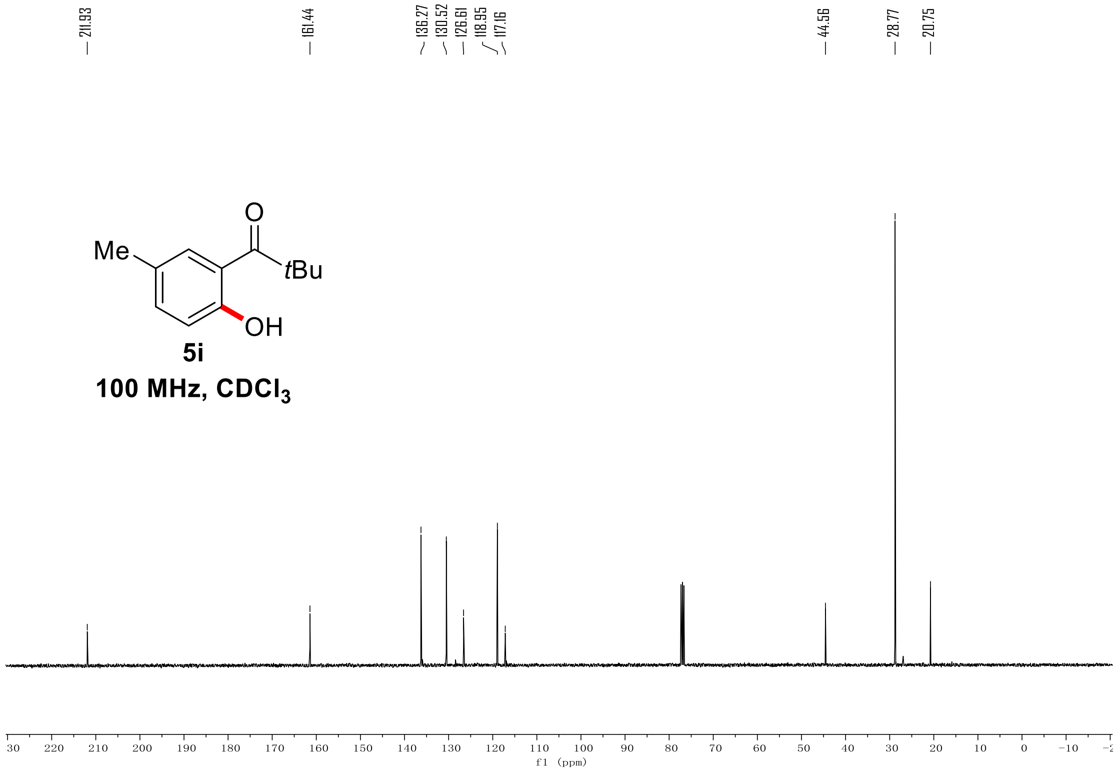
211.93
161.44
136.27
130.52
126.61
118.95
117.16
44.56
28.77
20.75
Me
O
tBu
OH
5i
100 MHz, CDCl3
30 220 210 200 190 180 170 160 150 140 130 120 110 100 90 80 70 60 50 40 30 20 10 0 -10
f1 (ppm)

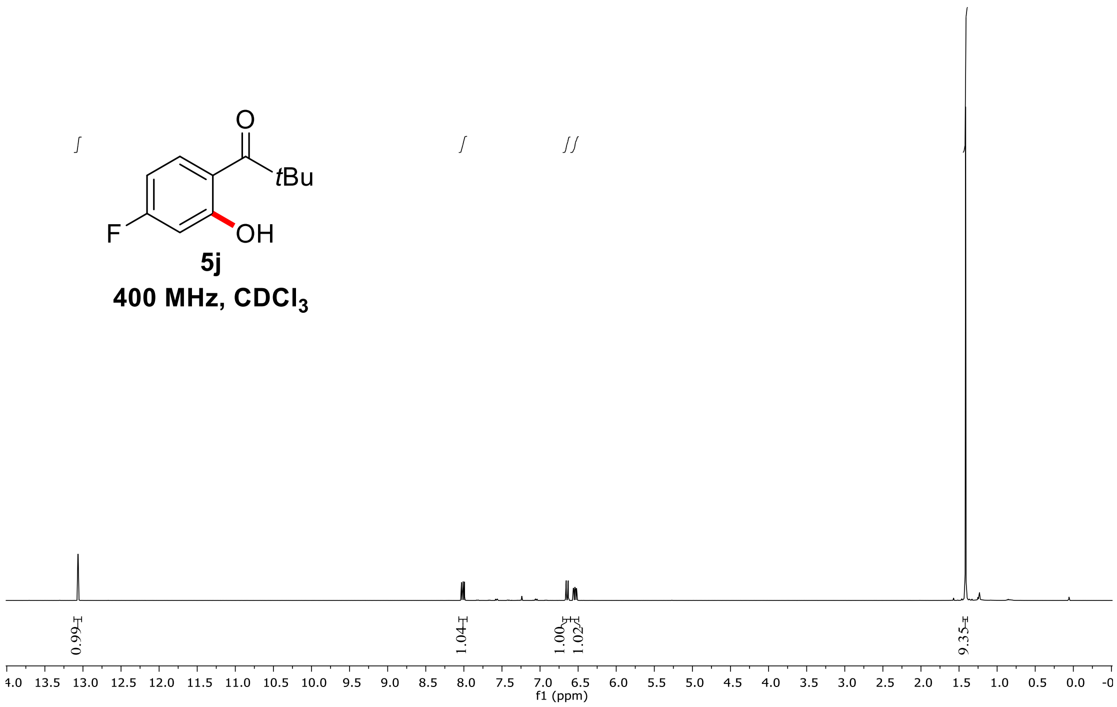
O
tBu
F
OH
5j
400 MHz, $CDCl_3$
0.99
1.04
1.00
1.02
9.35
f1 (ppm)

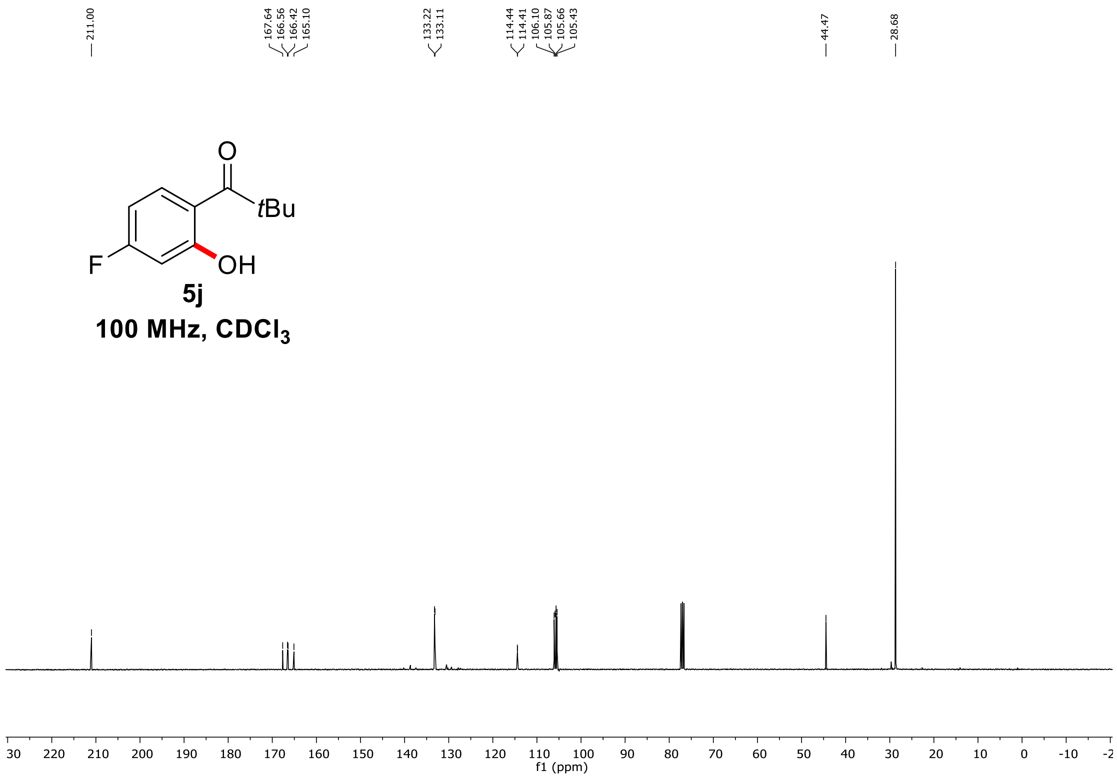
211.00
167.64
166.56
166.42
165.10
133.22
133.11
114.44
114.41
106.10
105.87
105.66
105.43
44.47
28.68
O
tBu
F
OH
5j
100 MHz, $CDCl_3$
f1 (ppm)

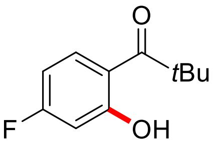

5j

376 MHz, $CDCl_3$

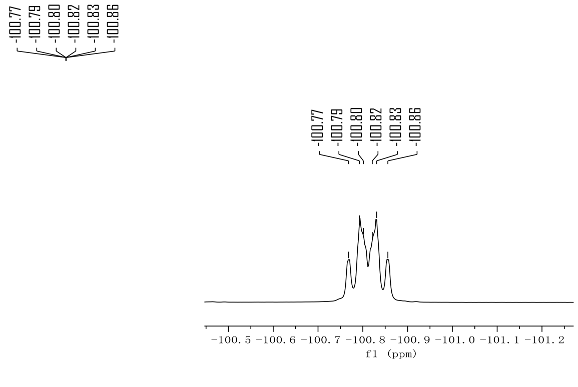

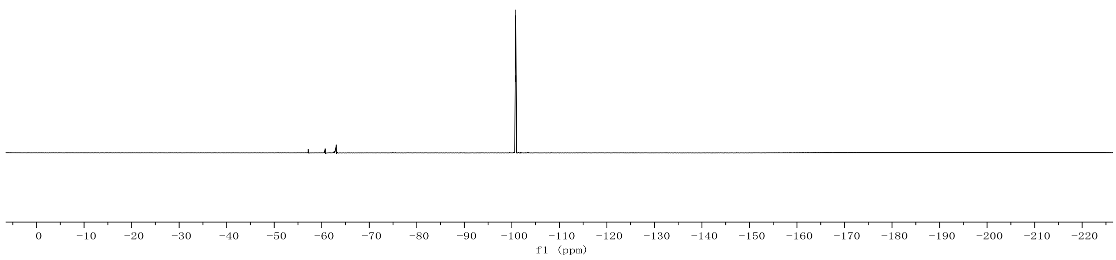

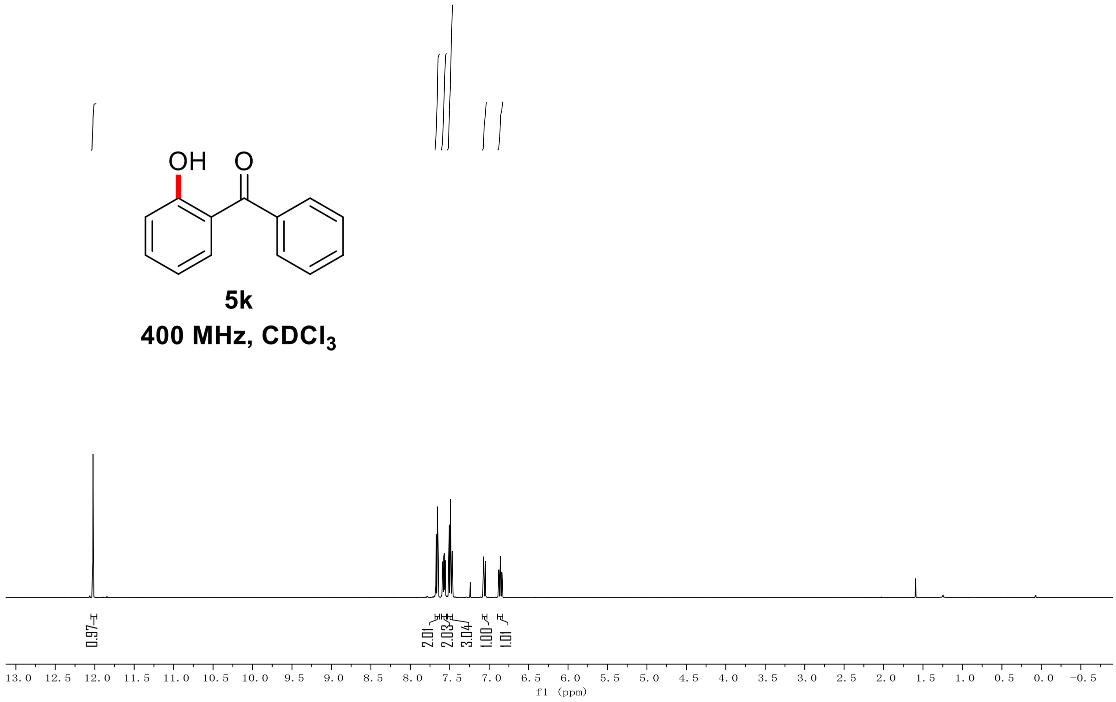

OH O
5k
400 MHz, CDCl3
0.97
2.01
2.03
3.04
1.00
1.01
13.0 12.5 12.0 11.5 11.0 10.5 10.0 9.5 9.0 8.5 8.0 7.5 7.0 6.5 6.0 5.5 5.0 4.5 4.0 3.5 3.0 2.5 2.0 1.5 1.0 0.5 0.0 -0.5
f1 (ppm)

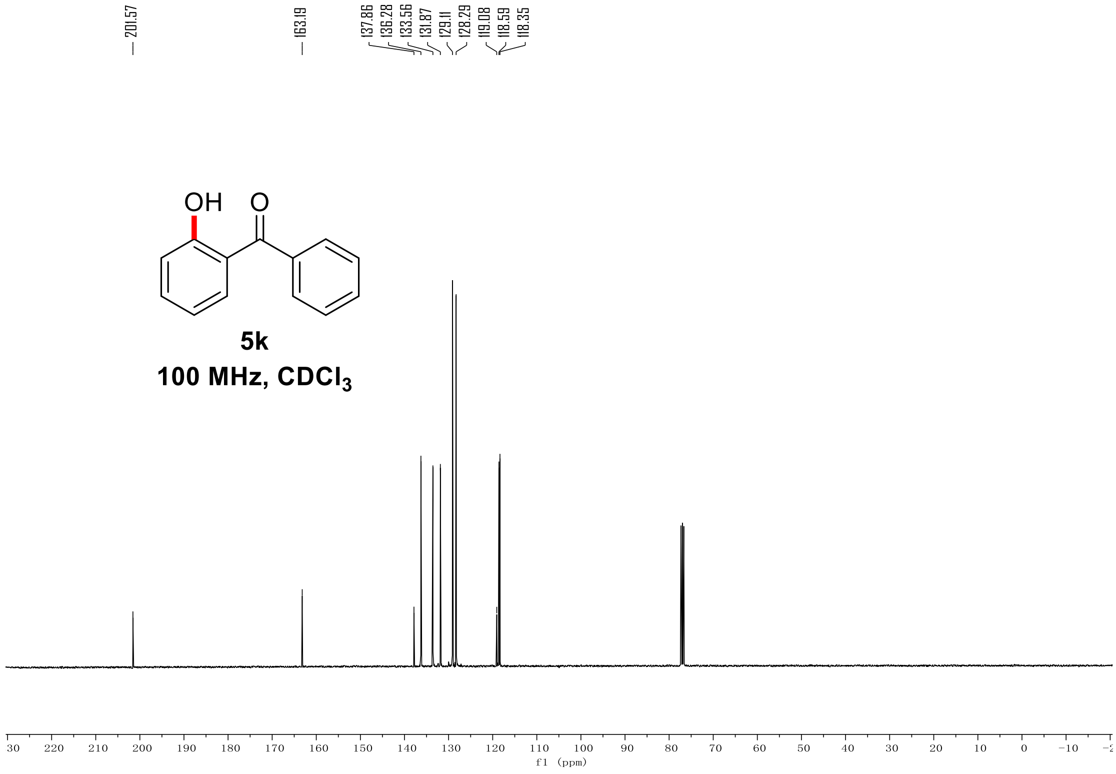

201.57
163.19
137.86
136.28
133.56
131.87
129.11
128.29
119.08
118.59
118.35
OH O
5k
100 MHz, CDCl3
220 210 200 190 180 170 160 150 140 130 120 110 100 90 80 70 60 50 40 30 20 10 0 -10
f1 (ppm)

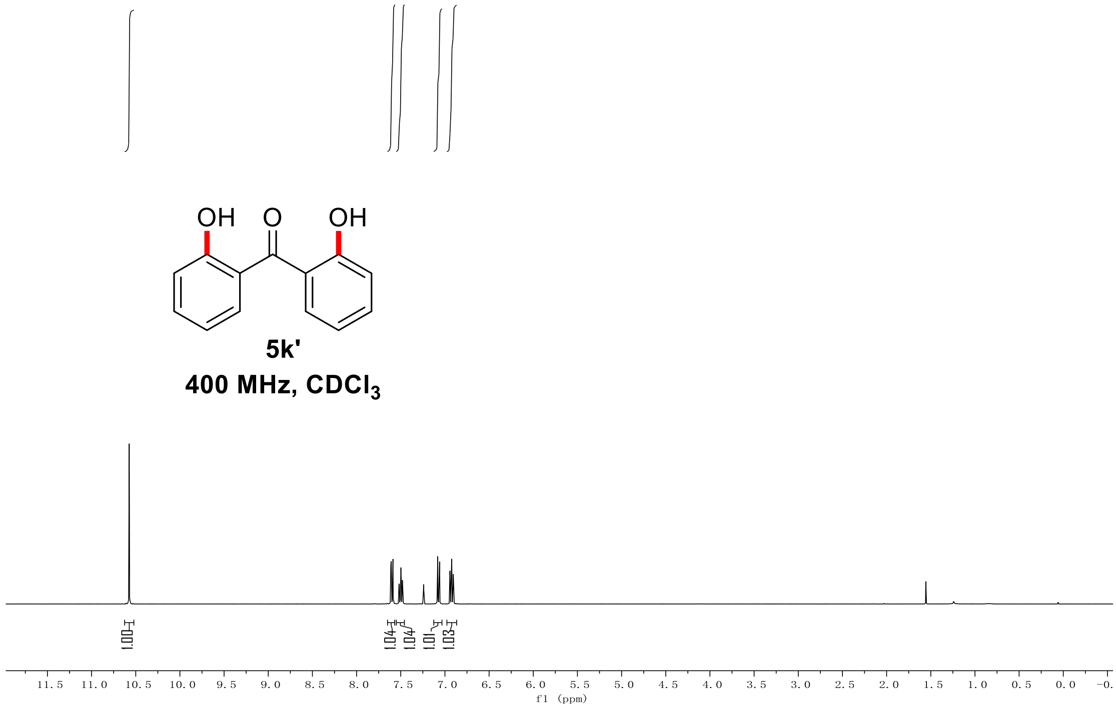
OH
O
OH
5k'
400 MHz, CDCl3
1.00
1.04
1.04
1.01
1.03
11.5
11.0
10.5
10.0
9.5
9.0
8.5
8.0
7.5
7.0
6.5
6.0
5.5
5.0
4.5
4.0
3.5
3.0
2.5
2.0
1.5
1.0
0.5
0.0
f1 (ppm)

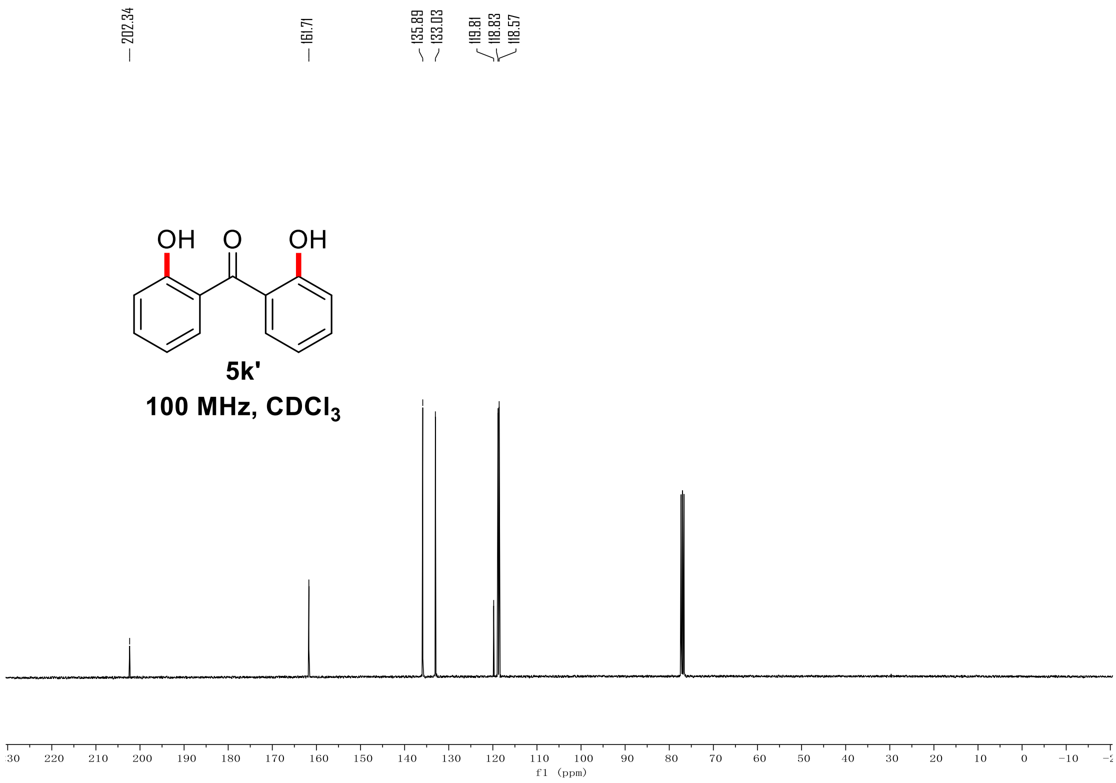
202.34
161.71
135.89
133.03
119.81
118.83
118.57
OH
O
OH
5k'
100 MHz, CDCl3
220
210
200
190
180
170
160
150
140
130
120
110
100
90
80
70
60
50
40
30
20
10
0
−10
f1 (ppm)

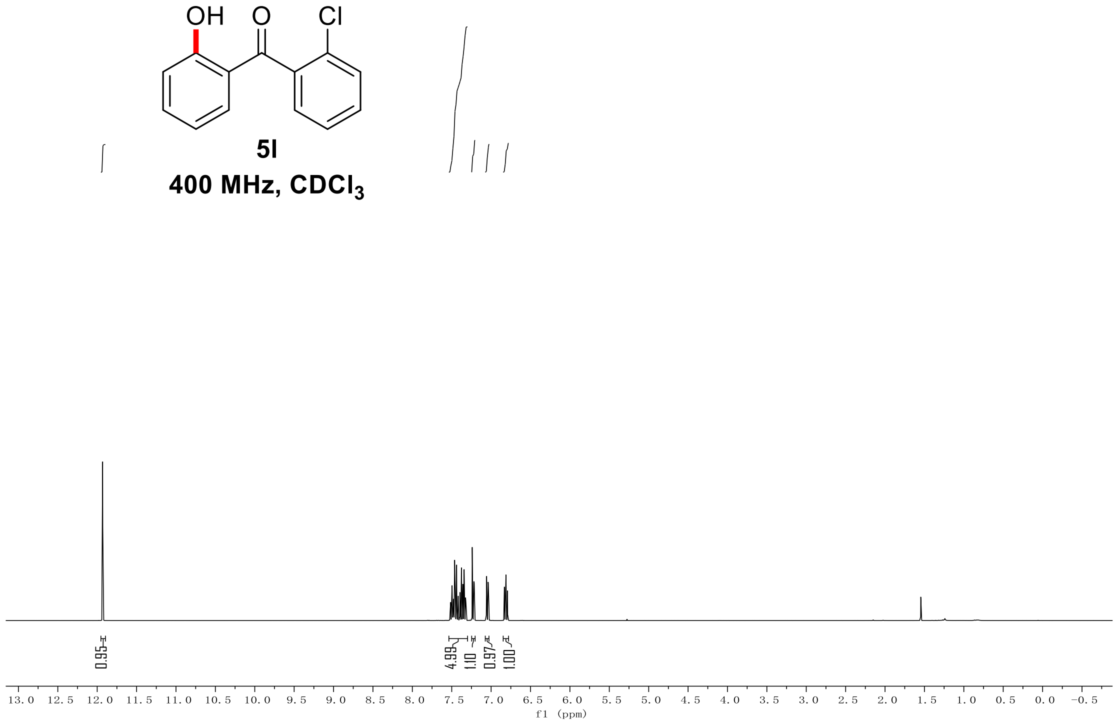
OH
O
Cl
5l
400 MHz, CDCl3
0.95
4.99
1.10
0.97
1.00
f1 (ppm)

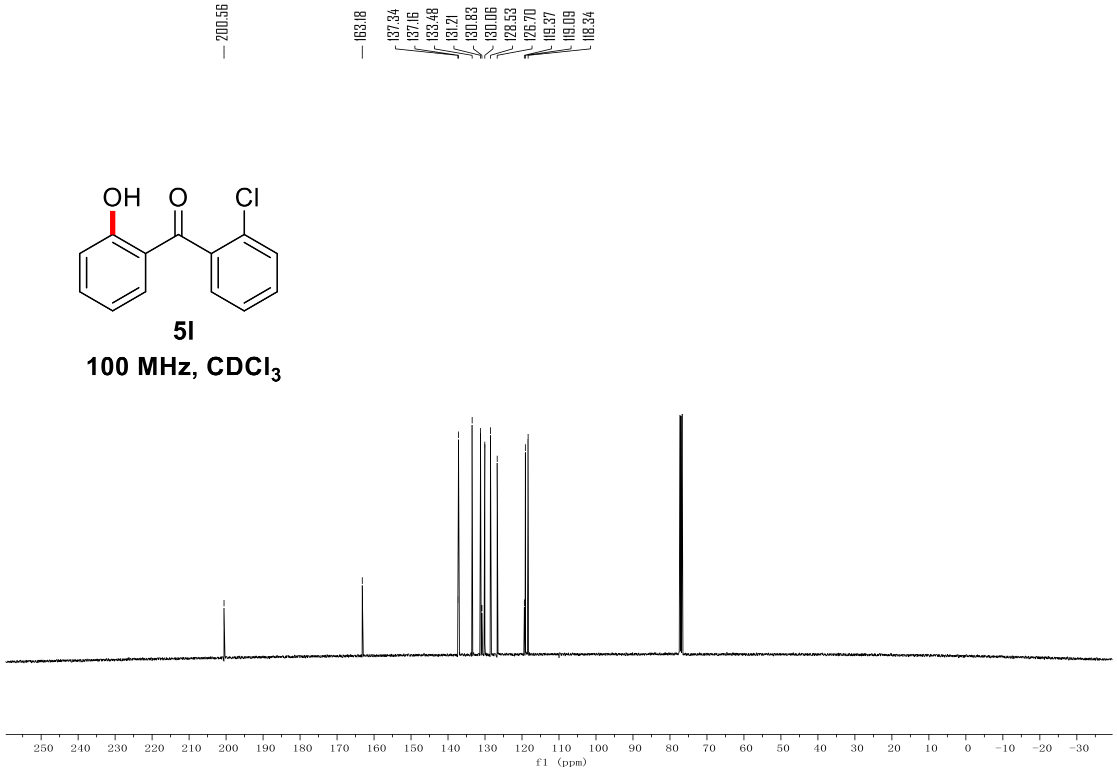
200.56
163.18
137.34
137.16
133.48
131.21
130.83
130.06
128.53
126.70
119.37
119.09
118.34
OH
O
Cl
5l
100 MHz, CDCl3
f1 (ppm)

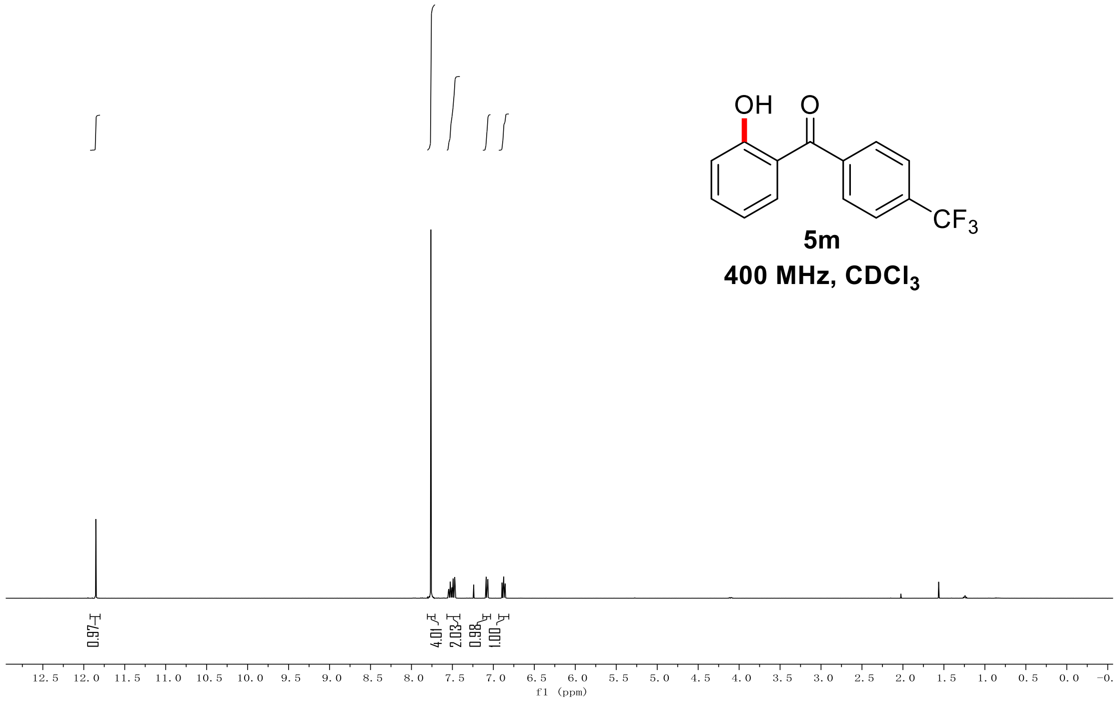
OH
O
CF3
5m
400 MHz, CDCl3
0.97
4.01
2.03
0.98
1.00
12.5 12.0 11.5 11.0 10.5 10.0 9.5 9.0 8.5 8.0 7.5 7.0 6.5 6.0 5.5 5.0 4.5 4.0 3.5 3.0 2.5 2.0 1.5 1.0 0.5 0.0
f1 (ppm)

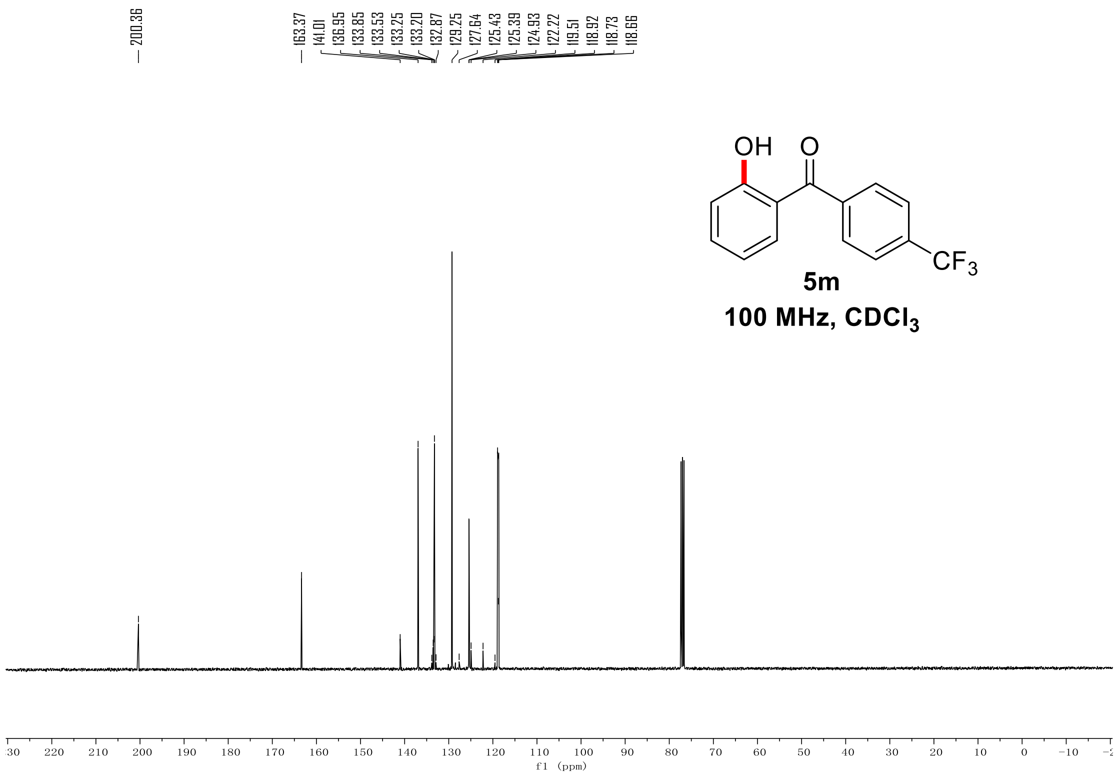
200.36
163.37
141.01
136.95
133.85
133.53
133.25
133.20
132.87
129.25
127.64
125.43
125.39
124.93
122.22
119.51
118.92
118.73
118.66
OH
O
CF3
5m
100 MHz, CDCl3
220 210 200 190 180 170 160 150 140 130 120 110 100 90 80 70 60 50 40 30 20 10 0 −10
f1 (ppm)

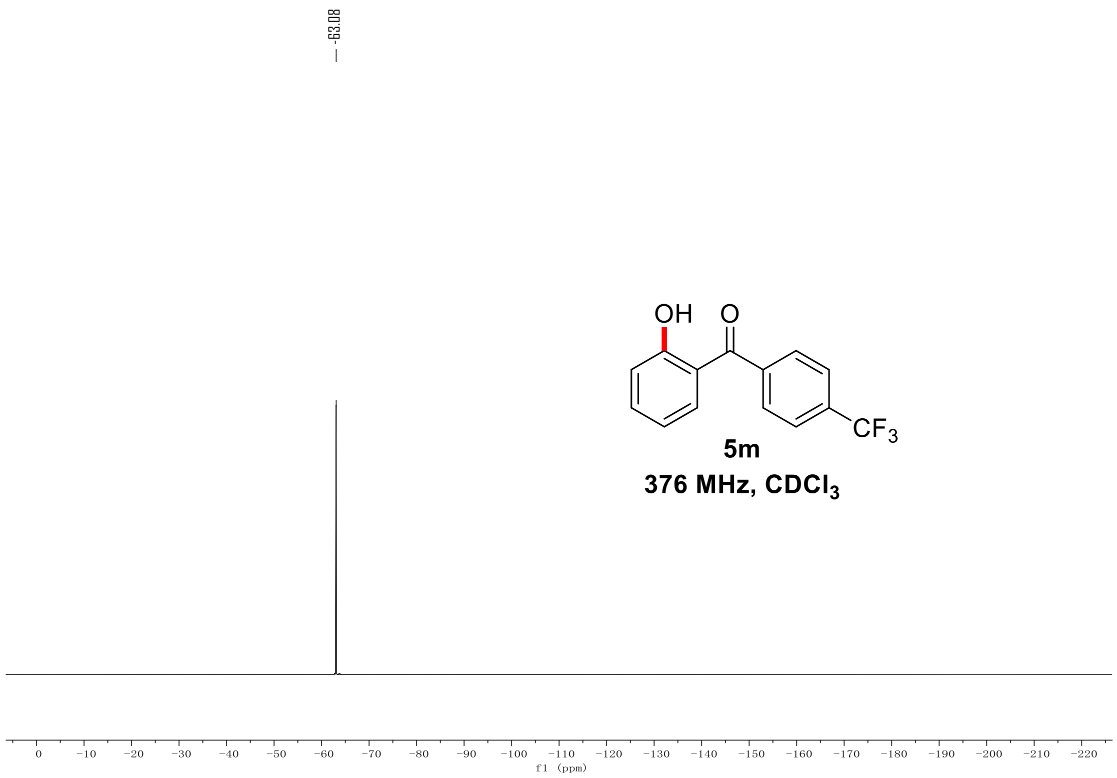
-63.08
OH
O
CF3
5m
376 MHz, CDCl3
0 −10 −20 −30 −40 −50 −60 −70 −80 −90 −100 −110 −120 −130 −140 −150 −160 −170 −180 −190 −200 −210 −220
f1 (ppm)

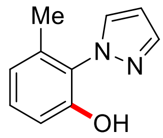

7a

400 MHz, $CDCl_3$

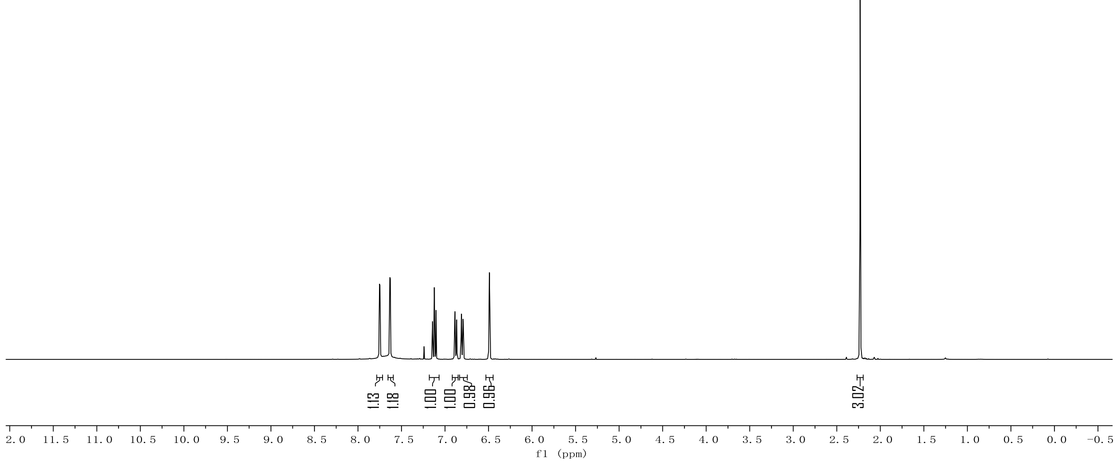

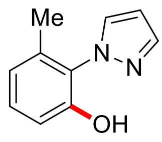

7a

100 MHz, $CDCl_3$

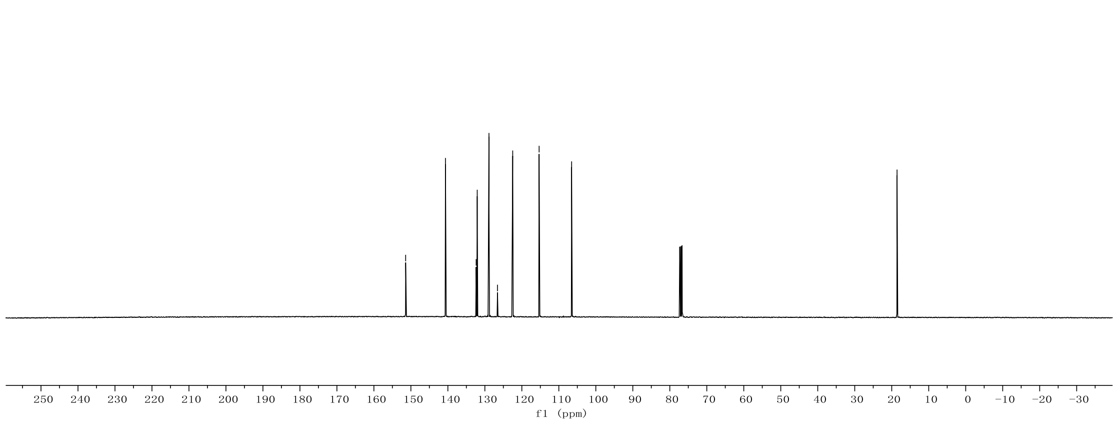

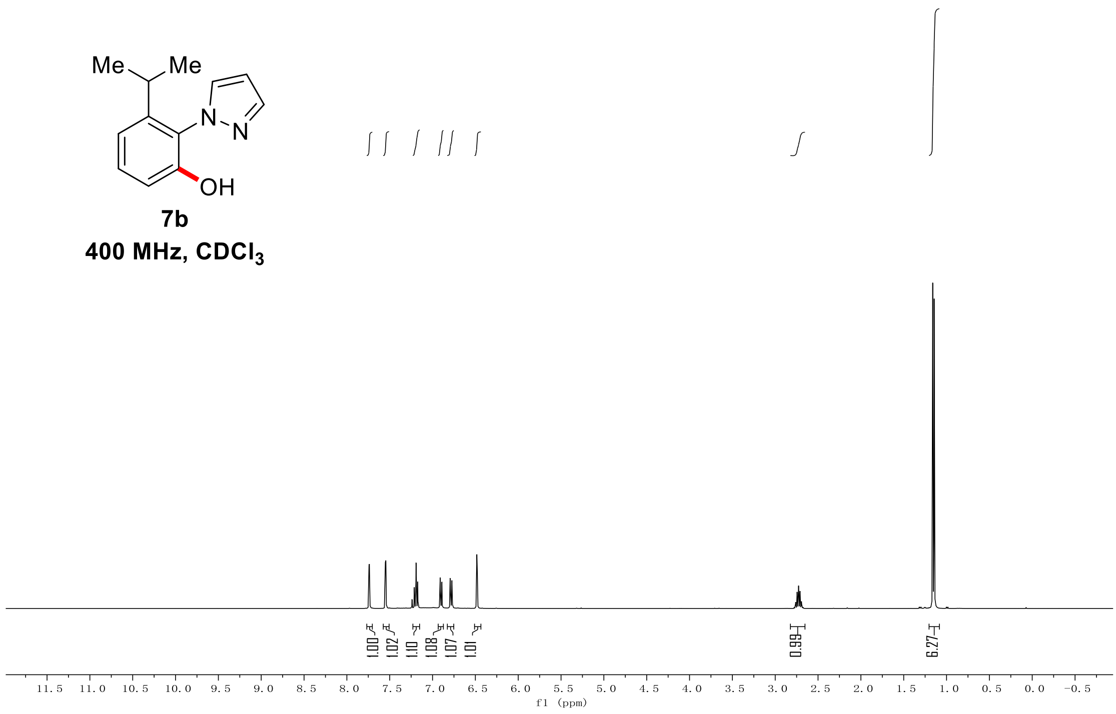

Me
Me
N
N
OH
7b
400 MHz, CDCl3
1.00
1.02
1.10
1.08
1.07
1.01
0.99
6.27
f1 (ppm)

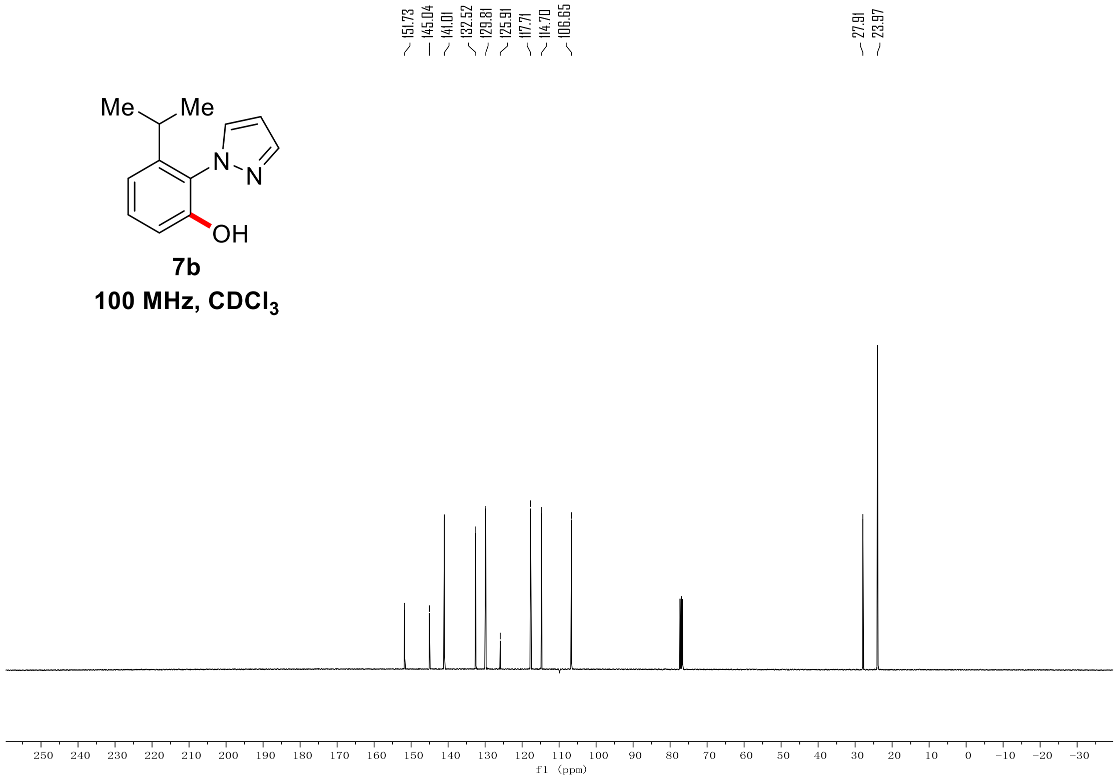

151.73
145.04
141.01
132.52
129.81
125.91
117.71
114.70
106.65
27.91
23.97
Me
Me
N
N
OH
7b
100 MHz, CDCl3
f1 (ppm)

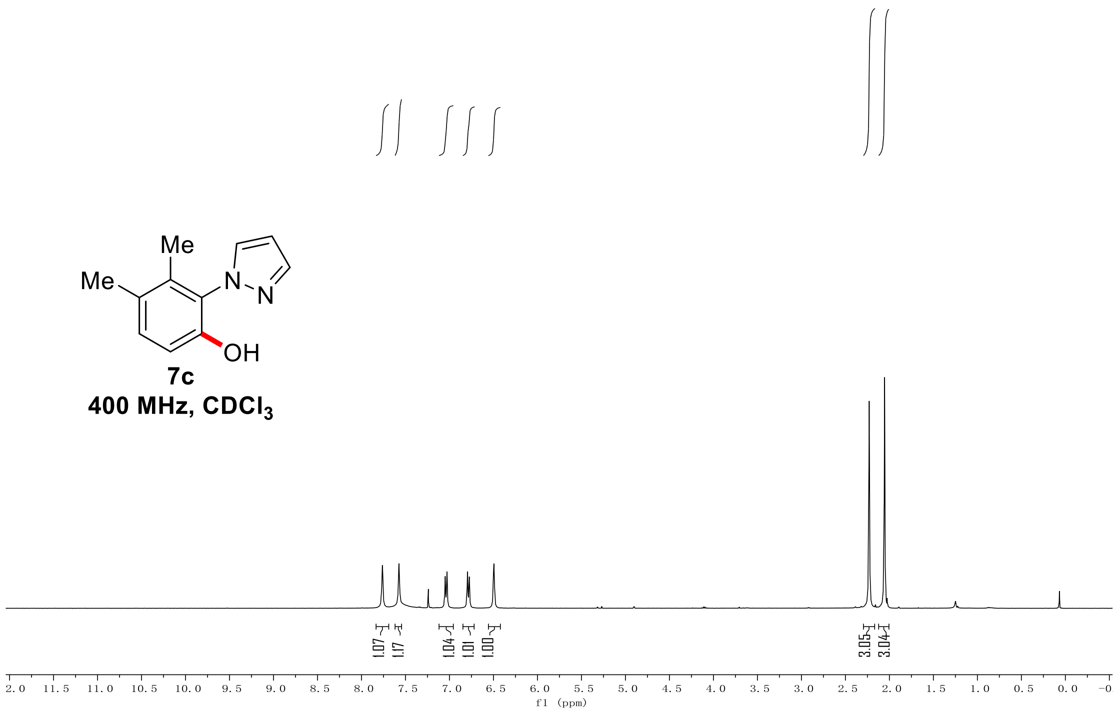
Me
Me
N
N
OH
7c
400 MHz, CDCl3
1.07
1.17
1.04
1.01
1.00
3.05
3.04
f1 (ppm)

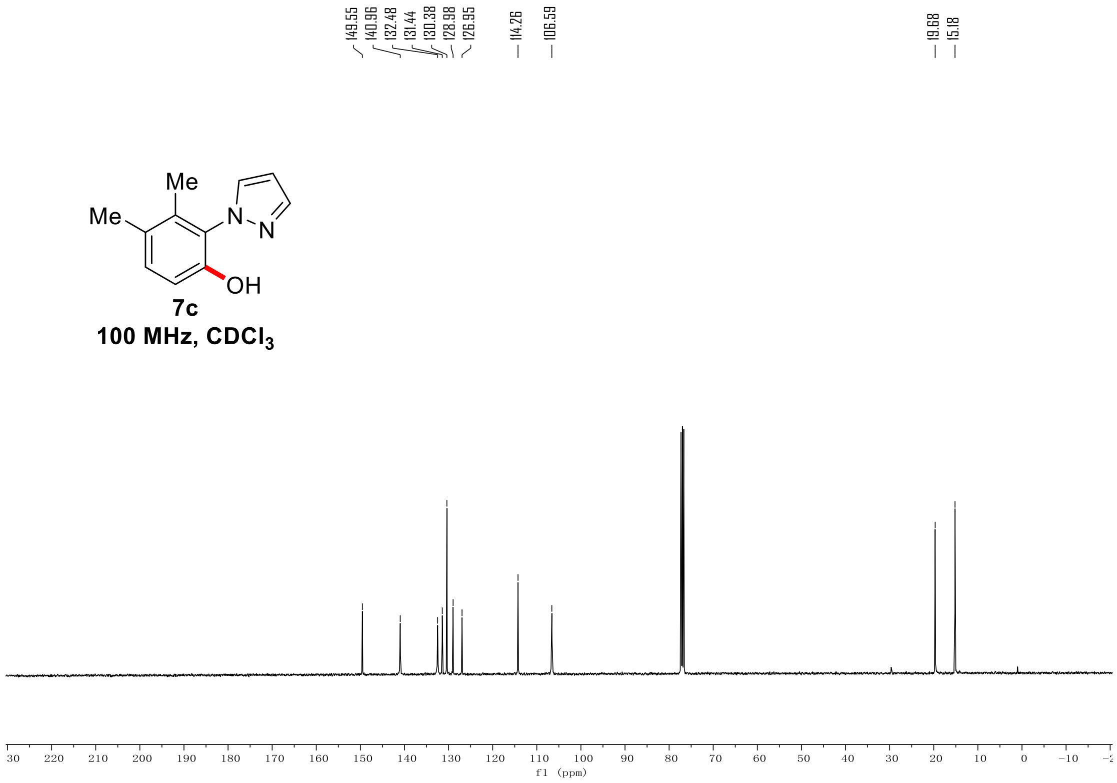
149.55
140.96
132.48
131.44
130.38
128.98
126.95
114.26
106.59
19.68
15.18
Me
Me
N
N
OH
7c
100 MHz, CDCl3
f1 (ppm)

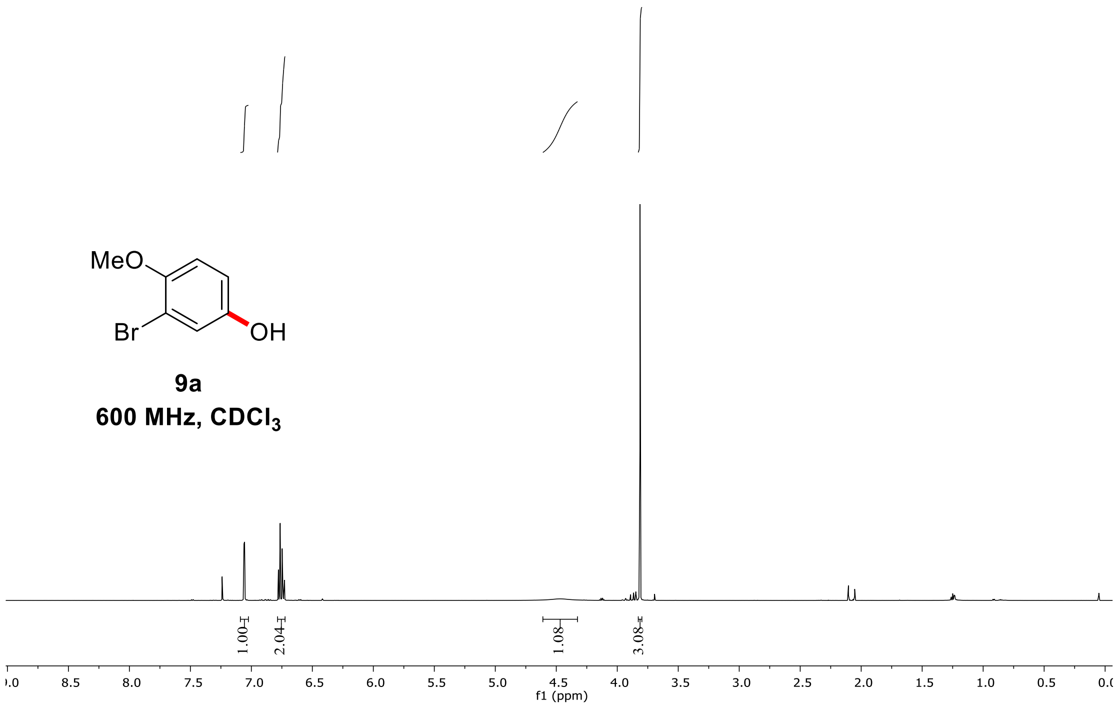
MeO
Br
OH
9a
600 MHz, CDCl3
1.00
2.04
1.08
3.08
f1 (ppm)

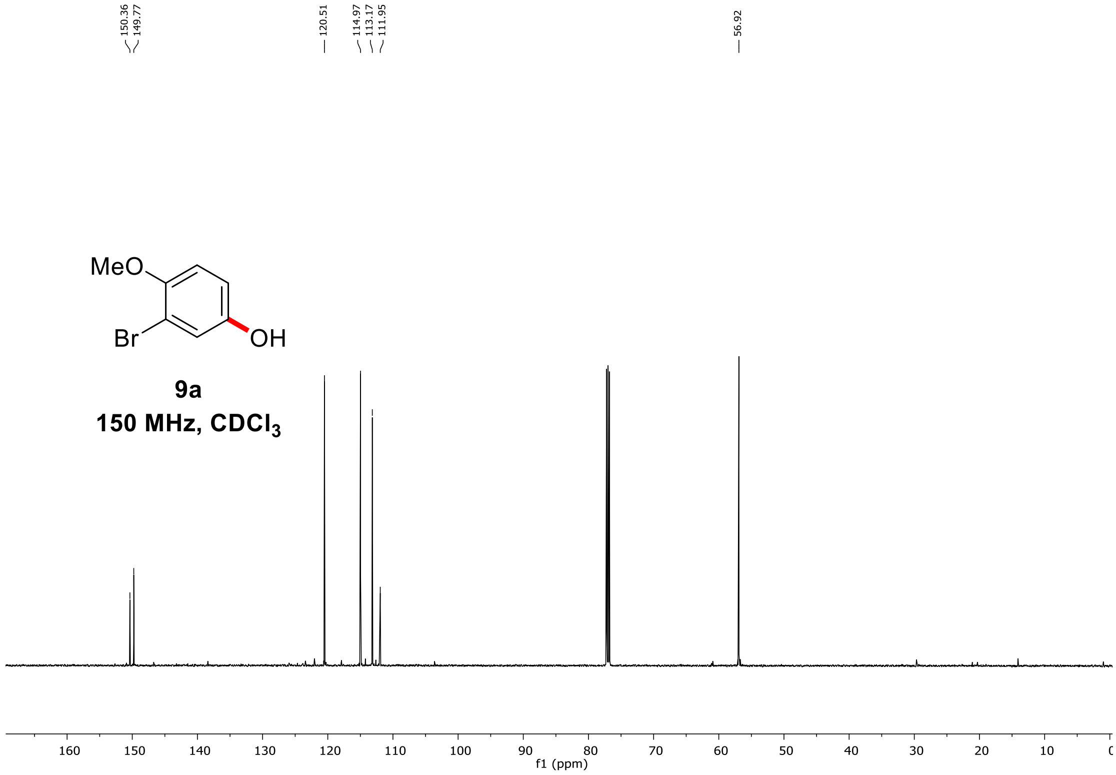
150.36
149.77
120.51
114.97
113.17
111.95
56.92
MeO
Br
OH
9a
150 MHz, CDCl3
f1 (ppm)

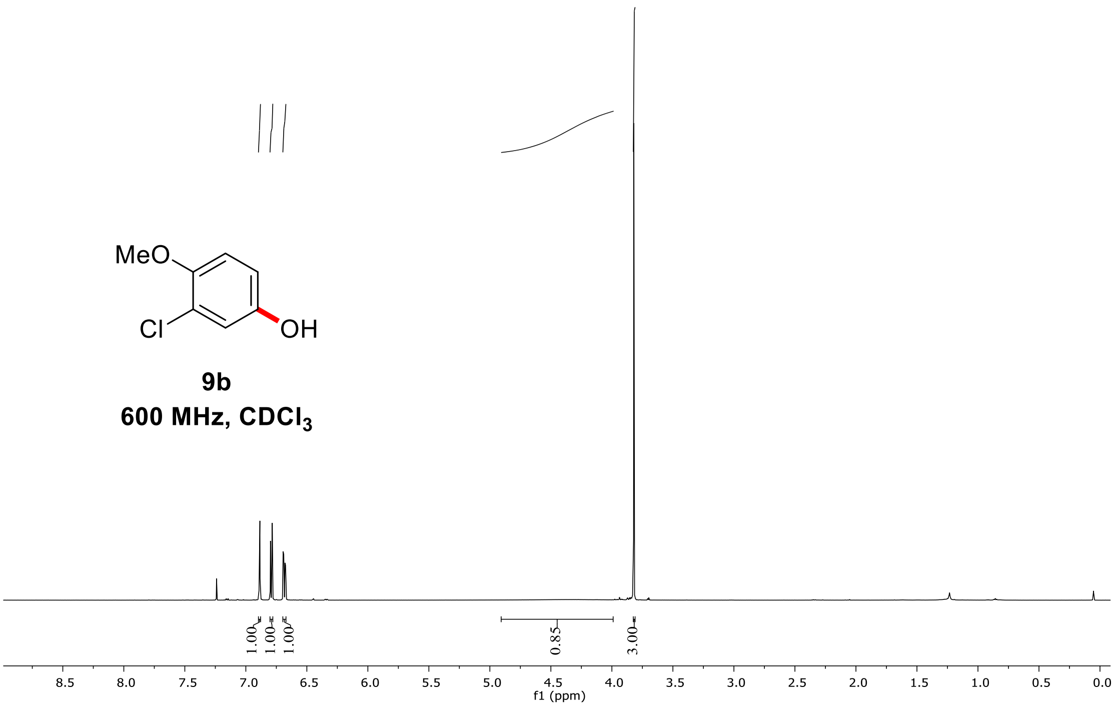

9b
600 MHz, $CDCl_3$

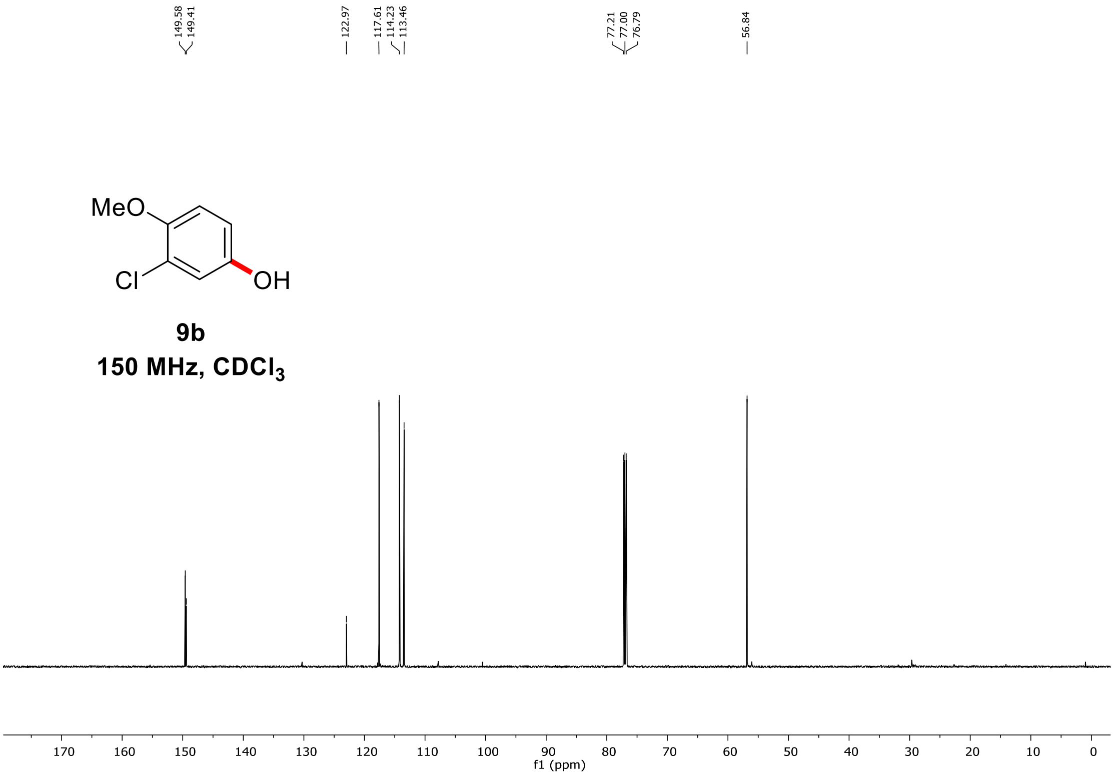

9b
150 MHz, $CDCl_3$

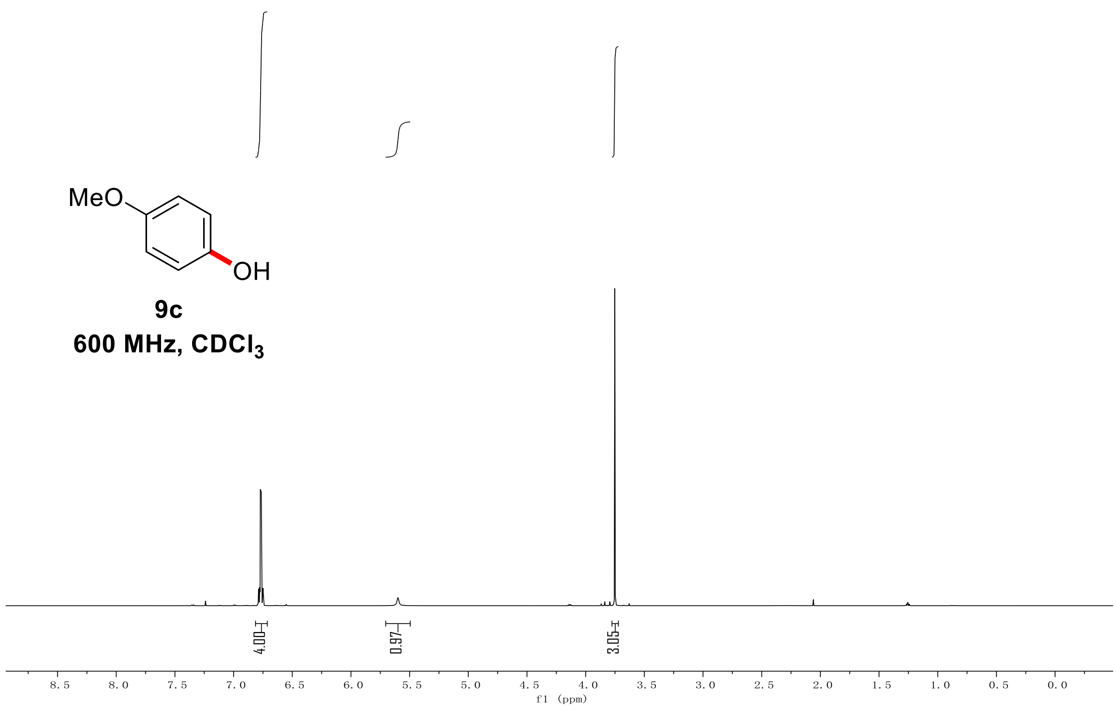

9c
600 MHz, $CDCl_3$

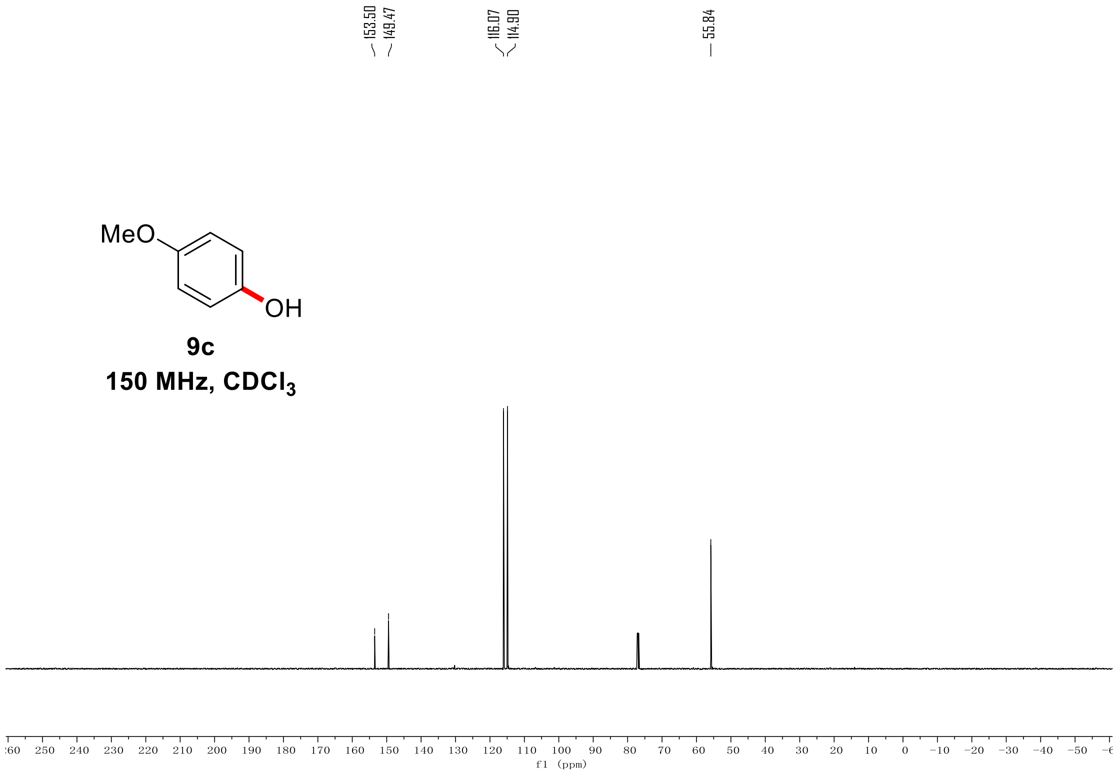

9c
150 MHz, $CDCl_3$
